# Population genomics of *Agrotis segetum* provide insights into the local adaptive evolution of agricultural pests

**DOI:** 10.1186/s12915-024-01844-x

**Published:** 2024-02-20

**Authors:** Ping Wang, Minghui Jin, Chao Wu, Yan Peng, Yanjin He, Hanyue Wang, Yutao Xiao

**Affiliations:** 1 Shenzhen Branch, Guangdong Laboratory of Lingnan Modern Agriculture, Key Laboratory of Gene Editing Technologies (Hainan), Ministry of Agriculture and Rural Affairs, Agricultural Genomics Institute at Shenzhen, Chinese Academy of Agricultural Sciences, Shenzhen, China; 2 School of Life Sciences, Henan University, Kaifeng, 475004 China; 3 Shenzhen Research Institute of Henan university, Shenzhen, 518000 China

**Keywords:** *Agrotis segetum*, Population genomics, Local adaptation, Evolution

## Abstract

**Background:**

The adaptive mechanisms of agricultural pests are the key to understanding the evolution of the pests and to developing new control strategies. However, there are few studies on the genetic basis of adaptations of agricultural pests. The turnip moth, *Agrotis segetum* (Lepidoptera: Noctuidae) is an important underground pest that affects a wide range of host plants and has a strong capacity to adapt to new environments. It is thus a good model for studying the adaptive evolution of pest species.

**Results:**

We assembled a high-quality reference genome of *A. segetum* using PacBio reads. Then, we constructed a variation map of *A. segetum* by resequencing 98 individuals collected from six natural populations in China. The analysis of the population structure showed that all individuals were divided into four well-differentiated populations, corresponding to their geographical distribution. Selective sweep analysis and environmental association studies showed that candidate genes associated with local adaptation were functionally correlated with detoxification metabolism and glucose metabolism.

**Conclusions:**

Our study of *A. segetum* has provided insights into the genetic mechanisms of local adaptation and evolution; it has also produced genetic resources for developing new pest management strategies.

**Supplementary Information:**

The online version contains supplementary material available at 10.1186/s12915-024-01844-x.

## Background

Habitat conditions are critical to insect development and reproduction. Over the long course of evolution, insects have developed the ability to rapidly adapt to their local habitat [1, 2]. Faced with the complex and changeable natural and anthropic environments, insects have evolved a series of adaptive strategies, including morphological, physiological, biochemical and molecular adaptations [3, 4]. Understanding these adaptive evolutionary mechanisms is important for developing new prevention and control strategies. Population genomics has been widely used in the analysis of genetic evolution, adaptive evolution, and important traits [5–7]. However, compared with other areas of biology such as plants, the field of agricultural pests remains insufficiently researched.

The turnip moth, *Agrotis segetum* (Lepidoptera: Noctuidae) is a polyphagous underground pest that harms a variety of crops and vegetables, including corn, wheat, cotton, potatoes, and tomatoes [8, 9]. *A. segetum* hides in shallow soil near crops during the day and comes out at night to feed. The larvae chew the stems of crop plants close to the ground, thereby killing the entire plant and causing severe economic and ecological damage [8, 10]. The moth is widely distributed in Europe, Asia, and Africa [11–14]. *A. segetum* is widely distributed in China, spanning multiple climatic environments, which provides a good model studying the environmental adaptability of agricultural pests [14, 15].

In this study, we assembled a high-quality reference genome of *A. segetum* (contig N50 = 2.53 Mb) using PacBio reads. Genome-wide variants, including single-nucleotide polymorphisms (SNPs) and structural variations (SVs), were identified by sequencing the genomes of individuals collected from China; we then analyzed the population structure based on SNPs and SVs. Selective sweep analysis was used to study the local adaptation of *A. segetum*, especially to cold tolerance, pesticide resistance, and host plant adaptability. This study revealed the genetic mechanisms of environmental adaptability of *A. segetum* and thus provides a reference for the study of the adaptive evolutionary mechanism of agricultural pests. The results can be employed to guide the development and application of new strategies for agricultural pest management.

## Results

### Genome variation and population structure among all accessions

**Fig. 1:**
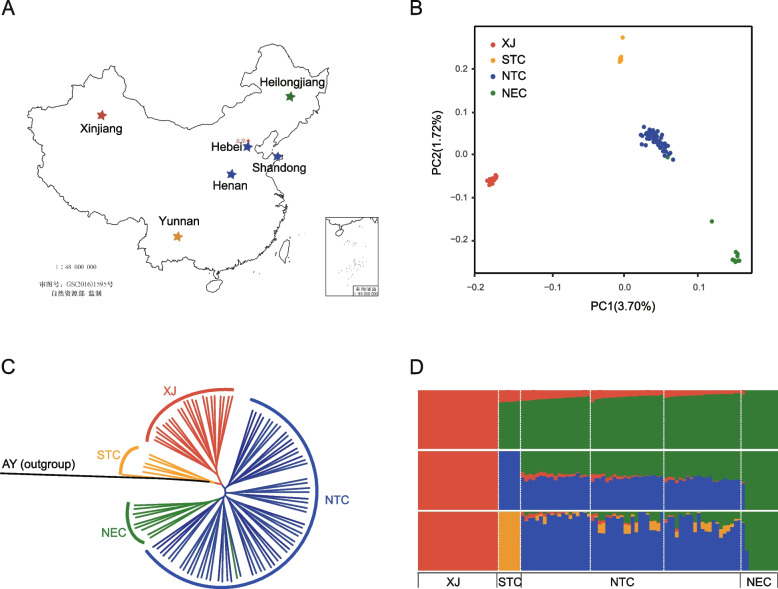
Geographical distribution and population structure of *A. segetum*. **A** Location diagram of sampling sites in Xinjiang region, North China (Henan, Hebei, Shandong), Northeast China (Heilongjiang), and South China (Yunnan). **B** PCA plot of the first two components with SNPs, with different colors representing different populations. **C** ML tree based on SNPs with *Agrotis ipsilon* (AY) as the outgroup. **D** Population structure analysis based on SNPs (*K*=2–4). The colors in each column represent the proportion of individual genomes in each ancestral population

A total of 35.82 Gb of PacBio reads were used to assemble a high-quality reference genome of *A. segetum* with an assembled size of 600 Mb and a contig N50 length of 2.53 Mb (Additional file 1) [16–33]. We re-sequenced 98 samples from six natural populations in North China (NTC), Northeast China (NEC), Xinjiang (XJ), and South China (STC) (Fig. 1A; Additional file 3: Table S4) and obtained 1811 Gb of high-quality clean reads after filtering. The average sequencing depth of these samples was 27.5× (Additional file 3: Table S5). Based on the reference genome of *A. segetum*, we generated a total of 1,065,969 high-quality SNPs, and annotated 1,478,705 SNPs using SnpEff software. The majority of SNPs (558,109) were located in the intergenic regions, accounting for 37.74%. An additional 18.22% of SNPs were located in coding regions, of which 32,706 were missense mutations and 236,797 were synonymous mutations. The numbers of SNPs located in introns and upstream or downstream of genes were 237,797 (16.08%), 189,533 (12.81%), and 207,960 (14.06%) respectively (Additional file 3: Table S6). We obtained a set of 35,069 SVs that were larger than 50 bp, including deletions (DEL), duplications (DUP), insertions (INS), and inversions (INV), of which DEL accounted for the majority (92.6%) (Additional file 2: Fig. S6; Additional file 3: Table S7).

To clarify the population structure of *A. segetum*, we used the SNPs with minor allele frequency (MAF) > 0.05 and linkage disequilibrium threshold (*r*^2^) < 0.05 to explore the relationships between different natural populations. The phylogenetic trees were constructed with *Agrotis ipsilon* as the outgroup based on the maximum likelihood (ML) method using SNPs (Fig. 1C). All the accessions were divided into four groups, namely XJ, STC, NEC, and NTC. The samples collected from different provinces of NTC were clustered into one branch on the evolutionary tree. Principal component analysis (PCA) showed clear genetic structure (Fig. 1B). Both PC1 and PC2 were divided into four groups, consistent with the phylogenetic tree. We further analyzed the population structure using ADMIXTURE (Additional file 2: Fig. S7). A value of *K*=4, there was a clear population structure and it was consistent with the results of phylogenetic tree and PCA (Fig. 1D). In addition, we analyzed the phylogenetic relationships of these re-sequenced individuals using SVs. The ML tree showed similar phylogenetic relationships (Additional file 2: Fig. S8), and the PCA and ADMIXTURE (K=4) results were consistent with the results from SNPs (Additional file 2: Figs. S9-S10)

### Population diversity and demographic history

**Fig. 2:**
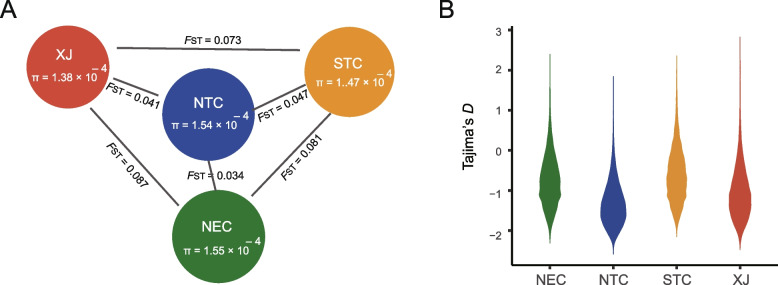
Population diversity and demographic history of *A. segetum*. **A** The genetic differentiation (*F*_ST_) and genetic diversity (*π*) between populations. The radius of the circle represents the size of the genetic diversity, and the length of the line represents the *F*_ST_ values between pairwise populations. **B** Violin plot of the genome-wide distribution of Tajima’s *D* values in NEC, NTC, STC, and XJ

To analyze the degree of population differentiation, we calculated the fixation index (*F*_ST_) between populations (Fig. 2A; Additional file 2: Fig. S11). The results showed that the *F*_ST_ values of XJ, STC, and NEC populations were higher and there was significant genetic difference. The level of genetic difference between NTC and the other three populations was low and the level of genetic difference between NTC and NEC populations was the least, results that were consistent with the phylogenetic analyses. We also calculated the nucleotide diversity (*π*) of each population to assess the level of genetic diversity. The results of *π* showed that the genetic diversity of XJ population (*π*=1.38×10^−4^) was the lowest. The nucleotide diversity of the NTC population (*π*=1.54×10^−4^) was very similar to that of NEC population (*π*=1.55×10^−4^), showing a high level of genetic diversity. The mean values of Tajima’s *D* of the four populations were negative, indicating that there were many low-frequency alleles in the populations (Fig. 2B). The negative value of Tajima’s *D* accounted for a high proportion in NTC and XJ, while STC accounted for the lowest proportion. The TreeMix result indicated that there was gene flow between the NEC and NTC populations, consistent with the results of the population structure analysis (Additional file 2: Fig. S12). We inferred the demographic history of *A. segetum* using PSMC. We found that the effective population sizes of the four populations decreased during the last glaciation (LG), and then gradually increased and expanded, among which the XJ population first differentiated independently (Additional file 2: Fig. S13).

### Selective signals for each population

Based on the present results, *A. segetum* was divided into four populations in China, distributed according to different geographical and climatic conditions. The populations of *A. segetum* may have evolved unique strategies to adapt to the local environments. Thus, we conducted composite likelihood ratio (CLR) analyses for each population to identify potential signatures of selective sweeps. The results of CLR analyses showed that 562 regions containing 539 genes were identified in the NTC population (Additional file 2: Fig. S14A; Additional file 3: Table S8). KEGG enrichment analysis showed that these genes were significantly enriched in pathways such as mineral absorption and ABC transporters (Additional file 2: Fig. S14B). ABC transporters mediate the efflux of compounds from the cytoplasm to the outside of the cell or into organelles and play multiple functions in xenobiotic transport and resistance in insects [34–36]. We identified 451 regions containing 537 genes that were selected in the NEC population. KEGG enrichment analysis showed that butanoate metabolism, the p53 signaling pathway, and tyrosine metabolism were significantly enriched (Additional file 3: Table S9; Additional file 2: Fig. S15). Among the selected genes, the gene collagen alpha-1 (IV) chain (*COL4A1*) exhibited strong selection. COL4A1 is an important component of the insect basement membrane and is crucial to the development of *Drosophila* and *Anopheles gambiae* [37]. Studies have shown that this gene may be related to temperature-sensitive lethality in silkworms [38]. In the XJ population, we identified 453 regions containing 463 genes. These selected genes were significantly enriched in spliceosome and the Hippo signaling pathway (Additional file 3: Table S10; Additional file 2: Fig. S16). Among the selected genes, the transformation growth factor regulator 1 (*TBRG1*) gene appeared to be under strong selection. TGF-β signaling is an important pathway affecting the development and differentiation of insects. The downregulation of TGF-β in *Helicoverpa armigera* can block developmental signals and induce pupal diapause[39, 40]. We identified 358 regions in the STC population, including 468 genes that were selected (Additional file 2: Fig. S17A; Additional file 3: Table S11). KEGG enrichment analysis showed that these genes were significantly enriched in pathways such as the p53 signaling pathway, ECM-receiver interaction, and nucleocytoplasmic transport (Additional file 2: Fig. S17B). We found that the odorant-binding protein (OBP) genes were under strong selection. The OBP is involved in the regulation of insect host recognition, foraging, courtship, and other behaviors [41].

### Genomic differential selection between populations

**Fig. 3:**
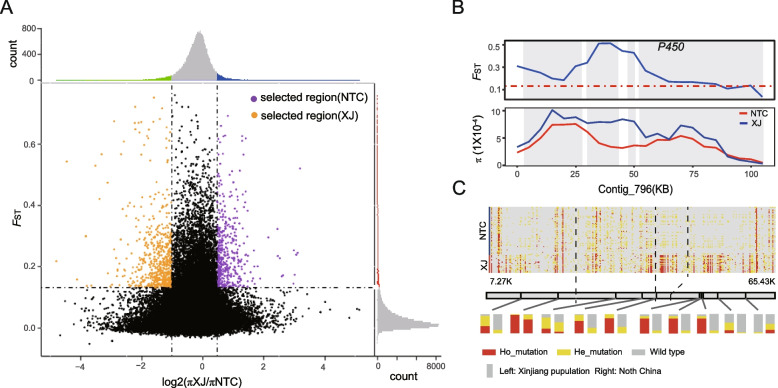
Selective sweep analysis and selected region between XJ and NTC populations. **A** Distribution of logarithmic ratio of *π* (log2(*π*XJ/*π*NTC)) and *F*_ST_ values. The dotted line represents the 5% threshold, and the common data points above the right (left) vertical dotted line and the horizontal dotted line were identified as the selected region of NTC(XJ) (orange was the selected region of XJ and purple was the selected region of NTC). **B** *F*_ST_ and *π* of the strongly selective signal P450 genes in XJ and NTC. **C** Locus genotypes of P450 genes. The bar chart showed the frequency of missense mutant alleles, and the colors represented the types of alleles

To further analyze the adaptability of populations to the local environments, we carried out the selective sweep analyses between populations based on *F*_ST_ and *π*. We calculated pairwise *F*_ST_ values and the logarithmic ratio of *π* between pairwise populations, and then selected the top 5% outlier regions as candidate selected regions. The selected region (Fig. 3A) between XJ and NTC populations included 203 genes selected in NTC population (*F*_ST_ > 0.132 and log2 (*π* XJ/*π* NTC) > 0.471) (Additional file 3: Table S12) and 263 genes selected in XJ (*F*_ST_ > 0.132 and log2 (*π* XJ/*π* NTC) < −1.017) (Additional file 3: Table S13). KEGG enrichment analysis showed that the selected region in NTC population was significantly enriched in fatty acid metabolism, terpenoid backbone biosynthesis, and the longevity regulating pathway. The selected region in XJ population was mainly enriched in pathways such as steroid hormone biosynthesis, retinol metabolism, and axon regeneration. Cytochrome P450 (P450) is involved in detoxification of harmful substances in host plants and synthetic pesticides and plays an important role in host adaptation and pesticide resistance of insects [42, 43]. We found that there were many P450 genes in the NTC population selected region, among which four P450 genes (about 103 Kb) showed strong signals of selection (Fig. 3B), and there was significant haplotype differentiation between the NTC and XJ populations. This region contained 135 synonymous mutation SNPs and 49 missense mutation SNPs. The missense mutation SNPs can lead to amino acid changes. Ten of missense mutation SNPs had significant allele frequency differences between the two populations (Fig. 3C; Additional file 3: Table S14). Insect gustatory receptors can perceive taste, regulate insect feeding behavior, and play key roles in host plant selection [44]. We also found some *GR* (gustatory receptor) genes in the selected region of NTC population, which may possibly be related to the different crop planting structures of the two regions.

**Fig. 4:**
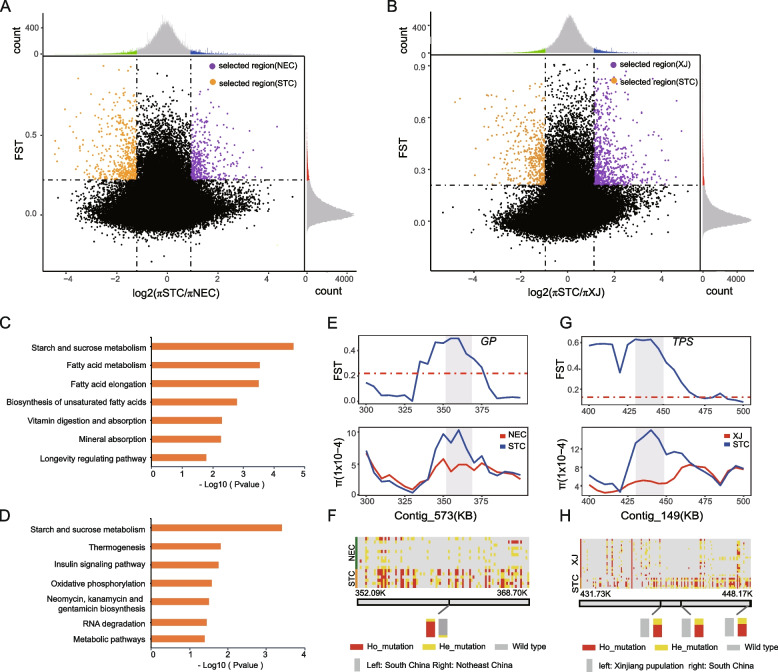
Selective sweep analysis and selected region between STC and NEC (XJ) populations. **A** Distribution of logarithmic ratio of *π* (log2(*π* XJ/*π* NTC)) and *F*_ST_ values of STC and NEC. The dotted line represents the 5% threshold, and the common data points above the right (left) vertical dotted line and the horizontal dotted line were identified as the selected region of STC (NEC) (orange was the selected region of STC and purple was the selected region of NEC). **B** Distribution of logarithmic ratio of *π* (log2(*π* XJ/*π* NTC)) and *F*_ST_ values of STC and XJ. Orange is the selected region of STC, purple is the selected region of XJ. **C**, **D** KEGG enrichment of genes in NEC (**C**) and XJ (**D**) selected regions. The horizontal coordinate is the *p*-value of the pathway. **E** *F*_ST_ and *π* values of strongly selective signaling gene *GP*. **F** Locus genotypes of *GP*. The bar chart showed the frequency of missense mutant alleles, and the colors represented the types of alleles. **G** *F*_ST_ and *π* values of gene *TPS*. **H** Locus genotypes of *TPS*

We performed selective sweep analyses between STC and NEC (or XJ) populations to identify outlier regions (Fig. 4A, B). The selected regions between STC and NEC populations included 214 genes in NEC population (*F*_ST_ > 0.221 and log2 (π STC/π NEC) > 0.922) and 210 genes in STC population (*F*_ST_ > 0.221 and log2 (π STC/π NEC) < −1.20902) (Additional file 3: Tables S15-S16). The XJ population identified 279 candidate genes (*F*_ST_ > 0.209 and log2 (*π* STC/*π* XJ) > 1.124), and the STC population identified 184 candidate genes (*F*_ST_ > 0.209 and log2 (*π* STC/*π*XJ) < −0.94984) in the selected regions between STC and XJ populations (Additional file 3: Table S17-S18). KEGG enrichment analysis of the NEC selected region showed that these genes were significantly enriched in the pathways of starch and sucrose metabolism, fatty acid elongation, and unsaturated fatty acid synthesis (Fig. 4C); the genes of the selected region of XJ population were significantly enriched in starch and sucrose metabolism, thermogenesis, and the insulin signaling pathway (Fig. 4D). *A. segetum* can overwinter to adapt to the low temperature climate [9]. Genes related to starch and sucrose metabolism were significantly enriched in both NEC and XJ populations, suggesting that glucose metabolism may play an important role in the cold tolerance of *A. segetum*. The previous study of Huang et al. [45] was consistent with our conclusions. In addition, fatty acids, as substrates for fat synthesis, also affect the cold tolerance of insects [46].

Glycogen phosphatase (GP) is a rate-limiting enzyme that degrades glycogen. By degrading glycogen, insects can accumulate cryoprotectants such as glycerol and trehalose to improve their cold tolerance [47, 48]. In the starch and sucrose metabolism pathway, we found that the gene *GP* had strong selective signals in NEC and XJ (Fig. 4E; Additional file 2: Fig. S18A). The gene *GP* showed significant haplotype differentiation in the two populations (STC and NEC (or XJ)). There were two missense mutation loci in this gene, one of which had a significant difference in the frequency of missense mutation alleles between the two populations (Fig. 4F; Additional file 3: Table S14). Research has shown that the GP activity of *Heortia vitessoides* [49] can be activated under cold stress. Trehalose, the main blood sugar of insects, can act as an antifreeze to help insects withstand low temperature [50]. Trehalose synthase is a key enzyme in the trehalose biosynthesis pathway. The gene *TPS* (trehalose synthase) in the starch and sucrose metabolic pathway was also strongly selected (Fig. 4G; Additional file 2: Fig. S18B), and the haplotype differentiation of *TPS* was also evident in both populations. SNP annotation showed that three missense mutation loci (from a total of five) had significantly different allele frequencies (Fig. 4H; Additional file 3: Table S14). Previous studies have shown that cold-resistant substances, including trehalose, are significantly increased in the body of *A. segetum* under low-temperature exercise [45]. Trehalose was also found to be involved in regulating the diapause of *H. armigera*, and TPS is closely related to trehalose content [51]. Through population selection analysis and environment association analysis of cotton bollworm, a series of important low-temperature adaptation genes including *TPS* genes were identified [52]. We speculated that the differences in *GP* and *TPS* between populations might also be related to the low-temperature adaptation of *A. segetum*. Pairwise selective sweep analyses between other populations (XJ and NEC, STC and NTC, and NTC and NEC) were also carried out, and a series of candidate genes were identified in their selected regions (Additional file 3: Tables S19-S24).

### Environmental association analysis of *A. segetum*

**Fig. 5:**
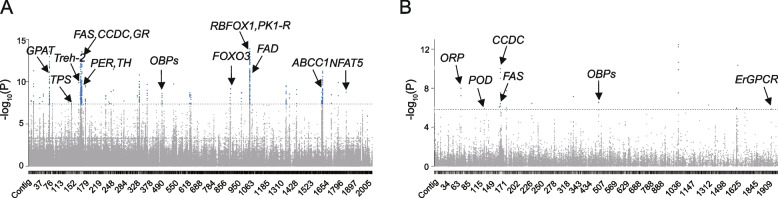
Association analysis of local environmental adaptation. **A** Manhattan plot of latitude association analysis based on SNPs. The blue dots were associated regions and the annotated genes were associated genes. **B** Manhattan plot of latitude association analysis based on SVs

We conducted environmental association analysis on all materials, considering three selected environmental factors: latitude, annual mean temperature (AMT), and minimum temperature in the coldest quarter (MTCQ) (Additional file 3: Table S25). These factors have crucial effects on insect adaptation, making them suitable for genotype-environment association analysis. We first analyzed the correlation between these environmental factors and SNPs. Using GEMMA, we identified a set of latitude-associated loci (Fig. 5A), including the genes *RBFOX1* (RNA-binding protein fox-1), *PK1-R* (pyrokinin-1 receptor), and *CCDC* (coiled-coil domain-containing protein AGAP005037). KEGG enrichment analysis showed that the unsaturated fatty acid synthesis, longevity regulating pathway, and starch and sucrose metabolism were significantly enriched, as well as several important signaling pathways such as AMPK and PPAR signaling (Additional file 2: Fig. S19). We searched for genes co-associated with latitude in the selected regions of NEC and XJ (from the selective sweep analyses between STC and NEC (or XJ)). Seven genes were identified (Table 1), including the *TPS* mentioned above. The gene with the highest *p*-value was *AS006811*, which is presumed to be closely related to latitude. However, the specific function of this gene has not been annotated, and further research is needed. The genes strongly associated with AMT and MTCQ were similar (Additional file 2: Fig. S20; Additional file 3: Table S26), among which the gene most markedly associated with temperature was *NURF* (nucleosome remodeling factor subunit). NURF is a member of the ISWI chromatin remodeling complex family, and it regulates gene expression through epigenetic modification and is a key regulatory factor in the development of various organisms [53, 54]. The genotype-environment association analysis using FaST-LMM well supports the previous results, and there is a considerable degree of overlap in the loci associated with the GEMMA analyses (Additional file 2: Fig. S21). Specifically, we found that there were 42 common genes in the two association analyses with latitude (Additional file 2: Fig. S22A). There were 50 common genes associated with AMT and 19 common genes associated with MTCQ (Additional file 2: Fig. S22B, C).

**Table 1:** Common genes of latitude association and selective sweep analysis using SNPs

Gene ID	Contig	*p*_value	YN/HLJ	YN/XJ	Annotation
AS006811	contig_171	5.04E−14	√	√	NA
AS006812	contig_171	4.36E−13	√	√	NA
AS016141	contig_933	6.73E−10	√	√	Forkhead box protein O3
AS017929	contig_1887	1.75E−09	√	√	Nuclear factor of activated T-cells 5
AS009080	contig_238	4.63E−09	√	√	Gustatory receptor
AS012477	contig_378	1.57E−08	√	√	NA
AS005143	contig_149	4.19E−08	√	√	Trehalose 6-phosphate synthase

We also performed environmental association analyses for all accessions using SVs. A total of nine genes were significantly associated with latitude (Fig. 5A; Table 2). Among these, seven genes were consistent with the latitude association analysis using SNPs. Two genes were significantly associated with temperature (Additional file 2: Fig. S23; Additional file 3: Table S27). Our results showed that many selected genes were not only selected at the SNP level, but also selected at the SV level.

**Table 2:** Summary of latitude-associated genes using SVs

Gene ID	Contig	*p*_value	Type	SNPs	Annotation
AS006396	contig_171	9.63E−11	DEL	√	Coiled-coil domain-containing protein AGAP005037
AS002712	contig_76	1.02E−08	DEL	√	Oxysterol-binding protein-related protein 3/6/7
AS011775	contig_339	7.54E−08	DEL	√	Exportin-1
AS013371	contig_505	2.82E−07	DEL	√	Odorant-binding protein
AS013370	contig_505	3.11E−07	DEL	√	Odorant-binding protein
AS006305	contig_169	8.65E−07	DEL	√	Fatty acid synthase
AS007242	contig_183	1.09E−06	DEL	√	Uncharacterized protein LOC111357700
AS004632	contig_138	1.35E−06	DEL	NA	Peroxidase
AS018442	contig_1972	1.36E−06	DEL	NA	Ecdysone-responsive G-protein coupled protein-2

## Discussion

In this study, we assembled a 600 Mb high-quality reference genome of *A. segetum* using PacBio reads. We sequenced the genomes of individuals from six natural populations in China, and constructed genomic variation maps based on SNPs and SVs. The results were used to study the population structure and genetic diversity of *A. segetum*. We found that all individuals were divided into four groups based on SNPs and SVs that corresponded to the geographic distribution. The Xinjiang region is surrounded by mountains and is relatively closed, forming an independent population with low genetic diversity. Individuals from the North China region clustered in a group, probably because the North China Plain is relatively flat and the moths could travel long distances [15]. The genetic difference between North China and Northeast China populations was the least, and gene flow occurred between the two regions, possibly corresponding to the migration of *A. segetum* [55]. Tajima’s* D* indicated that there were large numbers of low-frequency alleles in the populations that might be the result of directed selection or population expansion.

Evidence of local adaptation can be found by selective sweep analysis. Many P450 genes differed between the North China and Xinjiang populations. P450 is an important detoxifying metabolic enzyme that has been shown to be involved in host plant adaptation and pesticide resistance of many insects [42]. In North China, given the large variety of crops and high pesticide usage, P450 may be involved in the local adaption of *A. segetum*. Gene editing of P450 in *Spodoptera frugiperda* and *H*. *armigera* confirmed that P450 is involved in insect resistance to pesticides [56, 57].

The geographical distribution of species depends not only on their dispersal ability, but also on external environment factors, especially low temperatures. Insects have evolved a variety of coping strategies to adapt to low temperatures, such as morphological strategies (diapause) and physiological and biochemical strategies (e.g., accumulation of cryoprotectants and synthesis of unsaturated fatty acids) [4, 58]. *A. segetum* can overwinter in the north to adapt to low temperatures [9]. After low-temperature induction, the glycogen content in the body was closely related to temperature change, and glycometabolism plays an important role in the cold resistance of *A. segetum* [45]*.* We found that the potentially selected genes in the Northeast China and Xinjiang populations were significantly enriched in the starch and sucrose metabolism pathway, which may be related to the low-temperature adaptation of *A. segetum*. A recent study shows that the cotton bollworm is divided into three populations in China, confirming that the distribution of populations is related to geographical features [52]. Using selective sweep analysis between the Xinjiang and South China populations, researchers identified a series of genes involved in low-temperature adaptation, including the Trehalose transporter gene (*Tret1*) and the Trehalose 6-phosphate synthase gene (*TPS*). The populations of *A. segetum* have similar distribution patterns, and thus may also be related to geographical landscape. We also identified the *TPS* gene as being selected in the Northeast and Xinjiang populations and is correlated with latitude. TPS regulates the synthesis of trehalose, the main blood sugar in insects, and it can help insects resist low temperatures and other adverse environments. It has been proven to be involved in regulating diapause in many insects, including the cotton bollworm, *Sericinus montelus* and *Sitodiplosis mosellana* [51, 59, 60]. Trehalose is one of the important cold-resistant substances in *A. segetum* [45].

The environmental association analysis identified candidate genes associated with latitude and temperature. Insect populations at high latitude need to adapt to low-temperature environments [61]. The latitude association analysis also enriched genes related to unsaturated fatty acid synthesis and sucrose metabolism, further confirming the role of glycolipid metabolism in the resistance of *A. segetum* to low temperatures. Fewer genes were associated with the environmental association analysis using SVs, while most of these genes could also be associated with SNPs. Both SNPs and SVs are major sources of genomic variation and participate in the evolution and adaptation of species [62], SVs have greater influence on gene expression and phenotype [63]. However, it is undeniable that there are certain false positives in SVs identified by short-read sequencing [64, 65], and thus such data still need to be supplemented by long-read sequencing data.

## Conclusions

Our research results revealed the genetic distribution of *A. segetum* in China from the population genomics level, explained the multi-host and pesticide tolerance of this polyphagous insect, and analyzed the adaptation of *A. segetum* to local environments from the perspectives of selection and association analyses. Our research not only provides a genetic basis for the adaptation of this agricultural pest, but also increases our understanding of the local adaptability of agricultural pests.

## Methods

### Sampling and sequencing

A total of 98 wild *A. segetum* samples were collected from four major crop growing regions in North China, Northeast China, Xinjiang, and South China for resequencing (Additional file 3: Table S5). Samples were stored at −20℃ before DNA extraction. Genomic DNA was extracted from each individual using the PureLink Genomic DNA Mini Kit. DNA concentration was measured by NanoDrop and DNA integrity was assessed by agarose gel electrophoresis. The DNA samples were then sent to BGI, Shenzhen, China, for DNB (DNA Nanoball) sequencing.

### SNP and SV calling for population accessions

Raw reads were trimmed to obtain clean reads using Trimmomatic v0.39 [66]. Clean reads then were mapped to the reference genome of *A. segetum* by the BWA-MEM algorithm of BWA v0.7.17 [67] with default parameters. GATK v4.2.3.0 [68] was used to sort the alignment results and remove PCR duplicate reads. Sequence mapping rate and depth were calculated using Samtools [69], individuals with low mapping rates were removed. The HaplotypeCaller command of GATK was used to identify SNPs for each individual and to generate single GVCF files that were merged into a VCF file by the CombineGVCFs command. Then we identified the variants by the GenotypeGVCFs command. SNPs were filtered using a custom script and then hard filtered using the VariantFiltration command of GATK. The filtration criterion was “QD < 2.0 || MQ < 40.0 || FS > 60.0 || SOR > 3.0 || MQRankSum < -12.5 || ReadPosRankSum < -8.0”. To further obtain high-quality SNPs, we used VCFTools v0.1.16 [70] to preserve Bi-allelic SNPs with missing data rate less than 20% and minor allele frequency (MAF) greater than 0.01. Based on the genome of *A. segetum*, we employed SnpEff v4.3t [71] for SNP annotation to classify SNPs into exons, introns, intergenic regions, and upstream or downstream regions. SV calling was performed using Delly v1.1.6 [72] twice for each individual. After combining all samples of SVs using BCFTools v1.13 [73], we retained SVs with “PASS” tag and length greater than 50 bp. The translocations were excluded because of the potential uncertainty from short reads [74]. We further filtered with a missing rate of 20% to verify the accuracy of SVs. SVs annotations were performed by the software program Annovar [75].

### Population structure

SNPs with MAF > 0.05 in the dataset were retained by VCFTools and filtered according to linkage disequilibrium (LD) for population structure analysis. In order to analyze the phylogenetic relationships, the VCF file containing the population variation information was converted into a PHY file by TASSEL v5 [76]. A maximum likelihood (ML) tree with *A*. *ipsilon* as the outgroup was constructed by IQ-TREE v2.1.4 [77]. The reliability of the model ML tree was estimated using the ultrafast bootstrap (UFboot) method with 1000 repeats, and the best-fit model PMB+F+R7 was used as the evolutionary mutation model to build the tree. We visualized the tree using Interactive Tree Of Life (iTOL) v6 [78]. The same dataset was employed for principal component analysis (PCA) using PLINK v1.90b6.24 based on the variance-standardized relationship matrix [79]. The first three eigenvectors were retained to create a plot in two dimensions by the R package ggplot2. We inferred the population structure by ADMIXTURE v1.3.0 [80], with the number of clusters (K) set from 1 to 10. The R package Pophelper was used to generate a stacked distribution bar diagram. The same phylogenetic analysis and other population analyses with SNP datasets were also conducted using SVs.

### Population diversity and gene flow

According to the clustering results, nucleotide diversity (*π*), Tajima’s D, and *F*_ST_ were calculated by VCFTools using a 20-kb sliding window. Then, we calculated the inter-population weighted *F*_ST_ values and average *π* values. We used LD-filtered SNPs with no missing values to build the tree and inferred patterns of historical splitting and admixture events among populations using TreeMix [81].

### Demographic history

An individual with high sequencing depth was selected from each of the four populations to estimate the demographic history of *A. segetum* using PSMC v0.6.5 (pairwise sequentially Markovian coalescent) [82] with a mutation rate of 3×10^−9^ and three generations per year. The parameters were set as follows: “-N25 -t15 -r5 -p 4+25*2+4+6”.

### Detection of selective sweeps

To detect potential signals of natural selection, we conducted the CLR analysis for each population using SweeD v4.0.0 [83] with a 10-kb window. Regions with the top 1% highest CLR values were considered as outlier regions, and genes overlapping the outlier regions were considered as candidate selection genes.

We used a combination of *F*_ST_ and *π* to detect the signals of selection between populations. *F*_ST_ and *π* between populations were calculated by VCFTools using a 20-kb sliding window with a step size of 5 kb. The top 5% common regions of *F*_ST_ value and the logarithmic ratio of *π* between two populations were defined as candidate outlier regions, and the genes overlapping the outlier regions were considered as candidate selection genes. We then estimated the haplotypes of the candidate genes. The SNPs were extracted according to the gene location and were expanded by beagle [84]. Heat maps were plotted according to the genotype files.

### Environmental association analysis

Based on the latitude and longitude information of all sample collection sites, we used the R package to extract the corresponding values of environmental factors from World Clim 2.0 (www.worldclim.org) using a spatial resolution of 5 min. Environmental factors that have important effects on insect environmental adaptation, such as latitude and longitude, annual mean temperature, and minimum temperature in the coldest month, were used as the main phenotypic data. We performed environmental association analysis using the mixed linear model (GEMMA) [85] and the factored spectrally transformed linear mixed model (FaST-LMM) [86]. We initially used imputed high-quality genotypes for GEMMA to identify candidate loci while controlling for population structure and inbreeding effects through the calculation of the kinship matrix. To reduce the error rate of multiple hypothesis testing, the *p*-values were corrected using the Benjamin-Hochberg correction (0.05/number of independently separated SNPs). Subsequently, we employed the same dataset for FaST-LMM to identify candidate loci and applied an FDR correction with a *q*-value of 1% to adjust the *p*-values and establish the significance cutoff. The upstream and downstream candidate intervals of significant SNPs were determined according to the LD decay distance. Only genes located at or near significant SNPs were considered candidate genes. KEGG enrichment analysis was performed for the associated candidate genes. We also performed an environmental association analysis using SVs.

### Supplementary Information

**Additional file 1:** Genome assembly and phylogenetic analysis of *Agrotis segetum*.

**Additional file 2:** **Fig. S1.** The length and percentage of repeat elements in the *A. segetum* genome. **Fig. S2. **The distribution of CDS length in the genome of *A. segetum*. **Fig. S3.** Venn plot of functional annotations for predicted proteins of *A. segetum*. **Fig. S4. **Phylogenetic relationship and orthological comparison of 13 insects. **Fig. S5.** GO enrichment and KEGG enrichment of expanded genes in *A. segetum.* **Fig. S6.** Density of different sizes for each SV type.** Fig. S7.** Population structure analysis (K=2-6) based on SNPs. **Fig. S8.** The maximum likelihood (ML) tree based on SVs. **Fig. S9.** Principal components analysis (PCA) based on SVs. **Fig. S10.** Population structure analysis (K=2-6) based on SVs. **Fig. S11.** Heatmap of genetic differentiation index (*F*_ST)_ between pairwise populations.** Fig. S12.** Gene migration as inferred by Treemix. **Fig. S13.** Analysis of historical effective population size of *A. segetum* by PSMC.** Fig. S14.** The composite likelihood ratio (CLR) scores and gene enrichment in the NTC population. **Fig. S15.** The CLR scores and gene enrichment in the NEC population. **Fig. S16.** The CLR scores and gene enrichment in the XJ population. **Fig. S17.** The CLR scores and gene enrichment in the STC population. **Fig. S18.** Selective sweep analysis and selected region between STC and NEC (XJ) populations. **Fig. S19.** The top 10 pathways of KEGG enrichment of latitude-associated genes using GEMMA. **Fig. S20.** Manhattan plots of environmental association analysis using GEMMA.** Fig. S21.** Manhattan plots of environmental association analysis using FaST-LMM. **Fig. S22. **Venn diagrams of common genes in environmental association analysis. **Fig. S23.** Manhattan plots of environmental association analysis based on SVs.

**Additional file 3:** **Table S1.** Statistics of *A. segetum* genome sequencing data. **Table S2.** Assembly statistics of the genome of *A. segetum*. **Table S3.** BUSCO assessment of *A. segetum* genome assembly. **Table S4.** Sampling information of *A. segetum* collected in different areas. **Table S5.** Summary of the resequencing data of *A. segetum*. **Table S6.** Summary of the SNPs annotation. **Table S7.** Length distribution of SVs in different categories. **Table S8.** Genes of NTC selected region genes identified by CLR analysis. **Table S9.** Genes of NEC selected region genes identified by CLR analysis.** Table S10.** Genes of XJ selected region genes identified by CLR analysis. **Table S11.** Genes of STC selected region genes identified by CLR analysis. **Table S12.** Genes of NTC selected region genes identified by *F*_ST_ and π between XJ and NTC. **Table S13.** Genes of XJ selected region identified by *F*_ST_ and π between XJ and NTC. **Table S14.** Function and mutation types of four genes in the selected region. **Table S15.** Genes of NEC selected region identified by *F*_ST_ and π between STC and NEC. **Table S16.** Genes of STC selected region identified by* F*_ST_ and π between STC and NEC. **Table S17.** Genes of XJ selected region identified by *F*_ST_ and π between STC and XJ. **Table S18.** Genes of STC selected region identified by *F*_ST_ and π between STC and XJ. **Table S19.** Genes of NEC selected region identified by* F*_ST_ and π between XJ and NEC. **Table S20.** Genes of XJ selected region identified by* F*_ST_ and π between XJ and NEC. **Table S21.** Genes of NTC selected region identified by *F*_ST_ and π between STC and NTC. **Table S22.** Genes of STC selected region identified by *F*_ST_ and π between STC and NTC. **Table S23. **Genes of NEC selected region identified by *F*_ST_ and π between NTC and NEC. **Table S24.** Genes of NTC selected region identified by *F*_ST_ and π between NTC and NEC. **Table S25.** Regional and environmental data for environmental correlation analysis. **Table S26.** Strong associated genes in SNPs-environment association analysis using GEMMA. **Table S27.** Strong associated genes in SVs-environment association analysis using GEMMA.

## Data Availability

All data generated or analyzed during this study are included in this published article and its supplementary information files. The Genome and Transcriptome sequencing reads have been deposited at NCBI under the accession no. BioProject PRJNA595759 [87]. The genome assembly has been deposited at GenBank under accession JAQSVV000000000 [88]. All of the raw short-read sequencing data used for population analysis have been deposited at NCBI as BioProject PRJNA933099 [89]. The custom codes are available on GitHub (https://github.com/xiao-xiaoping/Population_genomics_pipline) [90].

## Abbreviations

KEGG: Kyoto Encyclopedia of Genes and Genomes
NTC: North China
NEC: Northeast China
XJ: Xinjiang
STC: South China
MAF: Minor allele frequency
LD: Linkage disequilibrium
DEL: Deletion
DUP: Duplication
INS: Insertion
INV: Inversions
ML: Maximum likelihood
PCA: Principal component analysis
*F*_ST_: Genetic differentiation index
π: Nucleotide diversity
P450: Cytochrome P450
GR: Taste receptor
GP: Glycogen phosphatase
TPS: Trehalose synthase
RBFOX1: RNA-binding protein fox-1
PK1-R: Pyrokinin-1 receptor
NURF: Nucleosome remodeling factor subunit
CCDC: Coiled-coil domain-containing protein AGAP005037
PSMC: Pairwise sequentially Markovian coalescent
LG: Last glaciation
GPAT: Glycerol-3-phosphate O-acyltransferase
Treh-2: Trehalase-2
FAS: Fatty acid synthase
PER: Period circadian protein
TH: Tyrosine 3-monooxygenase
OBPs: Odorant-binding protein
FOXO3: Forkhead box protein O3
FAD: Desaturase
ABCC1: Multidrug resistance-associated protein 1
NFAT5: Nuclear factor of activated T-cells 5
ORP: Oxysterol-binding protein-related protein
POD: Peroxidase
ErGPCR: Ecdysone-responsive G-protein coupled protein
CLR: Composite likelihood ratio
AMT: Annual mean temperature
MTCQ: Minimum temperature in the coldest quarter
UFboot: Ultrafast bootstrap
COL4A1: Collagen alpha-1 (IV) chain
TBRG1: Transformation growth factor regulator 1
