# Supplementary material for: Population genomics of Agrotis segetum provide insights into the local adaptive evolution of agricultural pests
Source: BMC Biol. 2024 Feb 20;22:42. doi: 10.1186/s12915-024-01844-x (PMC10877822; doi:10.1186/s12915-024-01844-x)
Supplement: Supplementary file 1 — Additional file 1: Genome assembly and phylogenetic analysis of Agrotis segetum. [file 12915_2024_1844_MOESM1_ESM.docx]

**Additional file 1.**

**Genome assembly and phylogenetic analysis**

A total of 35.82 Gb of PacBio reads and 31.56 Gb of Illumina HiSeq paired-end reads were obtained (Additional file 3: Table S1). PacBio reads was assembled with Flye to generate the initial assembly. After removing redundant sequences and polishing, a 600 Mb size genome was obtained. The number of contigs was 712 and contig N50 was 2,534 kb (Additional file 3: Table S2). In order to evaluate the accuracy and completeness of genome assembly, Illumina reads were mapping to the genome and the mapping rate was 98.55%. The BUSCO evaluation showed that 97.80% of the complete BUSCO genes were identified (Additional file 3: Table S3), so we got a high-quality genome of *A. segetum.* We found that the *A. segetum* genome had 251 Mb of repeats, accounting for about 41.87% of the whole genome (Additional file 2: Fig. S1). Combined with three gene prediction methods, a total of 18,522 genes were predicted as reference gene sets, with an average gene length of 10,765bp. The length distribution of coding sequence (CDS) was similar to other insects, and the average CDS length was 1,418 bp (Additional file 2: Fig. S2). Functional annotation results showed that about 89.58% of the genes were annotated by the NR database (Additional file 2: Fig. S3).

We clustered the protein sequences of 13 insect species and summarized the gene-set types. A phylogenetic tree of *Drosophila melanogaster* was constructed using 543 single-copy orthologous genes, and the time of species divergence was further estimated (Additional file 2: Fig. S4). The results showed that *A. segetum* is closely related to *A. ipsilon*, diverging from a common ancestor about 9.49 million years ago. Compared with other insects, *A. segetum* had 1,791 gene families expanded and 2,964 gene families contracted. We conducted GO enrichment and KEGG enrichment of the expanded gene families (Additional file 2: Fig. S5), and found that they were enriched in metabolic pathways such as detoxification metabolism, glycolipid metabolism, and immune metabolism, which was speculated to be related to the characteristics of *A. segetum* itself.

**Genome de-novo assembly and annotation**

Wild *A. segetum* individuals were reared on an artificial diet for multiple generations under constant laboratory conditions (25 ± 1 ℃, 55 ± 5% RH, 14L:10D). Genomic DNA of *A. segetum* was sequenced using the PacBio SMRT and Illumina Hiseq 2500 platform. The Illumina raw reads were filtered by trimming the adapter sequence and low-quality sequences to generate clean reads. Flye v2.9 [73] was used for genome assembly of PacBio reads to obtain a draft genome assembly. The PacBio raw reads were aligned with genome using Minimap v2 [74] and polished by Racon v1.4.20 [75] for three rounds. Then, we aligned the Illumina clean reads with the genome by BWA v0.7.17 [49] and then polished by Pilon v1.23 [76]. BUSCO v4.1.2 [77] assessed the completeness of the genome assembly by searching against insecta_odb10 data sets. We mapped the Illumina clean reads with the genome to obtain the mapping rate, the sequencing depth and coverage of the genome to evaluate the assembly quality.

RepeatModeler v1.0.11 (<http://www.repeatmasker.org/RepeatModeler.html>) constructed a de novo repeat library for ab initio prediction and RepeatMasker v4.0.7 (<http://www.repeatmasker.org/RepeatMasker/>) was used to identify the repeat sequences by comparing with the RepBase database. Combined with the above results, we identified the repetitive sequence of the *A. segetum* genome. Based on histolike transcriptome reads obtained by Illumina sequencing, we assembled transcripts using TopHat v2.1.1 [78] and Cufflink v2.2.1 [79], and then predicted gene structures by TransDecode v5.5.0 (<https://github.com/TransDecoder/TransDecoder>). For homology-based prediction, we mapped the protein sequences of *Bombyx mori*, *Helicoverpa armigera* and *Spodoptera litura* respectively to the *A. segetum* genome, and predicted by GeneWise v2 [80]. We employed Augustus v3.0.3 [81] for ab initio prediction through model training. Finally, three gene prediction results were integrated by Evidence Modeler v1.1.1 [82] to generate a reference gene set. The predicted protein sequences were aligned to eggNOG, NR, UniProt, and KEGG databases by Diamond v0.9.24 [83] to obtain gene functional annotation.

**Comparative genomics analysis**

Protein sequences from 13 insect species were used for comparative genomic analysis, including 12 lepidopteran insects: *Spodoptera litura* (Slit), *Spodoptera frugiperda* (Sfru), *Agrotis ipsilon* (Aips), *Helicoverpa armigera* (Harm), *Trichoplusia ni* (Trni), *Pieris rapae* (Prap), *Bombyx mori* (Bmor), *Plutella xylostella* (Pxyl), *Cydia pomonella* (Cpom), *Chilo suppressalis* (Csup), *Manduca sexta* (Msex), *Agrotis segetum* (Aseg) and a Diptera insects *Drosophila melanogaster* (Dmel) as outgroups. These protein sequences were downloaded from the database and the longest CDS transcripts were extracted for subsequent analysis. OrthoFinder v2.4.0 [84] identified Orthologues and homologues genes, in which diamond was used for multi-sequence alignment. We constructed a phylogenetic tree based on single-copy homologous genes. Protein sequences of single-copy homologous genes was used for multi-sequence alignment by MAFFT v7 [85]. Conserved sequences were then extracted by Gblocks v0.91b [86] and all single-copy sequences were merged. Subsequently, RAxML-NG v1.1 [87] was used to infer the phylogenetic tree based on the optimal amino acid replacement model "LG+I+G+F" estimated by Prottest v3.4 [88]. We made use of PAML package mcmctree 4.9 [89] to estimate divergence time among these insects, the time correction points were taken from TimeTree. Then the phylogenetic tree was visualized using Figtree v1.4.4 (<http://tree.bio.ed.ac.uk/software/figtree/>). CAFE v4.2 [90] conducted gene family expansion and contraction analysis based on OrthoFinder. GO and KEGG were further enriched in the extended gene family.

**References cited in supplementary material**

73. Kolmogorov M, Yuan J, Lin Y, Pevzner PA. Assembly of long, error-prone reads using repeat graphs. Nat Biotechnol. 2019;37(5):540-6.

74. Li H. Minimap2: Pairwise alignment for nucleotide sequences. Bioinformatics. 2018;34(18):3094-100.

75. Vaser R, Sovic I, Nagarajan N, Sikic M. Fast and accurate de novo genome assembly from long uncorrected reads. Genome Res. 2017;27(5):737-46.

76. Walker BJ, Abeel T, Shea T, Priest M, Earl AM. Pilon: An integrated tool for comprehensive microbial variant detection and genome assembly improvement. PLoS One. 2014;9(11):e112963.

77. Manni M, Berkeley MR, Seppey M, Simao FA, Zdobnov EM. Busco update: Novel and streamlined workflows along with broader and deeper phylogenetic coverage for scoring of eukaryotic, prokaryotic, and viral genomes. Mol Biol Evol. 2021;38(10):4647-54.

78. Kim D, Pertea G, Trapnell C, Pimentel H, Kelley R, Salzberg SL. Tophat2: Accurate alignment of transcriptomes in the presence of insertions, deletions and gene fusions. Genome Biol. 2013;14(4):R36.

79. Trapnell C, Williams BA, Pertea G, Mortazavi A, Kwan G, van Baren MJ, et al. Transcript assembly and quantification by rna-seq reveals unannotated transcripts and isoform switching during cell differentiation. Nat Biotechnol. 2010;28(5):511-5.

80. Birney E, Clamp M, Durbin R. Genewise and genomewise. Genome Res. 2004;14(5):988-95.

81. Stanke M, Diekhans M, Baertsch R, Haussler D. Using native and syntenically mapped cdna alignments to improve de novo gene finding. Bioinformatics. 2008;24(5):637-44.

82. Haas BJ, Salzberg SL, Zhu W, Pertea M, Allen JE, Orvis J, et al. Automated eukaryotic gene structure annotation using evidencemodeler and the program to assemble spliced alignments. Genome Biol. 2008;9(1):R7.

83. Buchfink B, Reuter K, Drost H-G. Sensitive protein alignments at tree-of-life scale using diamond. Nat Methods. 2021;18(4):366-8.

84. Emms DM, Kelly S. Orthofinder: Phylogenetic orthology inference for comparative genomics. Genome Biol. 2019;20(1):238.

85. Katoh K, Standley DM. Mafft multiple sequence alignment software version 7: Improvements in performance and usability. Mol Biol Evol. 2013;30(4):772-80.

86. Talavera G, Castresana J. Improvement of phylogenies after removing divergent and ambiguously aligned blocks from protein sequence alignments. Syst Biol. 2007;56(4):564-77.

87. Kozlov AM, Darriba D, Flouri T, Morel B, Stamatakis A. Raxml-ng: A fast, scalable and user-friendly tool for maximum likelihood phylogenetic inference. Bioinformatics. 2019;35(21):4453-5.

88. Darriba D, Taboada GL, Doallo R, Posada D. Prottest 3: Fast selection of best-fit models of protein evolution. Bioinformatics. 2011;27(8):1164-5.

89. Yang Z. Paml 4: Phylogenetic analysis by maximum likelihood. Mol Biol Evol. 2007;24(8):1586-91.

90. Han MV, Thomas GWC, Lugo-Martinez J, Hahn MW. Estimating gene gain and loss rates in the presence of error in genome assembly and annotation using cafe 3. Mol Biol Evol. 2013;30(8):1987-97.
