# Supplementary material for: Population genomics of Agrotis segetum provide insights into the local adaptive evolution of agricultural pests
Source: BMC Biol. 2024 Feb 20;22:42. doi: 10.1186/s12915-024-01844-x (PMC10877822; doi:10.1186/s12915-024-01844-x)
Supplement: Supplementary file 2 — Additional file 2: Fig. S1. The length and percentage of repeat elements in the A. segetum genome. Fig. S2. The distribution of CDS length in the genome of A. segetum. Fig. S3. Venn plot of functional annotations for predicted proteins of A. segetum. Fig. S4. Phylogenetic relationship and orthological comparison of 13 insects. Fig. S5. GO enrichment and KEGG enrichment of expanded genes in A. segetum. Fig. S6. Density of different sizes for each SV type. Fig. S7. Population structure analysis (K=2-6) based on SNPs. Fig. S8. The maximum likelihood (ML) tree based on SVs. Fig. S9. Principal components analysis (PCA) based on SVs. Fig. S10. Population structure analysis (K=2-6) based on SVs. Fig. S11. Heatmap of genetic differentiation index (FST) between pairwise populations. Fig. S12. Gene migration as inferred by Treemix. Fig. S13. Analysis of historical effective population size of A. segetum by PSMC. Fig. S14. The composite likelihood ratio (CLR) scores and gene enrichment in the NTC population. Fig. S15. The CLR scores and gene enrichment in the NEC population. Fig. S16. The CLR scores and gene enrichment in the XJ population. Fig. S17. The CLR scores and gene enrichment in the STC population. Fig. S18. Selective sweep analysis and selected region between STC and NEC (XJ) populations. Fig. S19. The top 10 pathways of KEGG enrichment of latitude-associated genes using GEMMA. Fig. S20. Manhattan plots of environmental association analysis using GEMMA. Fig. S21. Manhattan plots of environmental association analysis using FaST-LMM. Fig. S22. Venn diagrams of common genes in environmental association analysis. Fig. S23. Manhattan plots of environmental association analysis based on SVs. [file 12915_2024_1844_MOESM2_ESM.docx]

**Additional file 2:** **Figures S1-S23**


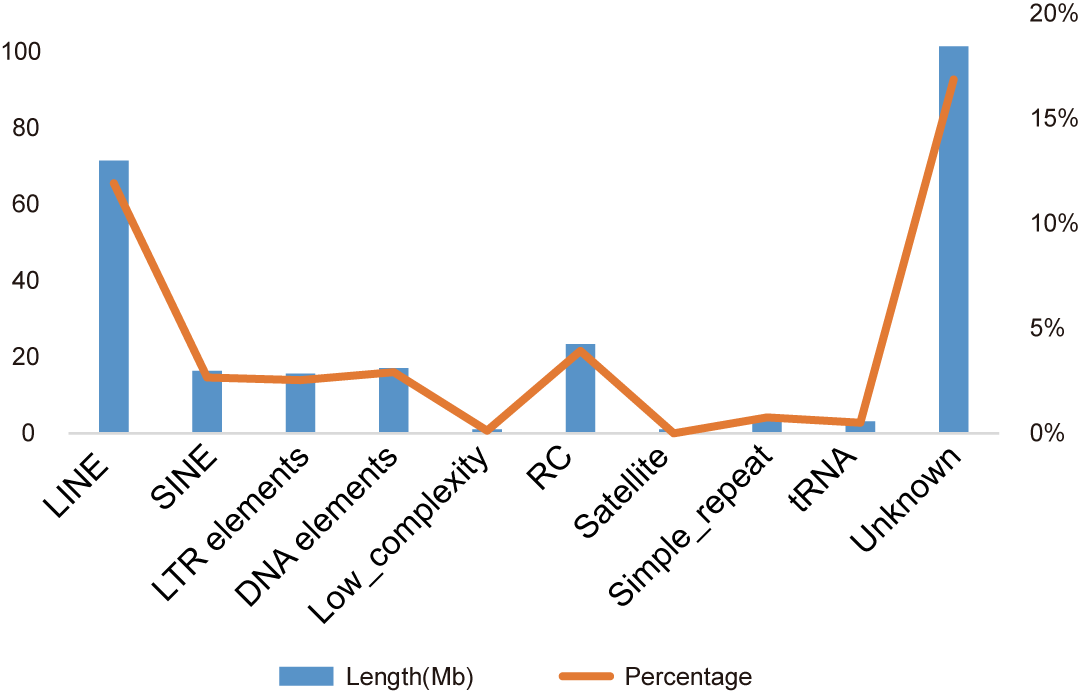


**Fig. S1.** The length and percentage of repeat elements in the *A.segetum* genome. Total repetitive sequences were 954 Mb (41.87%). The histogram shows the length of the repeat elements, and the line chart shows the percentage of repeat elements in genome.


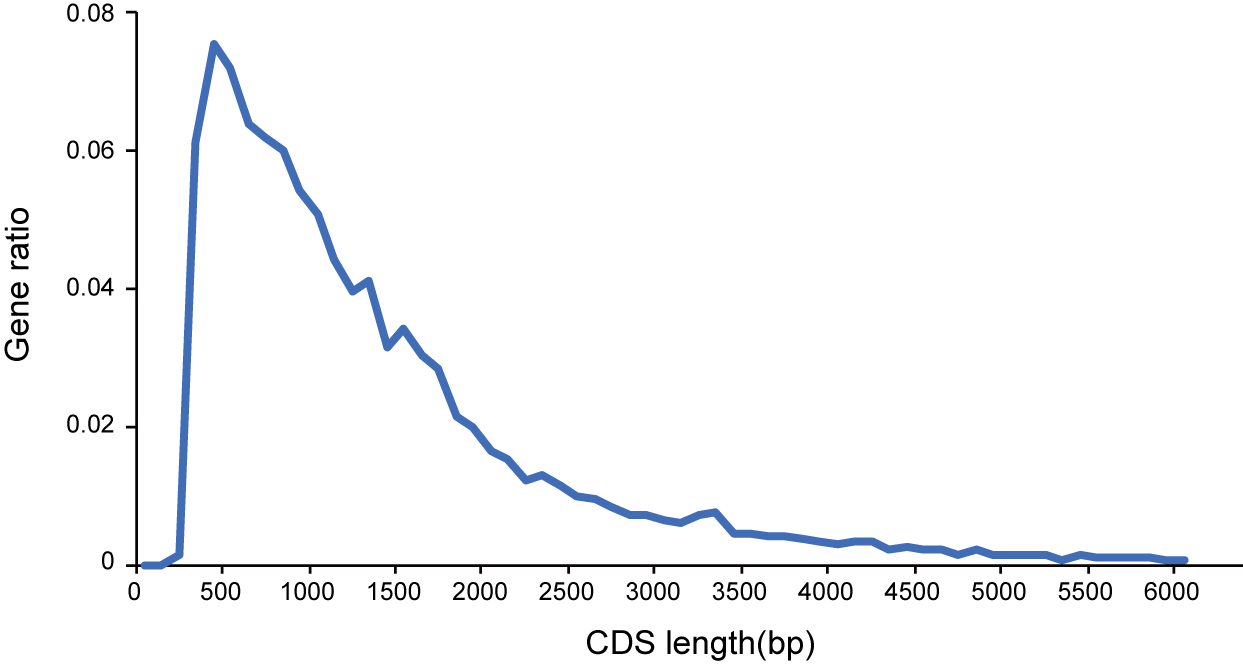


**Fig. S2.** The distribution of CDS length in the genome of *A.segetum* . x-axis: CDS length; y-axis: Gene ratio. The average CDS length is 1418 bp.


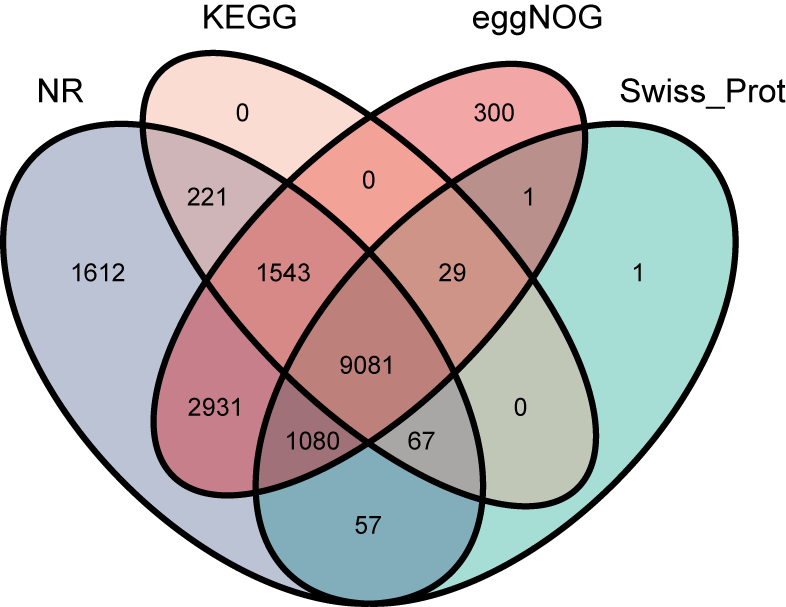


**Fig. S3.** Venn plot of functional annotations for predicted proteins of *A.segetum*. The number of genes predicted by NR (16,592), KEGG (10,941), eggNOG (14,965) and Swiss-Prot (10,316) databases. The intersection areas of different colors are predicted by at least two databases.


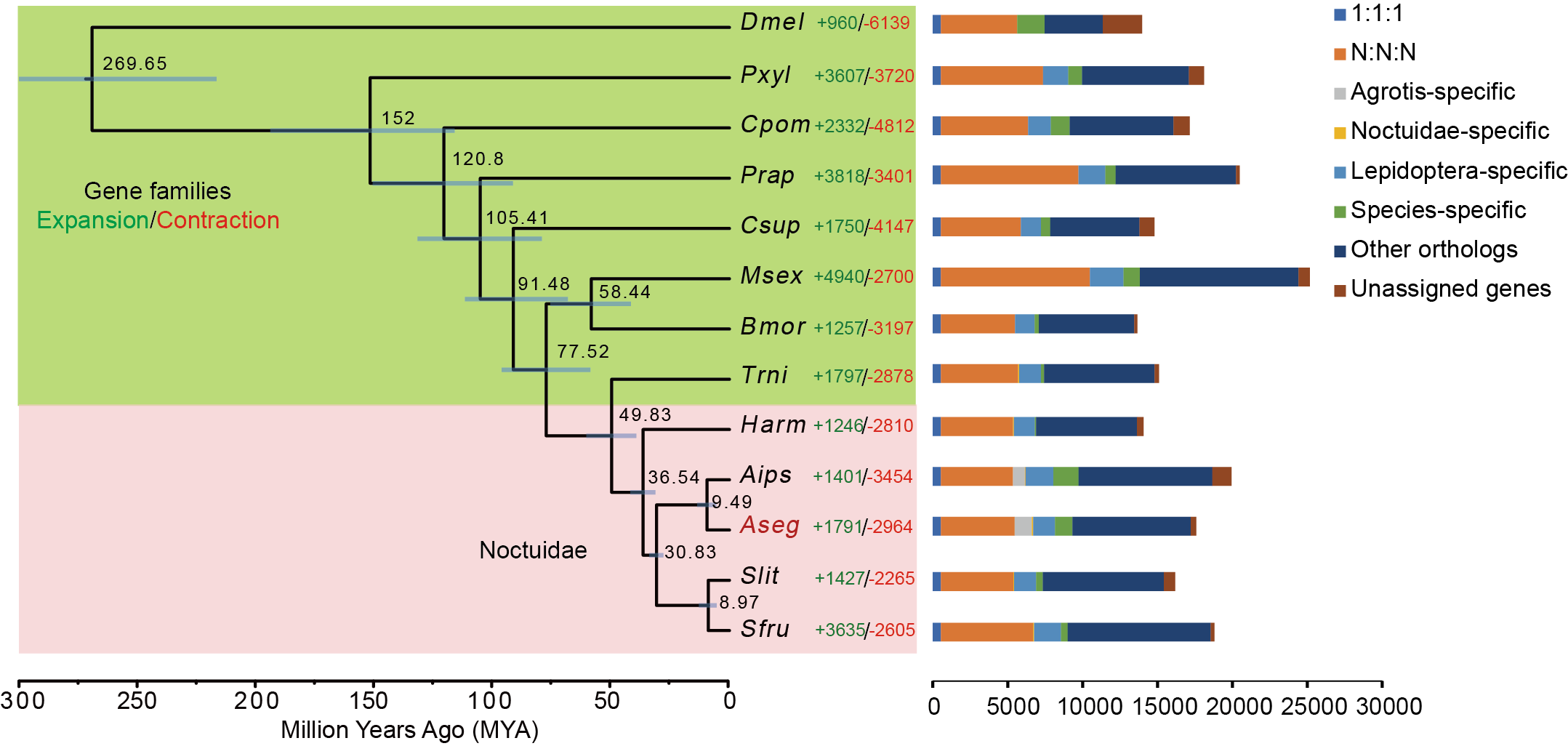


**Fig. S4.** Phylogenetic relationship and orthological comparison of 13 insects. Phylogenetic tree of Drosophila melanogaster as outgroup based on 543 single-copy orthologous genes. The location of *A. segetum* is highlighted in red. Estimated differentiation times (numbers) and 95% confidence intervals (blue bars) are shown at the nodes. The positive and negative numbers next to the species name are the number of expanded (green) / contracted (red) gene families derived from CAFE analysis. The histogram is a set of different types of genes for each species. 1:1:1, single copy genes; N:N:N, multicopy genes; Agrotis-specific, genes specific to the family of Agrotis; Noctuidae-specific, genes specific to the family of Noctuidae; lepidoptera-specific, genes specific to the family of lepidoptera; species-specific genes, genes that are unique to that species, not to other species.


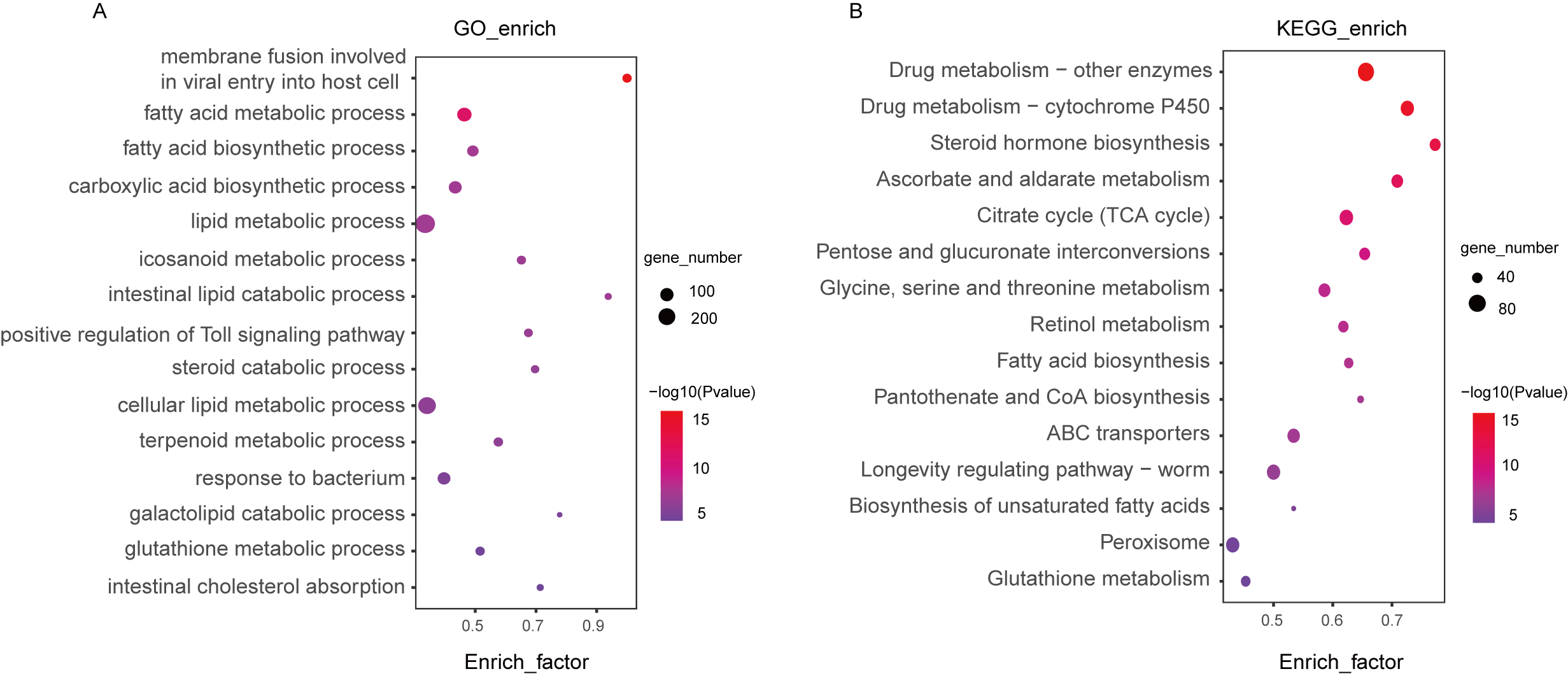


**Fig. S5.** GO enrichment and KEGG enrichment of expanded genes in *A.segetum*. X-axis: Enrich Facter; y-axis: pathway name; color of circle: *p*-value; size of circle: gene number.


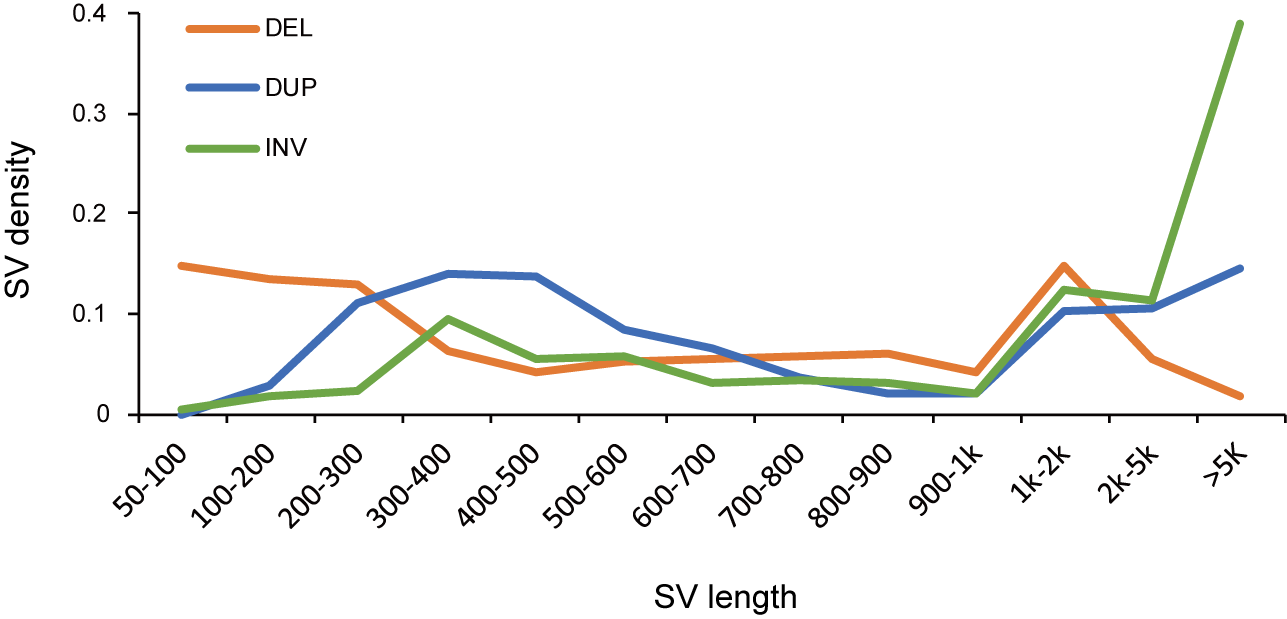


**Fig. S6.** Density of different sizes for each SV type. There were very few insertions and all of them are < 100 bp, so they were not shown in the figure.


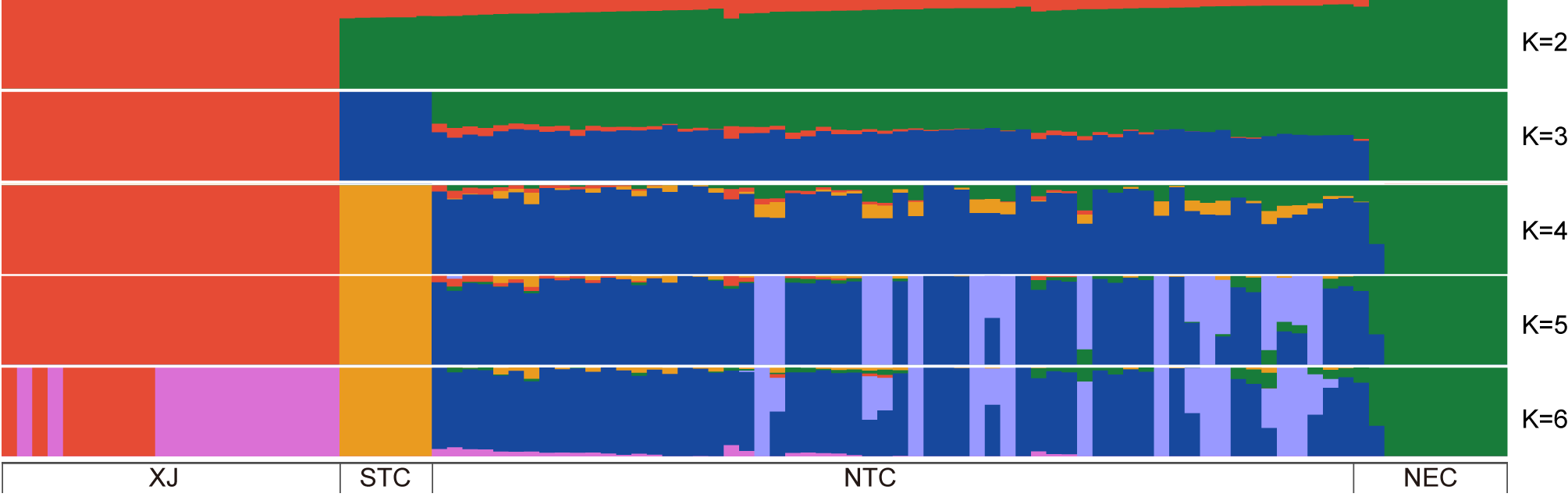


**Fig. S7.** Population structure analysis (K=2-6) based on SNPs. The color in each column indicates the proportion of individual in ancestral population.


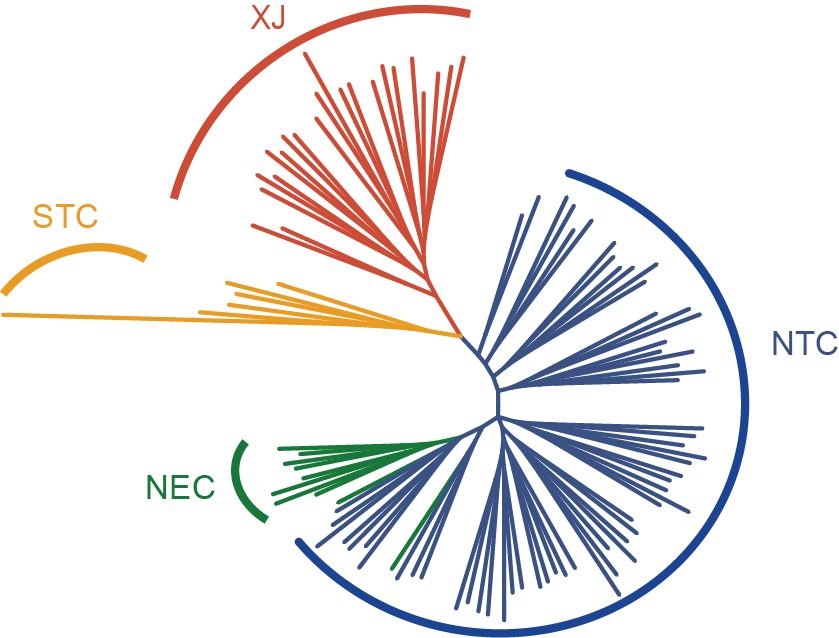


**Fig. S8.** The maximum likelihood (ML) tree based on SVs.


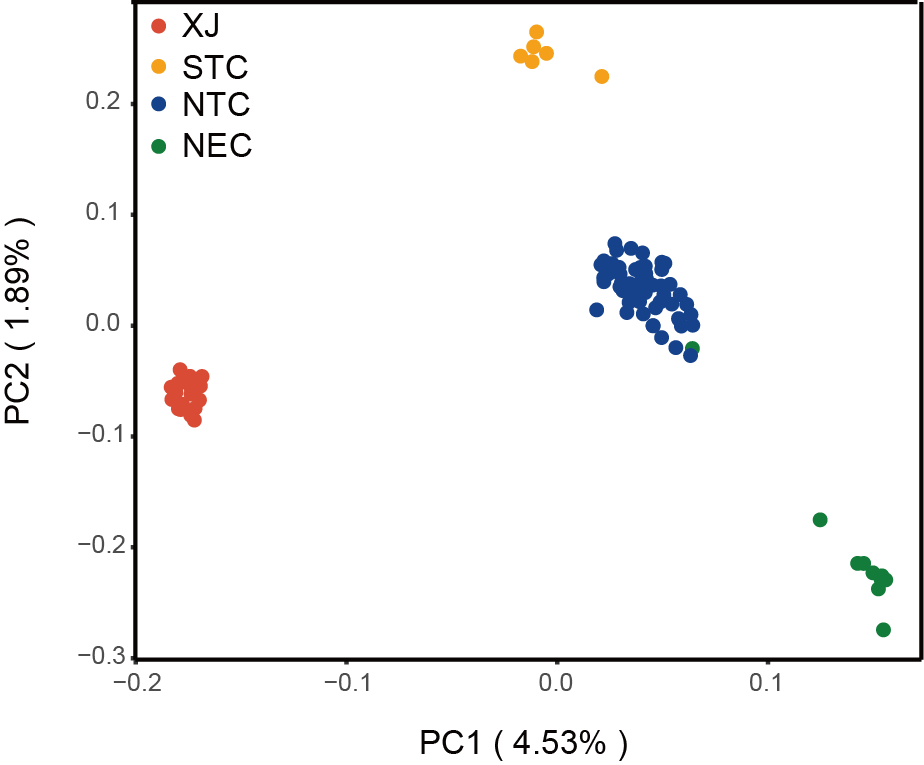


**Fig. S9**. Principal components analysis (PCA) based on SVs. Different colors represent different population.


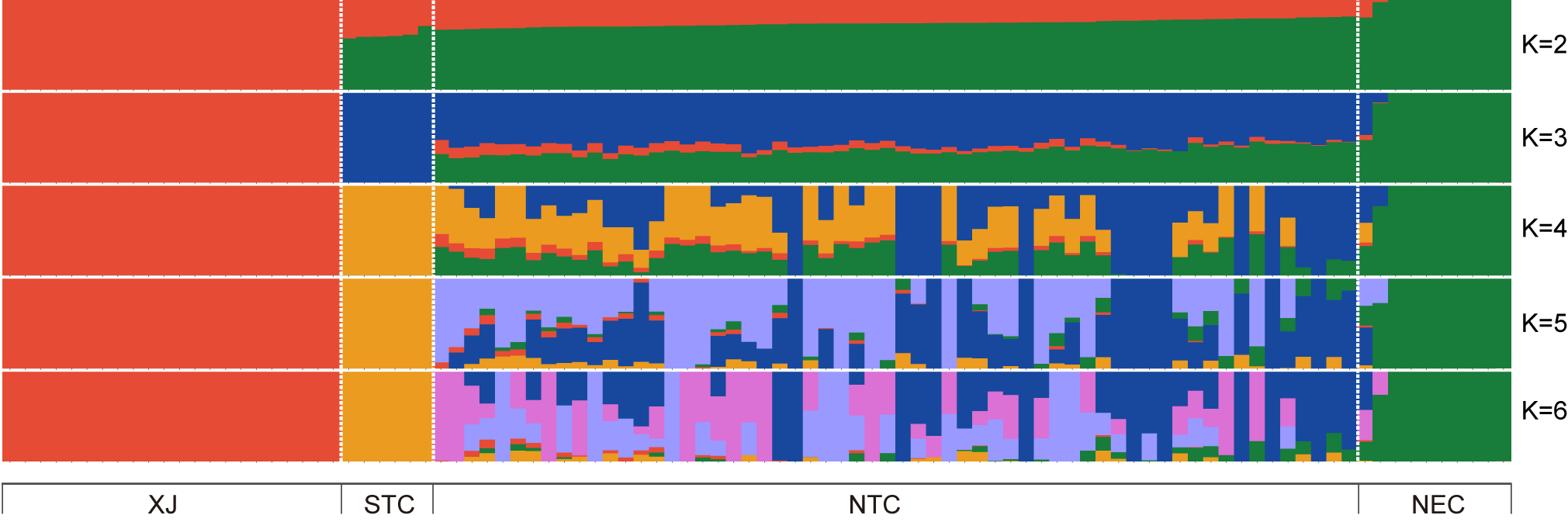


**Fig. S10.** Population structure analysis (K=2-6) based on SVs.


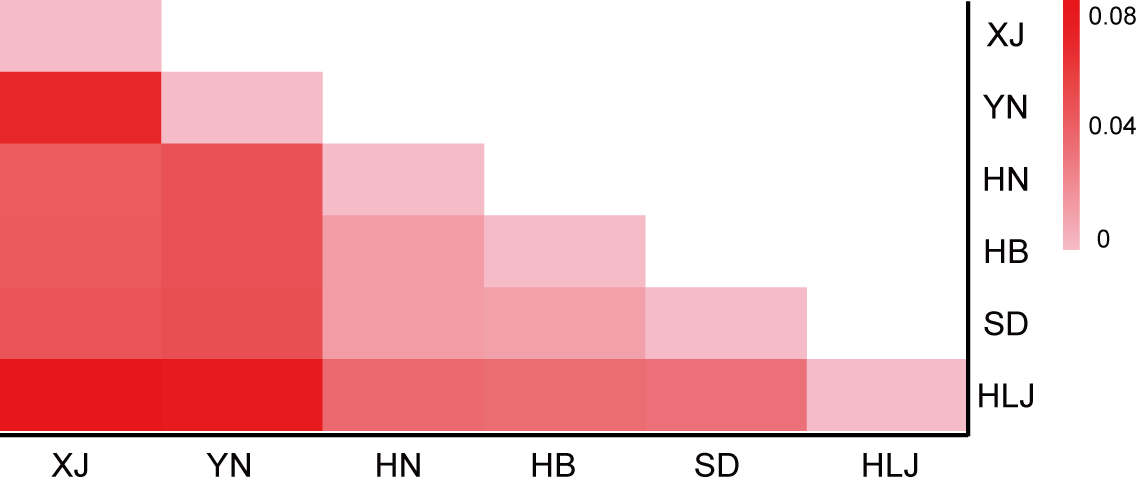


**Fig. S11.** Heatmap of genetic differentiation index (*F*_ST_) between pairwise populations. The module colors represent the *F*_ST_ value, which darkens as the color value increases.


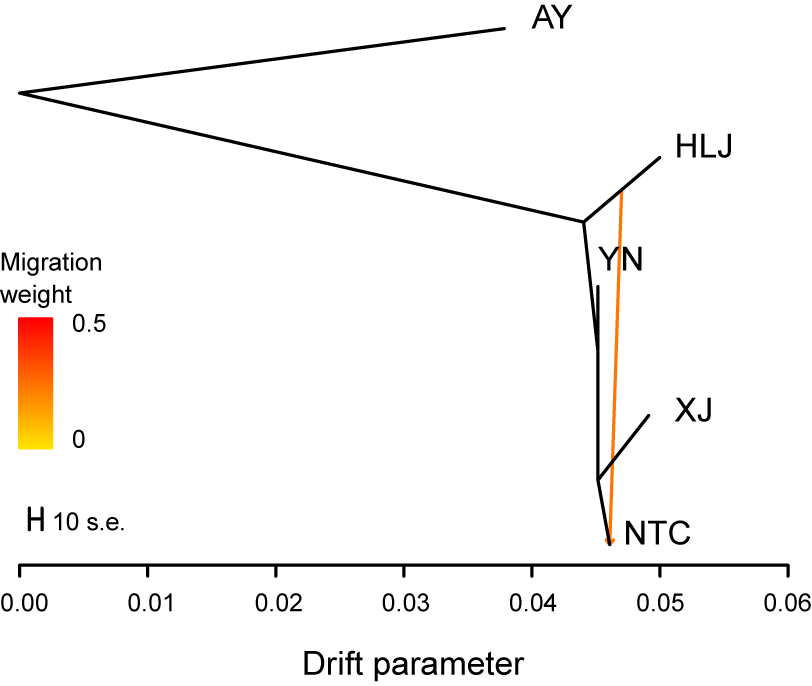


**Fig. S12.** Gene migration as inferred by Treemix. AY (*Agrotis ipsilon*) was the outgroup, and the arrow pointed to the direction of gene flow.


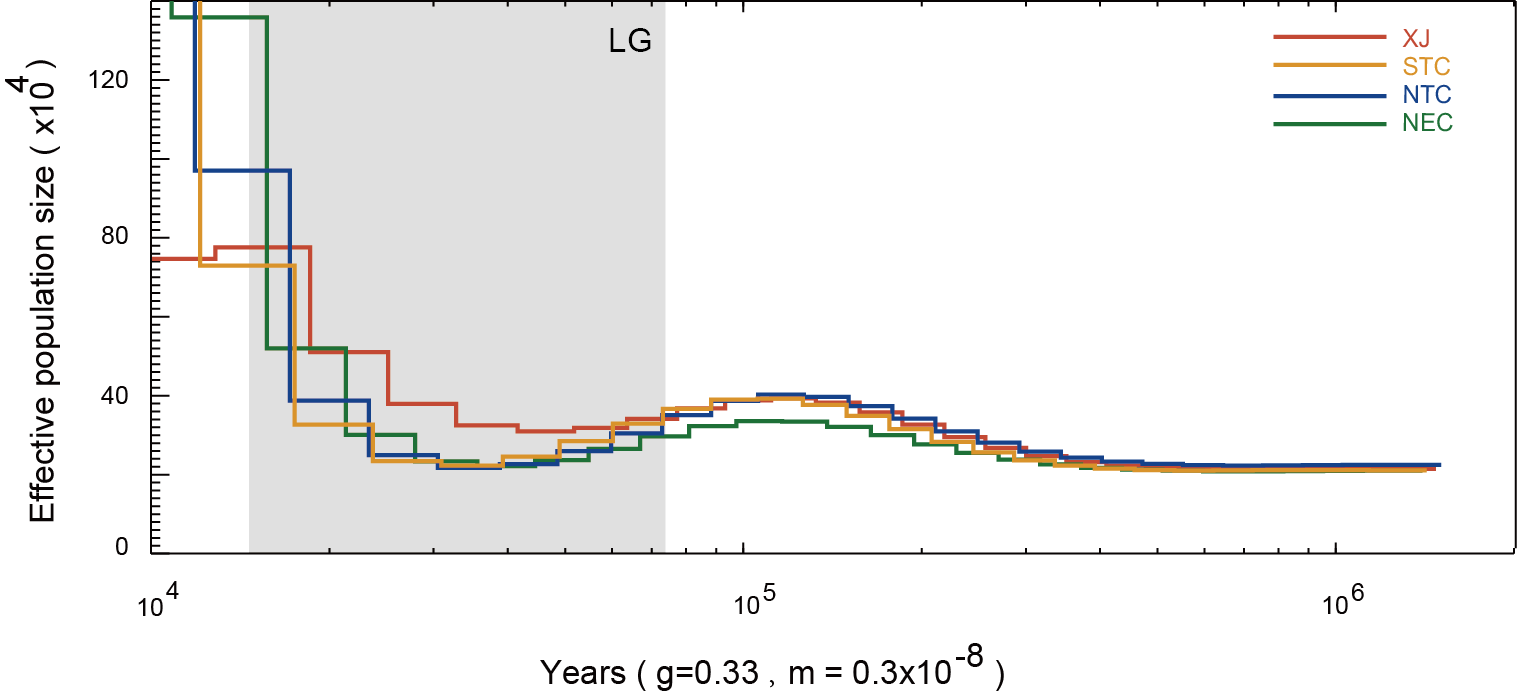


**Fig. S13.** Analysis of historical effective population size of *A. segetum* by PSMC. The colored lines represent different populations and the grey shade denotes the period of the last glacial (LG).


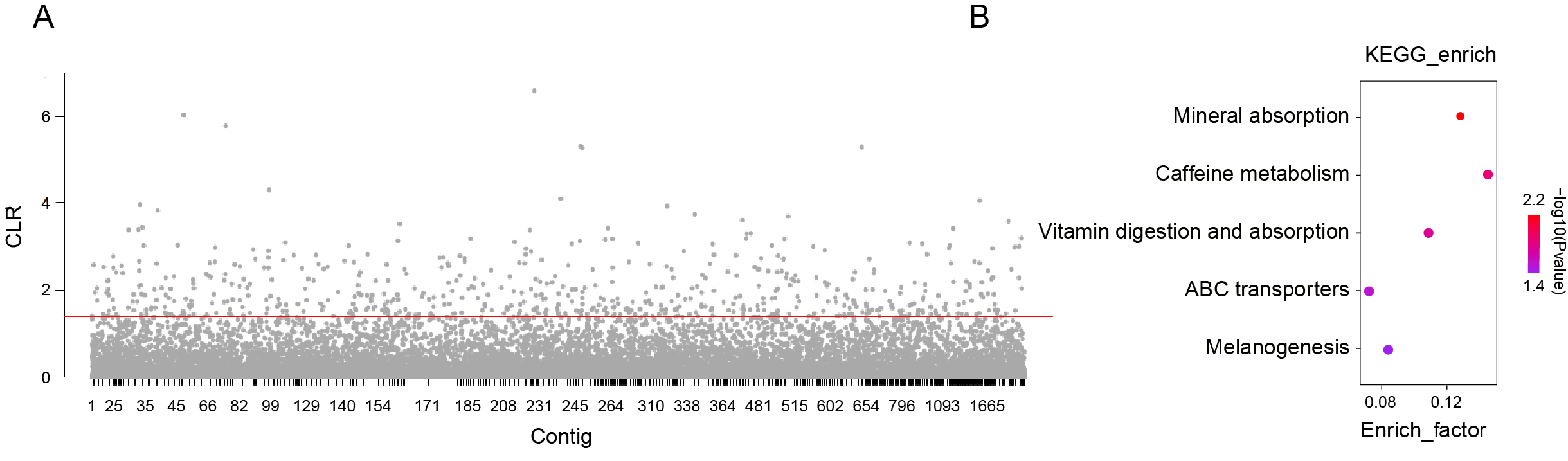


**Fig. S14.** The composite likelihood ratio (CLR) scores and gene enrichment in the NTC population. A. The CLR scores evaluated by SweeD in NTC population. The dashed lines mark the regions at the top 1%. B. The top 5 pathways of KEGG enrichment of selected genes.


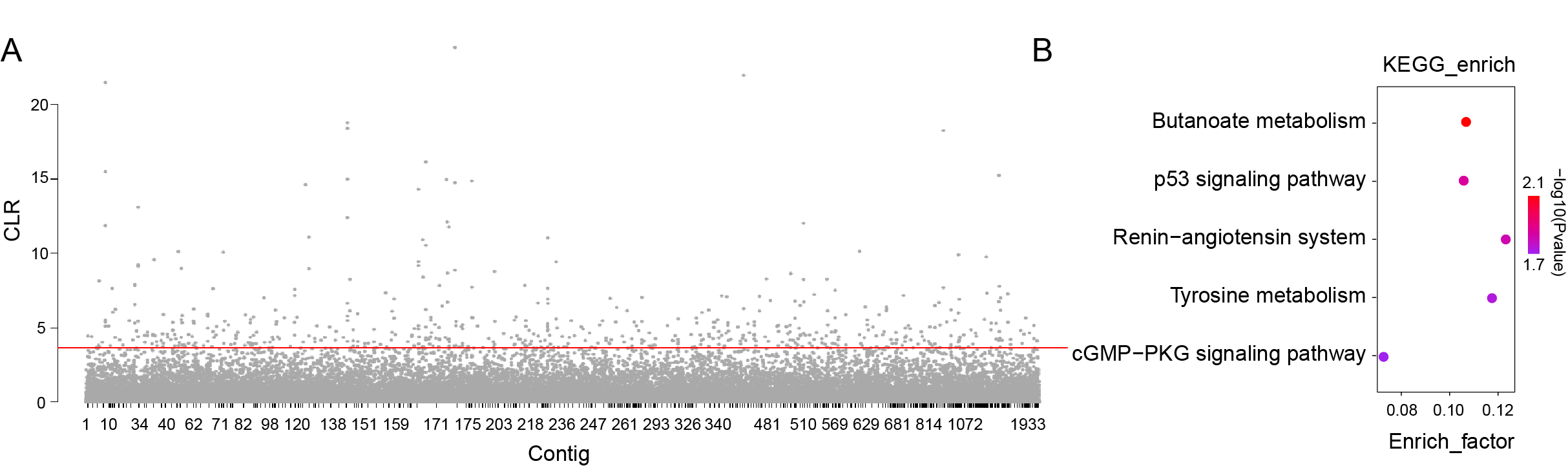


**Fig. S15.** The CLR scores and gene enrichment in the NEC population. A. The CLR scores evaluated by SweeD in NEC population. The dashed lines mark the regions at the top 1%. B. The top 5 pathways of KEGG enrichment of selected genes.


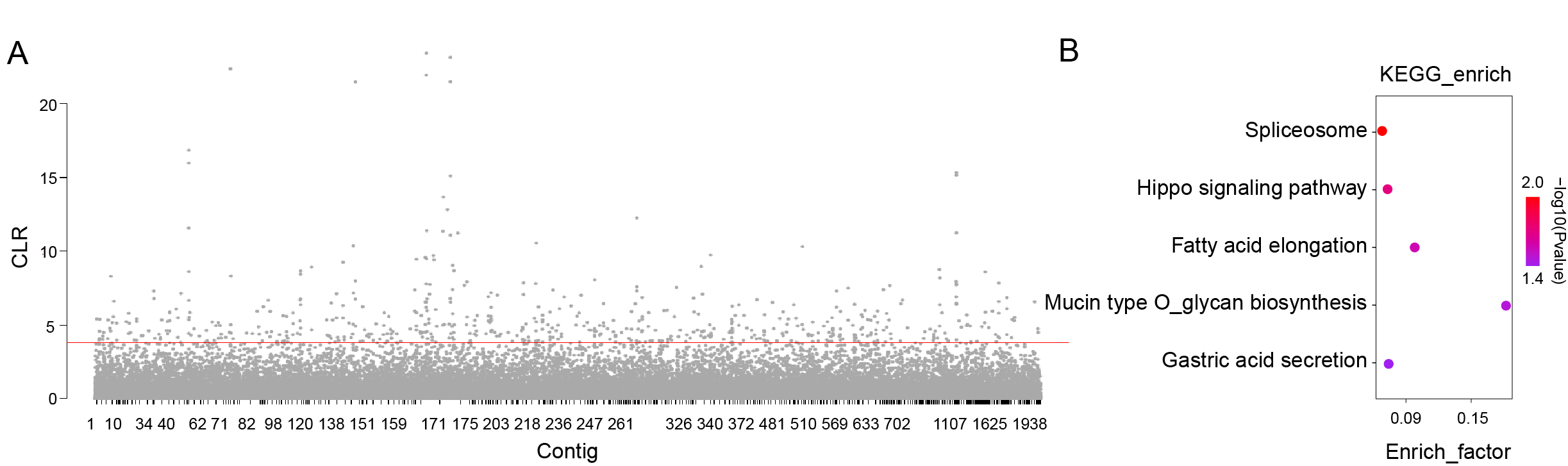


**Fig. S16.** The CLR scores and gene enrichment in the XJ population. A. The CLR scores evaluated by SweeD in XJ population. The dashed lines mark the regions at the top 1%. B. The top 5 pathways of KEGG enrichment of selected genes.


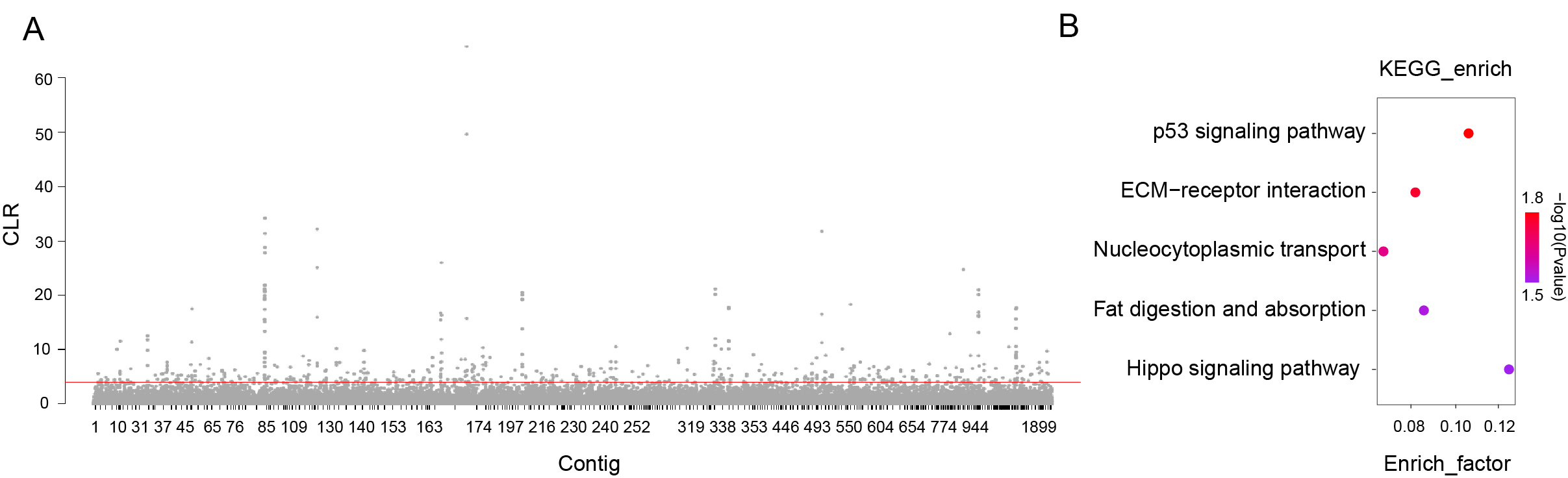


**Fig. S17.** The CLR scores and gene enrichment in the STC population. A. The CLR scores evaluated by SweeD in STC population. The dashed lines mark the regions at the top 1%. B. The top 5 pathways of KEGG enrichment of selected genes.


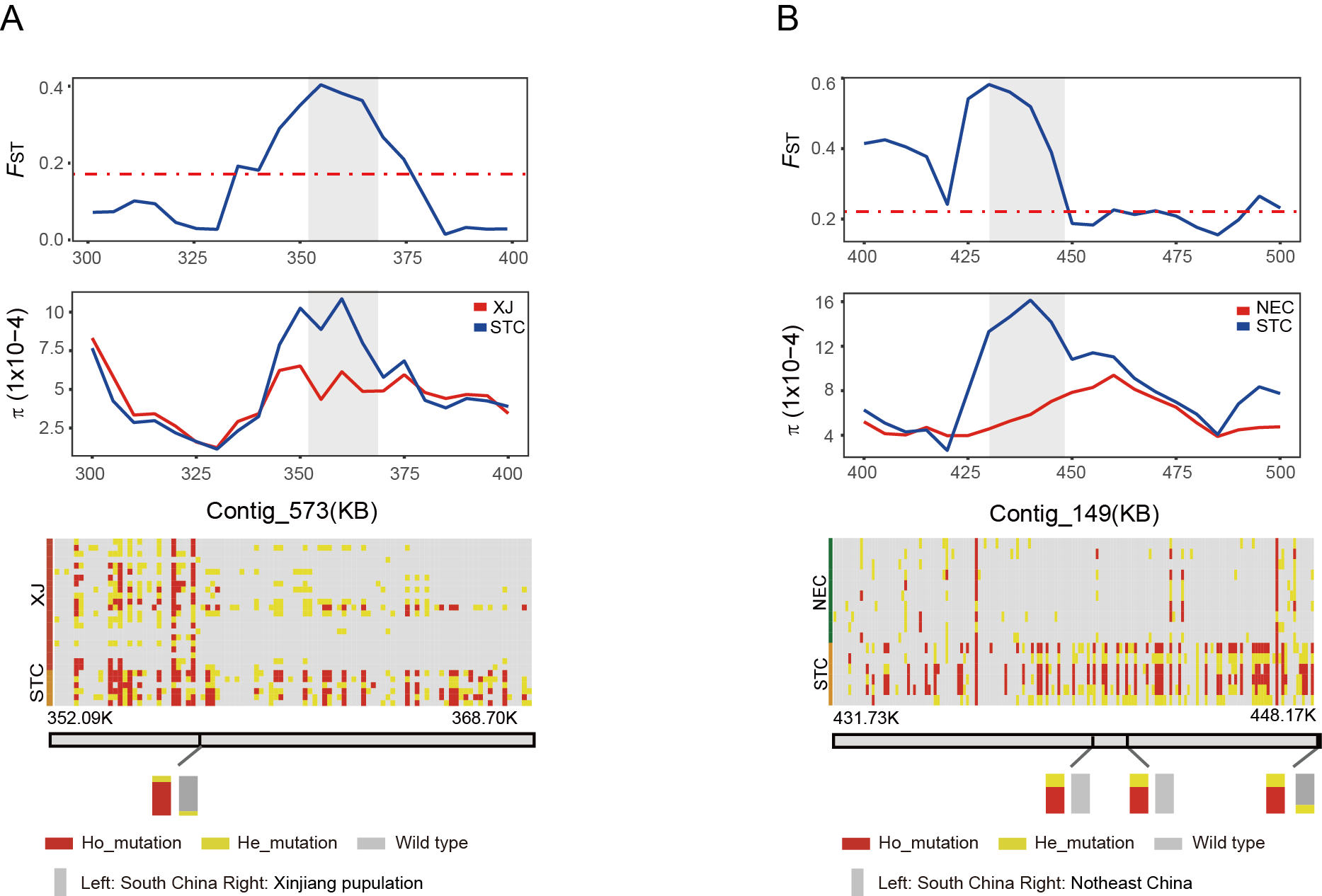


**Fig. S18.** Selective sweep analysis and selected region between STC and NEC (XJ) populations. A. *F*_ST_ and π value distribution and locus genotypes of *GP* of strong selective signal in STC and XJ population. The bar chart showed the frequency of missense mutant alleles, and the colors represented the types of alleles. B. *F*_ST_ and π value distribution and locus genotypes of *TPS* of strong selective signal in STC and XJ population.


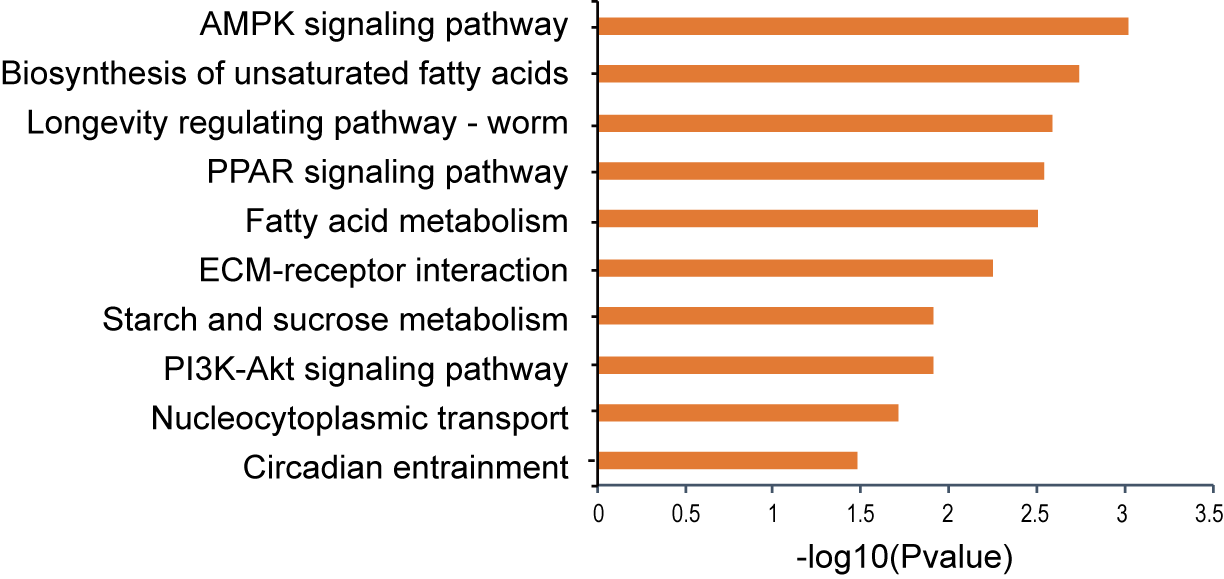


**Fig. S19.** The top 10 pathways of KEGG enrichment of latitude-associated genes using GEMMA.


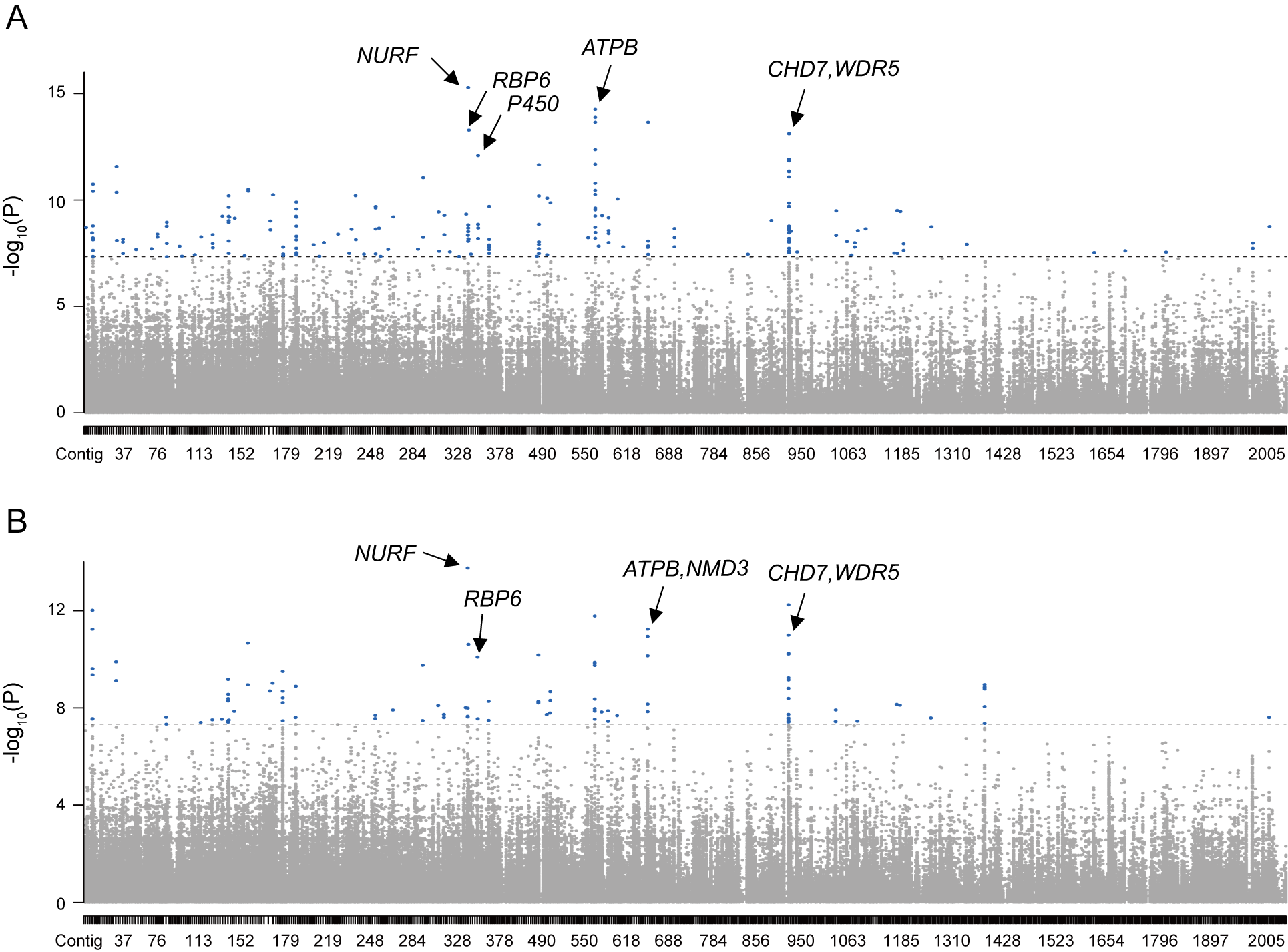


**Fig. S20.** Manhattan plots of environmental association analysis using GEMMA. The Manhattan plot of AMT (A) and MTCQ (B) based on SNPs. The blue dots are associated regions, and the labeled genes are strongly associated genes.


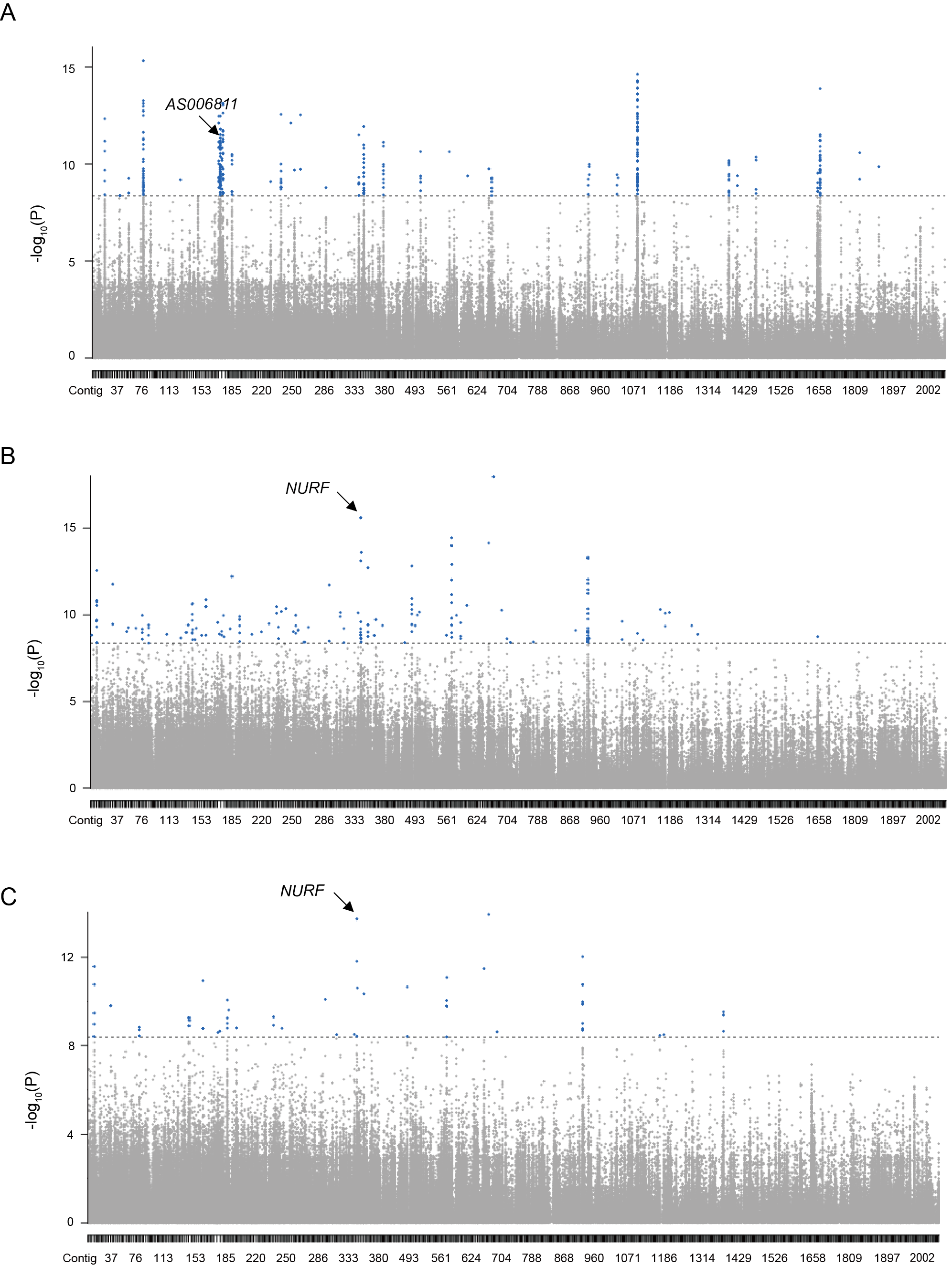


**Fig. S21.** Manhattan plots of environmental association analysis using FaST-LMM. The Manhattan plot of latitude (A), AMT (B) and MTCQ (C) based on SNPs. The blue dots are associated regions, and the labeled genes are strongly associated genes.


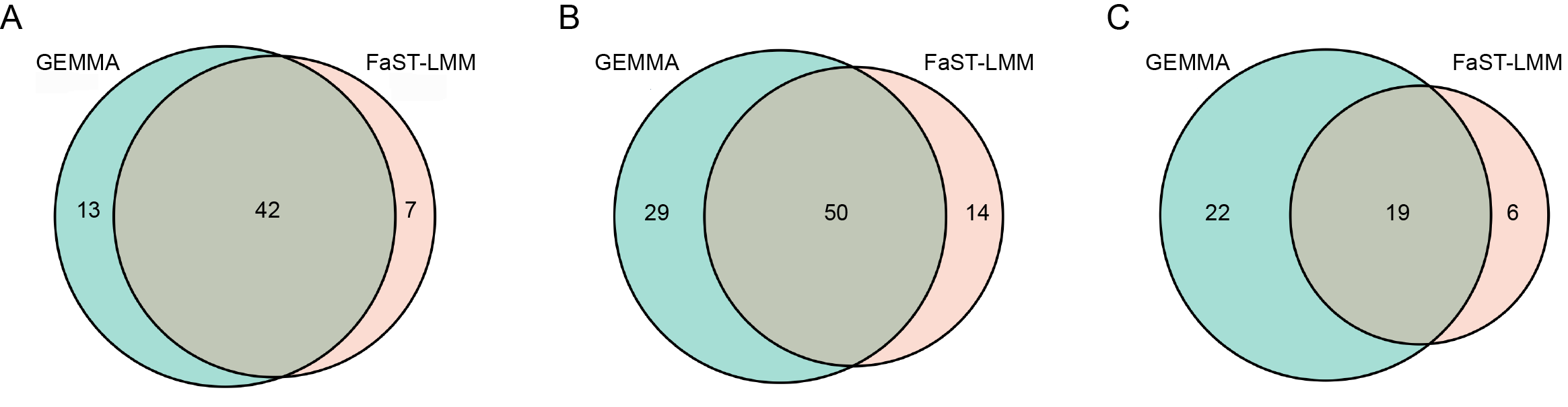


**Fig. S22.** Venn diagrams of common genes in environmental association analysis. A. The Venn diagram of latitude-associated genes. B. The Venn diagram of AMT-associated genes. C. The Venn diagram of MTCQ-associated genes.


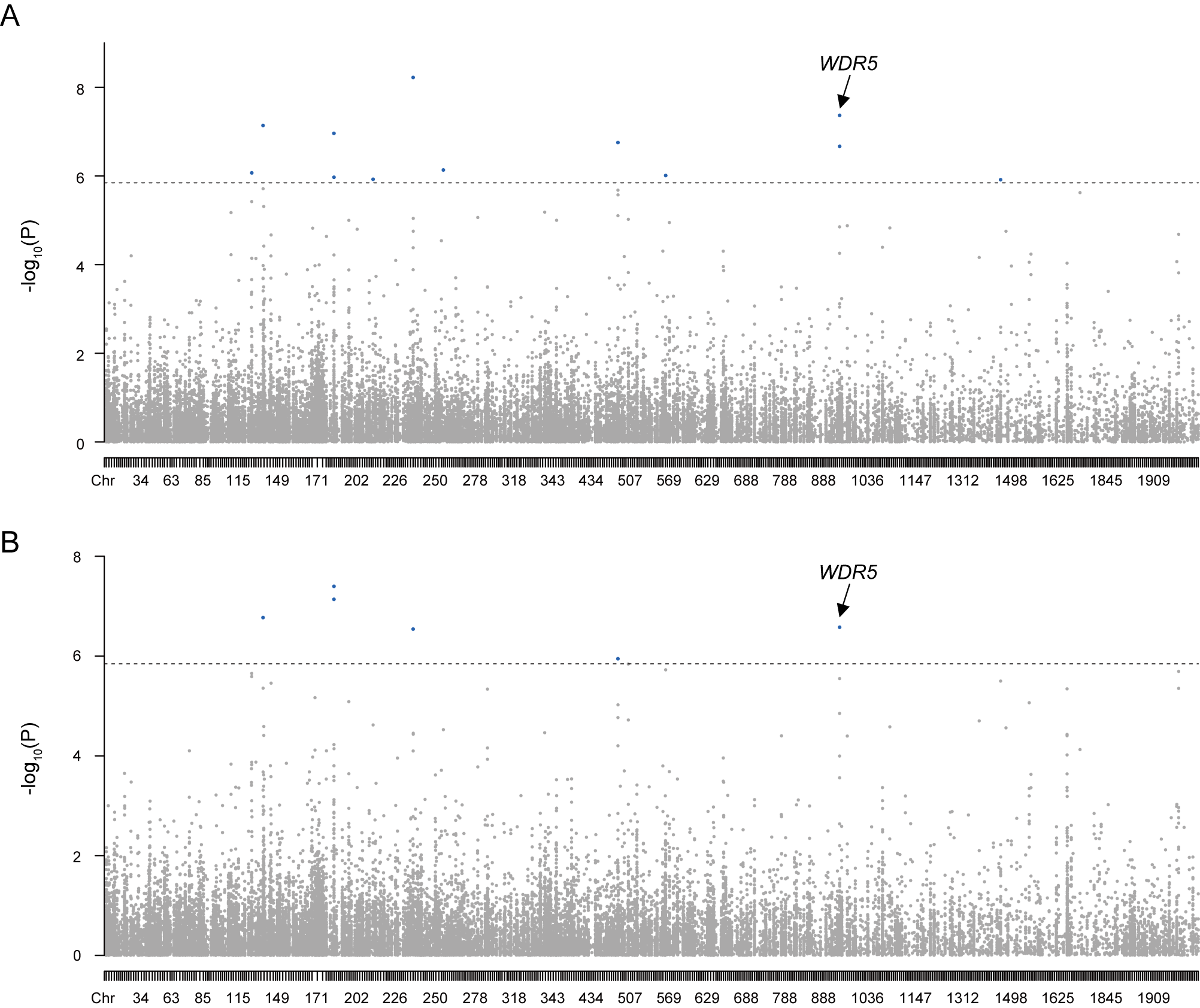


**Fig. S23.** Manhattan plots of environmental association analysis based on SVs. A. Manhattan plots of annual mean temperature based on SVs. B. Manhattan plots of min temperature of coldest month based on SVs. The blue dots are associated regions, and the labeled genes are strongly associated genes.
