# Supplementary material for: Population genomics of Agrotis segetum provide insights into the local adaptive evolution of agricultural pests
Source: BMC Biol. 2024 Feb 20;22:42. doi: 10.1186/s12915-024-01844-x (PMC10877822; doi:10.1186/s12915-024-01844-x)
Supplement: Supplementary file 3 — Additional file 3: Table S1. Statistics of A. segetum genome sequencing data. Table S2. Assembly statistics of the genome of A. segetum. Table S3. BUSCO assessment of A. segetum genome assembly. Table S4. Sampling information of A. segetum collected in different areas. Table S5. Summary of the resequencing data of A. segetum. Table S6. Summary of the SNPs annotation. Table S7. Length distribution of SVs in different categories. Table S8. Genes of NTC selected region genes identified by CLR analysis. Table S9. Genes of NEC selected region genes identified by CLR analysis. Table S10. Genes of XJ selected region genes identified by CLR analysis. Table S11. Genes of STC selected region genes identified by CLR analysis. Table S12. Genes of NTC selected region genes identified by FST and π between XJ and NTC. Table S13. Genes of XJ selected region identified by FST and π between XJ and NTC. Table S14. Function and mutation types of four genes in the selected region. Table S15. Genes of NEC selected region identified by FST and π between STC and NEC. Table S16. Genes of STC selected region identified by FST and π between STC and NEC. Table S17. Genes of XJ selected region identified by FST and π between STC and XJ. Table S18. Genes of STC selected region identified by FST and π between STC and XJ. Table S19. Genes of NEC selected region identified by FST and π between XJ and NEC. Table S20. Genes of XJ selected region identified by FST and π between XJ and NEC. Table S21. Genes of NTC selected region identified by FST and π between STC and NTC. Table S22. Genes of STC selected region identified by FST and π between STC and NTC. Table S23. Genes of NEC selected region identified by FST and π between NTC and NEC. Table S24. Genes of NTC selected region identified by FST and π between NTC and NEC. Table S25. Regional and environmental data for environmental correlation analysis. Table S26. Strong associated genes in SNPs-environment association analysis using GEMMA. Table S27 [file 12915_2024_1844_MOESM3_ESM.docx]

**Additional file 2:** **Table S1-S27**

**Table S1. Statistics of *A. segetum* genome sequencing data**

| **Pac Bio** | **Clean Reads** | **Clean Bases** |
| --- | --- | --- |
| 1 | 287,618 | 3,761,739,264 |
| 2 | 565,904 | 6,727,847,247 |
| 3 | 818,098 | 8,652,035,625 |
| 4 | 809,936 | 8,341,382,825 |
| 5 | 245,975 | 3,012,659,749 |
| Total | 3,291,492 | 35,824,387,786 |
| **Illumina** | **Clean Reads** | **Clean Bases** |
| - | 210,400,280 | 31,560,042,000 |
| **Illumina RNA-Seq data** | **Clean Reads** | **Clean Bases** |
| Larva | 49,363,280 | 14,808,984,000 |
| Pupae | 22,967,560 | 6,890,268,000 |
| Adult | 35,989,146 | 10,796,743,800 |

**Table S2 Assembly statistics of the genome of *A. segetum***

| **Category** | ***A.segetum*** |
| --- | --- |
| Assembled genome size (Mb) | 600 |
| Longest contig length (Kb) | 16,986 |
| Number of contigs | 712 |
| Contig N50 (Kb) | 2,534 |
| Contig N90 (Kb) | 576 |
| GC content (%) | 38.07 |
| Number of gene models | 18,522 |
| Mapping rate (%) | 98.55 |
| BUSCO complete gene ratio (%) | 97.80 |

**Table S3. BUSCO assessment of *A. segetum* genome assembly**

| **Term** | **Number** | **Rate** |
| --- | --- | --- |
| Complete BUSCOs (C) | 1336 | 97.70% |
| Complete and single-copy BUSCOs (S) | 1315 | 96.20% |
| Complete and duplicated BUSCOs (D) | 21 | 1.50% |
| Fragmented BUSCOs (F) | 9 | 0.70% |
| Missing BUSCOs (M) | 22 | 1.60% |
| Total BUSCO groups searched | 1367 |  |

**Table S4. Sampling information of *A. segetum* collected in different areas**

| **Region** | **Population ID** | **Location** | **Number** | **Data** |
| --- | --- | --- | --- | --- |
| Northwest China (NWC) | XJ | Korla,Xinjiang | 22 | 2020 |
| North China (NTC) | HN | Zhengzhou,Henan | 19 | 2020 |
|  | HB | Langfang,Hebei | 20 | 2020 |
|  | SD | Yantai,Shandong | 21 | 2020 |
| Northeast China (NEC) | HLJ | Herbin,Heilongjiang | 10 | 2020 |
| Southern China (STC) | YN | Xundian,Yunnan | 6 | 2022 |

**Table S5. Summary of the resequencing data of *A. segetum***

| **Population ID** | **Sample ID** | **Raw_Reads (M)** | **Raw_Base (G)** | **Clean_Reads (M)** | **Clean_Base (G)** | **Mapping_rate (**%**)** | **Depth** |
| --- | --- | --- | --- | --- | --- | --- | --- |
| XJ | ASXJ1 | 62.97 | 18.90 | 49.75 | 14.52 | 97.11 | 21.94 |
|  | ASXJ2 | 88.21 | 26.46 | 72.69 | 21.32 | 96.84 | 31.67 |
|  | ASXJ3 | 73.07 | 21.92 | 58.94 | 17.26 | 96.64 | 25.61 |
|  | ASXJ4 | 71.21 | 21.36 | 52.23 | 15.14 | 96.92 | 22.95 |
|  | ASXJ5 | 70.12 | 21.04 | 56.85 | 16.66 | 97.26 | 24.90 |
|  | ASXJ6 | 60.54 | 18.16 | 48.66 | 14.21 | 97.32 | 21.48 |
|  | ASXJ7 | 79.54 | 23.86 | 65.90 | 19.32 | 97.49 | 28.92 |
|  | ASXJ8 | 74.18 | 22.26 | 69.68 | 20.93 | 98.22 | 30.90 |
|  | ASXJ9 | 73.96 | 22.18 | 70.67 | 21.23 | 96.32 | 30.72 |
|  | ASXJ10 | 80.88 | 24.26 | 76.48 | 22.97 | 98.12 | 33.94 |
|  | ASXJ11 | 139.60 | 41.88 | 137.30 | 41.37 | 97.46 | 61.30 |
|  | ASXJ12 | 86.34 | 25.90 | 83.21 | 25.00 | 97.91 | 37.16 |
|  | ASXJ13 | 31.87 | 9.56 | 31.31 | 9.39 | 97.52 | 14.66 |
|  | ASXJ14 | 34.37 | 10.32 | 33.85 | 10.15 | 97.49 | 15.77 |
|  | ASXJ15 | 45.94 | 13.78 | 44.95 | 13.48 | 97.34 | 20.67 |
|  | ASXJ16 | 35.52 | 10.66 | 34.96 | 10.48 | 97.41 | 16.25 |
|  | ASXJ17 | 42.28 | 12.68 | 41.62 | 12.48 | 97.44 | 19.26 |
|  | ASXJ18 | 41.38 | 12.42 | 40.71 | 12.19 | 97.40 | 18.80 |
|  | ASXJ19 | 27.81 | 8.34 | 27.25 | 8.17 | 97.38 | 12.76 |
|  | ASXJ20 | 34.28 | 10.28 | 33.55 | 10.07 | 97.68 | 15.65 |
|  | ASXJ21 | 64.55 | 19.36 | 63.17 | 18.98 | 97.69 | 28.99 |
|  | ASXJ22 | 40.73 | 12.22 | 39.96 | 11.99 | 97.51 | 18.49 |
| HN | ASHN1 | 82.47 | 24.74 | 69.31 | 20.38 | 97.43 | 30.52 |
|  | ASHN2 | 76.49 | 22.94 | 61.90 | 18.12 | 97.38 | 27.20 |
|  | ASHN3 | 77.63 | 23.30 | 61.92 | 18.11 | 97.21 | 27.20 |
|  | ASHN4 | 58.97 | 17.70 | 45.69 | 13.30 | 97.03 | 20.16 |
|  | ASHN5 | 69.92 | 20.98 | 54.61 | 15.93 | 95.04 | 23.51 |
|  | ASHN6 | 73.55 | 22.06 | 58.50 | 17.11 | 92.43 | 23.77 |
|  | ASHN7 | 74.63 | 22.40 | 58.55 | 17.07 | 97.20 | 25.73 |
|  | ASHN8 | 46.40 | 13.92 | 36.31 | 10.57 | 97.01 | 16.20 |
|  | ASHN9 | 66.80 | 20.04 | 53.08 | 15.49 | 97.27 | 23.41 |
|  | ASHN10 | 36.69 | 11.00 | 35.93 | 10.80 | 97.38 | 16.59 |
|  | ASHN11 | 53.19 | 15.96 | 52.23 | 15.70 | 94.08 | 22.78 |
|  | ASHN12 | 50.83 | 15.24 | 49.87 | 14.99 | 97.38 | 22.83 |
|  | ASHN13 | 47.74 | 14.32 | 46.32 | 13.93 | 97.64 | 21.23 |
|  | ASHN14 | 32.75 | 9.82 | 32.14 | 9.66 | 97.21 | 14.91 |
|  | ASHN15 | 84.62 | 25.38 | 81.89 | 24.54 | 97.56 | 36.22 |
|  | ASHN16 | 37.81 | 11.34 | 37.12 | 11.16 | 97.47 | 17.17 |
|  | ASHN17 | 36.95 | 11.08 | 35.84 | 10.78 | 97.62 | 16.48 |
|  | ASHN18 | 46.90 | 14.06 | 46.08 | 13.86 | 97.44 | 21.13 |
|  | ASHN19 | 38.34 | 11.50 | 37.53 | 11.28 | 97.07 | 17.27 |
| HB | ASHB1 | 58.91 | 17.68 | 45.35 | 13.20 | 97.16 | 19.99 |
|  | ASHB2 | 65.14 | 19.54 | 52.08 | 15.23 | 97.45 | 22.93 |
|  | ASHB3 | 76.09 | 22.82 | 60.52 | 17.71 | 97.60 | 26.39 |
|  | ASHB4 | 78.50 | 23.54 | 59.37 | 17.29 | 96.35 | 25.60 |
|  | ASHB5 | 79.70 | 23.92 | 66.33 | 19.47 | 97.35 | 29.01 |
|  | ASHB6 | 82.28 | 24.68 | 66.92 | 19.59 | 97.58 | 29.24 |
|  | ASHB7 | 63.18 | 18.96 | 51.48 | 14.85 | 97.10 | 22.54 |
|  | ASHB8 | 67.92 | 20.38 | 53.49 | 15.38 | 97.23 | 23.33 |
|  | ASHB9 | 64.79 | 19.44 | 50.48 | 14.72 | 97.37 | 22.19 |
|  | ASHB10 | 54.78 | 16.44 | 41.20 | 11.95 | 97.17 | 18.15 |
|  | ASHB11 | 86.01 | 25.80 | 83.21 | 24.97 | 97.67 | 36.89 |
|  | ASHB12 | 116.80 | 35.02 | 114.60 | 34.38 | 97.54 | 51.17 |
|  | ASHB13 | 111.80 | 33.54 | 110.10 | 33.03 | 95.68 | 48.00 |
|  | ASHB14 | 71.88 | 21.56 | 70.82 | 21.23 | 93.64 | 30.36 |
|  | ASHB15 | 66.62 | 19.98 | 65.44 | 19.66 | 97.60 | 29.77 |
|  | ASHB16 | 53.02 | 15.90 | 51.95 | 15.58 | 97.49 | 23.69 |
|  | ASHB17 | 73.20 | 21.96 | 72.17 | 21.66 | 97.40 | 32.60 |
|  | ASHB18 | 89.51 | 26.86 | 88.21 | 26.49 | 97.17 | 39.60 |
|  | ASHB19 | 84.55 | 25.36 | 83.48 | 25.06 | 97.54 | 37.71 |
|  | ASHB20 | 123.60 | 37.08 | 119.80 | 35.82 | 97.11 | 52.94 |
| SD | ASSD1 | 54.63 | 16.40 | 40.72 | 11.82 | 97.27 | 18.04 |
|  | ASSD2 | 42.19 | 12.66 | 31.80 | 8.86 | 97.11 | 13.70 |
|  | ASSD3 | 47.78 | 14.34 | 38.84 | 11.21 | 97.28 | 17.19 |
|  | ASSD4 | 68.97 | 20.70 | 56.07 | 16.39 | 97.43 | 24.65 |
|  | ASSD5 | 54.87 | 16.46 | 41.69 | 11.94 | 97.41 | 18.09 |
|  | ASSD6 | 62.61 | 18.78 | 50.68 | 14.60 | 97.08 | 22.27 |
|  | ASSD7 | 58.46 | 17.54 | 46.94 | 13.72 | 97.51 | 20.69 |
|  | ASSD8 | 86.33 | 25.90 | 65.37 | 19.04 | 97.25 | 28.41 |
|  | ASSD9 | 292.40 | 87.72 | 235.50 | 68.65 | 97.42 | 99.66 |
|  | ASSD10 | 65.46 | 19.64 | 63.55 | 18.99 | 93.02 | 27.11 |
|  | ASSD11 | 50.87 | 15.26 | 49.87 | 14.96 | 96.88 | 22.56 |
|  | ASSD12 | 50.98 | 15.30 | 49.52 | 14.87 | 97.71 | 22.62 |
|  | ASSD13 | 57.19 | 17.16 | 56.32 | 16.91 | 97.63 | 25.72 |
|  | ASSD14 | 52.43 | 15.72 | 51.35 | 15.40 | 97.53 | 23.34 |
|  | ASSD15 | 92.01 | 27.60 | 90.57 | 27.19 | 97.59 | 40.78 |
|  | ASSD16 | 48.31 | 14.50 | 47.66 | 14.29 | 97.39 | 21.81 |
|  | ASSD17 | 59.27 | 17.78 | 58.02 | 17.39 | 97.33 | 26.15 |
|  | ASSD18 | 73.93 | 22.18 | 70.52 | 21.18 | 98.17 | 31.42 |
|  | ASSD19 | 61.68 | 18.50 | 60.29 | 18.10 | 95.14 | 26.48 |
|  | ASSD20 | 54.57 | 16.36 | 53.81 | 16.15 | 97.37 | 24.52 |
|  | ASSD21 | 62.85 | 18.86 | 61.22 | 18.39 | 95.79 | 26.96 |
| HLJ | ASHL1 | 65.41 | 19.62 | 56.08 | 16.46 | 97.02 | 23.47 |
|  | ASHL2 | 105.30 | 31.58 | 92.76 | 27.31 | 98.58 | 38.20 |
|  | ASHL3 | 89.60 | 26.88 | 79.01 | 23.29 | 98.43 | 33.29 |
|  | ASHL4 | 97.78 | 29.34 | 84.82 | 24.97 | 98.36 | 35.45 |
|  | ASHL5 | 93.42 | 28.02 | 80.86 | 23.83 | 98.21 | 34.77 |
|  | ASHL6 | 71.23 | 21.36 | 59.29 | 17.37 | 93.52 | 24.28 |
|  | ASHL7 | 82.89 | 24.86 | 70.25 | 20.63 | 97.87 | 29.73 |
|  | ASHL8 | 59.56 | 17.86 | 47.33 | 13.81 | 97.55 | 20.89 |
|  | ASHL9 | 72.22 | 21.66 | 62.55 | 18.44 | 97.92 | 27.80 |
|  | ASHL10 | 68.99 | 20.70 | 58.40 | 17.14 | 97.67 | 25.30 |
| YN | ASYN1 | 17.22 | 5.16 | 16.50 | 4.95 | 97.80 | 7.72 |
|  | ASYN2 | 49.61 | 14.88 | 48.03 | 14.44 | 97.32 | 21.87 |
|  | ASYN3 | 125.70 | 37.70 | 123.60 | 37.25 | 97.55 | 55.14 |
|  | ASYN4 | 114.90 | 34.46 | 112.60 | 33.92 | 97.47 | 49.90 |
|  | ASYN5 | 134.80 | 40.42 | 132.50 | 39.93 | 97.54 | 58.96 |
|  | ASYN6 | 71.39 | 21.42 | 65.64 | 19.79 | 97.38 | 29.68 |

**Table S6. Summary of the SNPs annotation**

| **Variant type** | **Count** | **Percent (%)** |
| --- | --- | --- |
| initiator_codon_variant | 15 | 0.001 |
| intergenic_region | 558109 | 37.743 |
| intron_variant | 237797 | 16.081 |
| missense_variant | 32706 | 2.212 |
| synonymous_variant | 236797 | 16.014 |
| upstream_gene_variant | 189533 | 12.810 |
| downstream_gene_variant | 207960 | 14.064 |
| non_coding_transcript_variant | 3 | 0.000 |
| splice_acceptor_variant | 97 | 0.007 |
| splice_donor_variant | 153 | 0.010 |
| splice_region_variant | 14948 | 1.011 |
| start_lost | 65 | 0.004 |
| stop_gained | 347 | 0.023 |
| stop_lost | 53 | 0.004 |
| stop_retained_variant | 116 | 0.008 |
| 5_prime_UTR_variant | 6 | 0.000 |
| annotated SNPs | 1,478,705 | 1 |
| total | 1065969 |  |

**Table S7. Length distribution of SVs in different categories**

| **Category** | **50-100bp** | **100-500bp** | **500bp-1k** | **1k-2k** | **2k-5k** | **5k-10K** | | **>10K** | **All** |
| --- | --- | --- | --- | --- | --- | --- | --- | --- | --- |
| DEL | 4,791 | 11,862 | 8,652 | 4,806 | 1,797 | | 413 | 146 | 32,467 |
| DUP | 0 | 470 | 252 | 116 | 119 | | 66 | 97 | 1,120 |
| INV | 6 | 282 | 254 | 183 | 166 | | 78 | 492 | 1,461 |
| INS | 21 | 0 | 0 | 0 | 0 | | 0 | 0 | 21 |

**Table S8. Genes of NTC selected region genes identified by CLR analysis.**

| **Gene ID** | **Contig** | **Annotation** |
| --- | --- | --- |
| AS008693 | contig_227 | DNA-directed RNA polymerase II subunit RPB1 |
| AS001981 | contig_57 | hypothetical protein B5V51_5309 |
| AS014936 | contig_635 | dual specificity protein phosphatase |
| AS009626 | contig_250 | proton-coupled folate transporter |
| AS003661 | contig_98 | replication termination factor |
| AS003662 | contig_98 | pre-rRNA-processing protein esf2 |
| AS003663 | contig_98 | uncharacterized protein LOC110381835 |
| AS003664 | contig_98 | coiled-coil-helix-coiled-coil-helix domain-containing protein 3 |
| AS017568 | contig_1624 | solute carrier family 46-member 3 |
| AS017569 | contig_1624 | proton-coupled folate transporter |
| AS000998 | contig_34 | endoribonuclease Dicer |
| AS000997 | contig_34 | uncharacterized protein LOC113522127 |
| AS011255 | contig_327 | scm-like with four MBT domains protein |
| AS011256 | contig_327 | CSC1-like protein 2 |
| AS001464 | contig_37 | hypothetical protein evm_011754 |
| AS018164 | contig_1899 | solute carrier family 25-member 35 |
| AS018166 | contig_1899 | uncharacterized protein LOC114355240 |
| AS018167 | contig_1899 | uncharacterized protein LOC110369699 |
| AS018165 | contig_1899 | uncharacterized protein LOC113508781 |
| AS006039 | contig_162 | ATP-binding cassette |
| AS006040 | contig_162 | hypothetical protein B5V51_5265 |
| AS006041 | contig_162 | NA |
| AS006042 | contig_162 | ATP-binding cassette |
| AS001098 | contig_34 | serine/threonine-protein kinase MRCK |
| AS016833 | contig_1203 | ranscription factor EB isoform X4 |
| AS000932 | contig_31 | octopamine receptor |
| AS000751 | contig_29 | KAT8 regulatory NSL complex subunit 2 |
| AS012746 | contig_446 | multiple inositol polyphosphate phosphatase 1 |
| AS018422 | contig_1957 | NA |
| AS018423 | contig_1957 | DCN1-like protein 3 |
| AS018424 | contig_1957 | eukaryotic peptide chain release factor GTP-binding subunit ERF3A |
| AS012731 | contig_446 | cationic amino acid transporter 2 |
| AS007423 | contig_193 | NA |
| AS007424 | contig_193 | hypothetical protein B5V51_4056 |
| AS007425 | contig_193 | probable nucleoporin Nup58 isoform X1 |
| AS010211 | contig_264 | uncharacterized protein LOC114363178 |
| AS010061 | contig_260 | hypothetical protein B5V51_2600 |
| AS005995 | contig_162 | uncharacterized protein LOC111351402 |
| AS008297 | contig_218 | hypothetical protein B5V51_855 |
| AS008296 | contig_218 | hypothetical protein B5V51_7055 |
| AS016111 | contig_932 | intermediate filament protein if |
| AS016112 | contig_932 | AP-3 complex subunit mu |
| AS012069 | contig_352 | protein mahjong |
| AS013190 | contig_489 | uncharacterized protein LOC110374702 |
| AS013191 | contig_489 | SUMO-conjugating enzyme UBC9-A |
| AS016756 | contig_1110 | methyltransferase-like protein 13 |
| AS014028 | contig_566 | fatty acid synthase |
| AS018303 | contig_1933 | protein tipE isoform X1 |
| AS002569 | contig_70 | uncharacterized protein K02A2.6 |
| AS002570 | contig_70 | uncharacterized protein K02A2.6 |
| AS016734 | contig_1110 | optic atrophy protein 1 |
| AS016735 | contig_1110 | unnamed protein product |
| AS017924 | contig_1887 | eukaryotic translation initiation factor 5B |
| AS008471 | contig_219 | glutamate decarboxylase |
| AS011546 | contig_338 | uncharacterized protein LOC111357782 |
| AS011547 | contig_338 | NA |
| AS011548 | contig_338 | hypothetical protein B5V51_7778 |
| AS016223 | contig_944 | protein patched |
| AS016222 | contig_944 | unnamed protein product |
| AS012104 | contig_352 | hypothetical protein B5V51_8330 |
| AS004435 | contig_134 | head-specific guanylate cyclase |
| AS004094 | contig_115 | zinc finger protein 652 |
| AS012462 | contig_378 | fasciclin-2 |
| AS013314 | contig_496 | Retrotransposable element Tf2 155 kDa protein |
| AS013315 | contig_496 | probable malonyl-CoA-acyl carrier protein transacylase |
| AS007195 | contig_175 | serine/threonine-protein phosphatase rdgC |
| AS000472 | contig_10 | limbic system-associated membrane protein |
| AS003631 | contig_98 | uncharacterized protein LOC111353355 |
| AS006810 | contig_171 | titin homolog |
| AS013001 | contig_481 | formin-2 |
| AS015081 | contig_656 | retinoblastoma-binding protein 5 |
| AS015080 | contig_656 | remodeling and spacing factor 1 |
| AS015082 | contig_656 | poly (ADP-ribose) glycohydrolase |
| AS003385 | contig_86 | solute carrier family 4 (anion exchanger) |
| AS013401 | contig_507 | obscurin isoform X3 |
| AS003942 | contig_113 | hypothetical protein B5V51_8915 |
| AS004944 | contig_140 | protein sickie isoform X6 |
| AS000962 | contig_34 | NA |
| AS017440 | contig_1567 | dynein heavy chain |
| AS002563 | contig_66 | NA |
| AS002564 | contig_66 | NA |
| AS005156 | contig_149 | uncharacterized protein LOC110384439 |
| AS005157 | contig_149 | VWFA and cache domain-containing protein CG16868 |
| AS017566 | contig_1624 | proton-coupled folate transporter |
| AS008455 | contig_219 | paired amphipathic helix protein Sin3b |
| AS006987 | contig_174 | glycerol kinase |
| AS001438 | contig_37 | orexin receptor type 1 |
| AS010964 | contig_310 | hypothetical protein B5V51_1295 |
| AS010965 | contig_310 | uncharacterized protein LOC110378845 |
| AS002941 | contig_82 | prophenoloxidase 1 |
| AS005799 | contig_159 | DNA topoisomerase 2-associated protein PAT1 |
| AS000613 | contig_28 | organic cation transporter protein |
| AS000614 | contig_28 | organic cation transporter protein |
| AS011246 | contig_325 | GATA zinc finger domain containing protein 1 |
| AS011247 | contig_325 | U6 snRNA-associated Sm-like protein LSm6 |
| AS011248 | contig_325 | uncharacterized protein LOC113506518 |
| AS011249 | contig_325 | uncharacterized protein LOC111364838 |
| AS004630 | contig_138 | peroxidase |
| AS012875 | contig_481 | protein rhomboid |
| AS012876 | contig_481 | hypothetical protein B5X24_HaOG207640 |
| AS003576 | contig_96 | angiotensin-converting enzyme |
| AS000451 | contig_10 | ATP-binding cassette |
| AS004566 | contig_138 | hypothetical protein B5X24_HaOG208365 |
| AS011333 | contig_327 | hypothetical protein B5X24_HaOG212791 |
| AS006163 | contig_168 | NA |
| AS009827 | contig_251 | hypothetical protein B5V51_420 |
| AS009828 | contig_251 | unnamed protein product |
| AS015150 | contig_677 | uncharacterized protein LOC111348327 |
| AS007809 | contig_203 | RNA-binding protein fusilli |
| AS000963 | contig_34 | hypothetical protein B5V51_6155 |
| AS000964 | contig_34 | hypothetical protein evm_012717 |
| AS017812 | contig_1862 | hypothetical protein B5V51_2845 |
| AS017813 | contig_1862 | uncharacterized protein LOC110373825 |
| AS013372 | contig_505 | odorant binding protein |
| AS013373 | contig_505 | odorant binding protein |
| AS014801 | contig_633 | zinc finger protein 184 |
| AS014802 | contig_633 | putative zinc finger protein 286B |
| AS005284 | contig_152 | insulin gene enhancer protein ISL-1 |
| AS000640 | contig_28 | polyprotein |
| AS006197 | contig_169 | hypothetical protein B5X24_HaOG203521 |
| AS006196 | contig_169 | NA |
| AS011903 | contig_343 | lysosome membrane protein 2 |
| AS005259 | contig_151 | hypothetical protein B5X24_HaOG212693 |
| AS002453 | contig_66 | fatty acyl-CoA reductase |
| AS013603 | contig_520 | arginyl-tRNA synthetase |
| AS013602 | contig_520 | WD repeat-containing protein 19 |
| AS012475 | contig_378 | NA |
| AS012476 | contig_378 | uncharacterized protein LOC111352613 |
| AS009051 | contig_238 | CDK5 regulatory subunit-associated protein 1 |
| AS009050 | contig_238 | hypothetical protein B5V51_10073 |
| AS008234 | contig_216 | NA |
| AS008235 | contig_216 | hypothetical protein B5V51_9167 |
| AS002486 | contig_66 | laccase 1 |
| AS017930 | contig_1888 | uncharacterized protein LOC113497488 |
| AS014110 | contig_569 | uncharacterized protein LOC114331484 |
| AS011353 | contig_330 | hypothetical protein B5V51_9932 |
| AS001596 | contig_40 | hypothetical protein B5V51_5656 |
| AS007481 | contig_194 | tyrosine-protein phosphatase non-receptor type 21 |
| AS004315 | contig_129 | acyl-CoA delta 11 desaturase |
| AS008704 | contig_227 | cyclic AMP response element-binding protein A |
| AS008705 | contig_227 | heparan-alpha-glucosaminide N-acetyltransferase |
| AS013527 | contig_512 | putative RNA-directed DNA polymerase from transposon BS |
| AS017894 | contig_1887 | sodium/potassium-transporting ATPase subunit beta |
| AS014775 | contig_633 | calpain-A |
| AS002180 | contig_58 | uncharacterized protein LOC111357785 |
| AS002718 | contig_76 | NA |
| AS004403 | contig_132 | hypothetical protein B5V51_11785 |
| AS000330 | contig_9 | caspase recruitment domain-containing protein 11 |
| AS000329 | contig_9 | uncharacterized protein LOC110372593 |
| AS005556 | contig_153 | protein disabled |
| AS013584 | contig_515 | xanthine dehydrogenase/oxidase |
| AS013650 | contig_523 | hypothetical protein B5X24_HaOG201578 |
| AS017165 | contig_1420 | phospholipid-translocating ATPase |
| AS008770 | contig_231 | uncharacterized protein LOC113491809 |
| AS008771 | contig_231 | uncharacterized protein LOC110376106 |
| AS016845 | contig_1203 | major heat shock 70 kDa protein Ba |
| AS016847 | contig_1203 | uncharacterized protein LOC112053448 |
| AS016848 | contig_1203 | heat shock protein 70 |
| AS016846 | contig_1203 | hypothetical protein evm_011128 |
| AS010198 | contig_264 | vegetative cell wall protein gp1 |
| AS010199 | contig_264 | cyclin-dependent kinase inhibitor 1C |
| AS010200 | contig_264 | testis-specific gene A8 protein |
| AS009003 | contig_237 | peroxidase |
| AS006993 | contig_174 | hypothetical protein B5V51_6032 |
| AS002874 | contig_80 | KRAB domain-containing zinc finger protein |
| AS003246 | contig_82 | hypothetical protein B5X24_HaOG207426 |
| AS003247 | contig_82 | mevalonate kinase |
| AS007673 | contig_201 | zinc finger protein 595 |
| AS007672 | contig_201 | uncharacterized protein LOC111357956 |
| AS007674 | contig_201 | zinc finger protein 26 |
| AS014316 | contig_601 | uncharacterized protein LOC110375775 |
| AS016160 | contig_933 | protein AAR2 homolog |
| AS016162 | contig_933 | PCI domain-containing protein 2 |
| AS016161 | contig_933 | MKI67 FHA domain-interacting nucleolar phosphoprotein |
| AS001040 | contig_34 | serine/threonine-protein phosphatase with EF-hands |
| AS013498 | contig_512 | Down syndrome cell adhesion molecule |
| AS005225 | contig_151 | target of rapamycin complex subunit lst8 |
| AS005224 | contig_151 | FACT complex subunit Ssrp1 |
| AS012967 | contig_481 | NA |
| AS012968 | contig_481 | 23 kDa integral membrane protein |
| AS000961 | contig_34 | uncharacterized protein LOC110380377 |
| AS003031 | contig_82 | NA |
| AS003032 | contig_82 | alpha-tocopherol transfer protein |
| AS011079 | contig_319 | neurexin |
| AS011190 | contig_325 | Uncharacterized protein K02A2.6 |
| AS015659 | contig_788 | hypothetical protein B5V51_13662 |
| AS011170 | contig_325 | hypothetical protein B5V51_6826 |
| AS008028 | contig_207 | mucin-17 |
| AS007463 | contig_194 | WD and tetratricopeptide repeats protein 1 |
| AS003187 | contig_82 | sex-lethal homolog isoform X1 |
| AS003186 | contig_82 | transmembrane protease serine 9 |
| AS009663 | contig_250 | farnesyl diphosphate synthase |
| AS001528 | contig_37 | hypothetical protein B5V51_12384 |
| AS000087 | contig_4 | apoptosis-inducing factor 1 |
| AS002183 | contig_58 | unnamed protein product |
| AS002184 | contig_58 | unnamed protein product |
| AS011744 | contig_338 | ribosome biogenesis protein YTM1 |
| AS006383 | contig_169 | LIM/homeobox protein Lhx1 |
| AS018432 | contig_1957 | nuclear distribution protein nudE |
| AS018431 | contig_1957 | protein disulfide isomerase family A |
| AS013308 | contig_496 | leucine carboxyl methyltransferase 1 |
| AS004275 | contig_129 | hypothetical protein B5V51_1968 |
| AS004276 | contig_129 | glutathione peroxidase |
| AS004274 | contig_129 | protein SON |
| AS007342 | contig_185 | ABC transporter G family member 20 |
| AS001710 | contig_40 | neuropathy target esterase sws isoform X3 |
| AS001413 | contig_37 | tRNA (cytosine34-C5)-methyltransferase |
| AS000326 | contig_9 | NA |
| AS009464 | contig_245 | E3 ubiquitin-protein ligase CHIP |
| AS012103 | contig_352 | transmembrane protease serine 9 |
| AS013273 | contig_493 | hypothetical protein B5V51_830 |
| AS013274 | contig_493 | ATP-binding cassette |
| AS017151 | contig_1400 | prolyl 4-hydroxylase |
| AS008275 | contig_217 | RAP1 GTPase activating protein 1 |
| AS009628 | contig_250 | hypothetical protein B5X24_HaOG207006 |
| AS000053 | contig_4 | unnamed protein product |
| AS000054 | contig_4 | hypothetical protein AB894_15215 |
| AS011893 | contig_343 | ankyrin repeat domain-containing protein 50 |
| AS008388 | contig_218 | hypoxia up-regulated protein 1 |
| AS008389 | contig_218 | forkhead box protein N3 |
| AS000304 | contig_9 | hypothetical protein B5V51_8051 |
| AS000305 | contig_9 | fatty acyl reductase |
| AS006510 | contig_171 | neurocalcin homolog |
| AS015630 | contig_788 | p77 homologue |
| AS015631 | contig_788 | NA |
| AS009118 | contig_239 | neuropeptide F receptor |
| AS017802 | contig_1855 | vacuolar protein sorting-associated protein 52 |
| AS017803 | contig_1855 | uncharacterized protein LOC110371892 |
| AS005734 | contig_156 | odorant binding protein |
| AS001052 | contig_34 | hypothetical protein B5V51_3074 |
| AS001053 | contig_34 | uncharacterized protein LOC110379650 |
| AS002526 | contig_66 | F-box/LRR-repeat protein 14 |
| AS017956 | contig_1889 | hypothetical protein RR48_01241 |
| AS017957 | contig_1889 | NA |
| AS017958 | contig_1889 | uncharacterized protein LOC110381960 |
| AS000868 | contig_31 | anaphase-promoting complex subunit 4 |
| AS008802 | contig_235 | unnamed protein product |
| AS008803 | contig_235 | hypothetical protein B5V51_10841 |
| AS005978 | contig_162 | leucine-rich repeat kinase 1 |
| AS007718 | contig_203 | dystrobrevin beta |
| AS005291 | contig_152 | hypothetical protein B5V51_9763 |
| AS001595 | contig_40 | NA |
| AS001177 | contig_34 | NA |
| AS001178 | contig_34 | hypothetical protein AB894_15215 |
| AS000349 | contig_9 | glyoxalase domain-containing protein 4 |
| AS005456 | contig_153 | phosphatidate phosphatase |
| AS011466 | contig_336 | hypothetical protein B5V51_9932 |
| AS008707 | contig_227 | phosphatidylinositol 4-phosphate 3-kinase C2 domain |
| AS005362 | contig_152 | UDP-glycosyltransferase 33V1 |
| AS003214 | contig_82 | solute carrier family 13 (sodium-dependent dicarboxylate transporter) |
| AS016535 | contig_1078 | uncharacterized protein LOC110371643 |
| AS016536 | contig_1078 | delta-9 desaturase |
| AS006890 | contig_174 | uncharacterized protein LOC110379374 |
| AS006892 | contig_174 | porphobilinogen synthase |
| AS006893 | contig_174 | prickle |
| AS006894 | contig_174 | hypothetical protein B5X24_HaOG206296 |
| AS006891 | contig_174 | hypothetical protein B5V51_1299 |
| AS012567 | contig_414 | antigen KI-67 |
| AS012568 | contig_414 | protein C12orf4 homolog |
| AS003680 | contig_98 | NA |
| AS003681 | contig_98 | NA |
| AS003679 | contig_98 | ABC transporter A family member 3 |
| AS006489 | contig_171 | Sensory neuron membrane protein 1 |
| AS005174 | contig_151 | NA |
| AS013636 | contig_523 | DNA excision repair protein ERCC-3 |
| AS013638 | contig_523 | NA |
| AS013637 | contig_523 | SWI/SNF-related matrix-associated actin-dependent regulator of chromatin subfamily A-like protein 1 |
| AS017649 | contig_1664 | titin |
| AS016140 | contig_933 | unnamed protein product |
| AS006695 | contig_171 | prostaglandin E synthase 2 |
| AS006696 | contig_171 | hypothetical protein B5V51_10515 |
| AS014439 | contig_604 | uncharacterized protein LOC115447671 |
| AS014440 | contig_604 | uncharacterized protein LOC111359073 |
| AS009986 | contig_258 | putative adenosylhomocysteinase 3 |
| AS001689 | contig_40 | receptor-mediated endocytosis protein 6 |
| AS007560 | contig_197 | uncharacterized protein LOC110375305 |
| AS007561 | contig_197 | calcium/calmodulin-dependent protein kinase II |
| AS014682 | contig_629 | hypothetical protein B5V51_15015 |
| AS014681 | contig_629 | homeobox protein CDX-1 |
| AS013185 | contig_489 | uncharacterized protein LOC111347763 |
| AS013186 | contig_489 | DNA repair protein RAD50 |
| AS008885 | contig_235 | low-density lipoprotein receptor-related protein 1 |
| AS010326 | contig_270 | NA |
| AS010327 | contig_270 | oocyte zinc finger protein XlCOF6 |
| AS005445 | contig_153 | uncharacterized protein LOC113503928 |
| AS002037 | contig_58 | 5'-nucleotidase |
| AS003834 | contig_107 | NA |
| AS003835 | contig_107 | endonuclease-reverse transcriptase |
| AS003836 | contig_107 | unnamed protein product |
| AS005914 | contig_161 | elongation of very long chain fatty acids protein 7 |
| AS011583 | contig_338 | tectonin beta-propeller repeat-containing protein 1 |
| AS011582 | contig_338 | splicing factor 3B subunit 1 |
| AS006612 | contig_171 | amyloid beta A4 precursor protein-binding family B member 1-interacting protein |
| AS002893 | contig_81 | uncharacterized protein KIAA1109 |
| AS010041 | contig_259 | LIM domain-binding protein 3 |
| AS005998 | contig_162 | hypothetical protein B5V51_9987 |
| AS005999 | contig_162 | transferin |
| AS003599 | contig_96 | hypothetical protein AB894_15215 |
| AS003600 | contig_96 | RNA binding protein fox-1 |
| AS003040 | contig_82 | very-long-chain 3-oxoacyl-CoA reductase |
| AS004634 | contig_138 | mediator of RNA polymerase II transcription subunit 4 |
| AS004635 | contig_138 | putative inner dynein arm light chain |
| AS010947 | contig_308 | transmembrane protease serine 9 |
| AS010218 | contig_265 | heparan sulfate 6-O-sulfotransferase HS6ST1 |
| AS002134 | contig_58 | regulator of G-protein signaling |
| AS001775 | contig_41 | hypothetical protein B5V51_3973 |
| AS001776 | contig_41 | uncharacterized protein LOC111354482 |
| AS001777 | contig_41 | uncharacterized protein LOC110373934 |
| AS005449 | contig_153 | AP-3 complex subunit mu |
| AS002769 | contig_78 | dynein heavy chain |
| AS014315 | contig_601 | fibulin 1/2 |
| AS000841 | contig_31 | transmembrane protease serine 2 |
| AS000842 | contig_31 | hypothetical protein RR46_04047 |
| AS010881 | contig_308 | hypothetical protein B5V51_14569 |
| AS009417 | contig_244 | uncharacterized protein LOC113230887 |
| AS010953 | contig_308 | clavesin-2 |
| AS000917 | contig_31 | xenotropic and polytropic retrovirus receptor 1 |
| AS005862 | contig_159 | sorting and assembly machinery component 50 |
| AS005861 | contig_159 | MFS transporter |
| AS008449 | contig_219 | homeobox protein GBX |
| AS005806 | contig_159 | acetyl-CoA carboxylase |
| AS010683 | contig_288 | protein real-time |
| AS002715 | contig_76 | hypothetical protein EVAR_35820_1 |
| AS010900 | contig_308 | furin-like protease 2 |
| AS002495 | contig_66 | NA |
| AS002496 | contig_66 | hypothetical protein B5V51_10841 |
| AS002497 | contig_66 | RNA binding protein fox-1 |
| AS013041 | contig_484 | uncharacterized protein LOC113507375 |
| AS013042 | contig_484 | NA |
| AS015961 | contig_891 | urate oxidase |
| AS015960 | contig_891 | MAGUK p55 subfamily member 6 |
| AS015962 | contig_891 | uncharacterized protein LOC110371087 |
| AS015010 | contig_654 | NEDD8-activating enzyme E1 catalytic subunit |
| AS015012 | contig_654 | F-type H+-transporting ATPase subunit beta |
| AS015013 | contig_654 | ubiquinol-cytochrome-c reductase complex assembly factor 1 |
| AS015011 | contig_654 | uncharacterized protein LOC110374329 |
| AS004518 | contig_135 | nose resistant to fluoxetine protein 6 |
| AS016675 | contig_1107 | Receptor-type tyrosine-protein phosphatase kappa |
| AS014790 | contig_633 | beclin 1 |
| AS014791 | contig_633 | uncharacterized protein LOC110381487 |
| AS010348 | contig_270 | methylenetetrahydrofolate dehydrogenase (NADP+) |
| AS010350 | contig_270 | NA |
| AS010351 | contig_270 | NA |
| AS010349 | contig_270 | NA |
| AS005120 | contig_144 | thioredoxin domain-containing protein 3 |
| AS005121 | contig_144 | kelch-like protein 20 |
| AS005142 | contig_149 | optic atrophy protein 1 |
| AS017988 | contig_1892 | uncharacterized protein LOC111353864 |
| AS015901 | contig_872 | tribbles homolog 2 |
| AS008368 | contig_218 | hypothetical protein B5X24_HaOG213347 |
| AS008369 | contig_218 | NA |
| AS008370 | contig_218 | Niemann-Pick C2 protein |
| AS007687 | contig_202 | ATP-binding cassette |
| AS011889 | contig_343 | hypothetical protein B5V51_3376 |
| AS015463 | contig_765 | hypothetical protein B5V51_11512 |
| AS015464 | contig_765 | neither inactivation nor afterpotential protein C |
| AS005756 | contig_156 | hypothetical protein B5V51_4982 |
| AS005757 | contig_156 | uncharacterized protein LOC111356588 |
| AS012918 | contig_481 | disintegrin and metalloproteinase domain-containing protein |
| AS012646 | contig_433 | uncharacterized protein LOC114243001 |
| AS012647 | contig_433 | SWI/SNF-related matrix-associated actin-dependent regulator of chromatin subfamily A-like protein 1 |
| AS015031 | contig_654 | DNA-directed RNA polymerase II subunit RPB1 |
| AS007690 | contig_202 | ATP-binding cassette |
| AS007691 | contig_202 | polypeptide N-acetylgalactosaminyltransferase |
| AS003790 | contig_107 | zinc finger and BTB domain-containing protein 48 |
| AS004562 | contig_138 | NA |
| AS010255 | contig_268 | integrin alpha-X |
| AS006689 | contig_171 | multidrug resistance protein 49 |
| AS016292 | contig_969 | putative nuclease HARBI1 |
| AS016293 | contig_969 | uncharacterized protein LOC114350010 |
| AS014840 | contig_635 | chromatin assembly factor 1 subunit B |
| AS008183 | contig_216 | retinol dehydrogenase 12 |
| AS008184 | contig_216 | lysosomal acid phosphatase |
| AS008726 | contig_228 | uncharacterized protein LOC111353114 |
| AS016661 | contig_1107 | NA |
| AS016298 | contig_969 | queuine tRNA-ribosyltransferase |
| AS010230 | contig_265 | odorant degrading enzyme CXE14 |
| AS010251 | contig_268 | guanine nucleotide-binding protein subunit alpha |
| AS001365 | contig_37 | actin-binding LIM protein |
| AS005388 | contig_153 | NA |
| AS005387 | contig_153 | RNA 3'-terminal phosphate cyclase (ATP) |
| AS005389 | contig_153 | minor histocompatibility antigen H13 |
| AS000743 | contig_29 | phosphatidylinositol 4-phosphate 5-kinase type-1 alpha |
| AS009857 | contig_252 | solute carrier family 7 (L-type amino acid transporter) |
| AS014726 | contig_633 | irregular chiasm C-roughest protein |
| AS009097 | contig_238 | uncharacterized protein LOC111354080 |
| AS009098 | contig_238 | unnamed protein product |
| AS015481 | contig_772 | uncharacterized protein LOC111355111 |
| AS015482 | contig_772 | hypothetical protein B5V51_7975 |
| AS015264 | contig_688 | NA |
| AS000473 | contig_12 | RNA-directed DNA polymerase |
| AS012035 | contig_345 | arylsulfatase B |
| AS010589 | contig_286 | ATP-dependent RNA helicase spindle-E |
| AS003620 | contig_96 | protein spaetzle-like |
| AS005500 | contig_153 | hypothetical protein B5V51_4746 |
| AS010600 | contig_286 | synaptosomal-associated protein 25 |
| AS005179 | contig_151 | G protein-coupled receptor Mth |
| AS001362 | contig_37 | uncharacterized protein LOC110382065 |
| AS001363 | contig_37 | transcription factor AP-2 |
| AS012627 | contig_430 | low molecular mass 30 kDa lipoprotein 21G1 |
| AS008203 | contig_216 | carcinine transporter |
| AS002826 | contig_80 | heat shock 70 kDa protein cognate 5 |
| AS005800 | contig_159 | acetate esterase 18 |
| AS010580 | contig_286 | Down syndrome cell adhesion molecule-like protein Dscam2 |
| AS009809 | contig_251 | L-dopachrome tautomerase yellow-f2 |
| AS009808 | contig_251 | gelsolin |
| AS011394 | contig_333 | MFS transporter |
| AS005352 | contig_152 | hypothetical protein B5V51_3515 |
| AS012694 | contig_446 | RNA binding protein fox-1 |
| AS001221 | contig_34 | cellular nucleic acid-binding protein |
| AS001222 | contig_34 | optic atrophy protein 1 |
| AS012782 | contig_448 | serine carboxypeptidase 1 |
| AS011169 | contig_325 | probable phytanoyl-CoA dioxygenase |
| AS017989 | contig_1892 | neuropeptide CCHamide-2 receptor |
| AS015122 | contig_662 | dynein heavy chain 1 |
| AS004547 | contig_138 | cubilin-like |
| AS005703 | contig_156 | 20S proteasome subunit alpha 7 |
| AS018029 | contig_1893 | uncharacterized protein LOC111362399 |
| AS007648 | contig_201 | low-density lipoprotein receptor-related protein |
| AS016034 | contig_917 | NADH-cytochrome b5 reductase 2 |
| AS015213 | contig_683 | WD and tetratricopeptide repeats protein 1 |
| AS007830 | contig_204 | NA |
| AS015054 | contig_655 | lipase member H-A |
| AS015055 | contig_655 | uncharacterized protein LOC111348357 |
| AS015056 | contig_655 | aldehyde dehydrogenase (NAD+) |
| AS012940 | contig_481 | leucine-rich repeat flightless-interacting protein 2 |
| AS017856 | contig_1879 | glucose-6-phosphate isomerase |
| AS017855 | contig_1879 | flap endonuclease-1 |
| AS009107 | contig_238 | S-adenosylmethionine decarboxylase |
| AS000872 | contig_31 | solute carrier family 10 (sodium/bile acid cotransporter) |
| AS013057 | contig_486 | globin-2 A chain |
| AS013058 | contig_486 | deoxyhypusine hydroxylase |
| AS013059 | contig_486 | hypothetical protein B5X24_HaOG211462 |
| AS012021 | contig_345 | UDP-glycosyltransferase 33T3 |
| AS009622 | contig_250 | diuretic hormone receptor |
| AS016124 | contig_932 | WD repeat-containing protein 35 |
| AS015682 | contig_804 | prominin |
| AS007656 | contig_201 | hypothetical protein B5V51_10989 |
| AS013306 | contig_496 | cytochrome b5 |
| AS013307 | contig_496 | integrator complex subunit 9 |
| AS016437 | contig_1036 | ubiquitin carboxyl-terminal hydrolase BAP1 |
| AS016438 | contig_1036 | clustered mitochondria protein |
| AS005820 | contig_159 | zinc finger protein 182 |
| AS015215 | contig_685 | A disintegrin and metalloproteinase with thrombospondin motifs 1 |
| AS014252 | contig_577 | uncharacterized protein LOC110383798 |
| AS000351 | contig_9 | hypothetical protein B5X24_HaOG212190 |
| AS000352 | contig_9 | NA |
| AS003927 | contig_113 | uncharacterized protein LOC110370198 |
| AS001971 | contig_57 | synaptojanin |
| AS004244 | contig_129 | transferrin |
| AS005077 | contig_142 | NA |
| AS006038 | contig_162 | NA |
| AS010637 | contig_288 | nitrogen permease regulator 2-like protein |
| AS014956 | contig_652 | glucose dehydrogenase |
| AS001576 | contig_38 | NA |
| AS011813 | contig_340 | chitinase |
| AS015657 | contig_788 | uncharacterized protein LOC110383219 |
| AS015658 | contig_788 | NA |
| AS016133 | contig_932 | membrane dipeptidase |
| AS015073 | contig_656 | aminoacylase |
| AS002595 | contig_70 | nuclear pore complex protein Nup214 |
| AS010094 | contig_262 | small subunit ribosomal protein S30 |
| AS010093 | contig_262 | elongator complex protein 1 |
| AS017556 | contig_1624 | hypothetical protein B5V51_911 |
| AS006639 | contig_171 | nucleobindin |
| AS009237 | contig_240 | RecName: Full=Neuropeptide CCHamide-2; Short=CCHa; Flags: Precursor |
| AS014068 | contig_566 | serine/threonine-protein kinase Chk1 |
| AS014069 | contig_566 | serine/threonine-protein kinase Chk1 |
| AS016953 | contig_1265 | glycogen synthase kinase 3 beta |
| AS016952 | contig_1265 | Vam6/Vps39-like protein vacuolar protein sorting-associated protein 39 |
| AS008119 | contig_208 | hypothetical protein B5V51_11035 |
| AS008118 | contig_208 | fatty alcohol acetyltransferase |
| AS008120 | contig_208 | traB domain-containing protein |
| AS003603 | contig_96 | acid sphingomyelinase-like phosphodiesterase 3a |
| AS001648 | contig_40 | alpha 1,4-N-acetylglucosaminyltransferase |
| AS016464 | contig_1053 | sodium channel protein Nach |
| AS016465 | contig_1053 | sodium channel protein Nach |
| AS007072 | contig_174 | protein apterous |
| AS013292 | contig_493 | hypothetical protein B5V51_2068 |
| AS001588 | contig_38 | NA |
| AS015036 | contig_654 | centrosomal protein of 290 kDa |
| AS015037 | contig_654 | centrosomal protein of 290 kDa |
| AS015038 | contig_654 | unnamed protein product |
| AS018024 | contig_1893 | dihydropyrimidinase |
| AS006445 | contig_171 | phosphopantothenate-cysteine ligase |
| AS001973 | contig_57 | NA |
| AS001974 | contig_57 | protein turtle isoform X2 |
| AS009710 | contig_251 | cytochrome c oxidase subunit 8 |
| AS009711 | contig_251 | zonadhesin |
| AS014047 | contig_566 | WD repeat-containing protein on Y chromosome |
| AS014045 | contig_566 | WD repeat-containing protein on Y chromosome |
| AS014046 | contig_566 | WD repeat-containing protein on Y chromosome |
| AS010079 | contig_261 | methylenetetrahydrofolate dehydrogenase (NADP+) |
| AS013486 | contig_510 | tryptophan 5-monooxygenase |
| AS013487 | contig_510 | TBC1 domain family member 24 |
| AS010402 | contig_274 | beta-1,3-galactosyltransferase 1 |
| AS010403 | contig_274 | pancreatic triacylglycerol lipase |
| AS015840 | contig_843 | NA |
| AS015841 | contig_843 | putative defensin-like protein precursor |
| AS015843 | contig_843 | putative defensin-like protein precursor |
| AS015844 | contig_843 | putative defensin-like protein precursor |
| AS015842 | contig_843 | putative defensin-like protein precursor |
| AS016302 | contig_969 | uncharacterized protein LOC111359979 |
| AS012643 | contig_432 | uncharacterized protein LOC111362300 |
| AS011714 | contig_338 | GTP binding protein |
| AS001220 | contig_34 | hypothetical protein B5X24_HaOG212154 |
| AS001976 | contig_57 | hrp65 protein |
| AS001043 | contig_34 | putative nuclease HARBI1 |
| AS016014 | contig_901 | testis-expressed protein 2 |
| AS006645 | contig_171 | solute carrier family 15 (oligopeptide transporter) |
| AS005825 | contig_159 | uncharacterized protein LOC111354891 |
| AS013180 | contig_489 | protein singed wings 2 |
| AS009593 | contig_250 | aspartyl-tRNA (Asn)/glutamyl-tRNA (Gln) amidotransferase subunit B |
| AS017171 | contig_1420 | ribosome biogenesis protein MAK21 |
| AS009243 | contig_240 | activin receptor type-2 |
| AS009242 | contig_240 | NA |
| AS006231 | contig_169 | cytochrome P450 |
| AS003123 | contig_82 | cytochrome P450 |
| AS003122 | contig_82 | cytochrome P450 |
| AS006670 | contig_171 | discoidin domain receptor family member 2 |
| AS000306 | contig_9 | Macrophage mannose receptor 1 |
| AS000307 | contig_9 | Macrophage mannose receptor 1 |
| AS003999 | contig_114 | carbohydrate 6-sulfotransferase 6 |
| AS004000 | contig_114 | carbohydrate sulfotransferase 4 |
| AS000335 | contig_9 | uncharacterized protein LOC116770084 |
| AS000336 | contig_9 | actin-binding LIM protein |
| AS000001 | contig_1 | tubby-related protein 4 |
| AS001910 | contig_45 | dynein regulatory complex subunit |
| AS010231 | contig_265 | cholinesterase 1 |
| AS010232 | contig_265 | hypothetical protein B5V51_3602 |
| AS015655 | contig_788 | P protein-like isoform |
| AS011018 | contig_315 | major facilitator superfamily domain-containing protein 10 |
| AS011019 | contig_315 | splicing factor U2AF 50 kDa subunit |
| AS011020 | contig_315 | uncharacterized protein LOC110376355 |
| AS014476 | contig_607 | protein split ends-like |
| AS001629 | contig_40 | NA |
| AS001630 | contig_40 | hypothetical protein B5V51_12355 |
| AS001631 | contig_40 | xanthine dehydrogenase/oxidase |

Notes: NA (not applicable).

**Table S9. Genes of NEC selected region genes identified by CLR analysis.**

| **Gene ID** | **Contig** | **Annotation** |
| --- | --- | --- |
| AS006970 | contig_174 | unnamed protein product |
| AS000394 | contig_9 | protein PRR14L |
| AS000395 | contig_9 | neuferricin homolog |
| AS004932 | contig_140 | bromodomain-containing protein 3 |
| AS004933 | contig_140 | metal-response element-binding transcription factor 2 |
| AS004934 | contig_140 | leucine-rich repeat-containing protein |
| AS016130 | contig_932 | collagen alpha-1(IV) chain |
| AS006442 | contig_171 | NA |
| AS000397 | contig_9 | gametogenetin-binding protein |
| AS000396 | contig_9 | 3-oxoacyl-[acyl-carrier-protein] synthase |
| AS000398 | contig_9 | ribosome maturation protein SBDS |
| AS006799 | contig_171 | unnamed protein product |
| AS006798 | contig_171 | gamma-aminobutyric acid receptor subunit beta |
| AS007246 | contig_183 | D-beta-hydroxybutyrate dehydrogenase |
| AS004248 | contig_129 | oligoribonuclease |
| AS004249 | contig_129 | LETM1 and EF-hand domain-containing protein 1 |
| AS001098 | contig_34 | serine/threonine-protein kinase MRCK |
| AS004931 | contig_140 | high affinity copper uptake protein 1-like |
| AS006810 | contig_171 | titin homolog |
| AS013427 | contig_507 | phospholipid scramblase 2-like |
| AS013426 | contig_507 | phospholipid scramblase 2-like |
| AS000393 | contig_9 | calcium-transporting ATPase sarcoplasmic/endoplasmic reticulum type |
| AS006854 | contig_171 | roquin-1 isoform X2 |
| AS014516 | contig_607 | pollen-specific leucine-rich repeat extensin-like protein 1 |
| AS001904 | contig_45 | Uncharacterized protein OBRU01_26018 |
| AS001905 | contig_45 | hypothetical protein evm_011729 |
| AS001906 | contig_45 | calcium-sensing receptor |
| AS016516 | contig_1072 | speckle targeted PIP5K1A-regulated poly(A) polymerase |
| AS016517 | contig_1072 | tudor domain-containing protein 1/4/6/7 |
| AS017210 | contig_1449 | alpha-2-macroglobulin receptor-associated protein |
| AS017211 | contig_1449 | nkyrin repeat domain-containing protein 17 |
| AS001464 | contig_37 | hypothetical protein evm_011754 |
| AS001094 | contig_34 | hypothetical protein B5X24_HaOG203884 |
| AS001095 | contig_34 | hypothetical protein B5X24_HaOG213390 |
| AS001096 | contig_34 | DNA excision repair protein ERCC-3 |
| AS001097 | contig_34 | NA |
| AS001986 | contig_57 | zonadhesin |
| AS004297 | contig_129 | hypothetical protein B5X24_HaOG201630 |
| AS006971 | contig_174 | uncharacterized protein LOC108560498 |
| AS007690 | contig_202 | ATP-binding cassette sub-family G member 1 |
| AS007691 | contig_202 | polypeptide N-acetylgalactosaminyltransferase |
| AS006814 | contig_171 | solute carrier family 35 |
| AS006815 | contig_171 | uncharacterized protein LOC111360635 |
| AS013208 | contig_489 | ESF1 homolog |
| AS013207 | contig_489 | DNA (cytosine-5)-methyltransferase 1 |
| AS013209 | contig_489 | TP53-regulated inhibitor of apoptosis 1-like |
| AS013210 | contig_489 | transcription elongation factor SPT4 |
| AS013211 | contig_489 | NA |
| AS013896 | contig_561 | annulin isoform X2 |
| AS004980 | contig_140 | regulating synaptic membrane exocytosis protein |
| AS013428 | contig_507 | phospholipid scramblase 2-like |
| AS000962 | contig_34 | NA |
| AS000963 | contig_34 | hypothetical protein B5V51_6155 |
| AS000964 | contig_34 | hypothetical protein evm_012717 |
| AS006656 | contig_171 | hypothetical protein B5V51_11410 |
| AS006657 | contig_171 | NA |
| AS017627 | contig_1664 | alpha-(1,3)-fucosyltransferase C-like |
| AS007249 | contig_183 | prefoldin subunit 3 |
| AS007250 | contig_183 | U3 small nucleolar RNA-associated protein 20 |
| AS000518 | contig_25 | hypothetical protein B5V51_7957 |
| AS014086 | contig_567 | NA |
| AS014085 | contig_567 | MFS transporter |
| AS014087 | contig_567 | uncharacterized protein LOC111357747 |
| AS017242 | contig_1458 | NA |
| AS017243 | contig_1458 | hypothetical protein B5X24_HaOG209853 |
| AS013602 | contig_520 | WD repeat-containing protein 19 |
| AS013603 | contig_520 | arginyl-tRNA synthetase |
| AS015303 | contig_711 | NA |
| AS004084 | contig_115 | hemicentin-1-like |
| AS012009 | contig_345 | tachykinin-like peptides receptor |
| AS012010 | contig_345 | hypothetical protein AB894_15215 |
| AS010594 | contig_286 | enolase-phosphatase E1 |
| AS010595 | contig_286 | hypothetical protein B5V51_3014 |
| AS003559 | contig_96 | dynactin subunit 4 |
| AS003560 | contig_96 | oxygen-dependent protoporphyrinogen oxidase |
| AS003561 | contig_96 | histone arginine demethylase JMJD6 |
| AS009909 | contig_256 | tonsoku-like protein |
| AS008712 | contig_227 | eukaryotic translation initiation factor 5B |
| AS016537 | contig_1078 | NA |
| AS012836 | contig_448 | vegetative cell wall protein gp1-like |
| AS012837 | contig_448 | NA |
| AS012838 | contig_448 | voltage-dependent calcium channel L type alpha-1D |
| AS013291 | contig_493 | Mago nashi |
| AS017625 | contig_1664 | nuclear pore complex protein Nup53 |
| AS016029 | contig_917 | protein lin-9 |
| AS016030 | contig_917 | uncharacterized protein LOC111358470 |
| AS016031 | contig_917 | uncharacterized protein LOC110371831 |
| AS008714 | contig_227 | ATP-dependent RNA helicase SUPV3L1/SUV3 |
| AS008715 | contig_227 | hypothetical protein B5V51_10747 |
| AS008716 | contig_227 | transcriptional adapter 1 |
| AS013375 | contig_505 | hypothetical protein B5X24_HaOG207672 |
| AS013374 | contig_505 | JHBP domain-containing protein |
| AS015953 | contig_891 | plexin-B |
| AS015954 | contig_891 | plexin-B |
| AS015955 | contig_891 | abhydrolase domain-containing protein 2 |
| AS014648 | contig_629 | Ras-related and estrogen-regulated growth inhibitor |
| AS015014 | contig_654 | 60S ribosomal export protein NMD3 |
| AS015015 | contig_654 | ATP synthase subunit beta |
| AS015016 | contig_654 | 60S ribosomal protein L24 |
| AS015017 | contig_654 | protein FAM76A isoform X2 |
| AS010031 | contig_259 | probable uridine nucleosidase 2 |
| AS010032 | contig_259 | isocitrate dehydrogenase (NAD+) |
| AS002236 | contig_62 | F-box and WD-40 domain protein 4 |
| AS002237 | contig_62 | hypothetical protein B5V51_9330 |
| AS002238 | contig_62 | large subunit ribosomal protein L20 |
| AS002239 | contig_62 | E3 ubiquitin-protein ligase Arkadia |
| AS015023 | contig_654 | ATP-dependent RNA helicase DDX54 |
| AS015022 | contig_654 | tubulin-specific chaperone D |
| AS006441 | contig_171 | hypothetical protein B5X24_HaOG212193 |
| AS016277 | contig_969 | hypothetical protein B5V51_12982 |
| AS016278 | contig_969 | hypothetical protein B5V51_12983 |
| AS016279 | contig_969 | hypothetical protein B5V51_12984 |
| AS011756 | contig_338 | cerebellar degeneration-related protein 2-like |
| AS017655 | contig_1664 | multidrug resistance-associated protein 1-like |
| AS015010 | contig_654 | NEDD8-activating enzyme E1 catalytic subunit |
| AS015012 | contig_654 | hypothetical protein B5X24_HaOG206250 |
| AS015013 | contig_654 | ubiquinol-cytochrome-c reductase complex assembly factor 1 |
| AS015011 | contig_654 | uncharacterized protein LOC110374329 |
| AS010219 | contig_265 | ATP-binding cassette |
| AS013917 | contig_561 | hypothetical protein B5X24_HaOG203565 |
| AS004976 | contig_140 | F-box and leucine-rich repeat protein 6 |
| AS005803 | contig_159 | splicing factor 45 |
| AS011629 | contig_338 | hypothetical protein B5V51_1460 |
| AS011630 | contig_338 | serine hydrolase-like protein |
| AS011631 | contig_338 | fatty alcohol acetyltransferase |
| AS011632 | contig_338 | tRNA (guanine9-N1)-methyltransferase |
| AS011633 | contig_338 | argininosuccinate lyase |
| AS011628 | contig_338 | chitinase 10 isoform X1 |
| AS016515 | contig_1072 | speckle targeted PIP5K1A-regulated poly(A) polymerase |
| AS001623 | contig_40 | ecdysone-induced protein 74EF isoform X1 |
| AS014290 | contig_601 | twitchin |
| AS003146 | contig_82 | putative tyramine receptor 2 |
| AS002470 | contig_66 | leucine-rich repeat-containing G-protein coupled receptor 5 |
| AS003332 | contig_85 | gustatory receptor |
| AS003333 | contig_85 | hypothetical protein B5V51_10841 |
| AS003334 | contig_85 | glucose dehydrogenase [FAD, quinone]-like |
| AS003335 | contig_85 | glucose dehydrogenase [FAD, quinone]-like |
| AS004118 | contig_120 | hypothetical protein evm_002337 |
| AS007247 | contig_183 | hypothetical protein B5V51_5680 |
| AS014084 | contig_567 | major facilitator superfamily domain-containing protein 8 |
| AS018219 | contig_1907 | WASH complex subunit 1 |
| AS018221 | contig_1907 | hypothetical protein B5V51_3496 |
| AS018220 | contig_1907 | hypothetical protein B5V51_3495 |
| AS013290 | contig_493 | 40S ribosomal protein S8 |
| AS011192 | contig_325 | V-type H+-transporting ATPase subunit C |
| AS011193 | contig_325 | glycosyltransferase-like protein LARGE |
| AS017156 | contig_1400 | non-lysosomal glucosylceramidase |
| AS017157 | contig_1400 | uncharacterized protein LOC114352369 |
| AS017158 | contig_1400 | hypothetical protein B5V51_13622 |
| AS017159 | contig_1400 | T-cell activation inhibitor |
| AS003259 | contig_82 | myosin regulatory light chain sqh isoform X1 |
| AS003260 | contig_82 | glucose oxidase |
| AS001983 | contig_57 | unnamed protein product |
| AS001984 | contig_57 | large subunit ribosomal protein L48 |
| AS011889 | contig_343 | hypothetical protein B5V51_3376 |
| AS014687 | contig_629 | NA |
| AS006513 | contig_171 | DNA damage-responsive transcriptional repressor RPH1 |
| AS008127 | contig_208 | elongation of very long chain fatty acids protein 7 |
| AS016411 | contig_1036 | HEAT repeat-containing protein 6 |
| AS016412 | contig_1036 | hypothetical protein B5V51_8566 |
| AS008314 | contig_218 | plexin A3 |
| AS009133 | contig_239 | hypothetical protein B5V51_7039 |
| AS009134 | contig_239 | uncharacterized protein LOC110375917 |
| AS000870 | contig_31 | uncharacterized protein LOC111357099 |
| AS000871 | contig_31 | hypothetical protein B5V51_4688 |
| AS009749 | contig_251 | hypothetical protein B5V51_576 |
| AS009750 | contig_251 | WD repeat domain-containing protein 83 |
| AS009751 | contig_251 | pollen-specific leucine-rich repeat extensin-like protein 2 |
| AS002219 | contig_62 | histone acetyltransferase 1 |
| AS002220 | contig_62 | male-specific lethal 3 |
| AS002221 | contig_62 | larval cuticle protein A3A-like |
| AS001093 | contig_34 | uncharacterized protein LOC110379436 |
| AS006987 | contig_174 | glycerol kinase |
| AS003771 | contig_107 | 4-hydroxybenzoate polyprenyltransferase |
| AS016192 | contig_944 | uncharacterized protein LOC111353521 |
| AS016193 | contig_944 | myoneurin-like |
| AS010012 | contig_258 | segmentation polarity homeobox protein engrailed |
| AS011838 | contig_340 | odium-dependent nutrient amino acid transporter 1 |
| AS017932 | contig_1888 | hypothetical protein B5V51_3821 |
| AS011850 | contig_340 | hypothetical protein B5V51_5098 |
| AS001796 | contig_41 | reverse transcriptase |
| AS001797 | contig_41 | Gag-pol polyprotein |
| AS002005 | contig_58 | trithorax group protein osa |
| AS012032 | contig_345 | UDP-glucuronosyltransferase 2B31 |
| AS003246 | contig_82 | hypothetical protein B5X24_HaOG207426 |
| AS003247 | contig_82 | mevalonate kinase |
| AS011498 | contig_336 | programmed cell death protein 6 |
| AS011499 | contig_336 | uncharacterized protein LOC110375190 |
| AS011497 | contig_336 | dolichyl-phosphate beta-glucosyltransferase |
| AS000961 | contig_34 | uncharacterized protein LOC110380377 |
| AS002251 | contig_62 | cytochrome P450 314A1 |
| AS017548 | contig_1624 | insulin-like precursor polypeptide AA |
| AS008709 | contig_227 | hypothetical protein B5V51_3909 |
| AS003760 | contig_107 | uncharacterized protein LOC111361794 |
| AS013926 | contig_561 | ubiquitin carboxyl-terminal hydrolase CYLD |
| AS009703 | contig_251 | uncharacterized protein LOC110380130 |
| AS016048 | contig_919 | MFS transporter |
| AS013193 | contig_489 | MFS transporter |
| AS012028 | contig_345 | cytochrome P450 |
| AS012992 | contig_481 | CD63 antigen |
| AS018369 | contig_1938 | hypothetical protein B5V51_5093 |
| AS018370 | contig_1938 | uncharacterized protein LOC110376076 |
| AS015385 | contig_757 | DNA-directed RNA polymerase |
| AS010433 | contig_280 | dihydroorotate dehydrogenase |
| AS010434 | contig_280 | hexokinase |
| AS004418 | contig_134 | hypothetical protein B5V51_11547 |
| AS004419 | contig_134 | potassium channel subfamily K member 2-like |
| AS007342 | contig_185 | ABC transporter G family member 20 |
| AS000453 | contig_10 | ATP-binding cassette sub-family G member 4-like |
| AS008707 | contig_227 | phosphatidylinositol 4-phosphate 3-kinase C2 domain |
| AS013122 | contig_487 | hypothetical protein B5V51_12826 |
| AS013123 | contig_487 | meso-butanediol dehydrogenase / (S,S)-butanediol dehydrogenase / diacetyl reductase |
| AS013124 | contig_487 | meso-butanediol dehydrogenase / (S,S)-butanediol dehydrogenase / diacetyl reductase |
| AS013125 | contig_487 | hypothetical protein B5V51_12826 |
| AS013126 | contig_487 | hypothetical protein B5V51_1926 |
| AS013139 | contig_487 | PAN2-PAN3 deadenylation complex catalytic subunit PAN2 |
| AS004621 | contig_138 | NA |
| AS014779 | contig_633 | NA |
| AS014780 | contig_633 | NA |
| AS015952 | contig_891 | transformation/transcription domain-associated protein |
| AS006648 | contig_171 | Kip1 ubiquitination-promoting complex protein 1 |
| AS008877 | contig_235 | putative uncharacterized protein DDB_G0282129 |
| AS015340 | contig_757 | glyoxylate/hydroxypyruvate reductase |
| AS006231 | contig_169 | cytochrome P450 |
| AS015429 | contig_757 | uncharacterized protein LOC11135412 |
| AS015431 | contig_757 | hypothetical protein B5X24_HaOG205225 |
| AS015430 | contig_757 | pre-mRNA-processing factor 6 |
| AS015432 | contig_757 | carboxypeptidase B |
| AS009366 | contig_240 | actin-like protein 6B |
| AS009365 | contig_240 | cyclin-dependent kinase 2 |
| AS005409 | contig_153 | ceramide-1-phosphate transfer protein |
| AS008713 | contig_227 | uncharacterized protein LOC112046174 |
| AS005059 | contig_142 | hypothetical protein B5V51_8432 |
| AS005060 | contig_142 | H/ACA ribonucleoprotein complex non-core subunit NAF1 |
| AS017379 | contig_1535 | afadin |
| AS014712 | contig_629 | NA |
| AS014713 | contig_629 | hypothetical protein B5V51_5163 |
| AS002718 | contig_76 | NA |
| AS010482 | contig_284 | glucose dehydrogenase [FAD, quinone]-like |
| AS010483 | contig_284 | mannosyl-glycoprotein endo-beta-N-acetylglucosaminidase |
| AS014105 | contig_569 | 5-hydroxytryptamine receptor 4 |
| AS001028 | contig_34 | putative endonuclease/reverse transcriptase |
| AS001029 | contig_34 | reverse transcriptase |
| AS001030 | contig_34 | succinate dehydrogenase (ubiquinone) flavoprotein subunit |
| AS012035 | contig_345 | arylsulfatase B |
| AS001027 | contig_34 | uncharacterized protein LOC111363975 |
| AS001026 | contig_34 | NA |
| AS004707 | contig_138 | homeotic protein Sex combs reduced |
| AS013000 | contig_481 | ATP-binding cassette |
| AS011909 | contig_343 | uncharacterized protein LOC105561615 |
| AS002534 | contig_66 | unnamed protein product |
| AS014984 | contig_654 | timeless |
| AS015658 | contig_788 | NA |
| AS013192 | contig_489 | transcription initiation factor TFIIH subunit 2 |
| AS014088 | contig_567 | exocyst complex component 1 |
| AS015184 | contig_681 | UPF0489 protein C5orf22 |
| AS013630 | contig_523 | NA |
| AS006519 | contig_171 | 5'-AMP-activated protein kinase |
| AS012419 | contig_374 | ionotropic receptor 21a |
| AS012418 | contig_374 | myocyte-specific enhancer factor 2 |
| AS012420 | contig_374 | hypothetical protein B5X24_HaOG215908 |
| AS018294 | contig_1933 | NA |
| AS016891 | contig_1212 | hypothetical protein B5V51_3968 |
| AS012005 | contig_345 | tyrosine kinase receptor Cad96Ca |
| AS007882 | contig_204 | uncharacterized protein LOC111356386 |
| AS013165 | contig_489 | serine/arginine repetitive matrix protein 1 |
| AS008404 | contig_219 | hypothetical protein AB894_15215 |
| AS001594 | contig_40 | NA |
| AS018167 | contig_1899 | uncharacterized protein LOC110369699 |
| AS018168 | contig_1899 | solute carrier family 25 member 35-like isoform X2 |
| AS018169 | contig_1899 | solute carrier family 25 member 35-like isoform X1 |
| AS011761 | contig_338 | transcription initiation factor TFIID subunit 2 |
| AS001707 | contig_40 | neurogenic locus Notch protein isoform X2 |
| AS011412 | contig_333 | NADH dehydrogenase (ubiquinone) 1 beta subcomplex subunit 6 |
| AS011413 | contig_333 | 18S rRNA (adenine1779-N6/adenine1780-N6)-dimethyltransferase |
| AS011414 | contig_333 | transportin-1 |
| AS011411 | contig_333 | mitogen-activated protein kinase 14B |
| AS001834 | contig_45 | intraflagellar transport protein 46 |
| AS005844 | contig_159 | tau tubulin kinase |
| AS014963 | contig_652 | clavesin-1 |
| AS016652 | contig_1107 | NA |
| AS016651 | contig_1107 | uncharacterized protein LOC110370549 |
| AS001450 | contig_37 | FUN14 domain-containing protein 1 |
| AS011579 | contig_338 | toll-like receptor 13 |
| AS002599 | contig_70 | transcription initiation factor TFIID subunit 1 |
| AS013504 | contig_512 | atrial natriuretic peptide-converting enzyme |
| AS004082 | contig_115 | hemicentin-2 |
| AS013460 | contig_510 | NA |
| AS002976 | contig_82 | coronin-6 isoform X1 |
| AS008748 | contig_230 | adrenodoxin-like protein |
| AS008749 | contig_230 | zinc finger protein 62 |
| AS012301 | contig_372 | phytanoyl-CoA dioxygenase domain-containing protein 1 |
| AS012302 | contig_372 | NA |
| AS001292 | contig_35 | protein Skeletor, isoforms D/E |
| AS011136 | contig_323 | protein N-terminal glutamine amidohydrolase |
| AS011135 | contig_323 | methionyl aminopeptidase |
| AS001484 | contig_37 | KRAB domain-containing zinc finger protein |
| AS016276 | contig_969 | NA |
| AS014844 | contig_635 | apoptosis-resistant E3 ubiquitin protein ligase 1 |
| AS013879 | contig_557 | NA |
| AS013880 | contig_557 | NA |
| AS000034 | contig_1 | uncharacterized protein LOC105555876 |
| AS008496 | contig_219 | ubiquitin-like modifier-activating enzyme ATG7 |
| AS008497 | contig_219 | NA |
| AS015357 | contig_757 | zinc finger HIT domain-containing protein 1 |
| AS015358 | contig_757 | meiotic nuclear division protein 1 |
| AS015359 | contig_757 | exportin-5 |
| AS015360 | contig_757 | NA |
| AS015684 | contig_804 | NA |
| AS015685 | contig_804 | mannosyl-glycoprotein endo-beta-N-acetylglucosaminidase |
| AS008979 | contig_236 | protein anon-73B1 |
| AS008978 | contig_236 | protein suppressor of hairy wing |
| AS002184 | contig_58 | unnamed protein product |
| AS002183 | contig_58 | unnamed protein product |
| AS000121 | contig_4 | unnamed protein product |
| AS006186 | contig_168 | glutamate receptor ionotropic, kainate 3-like |
| AS014165 | contig_573 | endonuclease/reverse transcriptase |
| AS012765 | contig_447 | uncharacterized protein LOC111358149 |
| AS006329 | contig_169 | CDP-diacylglycerol--inositol 3-phosphatidyltransferase |
| AS006330 | contig_169 | T-complex protein 1 subunit zeta |
| AS006331 | contig_169 | protein unc-45 homolog B |
| AS016542 | contig_1078 | desaturase |
| AS001713 | contig_40 | uncharacterized protein LOC110379640 |
| AS002569 | contig_70 | NA |
| AS002570 | contig_70 | uncharacterized protein K02A2.6 |
| AS001981 | contig_57 | hypothetical protein B5V51_5309 |
| AS000578 | contig_26 | protein tramtrack |
| AS016572 | contig_1094 | defensin precursor |
| AS016571 | contig_1094 | alpha-methyldopa hypersensitive protein |
| AS006780 | contig_171 | uncharacterized protein LOC113521451 |
| AS004834 | contig_140 | PDZ domain-containing protein 8 |
| AS008027 | contig_207 | hypothetical protein B5V51_14725 |
| AS008028 | contig_207 | mucin-17 |
| AS006443 | contig_171 | NA |
| AS006444 | contig_171 | hypothetical protein B5V51_13180 |
| AS011910 | contig_343 | 43 kDa receptor-associated protein of the synapse homolog |
| AS009824 | contig_251 | phosphatidylserine decarboxylase |
| AS009823 | contig_251 | KRAB domain-containing zinc finger protein |
| AS009822 | contig_251 | cytoplasmic FMR1 interacting protein |
| AS006604 | contig_171 | prefoldin subunit 1 |
| AS006605 | contig_171 | ras-specific guanine nucleotide-releasing factor RalGPS2 |
| AS017639 | contig_1664 | solute carrier family 25 (mitochondrial carnitine/acylcarnitine transporter) |
| AS017640 | contig_1664 | kelch-like protein 10 |
| AS002974 | contig_82 | hypothetical protein B5V51_3466 |
| AS002975 | contig_82 | probable ATP-dependent RNA helicase DDX55 |
| AS002973 | contig_82 | ATP-dependent RNA helicase DDX55/SPB4 |
| AS007587 | contig_197 | Mesenchymal stem cell protein DSCD75 |
| AS007588 | contig_197 | carboxypeptidase E |
| AS010787 | contig_304 | carboxypeptidase A4 |
| AS013736 | contig_538 | 4-hydroxyphenylpyruvate dioxygenase |
| AS013737 | contig_538 | uncharacterized protein LOC114362327 |
| AS013738 | contig_538 | UDP-N-acetylglucosamine 1-carboxyvinyltransferase |
| AS010318 | contig_270 | DNA-directed RNA polymerase I subunit RPA49 |
| AS001858 | contig_45 | NA |
| AS006655 | contig_171 | uncharacterized protein LOC110374481 |
| AS006855 | contig_171 | dual oxidase maturation factor 1 |
| AS017549 | contig_1624 | insulin-like growth factor 2 |
| AS017550 | contig_1624 | tubulin alpha-8 chain-like |
| AS006485 | contig_171 | hypothetical protein B5V51_5679 |
| AS008206 | contig_216 | NA |
| AS011665 | contig_338 | fungal protease inhibitor-1 |
| AS011664 | contig_338 | fatty alcohol acetyltransferase |
| AS003703 | contig_101 | hypothetical protein B5X24_HaOG213176 |
| AS017995 | contig_1892 | hypothetical protein B5X24_HaOG212091 |
| AS006834 | contig_171 | hypothetical protein B5V51_9800 |
| AS011187 | contig_325 | hypothetical protein B5V51_10488 |
| AS015094 | contig_657 | protein peste-like isoform X1 |
| AS001771 | contig_41 | hypothetical protein B5V51_5885 |
| AS006659 | contig_171 | hypothetical protein B5X24_HaOG212193 |
| AS006658 | contig_171 | period circadian protein |
| AS014144 | contig_570 | hypothetical protein AB894_15215 |
| AS014145 | contig_570 | unnamed protein product |
| AS014146 | contig_570 | unnamed protein product |
| AS014147 | contig_570 | cellular nucleic acid-binding protein |
| AS009922 | contig_256 | uncharacterized protein LOC110371998 |
| AS000689 | contig_28 | hypothetical protein B5V51_1228 |
| AS000690 | contig_28 | 60S ribosomal protein L26 |
| AS000688 | contig_28 | proteoglycan 4-like |
| AS000919 | contig_31 | glycerol-3-phosphate acyltransferase 1 |
| AS005789 | contig_156 | hypothetical protein B5X24_HaOG202972 |
| AS013255 | contig_489 | trypsin 5G1-like |
| AS013256 | contig_489 | serine protease snake-like |
| AS008672 | contig_226 | longitudinals lacking protein, isoforms H/M/V-like |
| AS008673 | contig_226 | nuclear migration protein nudC |
| AS004474 | contig_134 | ankyrin repeat and LEM domain-containing protein 2 |
| AS017686 | contig_1665 | beta-1,3-galactosyltransferase 1 |
| AS011092 | contig_319 | dual oxidase maturation factor 1 |
| AS012360 | contig_374 | NA |
| AS009825 | contig_251 | bromodomain-containing protein 8 |
| AS017626 | contig_1664 | uncharacterized protein LOC111348605 |
| AS011609 | contig_338 | protein sidekick |
| AS001569 | contig_38 | tyrosine-protein phosphatase ERK |
| AS008296 | contig_218 | hypothetical protein B5V51_7055 |
| AS008297 | contig_218 | hypothetical protein B5V51_855 |
| AS005058 | contig_142 | fumarylacetoacetase |
| AS018439 | contig_1957 | solute carrier family 35 (adenosine 3'-phospho 5'-phosphosulfate transporter) |
| AS012505 | contig_379 | mitochondrial trans-2-enoyl-CoA reductase |
| AS012504 | contig_379 | uncharacterized protein LOC111353712 |
| AS010627 | contig_288 | carboxylesterase |
| AS008368 | contig_218 | hypothetical protein B5X24_HaOG213347 |
| AS008369 | contig_218 | NA |
| AS008370 | contig_218 | Niemann-Pick C2 protein |
| AS008556 | contig_219 | angiotensin-converting enzyme-like |
| AS005156 | contig_149 | uncharacterized protein LOC110384439 |
| AS005157 | contig_149 | VWFA and cache domain-containing protein CG16868 |
| AS015333 | contig_754 | uncharacterized protein LOC110383954 |
| AS005408 | contig_153 | optineurin |
| AS004393 | contig_132 | uncharacterized protein LOC110376338 |
| AS016518 | contig_1072 | uncharacterized protein LOC111355311 |
| AS016519 | contig_1072 | Iron/zinc purple acid phosphatase-like protein |
| AS004219 | contig_128 | AS004219 |
| AS003651 | contig_98 | ribonucleoside-diphosphate reductase subunit M2 |
| AS003653 | contig_98 | arrestin domain-containing protein 4 |
| AS003652 | contig_98 | COP9 signalosome complex subunit 2 |
| AS001298 | contig_36 | dentin sialophosphoprotein |
| AS000324 | contig_9 | NA |
| AS003576 | contig_96 | angiotensin-converting enzyme |
| AS003203 | contig_82 | hypothetical protein B5X24_HaOG203646 |
| AS003204 | contig_82 | uncharacterized protein LOC111360028 |
| AS008262 | contig_216 | inactive ubiquitin carboxyl-terminal hydrolase MINDY-4B |
| AS009664 | contig_250 | farnesyl diphosphate synthase |
| AS001617 | contig_40 | probable tRNA pseudouridine synthase 2 |
| AS001618 | contig_40 | PAXIP1-associated glutamate-rich protein 1A |
| AS001619 | contig_40 | hypothetical protein B5V51_10738 |
| AS001621 | contig_40 | uncharacterized protein LOC111355191 |
| AS001622 | contig_40 | vesicle transport protein SEC22 |
| AS001620 | contig_40 | nudC domain-containing protein 1 |
| AS002723 | contig_76 | nuclear pore complex protein Nup53 |
| AS018131 | contig_1899 | cytochrome b5 domain-containing protein 1 |
| AS018132 | contig_1899 | RWD domain-containing protein 4 |
| AS018133 | contig_1899 | serine/threonine-protein kinase Aurora-2 |
| AS018134 | contig_1899 | ionotropic receptor 68a |
| AS007089 | contig_174 | melatonin-related receptor-like |
| AS002941 | contig_82 | prophenoloxidase 1 |
| AS005284 | contig_152 | insulin gene enhancer protein ISL-1 |
| AS015122 | contig_662 | dynein heavy chain 1 |
| AS003319 | contig_85 | dynein heavy chain 3, axonemal |
| AS009086 | contig_238 | Ig-like and fibronectin type-III domain-containing protein 2 |
| AS003385 | contig_86 | solute carrier family 4 (anion exchanger) |
| AS015853 | contig_843 | SWI/SNF-related matrix-associated actin-dependent regulator of chromatin subfamily A-like protein 1 |
| AS001427 | contig_37 | ethanolamine phosphotransferase |
| AS011188 | contig_325 | trypsin |
| AS006493 | contig_171 | L-galactose dehydrogenase |
| AS015598 | contig_783 | uncharacterized protein LOC111359810 |
| AS008757 | contig_231 | cGMP-dependent protein kinase 1 |
| AS013835 | contig_550 | NA |
| AS013836 | contig_550 | hypothetical protein evm_013750 |
| AS011911 | contig_343 | CUE domain-containing protein 2 |
| AS005552 | contig_153 | uncharacterized protein LOC111365097 |
| AS005553 | contig_153 | hypothetical protein B5V51_2312 |
| AS017994 | contig_1892 | hypothetical protein B5V51_11764 |
| AS002852 | contig_80 | gamma-aminobutyric acid type B receptor |
| AS002853 | contig_80 | ATP-binding cassette |
| AS016275 | contig_969 | NA |
| AS002049 | contig_58 | putative odorant receptor |
| AS008118 | contig_208 | fatty alcohol acetyltransferase |
| AS000734 | contig_29 | carnosine N-methyltransferase |
| AS000735 | contig_29 | probable serine/threonine-protein kinase DDB_G0278901 |
| AS003258 | contig_82 | dnaJ homolog subfamily C member 30-like |
| AS003257 | contig_82 | arginine/serine-rich coiled-coil protein 2 |
| AS002218 | contig_62 | E3 ubiquitin-protein ligase KCMF1-like |
| AS003140 | contig_82 | mannose receptor |
| AS003141 | contig_82 | fructose-bisphosphate aldolase |
| AS013138 | contig_487 | hypothetical protein B5X24_HaOG211169 |
| AS014143 | contig_570 | hypothetical protein B5V51_14228 |
| AS003287 | contig_82 | uncharacterized protein LOC111360097 |
| AS006983 | contig_174 | glutamate receptor 1 |
| AS006225 | contig_169 | MICOS complex subunit mic60-like |
| AS002419 | contig_66 | Down syndrome cell adhesion molecule-like protein Dscam2 |
| AS000500 | contig_12 | phosphofurin acidic cluster sorting protein 1 |
| AS012993 | contig_481 | CD63 antigen |
| AS014594 | contig_627 | hypothetical protein B5V51_254 |
| AS014595 | contig_627 | hypothetical protein B5V51_253 |
| AS012501 | contig_379 | cytochrome b5 reductase 4 |
| AS001507 | contig_37 | orexin receptor type 1-like |
| AS010331 | contig_270 | uncharacterized protein LOC113507525 |
| AS010332 | contig_270 | NA |
| AS010333 | contig_270 | nucleoprotein |
| AS011983 | contig_345 | uncharacterized protein LOC110378016 |
| AS001833 | contig_45 | transforming acidic coiled-coil-containing protein 3 |
| AS006955 | contig_174 | NA |
| AS006956 | contig_174 | NA |
| AS008325 | contig_218 | insulin-like receptor |
| AS005061 | contig_142 | transcription factor GAGA-like |
| AS005062 | contig_142 | inositol-3-phosphate synthase |
| AS013545 | contig_512 | transmembrane protein 205 |
| AS006547 | contig_171 | nuclear receptor subfamily 2 group E member 1 |
| AS006548 | contig_171 | MFS transporter |
| AS006860 | contig_171 | PHD finger protein rhinoceros |
| AS006224 | contig_169 | cellular tumor antigen p53-like |
| AS004026 | contig_114 | NA |
| AS004027 | contig_114 | bombyxin A-1 |
| AS007186 | contig_175 | ethanolamine kinase |
| AS005142 | contig_149 | optic atrophy protein 1 |
| AS013401 | contig_507 | obscurin isoform X3 |
| AS002054 | contig_58 | Dynein heavy chain 3, axonema |
| AS006404 | contig_171 | NA |
| AS006405 | contig_171 | NA |
| AS006646 | contig_171 | sulfhydryl oxidase 1 |
| AS009002 | contig_237 | peroxidase |
| AS016726 | contig_1110 | hypothetical protein B5V51_8040 |
| AS010320 | contig_270 | proteasome subunit alpha type-6 |
| AS010321 | contig_270 | unnamed protein product |
| AS010322 | contig_270 | ras-related protein Rab-10 |
| AS001609 | contig_40 | phenoloxidase-activating factor 1 |
| AS006398 | contig_171 | sarcosine oxidase / L-pipecolate oxidase |
| AS006399 | contig_171 | NA |
| AS014850 | contig_635 | DDB1- and CUL4-associated factor 7 |
| AS014851 | contig_635 | NA |
| AS007035 | contig_174 | unnamed protein product |
| AS007034 | contig_174 | protein NDRG3 |
| AS005281 | contig_152 | endoplasmic reticulum metallopeptidase 1 |
| AS017648 | contig_1664 | NA |
| AS009535 | contig_248 | Retrotransposable element |
| AS008581 | contig_221 | hypoxia-inducible factor prolyl hydroxylase |
| AS008582 | contig_221 | papilin-like isoform X2 |
| AS013891 | contig_557 | lachesin-like isoform X2 |
| AS016437 | contig_1036 | ubiquitin carboxyl-terminal hydrolase BAP1 |
| AS016438 | contig_1036 | clustered mitochondria protein homolog |
| AS008811 | contig_235 | digestive cysteine proteinase 2 |
| AS016047 | contig_919 | AS016047 |
| AS015498 | contig_772 | uncharacterized protein LOC110384255 |
| AS015499 | contig_772 | T-complex protein 1 subunit gamma |
| AS000227 | contig_6 | uncharacterized protein LOC110379783 |
| AS000229 | contig_6 | protein arginine methyltransferase NDUFAF7 homolog |
| AS000228 | contig_6 | RNA-binding protein 28-like |
| AS011973 | contig_345 | aminopeptidase N |
| AS011974 | contig_345 | aminopeptidase N-like |
| AS007177 | contig_175 | ER membrane protein complex subunit 10 |
| AS007178 | contig_175 | DEAD-box helicase Dbp80 |
| AS007179 | contig_175 | myo-inositol 2-dehydrogenase / D-chiro-inositol 1-dehydrogenase |
| AS003484 | contig_94 | beta-catenin-like protein 1 |
| AS003485 | contig_94 | NA |
| AS003486 | contig_94 | hydroxyacid oxidase 1 |
| AS013649 | contig_523 | periodic tryptophan protein 1 |
| AS013650 | contig_523 | hypothetical protein B5X24_HaOG201578 |
| AS003628 | contig_98 | proteasome subunit beta type-2-like |

Notes: NA (not applicable).

**Table S10. Genes of XJ selected region genes identified by CLR analysis.**

| **Gene ID** | **Contig** | **Annotation** |
| --- | --- | --- |
| AS006804 | contig_171 | tyrosine-protein phosphatase non-receptor type 2 |
| AS004980 | contig_140 | regulating synaptic membrane exocytosis protein 1 |
| AS001976 | contig_57 | hrp65 protein-like |
| AS001977 | contig_57 | uncharacterized protein KIAA1143 homolog |
| AS001978 | contig_57 | transforming growth factor beta regulator 1 |
| AS016537 | contig_1078 | NA |
| AS006656 | contig_171 | coatomer subunit gamma |
| AS006657 | contig_171 | NA |
| AS006737 | contig_171 | craniofacial development protein 2 |
| AS006738 | contig_171 | endonuclease-reverse transcriptase |
| AS010432 | contig_280 | Hermansky-Pudlak syndrome 1 protein |
| AS010433 | contig_280 | dihydroorotate dehydrogenase |
| AS010434 | contig_280 | hexokinase type 2 |
| AS016538 | contig_1078 | desaturase |
| AS006957 | contig_174 | glucose transporter type 1 |
| AS008412 | contig_219 | reverse transcriptase |
| AS011783 | contig_339 | NA |
| AS006492 | contig_171 | galactosylgalactosylxylosylprotein 3-beta-glucuronosyltransferase S |
| AS006384 | contig_169 | trichoplein keratin filament-binding protein |
| AS006503 | contig_171 | protein SERAC1-like |
| AS006504 | contig_171 | uncharacterized protein LOC108758204 |
| AS006854 | contig_171 | roquin-1 isoform X2 |
| AS011594 | contig_338 | NADH dehydrogenase [ubiquinone] 1 beta subcomplex subunit 11 |
| AS011595 | contig_338 | uncharacterized protein LOC111351645 |
| AS011596 | contig_338 | extensin |
| AS004248 | contig_129 | oligoribonuclease |
| AS004249 | contig_129 | LETM1 and EF-hand domain-containing protein 1 |
| AS016099 | contig_932 | golgin subfamily A member 2 |
| AS006877 | contig_174 | zinc finger protein 107-like |
| AS001979 | contig_57 | menin |
| AS001980 | contig_57 | NA |
| AS000324 | contig_9 | NA |
| AS004979 | contig_140 | host cell factor |
| AS016533 | contig_1078 | hypothetical protein B5V51_2740 |
| AS007097 | contig_174 | ynein heavy chain 5, axonemal isoform X2 |
| AS008250 | contig_216 | NA |
| AS017634 | contig_1664 | nuclear pore complex protein Nup53 |
| AS008403 | contig_219 | egmentation protein even-skipped-like |
| AS006442 | contig_171 | NA |
| AS007057 | contig_174 | serine/threonine-protein kinase PRP4 |
| AS015130 | contig_662 | stress-induced-phosphoprotein 1 |
| AS014361 | contig_603 | ypothetical protein B5V51_10874 |
| AS015023 | contig_654 | ATP-dependent RNA helicase DDX54 |
| AS015022 | contig_654 | tubulin-specific chaperone D |
| AS006148 | contig_167 | transcriptional coactivator YAP1 |
| AS010431 | contig_280 | BRISC complex subunit FAM175B-like |
| AS014188 | contig_573 | uncharacterized protein LOC111348420 |
| AS014189 | contig_573 | electron transfer flavoprotein regulatory factor 1 |
| AS014190 | contig_573 | synaptotagmin 1 |
| AS001310 | contig_36 | AMP deaminase 2 |
| AS004634 | contig_138 | mediator of RNA polymerase II transcription subunit 4 |
| AS004635 | contig_138 | putative inner dynein arm light chain |
| AS006435 | contig_171 | uncharacterized protein LOC110375138 |
| AS006434 | contig_171 | succinate dehydrogenase [ubiquinone] flavoprotein subunit |
| AS008249 | contig_216 | three prime repair exonuclease 2 |
| AS007691 | contig_202 | polypeptide N-acetylgalactosaminyltransferase |
| AS009301 | contig_240 | synaptic vesicle glycoprotein 2B |
| AS007523 | contig_196 | endocuticle structural glycoprotein ABD-4-like |
| AS017893 | contig_1887 | laminin subunit alpha-1-like |
| AS001314 | contig_36 | thyroid transcription factor 1-like |
| AS012576 | contig_414 | cytochrome P450 |
| AS015342 | contig_757 | Niemann-Pick C1 protein |
| AS003509 | contig_94 | multidrug resistance protein 1-like |
| AS000394 | contig_9 | protein PRR14L |
| AS000395 | contig_9 | membrane-associated progesterone receptor component |
| AS017632 | contig_1664 | tyrosine-protein kinase transmembrane receptor Ror |
| AS017633 | contig_1664 | hypothetical protein B5V51_3105 |
| AS018365 | contig_1938 | nuclear pore complex protein Nup107 |
| AS018366 | contig_1938 | DDRGK domain-containing protein 1-like |
| AS018367 | contig_1938 | transmembrane emp24 domain-containing protein eca |
| AS012903 | contig_481 | unnamed protein product |
| AS004555 | contig_138 | malate dehydrogenase (oxaloacetate-decarboxylating) (NADP+) |
| AS009750 | contig_251 | WD repeat domain-containing protein 83 |
| AS009751 | contig_251 | pollen-specific leucine-rich repeat extensin-like protein 2 |
| AS010397 | contig_274 | mitochondrial ribosomal protein L54 |
| AS010399 | contig_274 | synaptic vesicular amine transporter-like |
| AS010400 | contig_274 | MFS transporter |
| AS010398 | contig_274 | outer dense fiber protein 3-like |
| AS016111 | contig_932 | intermediate filament protein if |
| AS016112 | contig_932 | AP-3 complex subunit mu |
| AS016113 | contig_932 | hypothetical protein B5V51_3066 |
| AS013290 | contig_493 | 40S ribosomal protein S8 |
| AS013291 | contig_493 | Mago nashi |
| AS012241 | contig_363 | uncharacterized protein LOC111359797 |
| AS004740 | contig_138 | NA |
| AS009108 | contig_238 | fatty acid amide hydrolase 2 |
| AS009107 | contig_238 | S-adenosylmethionine decarboxylase proenzyme |
| AS003408 | contig_92 | trafficking protein particle complex subunit 10 |
| AS008467 | contig_219 | unnamed protein product |
| AS014187 | contig_573 | signal-induced proliferation-associated 1-like protein 1 |
| AS008900 | contig_236 | cAMP-specific phosphodiesterase 4 |
| AS001718 | contig_40 | uncharacterized protein LOC111357008 |
| AS010988 | contig_315 | U6 snRNA-associated Sm-like protein LSm5 |
| AS010989 | contig_315 | Targeting protein for Xklp2-B |
| AS010987 | contig_315 | targeting protein for Xklp2 |
| AS014706 | contig_629 | gamma-tubulin complex component 2 |
| AS014707 | contig_629 | dynamin 1-like protein |
| AS014899 | contig_635 | protein FAM49A |
| AS006147 | contig_167 | transcriptional coactivator YAP1 |
| AS006146 | contig_167 | gastrula zinc finger protein XlCGF46.1-like |
| AS017474 | contig_1573 | transient receptor potential cation channel subfamily A member 1 |
| AS003263 | contig_82 | centromere protein J |
| AS003264 | contig_82 | nardilysin |
| AS014252 | contig_577 | uncharacterized protein LOC110383798 |
| AS010306 | contig_269 | uncharacterized protein LOC116765719 |
| AS010307 | contig_269 | uncharacterized protein LOC111349470 |
| AS013102 | contig_486 | zinc finger and SCAN domain-containing protein 21 |
| AS013103 | contig_486 | tyrosyl-tRNA synthetase |
| AS008717 | contig_227 | large subunit ribosomal protein L27 |
| AS001575 | contig_38 | activating signal cointegrator complex subunit 3 |
| AS010701 | contig_288 | GATOR complex protein WDR24 |
| AS010702 | contig_288 | ubiquitin carboxyl-terminal hydrolase 30 |
| AS005533 | contig_153 | hypothetical protein B5V51_9932 |
| AS005532 | contig_153 | inhibitor of Bruton tyrosine kinase |
| AS009243 | contig_240 | activin receptor type-2 |
| AS009244 | contig_240 | eukaryotic translation initiation factor 4E type 2 |
| AS017631 | contig_1664 | hypothetical protein B5V51_3103 |
| AS017630 | contig_1664 | beta-1-syntrophin |
| AS000696 | contig_28 | A-type muscarinic acetylcholine receptor |
| AS015083 | contig_656 | CUGBP Elav-like family member 4 |
| AS011529 | contig_336 | optic atrophy protein 1 |
| AS011528 | contig_336 | transient receptor potential-gamma protein-like |
| AS014631 | contig_629 | heat shock protein beta-1 |
| AS012766 | contig_447 | succinate dehydrogenase (ubiquinone) flavoprotein subunit |
| AS016743 | contig_1110 | mediator of RNA polymerase II transcription subunit 15 |
| AS016744 | contig_1110 | tripartite motif-containing protein 45 |
| AS008559 | contig_219 | collagen |
| AS012321 | contig_372 | glycine cleavage system H protein |
| AS012323 | contig_372 | protein henna |
| AS012322 | contig_372 | ATP synthase subunit gamma, mitochondrial-like isoform X1 |
| AS012176 | contig_359 | Ca2+ transporting ATPase |
| AS012177 | contig_359 | NA |
| AS006284 | contig_169 | PAX-interacting protein 1 |
| AS007284 | contig_185 | homeobox protein Hmx-like |
| AS003771 | contig_107 | 4-hydroxybenzoate polyprenyltransferase |
| AS007526 | contig_196 | endocuticle structural glycoprotein SgAbd-2 |
| AS009212 | contig_240 | NA |
| AS007572 | contig_197 | NA |
| AS014858 | contig_635 | NA |
| AS003841 | contig_107 | catenin alpha |
| AS010594 | contig_286 | enolase-phosphatase E1 |
| AS010595 | contig_286 | hypothetical protein B5V51_3014 |
| AS005648 | contig_154 | gamma-interferon-inducible lysosomal thiol reductase-like |
| AS005649 | contig_154 | splicing factor 45 |
| AS005650 | contig_154 | serine/threonine-protein kinase greatwall |
| AS011105 | contig_319 | nuclear pore complex protein Nup133 |
| AS011106 | contig_319 | NA |
| AS011104 | contig_319 | structural maintenance of chromosome 2 |
| AS000382 | contig_9 | troponin C-like |
| AS014652 | contig_629 | bestrophin 1a |
| AS001987 | contig_57 | hypothetical protein B5V51_5319 |
| AS001988 | contig_57 | ABC transporter C family member 13 |
| AS018040 | contig_1893 | potassium channel subfamily K member 9 |
| AS014054 | contig_566 | nicotinic acetylcholine receptor alpha-5 |
| AS014053 | contig_566 | DNA-binding protein D-ETS-6 |
| AS014055 | contig_566 | ionotropic receptor 75d |
| AS004633 | contig_138 | NA |
| AS013035 | contig_484 | ubiquitin carboxyl-terminal hydrolase 7 |
| AS000030 | contig_1 | uncharacterized protein LOC110374347 |
| AS013181 | contig_489 | UPF0454 protein C12orf49 |
| AS018023 | contig_1892 | beta 1,4-N-acetylgalactosaminyl transferase |
| AS001981 | contig_57 | hypothetical protein B5V51_5309 |
| AS004467 | contig_134 | NA |
| AS001451 | contig_37 | uncharacterized protein LOC111351052 |
| AS000268 | contig_9 | NA |
| AS000267 | contig_9 | rotatin |
| AS015122 | contig_662 | dynein heavy chain 1 |
| AS007244 | contig_183 | uncharacterized protein LOC113505275 |
| AS009938 | contig_256 | collagen alpha-1(I) chain |
| AS016598 | contig_1102 | unnamed protein product |
| AS004069 | contig_115 | hypothetical protein B5V51_954 |
| AS007524 | contig_196 | uncharacterized protein LOC111349498 |
| AS007525 | contig_196 | neurofilament heavy polypeptide |
| AS010482 | contig_284 | glucose dehydrogenase [FAD, quinone] |
| AS010483 | contig_284 | mannosyl-glycoprotein endo-beta-N-acetylglucosaminidase |
| AS001543 | contig_37 | gustatory receptor |
| AS001544 | contig_37 | hypothetical protein B5V51_10841 |
| AS001545 | contig_37 | leech-derived tryptase inhibitor C-like |
| AS000637 | contig_28 | putative nuclease HARBI1 |
| AS000636 | contig_28 | hypothetical protein B5V51_4845 |
| AS013969 | contig_561 | cysteine/serine-rich nuclear protein |
| AS008556 | contig_219 | angiotensin-converting enzyme-like |
| AS013609 | contig_520 | TNF receptor-associated factor 6 |
| AS009435 | contig_244 | RING finger protein nhl-1 |
| AS000359 | contig_9 | hypothetical protein B5V51_6433 |
| AS000360 | contig_9 | uncharacterized protein LOC110372559 |
| AS000361 | contig_9 | succinate dehydrogenase (ubiquinone) flavoprotein subunit |
| AS000362 | contig_9 | unnamed protein product |
| AS002504 | contig_66 | uncharacterized protein LOC111364797 |
| AS009752 | contig_251 | cytoplasmic polyadenylation element-binding protein 2 |
| AS004841 | contig_140 | GTPase-activating Rap/Ran-GAP domain-like protein 3 |
| AS006423 | contig_171 | paraplegin |
| AS000449 | contig_9 | carboxypeptidase A |
| AS000448 | contig_9 | ntegrin alpha-9-like |
| AS012120 | contig_353 | sorting nexin lst-4 |
| AS012121 | contig_353 | CD151 antigen-like |
| AS003962 | contig_113 | WD and tetratricopeptide repeats protein 1 |
| AS005355 | contig_152 | glucuronosyltransferase |
| AS008273 | contig_216 | NA |
| AS006785 | contig_171 | NA |
| AS004940 | contig_140 | gamma-aminobutyric acid type B receptor |
| AS006678 | contig_171 | Retrotransposable element |
| AS006677 | contig_171 | ubiquitin carboxyl-terminal hydrolase 35/38 |
| AS006679 | contig_171 | uncharacterized protein LOC110374495 |
| AS006383 | contig_169 | LIM/homeobox protein Lhx1 |
| AS013432 | contig_507 | RNA binding protein fox-1 |
| AS016761 | contig_1123 | uncharacterized protein LOC110376155 |
| AS016762 | contig_1123 | lipase member H-like |
| AS007095 | contig_174 | NA |
| AS002720 | contig_76 | gustatory receptor |
| AS008693 | contig_227 | DNA-directed RNA polymerase II subunit RPB1 |
| AS009191 | contig_239 | zinc finger protein 420-like |
| AS009192 | contig_239 | DNA mismatch repair protein MLH1 |
| AS008869 | contig_235 | hypothetical protein B5X24_HaOG203199 |
| AS008868 | contig_235 | uncharacterized protein LOC110376637 |
| AS012240 | contig_363 | sulfide:quinone oxidoreductase |
| AS002534 | contig_66 | unnamed protein product |
| AS007238 | contig_183 | F-type H+-transporting ATPase subunit O |
| AS009348 | contig_240 | hypothetical protein B5X24_HaOG205602 |
| AS013851 | contig_550 | probable sodium-coupled neutral amino acid transporter 6 |
| AS009761 | contig_251 | zinc finger protein 91 |
| AS009760 | contig_251 | zinc finger protein 808 |
| AS004987 | contig_140 | saccharopine dehydrogenase-like oxidoreductase |
| AS006548 | contig_171 | MFS transporter |
| AS006838 | contig_171 | NA |
| AS006837 | contig_171 | transmembrane channel-like protein 2 |
| AS010693 | contig_288 | uncharacterized protein LOC110383482 |
| AS010691 | contig_288 | structural maintenance of chromosomes protein 1A |
| AS010692 | contig_288 | transmembrane protein 222 |
| AS017139 | contig_1374 | E3 ubiquitin-protein ligase TRIM9 |
| AS003291 | contig_82 | uncharacterized protein LOC110370955 |
| AS003292 | contig_82 | uncharacterized protein LOC110370955 |
| AS016660 | contig_1107 | uncharacterized protein LOC111348276 |
| AS017379 | contig_1535 | afadin |
| AS012394 | contig_374 | hypothetical protein B5V51_11986 |
| AS014073 | contig_566 | protein borderless-like |
| AS011838 | contig_340 | sodium-dependent nutrient amino acid transporter 1-like |
| AS010165 | contig_264 | programmed cell death protein |
| AS010164 | contig_264 | lipase 1-like |
| AS009837 | contig_251 | hypothetical protein B5V51_9091 |
| AS009561 | contig_250 | NA |
| AS009562 | contig_250 | odorant binding protein |
| AS009563 | contig_250 | NA |
| AS009564 | contig_250 | eukaryotic translation elongation factor 1 epsilon-1 |
| AS002083 | contig_58 | uncharacterized protein LOC111348971 |
| AS018439 | contig_1957 | solute carrier family 35 (adenosine 3'-phospho 5'-phosphosulfate transporter) |
| AS014251 | contig_577 | hypothetical protein EVAR_24085_1 |
| AS006052 | contig_162 | F-box and WD-40 domain protein 1/11 |
| AS015916 | contig_891 | argininosuccinate synthase |
| AS014615 | contig_629 | protein bric-a-brac 1 |
| AS013454 | contig_508 | NA |
| AS013453 | contig_508 | NA |
| AS005179 | contig_151 | G protein-coupled receptor Mth |
| AS004250 | contig_129 | uncharacterized protein LOC113505268 |
| AS009459 | contig_244 | myosin heavy chain 6/7 |
| AS011507 | contig_336 | hypothetical protein B5V51_6245 |
| AS011210 | contig_325 | SWI/SNF-related matrix-associated actin-dependent regulator of chromatin subfamily A-like protein 1 |
| AS015031 | contig_654 | DNA-directed RNA polymerase II subunit RPB1 |
| AS009488 | contig_245 | phosphatidylinositol glycan |
| AS012448 | contig_377 | hypothetical protein B5X24_HaOG214372 |
| AS012447 | contig_377 | tyrosine-protein phosphatase non-receptor type 23 |
| AS014071 | contig_566 | ATP-binding cassette |
| AS014072 | contig_566 | hypothetical protein B5V51_12815 |
| AS002355 | contig_65 | protein transport protein SEC61 subunit alpha |
| AS002356 | contig_65 | midnolin homolog |
| AS008823 | contig_235 | NA |
| AS008409 | contig_219 | diacylglycerol kinase (ATP) |
| AS011669 | contig_338 | trafficking kinesin-binding protein milt |
| AS000320 | contig_9 | proton channel OtopLc |
| AS000321 | contig_9 | NA |
| AS000081 | contig_4 | sorting nexin-19 |
| AS009622 | contig_250 | diuretic hormone receptor |
| AS009560 | contig_250 | eukaryotic translation elongation factor 1 epsilon-1 |
| AS006404 | contig_171 | NA |
| AS011612 | contig_338 | hypothetical protein B5V51_1451 |
| AS005705 | contig_156 | NA |
| AS011625 | contig_338 | uncharacterized protein LOC110370010 |
| AS011626 | contig_338 | caspase 7 |
| AS011627 | contig_338 | uncharacterized protein LOC110370008 |
| AS010591 | contig_286 | intron-binding protein aquarius |
| AS009937 | contig_256 | succinate dehydrogenase (ubiquinone) flavoprotein subunit |
| AS012765 | contig_447 | uncharacterized protein LOC111358149 |
| AS011466 | contig_336 | hypothetical protein B5V51_9932 |
| AS008707 | contig_227 | phosphatidylinositol 4-phosphate 3-kinase C2 domain |
| AS013162 | contig_488 | beta-catenin-like protein 1 |
| AS003476 | contig_94 | wingless-type MMTV integration site family |
| AS003477 | contig_94 | UDP-glucose:glycoprotein glucosyltransferase |
| AS002696 | contig_74 | hypothetical protein B5X24_HaOG214938 |
| AS007651 | contig_201 | zinc finger protein 585A-like |
| AS014002 | contig_566 | WD and tetratricopeptide repeats protein 1 |
| AS018443 | contig_1972 | hypothetical protein B5V51_3838 |
| AS018442 | contig_1972 | ecdysone-responsive G-protein coupled protein-2 |
| AS007908 | contig_204 | xanthine dehydrogenase 1-like |
| AS000167 | contig_4 | succinate dehydrogenase (ubiquinone) flavoprotein subunit |
| AS000715 | contig_29 | hormone receptor 3 |
| AS013693 | contig_530 | protein FAM161A |
| AS013694 | contig_530 | protein unc-79 homolog |
| AS014793 | contig_633 | uncharacterized protein LOC110381489 |
| AS003040 | contig_82 | very-long-chain 3-oxoacyl-CoA reductase |
| AS002725 | contig_76 | unnamed protein product |
| AS002724 | contig_76 | unnamed protein product |
| AS011789 | contig_339 | histidine-rich glycoprotein |
| AS011790 | contig_339 | histidine-rich glycoprotein |
| AS011791 | contig_339 | piggyBac transposable element-derived protein 3 |
| AS013076 | contig_486 | uncharacterized protein LOC111359425 |
| AS007151 | contig_175 | unnamed protein product |
| AS003152 | contig_82 | nose resistant to fluoxetine protein 6 |
| AS006810 | contig_171 | titin homolog |
| AS009702 | contig_251 | histidine triad (HIT) family protein |
| AS005803 | contig_159 | splicing factor 45 |
| AS005804 | contig_159 | hypothetical protein B5V51_9231 |
| AS006455 | contig_171 | vacuolar protein sorting-associated protein 13B |
| AS005692 | contig_154 | hypothetical protein B5V51_1538 |
| AS005693 | contig_154 | snurportin-1 |
| AS013508 | contig_512 | NA |
| AS005253 | contig_151 | neurofilament heavy polypeptide-like |
| AS005254 | contig_151 | unnamed protein product |
| AS005255 | contig_151 | serine/threonine-protein kinase S6KL |
| AS004925 | contig_140 | alcohol oxidase 2 |
| AS007734 | contig_203 | NA |
| AS005305 | contig_152 | reverse transcriptase |
| AS007242 | contig_183 | uncharacterized protein LOC111357700 |
| AS002566 | contig_66 | unnamed protein product |
| AS003312 | contig_82 | probable 18S rRNA (guanine-N(7))-methyltransferase |
| AS003313 | contig_82 | hypothetical protein B5V51_4132 |
| AS003311 | contig_82 | DNA replication ATP-dependent helicase Dna2 |
| AS001413 | contig_37 | tRNA (cytosine34-C5)-methyltransferase |
| AS017551 | contig_1624 | hypothetical protein B5V51_11193 |
| AS014646 | contig_629 | DENN domain-containing protein 5 |
| AS014483 | contig_607 | polypeptide N-acetylgalactosaminyltransferase 3 |
| AS003956 | contig_113 | fanconi-associated nuclease 1 |
| AS011314 | contig_327 | hypothetical protein B5X24_HaOG212154 |
| AS002029 | contig_58 | UDP-glucuronosyltransferase 2B1 |
| AS013601 | contig_520 | SH3 and multiple ankyrin repeat domains protein 3 |
| AS004480 | contig_134 | uncharacterized protein LOC111354380 |
| AS004481 | contig_134 | serine/threonine-protein phosphatase 6 regulatory ankyrin repeat subunit B |
| AS009985 | contig_258 | NA |
| AS009986 | contig_258 | putative adenosylhomocysteinase 3 |
| AS014096 | contig_567 | unnamed protein product |
| AS014097 | contig_567 | NA |
| AS000944 | contig_31 | facilitated trehalose transporter Tret1-like |
| AS000943 | contig_31 | facilitated trehalose transporter Tret1-like |
| AS013104 | contig_486 | rho GTPase-activating protein 44 |
| AS011801 | contig_340 | MFS transporter |
| AS011750 | contig_338 | U3 small nucleolar RNA-associated protein 7 |
| AS011751 | contig_338 | transcription factor E2F4/5 |
| AS011582 | contig_338 | splicing factor 3B subunit 1 |
| AS004523 | contig_135 | fatty alcohol acetyltransferase |
| AS004524 | contig_135 | acetyl-CoA acyltransferase 2 |
| AS007710 | contig_203 | NA |
| AS014738 | contig_633 | uncharacterized protein LOC113505179 |
| AS002182 | contig_58 | uncharacterized protein LOC111349066 |
| AS000154 | contig_4 | NA |
| AS009565 | contig_250 | NA |
| AS009566 | contig_250 | odorant binding protein |
| AS008921 | contig_236 | uncharacterized protein LOC116770084 |
| AS008920 | contig_236 | luciferin 4-monooxygenase |
| AS003538 | contig_96 | gustatory receptor |
| AS004760 | contig_139 | ribosomal protein S6 kinase alpha-5 |
| AS011782 | contig_339 | protein HIRA/HIR1 |
| AS005269 | contig_151 | sarcolemmal membrane-associated protein |
| AS006472 | contig_171 | protein furry |
| AS006473 | contig_171 | somatostatin receptor type 4-like |
| AS011709 | contig_338 | pleckstrin homology domain containing family A member 8 |
| AS011708 | contig_338 | DNA polymerase delta catalytic subunit |
| AS002636 | contig_71 | dishevelled associated activator of morphogenesis |
| AS004660 | contig_138 | autophagy-related protein 16 |
| AS018004 | contig_1892 | uncharacterized protein LOC116771415 |
| AS018003 | contig_1892 | probable histone-binding protein Caf1 |
| AS011670 | contig_338 | DNA primase small subunit |
| AS011671 | contig_338 | peroxidase |
| AS014515 | contig_607 | putative neuropeptide precursor protein |
| AS002562 | contig_66 | unnamed protein product |
| AS016070 | contig_919 | IKAROS family zinc finger protein |
| AS016071 | contig_919 | ransmembrane protein 184C |
| AS001341 | contig_36 | exosome complex component RRP46 |
| AS001342 | contig_36 | NA |
| AS001340 | contig_36 | hypothetical protein B5V51_2647 |
| AS010253 | contig_268 | NA |
| AS015780 | contig_831 | uncharacterized protein LOC113493037 |
| AS015781 | contig_831 | GTP-binding nuclear protein Ran |
| AS012695 | contig_446 | metabotropic glutamate receptor 2-like |
| AS000263 | contig_9 | kinesin family member C1 |
| AS000264 | contig_9 | inositol-trisphosphate 3-kinase homolog |
| AS002751 | contig_78 | sodium/potassium-transporting ATPase subunit |
| AS002750 | contig_78 | uncharacterized protein LOC105380449 |
| AS015154 | contig_677 | NA |
| AS015155 | contig_677 | NA |
| AS015156 | contig_677 | NA |
| AS015153 | contig_677 | progestin and adipoQ receptor family member 3 |
| AS011048 | contig_319 | hemicentin-2 |
| AS012566 | contig_414 | uncharacterized protein LOC110380777 |
| AS012567 | contig_414 | antigen KI-67 |
| AS012565 | contig_414 | nucleoplasmin-like protein |
| AS014293 | contig_601 | NA |
| AS008870 | contig_235 | secretory phospholipase A2 |
| AS005434 | contig_153 | aldehyde dehydrogenase (NAD+) |
| AS011971 | contig_345 | muscarinic acetylcholine receptor M5 |
| AS001431 | contig_37 | caspase 7 |
| AS006871 | contig_174 | NA |
| AS006872 | contig_174 | NA |
| AS008719 | contig_227 | NA |
| AS008718 | contig_227 | intermediate filament protein if |
| AS004761 | contig_139 | uncharacterized protein LOC108758204 |
| AS013951 | contig_561 | F-box protein 42 |
| AS013952 | contig_561 | pre-mRNA-splicing factor SYF1 |
| AS013950 | contig_561 | unnamed protein product |
| AS000785 | contig_29 | mothers against decapentaplegic homolog 2/3 |
| AS000078 | contig_4 | TNF receptor-associated factor 1 |
| AS011074 | contig_319 | receptor-type tyrosine-protein phosphatase gamma |
| AS011075 | contig_319 | probable methyltransferase-like protein 15 homolog |
| AS011073 | contig_319 | protein preli-like |
| AS000472 | contig_10 | limbic system-associated membrane protein-like |
| AS005332 | contig_152 | NA |
| AS005333 | contig_152 | serine protease 7-like |
| AS014897 | contig_635 | hypothetical protein B5V51_6569 |
| AS012309 | contig_372 | E3 ubiquitin-protein ligase MYCBP2 |
| AS002455 | contig_66 | uncharacterized protein LOC111351402 |
| AS010294 | contig_269 | collagen |
| AS008461 | contig_219 | uncharacterized protein LOC113508911 |
| AS014487 | contig_607 | NA |
| AS007159 | contig_175 | branched-chain amino acid aminotransferase |
| AS007160 | contig_175 | succinate dehydrogenase assembly factor 2-A |
| AS010742 | contig_293 | brachyurin-like |
| AS013252 | contig_489 | disintegrin and metalloproteinase domain-containing protein 10 |
| AS013253 | contig_489 | uncharacterized protein LOC113235851 |
| AS003379 | contig_86 | DDB1- and CUL4-associated factor 15 |
| AS003378 | contig_86 | nuclear pore complex protein Nup53 |
| AS002373 | contig_65 | ATP-binding cassette sub-family G member 8 |
| AS010425 | contig_280 | O-palmitoleoyl-L-serine hydrolase |
| AS010424 | contig_280 | PI-PLC X domain-containing protein 3 |
| AS003787 | contig_107 | nucleolysin TIA-1 |
| AS012980 | contig_481 | ADP-ribosylation factor-related protein 1 |
| AS012979 | contig_481 | uncharacterized protein LOC110372733 |
| AS016293 | contig_969 | uncharacterized protein LOC114350010 |
| AS015304 | contig_711 | facilitated trehalose transporter Tret1-2 |
| AS012794 | contig_448 | apolipophorins |
| AS005139 | contig_149 | DnaJ homolog subfamily C member 10 |
| AS014040 | contig_566 | NF-kappa-B inhibitor cactus-like |
| AS017005 | contig_1285 | dmX-like protein 2 |
| AS005854 | contig_159 | 26S proteasome non-ATPase regulatory subunit 13 |
| AS013606 | contig_520 | GDP-fucose transporter 1 |
| AS013607 | contig_520 | iduronate 2-sulfatase |
| AS013605 | contig_520 | hypothetical protein B5V51_11135 |
| AS011304 | contig_327 | protein FAM8A1 |
| AS011303 | contig_327 | ras-related protein Rab6 |
| AS005088 | contig_142 | uncharacterized protein LOC110380526 |
| AS010901 | contig_308 | hypothetical protein AB894_15215 |
| AS016838 | contig_1203 | hypothetical protein B5V51_12871 |
| AS010900 | contig_308 | urin-like protease 2 |
| AS010645 | contig_288 | solute carrier family 35 (adenosine 3'-phospho 5'-phosphosulfate transporter) |
| AS010646 | contig_288 | thyroid stimulating hormone receptor |
| AS003876 | contig_107 | NA |
| AS003877 | contig_107 | NA |
| AS009267 | contig_240 | transcription elongation factor B |
| AS009266 | contig_240 | zinc finger protein 582-like |
| AS009268 | contig_240 | rotein Peter pan |
| AS006520 | contig_171 | prostatic acid phosphatase |
| AS010499 | contig_284 | alpha-1,6-mannosyl-glycoprotein 2-beta-N-acetylglucosaminyltransferase-like |
| AS001800 | contig_41 | NA |
| AS002341 | contig_65 | glutamate synthase [NADH] |
| AS006396 | contig_171 | coiled-coil domain-containing protein AGAP005037 |

Notes: NA (not applicable).

**Table S11. Genes of STC selected region genes identified by CLR analysis.**

| **Gene ID** | **Contig** | **Annotation** |
| --- | --- | --- |
| AS003293 | contig_82 | uncharacterized protein LOC111360097 |
| AS013371 | contig_505 | odorant binding protein |
| AS003291 | contig_82 | NA |
| AS003292 | contig_82 | uncharacterized protein LOC110370955 |
| AS003290 | contig_82 | uncharacterized protein LOC110370955 |
| AS006403 | contig_171 | basement membrane-specific heparan sulfate proteoglycan core protein |
| AS003284 | contig_82 | glucose dehydrogenase [FAD, quinone]-like |
| AS011494 | contig_336 | cubilin-like |
| AS016538 | contig_1078 | desaturase |
| AS003285 | contig_82 | pupal cuticle protein |
| AS003294 | contig_82 | NA |
| AS007853 | contig_204 | uncharacterized protein LOC111356548 |
| AS007854 | contig_204 | probable aldehyde oxidase gad-3 |
| AS007855 | contig_204 | probable aldehyde oxidase gad-3 |
| AS013951 | contig_561 | F-box only protein 42 |
| AS013952 | contig_561 | pre-mRNA-splicing factor SYF1 |
| AS013950 | contig_561 | unnamed protein product |
| AS013953 | contig_561 | zinc finger CCCH domain-containing protein 13 |
| AS011774 | contig_339 | DNA repair endonuclease XPF |
| AS011775 | contig_339 | exportin-1 |
| AS001992 | contig_57 | transmembrane protein 177 |
| AS001991 | contig_57 | condensin-2 complex subunit D3 |
| AS013372 | contig_505 | odorant binding protein |
| AS017613 | contig_1664 | thyrostimulin beta-5 subunit-like |
| AS003283 | contig_82 | NA |
| AS015823 | contig_842 | uncharacterized protein LOC114249218 |
| AS001098 | contig_34 | serine/threonine-protein kinase MRCK |
| AS011495 | contig_336 | cubilin |
| AS001096 | contig_34 | DNA excision repair protein ERCC-3 |
| AS001097 | contig_34 | NA |
| AS001981 | contig_57 | hypothetical protein B5V51_5309 |
| AS013370 | contig_505 | odorant binding protein |
| AS009599 | contig_250 | uncharacterized protein LOC111349709 |
| AS017405 | contig_1549 | hemicentin-2-like |
| AS017404 | contig_1549 | cytochrome p450 CYP6AB14 |
| AS011035 | contig_319 | estrogen-related receptor ERR |
| AS004481 | contig_134 | serine/threonine-protein phosphatase 6 regulatory ankyrin repeat subunit B |
| AS011772 | contig_339 | protein-L-isoaspartate(D-aspartate) O-methyltransferase |
| AS013693 | contig_530 | protein FAM161A |
| AS011651 | contig_338 | integrin beta-8-like |
| AS011653 | contig_338 | MOB kinase activator-like 4 |
| AS011652 | contig_338 | ribonuclease P protein subunit p25-like protein |
| AS000460 | contig_10 | protein retinal degeneration B |
| AS000461 | contig_10 | NA |
| AS018339 | contig_1938 | NA |
| AS003286 | contig_82 | uncharacterized protein LOC110370955 |
| AS017624 | contig_1664 | pro-resilin isoform X1 |
| AS017625 | contig_1664 | nuclear pore complex protein Nup53 |
| AS012141 | contig_353 | teneurin-m isoform X5 |
| AS016253 | contig_957 | alkaline phosphatase |
| AS003999 | contig_114 | carbohydrate 6-sulfotransferase 6 |
| AS004000 | contig_114 | carbohydrate sulfotransferase 4 |
| AS007096 | contig_174 | Arf-GAP with Rho-GAP domain |
| AS006231 | contig_169 | cytochrome P450 |
| AS017615 | contig_1664 | hypothetical protein B5V51_2691 |
| AS017616 | contig_1664 | uncharacterized protein LOC110384379 |
| AS013291 | contig_493 | Mago nashi |
| AS002341 | contig_65 | glutamate synthase [NADH] |
| AS006792 | contig_171 | gamma-aminobutyric acid receptor subunit beta-like |
| AS010862 | contig_308 | hypothetical protein B5V51_5482 |
| AS010863 | contig_308 | hypothetical protein B5V51_5482 |
| AS010861 | contig_308 | ejaculatory bulb-specific protein 3-like |
| AS004085 | contig_115 | hemicentin-1-like |
| AS009488 | contig_245 | phosphatidylinositol glycan |
| AS014175 | contig_573 | ATP synthase subunit delta |
| AS014176 | contig_573 | Down syndrome cell adhesion molecule |
| AS003282 | contig_82 | choline dehydrogenase |
| AS018171 | contig_1899 | protein HIRA homolog |
| AS004541 | contig_138 | suppressor of lurcher protein 1 |
| AS010864 | contig_308 | synaptic vesicle glycoprotein 2B-like |
| AS004480 | contig_134 | uncharacterized protein LOC111354380 |
| AS007129 | contig_175 | adenylate cyclase type 9 |
| AS013203 | contig_489 | serine protease persephone-like |
| AS015387 | contig_757 | hypothetical protein B5V51_12048 |
| AS011486 | contig_336 | RNA-directed DNA polymerase |
| AS012269 | contig_364 | uncharacterized protein LOC110383465 |
| AS012270 | contig_364 | hypothetical protein B5V51_10673 |
| AS006926 | contig_174 | uncharacterized protein LOC111360253 |
| AS002141 | contig_58 | calcineurin-binding protein cabin-1 |
| AS002143 | contig_58 | transmembrane protease serine 11D |
| AS002142 | contig_58 | protein unc-50 homolog |
| AS001966 | contig_57 | intermediate filament protein if |
| AS001965 | contig_57 | protein HIRA/HIR1 |
| AS003507 | contig_94 | NA |
| AS003508 | contig_94 | ATP-dependent RNA helicase bel |
| AS004482 | contig_134 | uncharacterized protein LOC110374158 |
| AS004483 | contig_134 | NA |
| AS007130 | contig_175 | hypothetical protein evm_013249 |
| AS001094 | contig_34 | hypothetical protein B5X24_HaOG203884 |
| AS001095 | contig_34 | hypothetical protein B5X24_HaOG213390 |
| AS010140 | contig_263 | acetylcholine receptor subunit alpha-like |
| AS010139 | contig_263 | acetylcholine receptor subunit alpha-like isoform X3 |
| AS014015 | contig_566 | ATP-binding cassette sub-family G member 4 |
| AS006232 | contig_169 | unnamed protein product |
| AS016543 | contig_1078 | glutamate receptor 2 |
| AS001511 | contig_37 | hypothetical protein AB894_15215 |
| AS001512 | contig_37 | RNA binding protein fox-1 |
| AS017857 | contig_1880 | protein sister of odd and bowel-like |
| AS008942 | contig_236 | popeye domain-containing protein 3-like |
| AS008943 | contig_236 | probable cation-transporting ATPase 13A3 |
| AS017626 | contig_1664 | uncharacterized protein LOC111348605 |
| AS003287 | contig_82 | uncharacterized protein LOC111360097 |
| AS006971 | contig_174 | NA |
| AS014388 | contig_603 | unnamed protein product |
| AS014389 | contig_603 | glycogen debranching enzyme |
| AS003559 | contig_96 | dynactin subunit 4 |
| AS003560 | contig_96 | oxygen-dependent protoporphyrinogen oxidase |
| AS003561 | contig_96 | histone arginine demethylase JMJD6 |
| AS003771 | contig_107 | 4-hydroxybenzoate polyprenyltransferase |
| AS014682 | contig_629 | hypothetical protein B5V51_15015 |
| AS014683 | contig_629 | mediator of RNA polymerase II transcription subunit 7 |
| AS015976 | contig_900 | probable isocitrate dehydrogenase subunit alpha |
| AS006645 | contig_171 | solute carrier family 15 (oligopeptide transporter) |
| AS006958 | contig_174 | tubulin alpha-1 chain-like |
| AS011771 | contig_339 | male-enhanced antigen 1 |
| AS011770 | contig_339 | serpin B8-like |
| AS016986 | contig_1276 | AiC5 chymotrypsinogen |
| AS016987 | contig_1276 | AiC5 chymotrypsinogen |
| AS009301 | contig_240 | synaptic vesicle glycoprotein 2B-like |
| AS006405 | contig_171 | NA |
| AS016314 | contig_972 | leukocyte receptor cluster member 1 homolog |
| AS016313 | contig_972 | WASH complex subunit 5 |
| AS009601 | contig_250 | prickle |
| AS009602 | contig_250 | hypothetical protein B5V51_8723 |
| AS012927 | contig_481 | DnaJ homolog subfamily C member 16 |
| AS014317 | contig_601 | Delta24-sterol reductase |
| AS018140 | contig_1899 | protein KIAA0100 |
| AS000715 | contig_29 | hormone receptor 3 |
| AS004942 | contig_140 | hypothetical protein B5V51_4400 |
| AS001509 | contig_37 | uncharacterized protein LOC111355311 |
| AS001510 | contig_37 | NA |
| AS006936 | contig_174 | uncharacterized protein LOC110376556 |
| AS006937 | contig_174 | U-box domain-containing protein 5 |
| AS014645 | contig_629 | hypothetical protein B5V51_12603 |
| AS011594 | contig_338 | NADH dehydrogenase [ubiquinone] 1 beta subcomplex subunit 11 |
| AS011595 | contig_338 | uncharacterized protein LOC111351645 |
| AS011596 | contig_338 | extensin |
| AS009090 | contig_238 | craniofacial development protein 2-like |
| AS008456 | contig_219 | hypothetical protein B5V51_9294 |
| AS007098 | contig_174 | ADP/ATP translocase 1-like |
| AS006129 | contig_167 | eukaryotic translation initiation factor 3 subunit E |
| AS006130 | contig_167 | 39S ribosomal protein L53 |
| AS006131 | contig_167 | protein argonaute-2 isoform X2 |
| AS004335 | contig_129 | uncharacterized protein LOC111357516 |
| AS009086 | contig_238 | Ig-like and fibronectin type-III domain-containing protein 2 |
| AS006821 | contig_171 | hypothetical protein B5X24_HaOG204140 |
| AS006920 | contig_174 | OTU domain-containing protein 3 |
| AS014482 | contig_607 | ATP-dependent RNA helicase DHX36 |
| AS013996 | contig_566 | hypothetical protein AB894_15215 |
| AS015099 | contig_657 | hypothetical protein B5V51_1064 |
| AS015100 | contig_657 | NA |
| AS018236 | contig_1908 | histone deacetylase complex subunit SAP30 |
| AS018235 | contig_1908 | high mobility group protein B1 |
| AS018237 | contig_1908 | exosome complex component RRP45 |
| AS005849 | contig_159 | copper-transporting ATPase 1 |
| AS002048 | contig_58 | putative odorant receptor |
| AS009091 | contig_238 | uncharacterized protein LOC110382069 |
| AS004315 | contig_129 | acyl-CoA delta 11 desaturase |
| AS004866 | contig_140 | 1-acyl-sn-glycerol-3-phosphate acyltransferase alpha |
| AS004867 | contig_140 | 60S ribosomal protein L5 |
| AS004868 | contig_140 | carboxylesterase type B |
| AS006790 | contig_171 | uncharacterized protein LOC111355311 |
| AS013416 | contig_507 | hairy/enhancer-of-split related with YRPW motif protein |
| AS013434 | contig_508 | hypothetical protein B5V51_11412 |
| AS006183 | contig_168 | hypothetical protein B5V51_11074 |
| AS006182 | contig_168 | hypothetical protein B5V51_11075 |
| AS009356 | contig_240 | NA |
| AS009358 | contig_240 | cytochrome c oxidase subunit NDUFA4 |
| AS009355 | contig_240 | splicing factor 1 |
| AS009357 | contig_240 | MOB kinase activator-like 1 |
| AS005017 | contig_140 | uncharacterized protein LOC115447671 |
| AS005018 | contig_140 | hypothetical protein B5X24_HaOG201306 |
| AS004660 | contig_138 | autophagy-related protein 16 |
| AS013204 | contig_489 | NFX1-type zinc finger-containing protein 1-like |
| AS018340 | contig_1938 | NA |
| AS017641 | contig_1664 | alpha-aminoadipic semialdehyde synthase |
| AS004978 | contig_140 | rab-like protein 6 isoform X1 |
| AS004979 | contig_140 | host cell factor |
| AS005031 | contig_140 | fumarylacetoacetase |
| AS005030 | contig_140 | probable multidrug resistance-associated protein |
| AS013954 | contig_561 | SET domain-containing protein SmydA-8-like |
| AS013955 | contig_561 | protein msta-like |
| AS013956 | contig_561 | protein msta-like isoform X1 |
| AS003140 | contig_82 | mannose receptor |
| AS003141 | contig_82 | fructose-bisphosphate aldolase |
| AS001591 | contig_40 | NA |
| AS012695 | contig_446 | metabotropic glutamate receptor 2 |
| AS014471 | contig_607 | hypothetical protein RR48_10692 |
| AS014472 | contig_607 | 3-oxoacyl- reductase |
| AS014473 | contig_607 | serine/threonine-protein kinase fray2-like |
| AS001451 | contig_37 | uncharacterized protein LOC111351052 |
| AS001430 | contig_37 | caspase 7 |
| AS001431 | contig_37 | caspase-1-B-like |
| AS004758 | contig_139 | ribosomal protein S6 kinase alpha-5 |
| AS004757 | contig_139 | CCAAT/enhancer-binding protein zeta |
| AS018353 | contig_1938 | probable methylthioribulose-1-phosphate dehydratase |
| AS018354 | contig_1938 | zinc finger protein 345-like |
| AS002407 | contig_65 | proteasomal ATPase-associated factor 1 |
| AS010081 | contig_261 | DNA excision repair protein ERCC-3 |
| AS010080 | contig_261 | nuclear pore complex protein Nup53 |
| AS018040 | contig_1893 | potassium channel subfamily K member 9-like |
| AS012021 | contig_345 | UDP-glycosyltransferase 33T3 |
| AS011654 | contig_338 | hypothetical protein B5V51_14542 |
| AS011655 | contig_338 | corepressor interacting with RBPJ 1 |
| AS009089 | contig_238 | uncharacterized protein LOC110377889 |
| AS007024 | contig_174 | uncharacterized protein LOC111361095 |
| AS002526 | contig_66 | F-box/LRR-repeat protein 14 |
| AS013511 | contig_512 | actin-binding LIM protein |
| AS013510 | contig_512 | peroxidase |
| AS012476 | contig_378 | NA |
| AS015824 | contig_842 | counting factor associated protein D-like |
| AS018032 | contig_1893 | NA |
| AS018033 | contig_1893 | rho GTPase-activating protein 190 |
| AS012309 | contig_372 | E3 ubiquitin-protein ligase MYCBP2 |
| AS001771 | contig_41 | hypothetical protein B5V51_5885 |
| AS001772 | contig_41 | putative oxidoreductase GLYR1 homolog |
| AS003281 | contig_82 | condensin complex subunit 3 |
| AS001314 | contig_36 | thyroid transcription factor 1-like |
| AS006810 | contig_171 | titin homolog |
| AS011825 | contig_340 | uncharacterized protein LOC115450773 |
| AS011826 | contig_340 | hypothetical protein B5X24_HaOG209551 |
| AS001710 | contig_40 | neuropathy target esterase sws |
| AS008471 | contig_219 | glutamate decarboxylase |
| AS015564 | contig_774 | pre-mRNA-splicing factor ATP-dependent RNA helicase DHX16 |
| AS015565 | contig_774 | WW domain-containing oxidoreductase-like |
| AS015342 | contig_757 | Niemann-Pick C1 protein |
| AS002047 | contig_58 | NA |
| AS001894 | contig_45 | retinol-binding protein 4 |
| AS012020 | contig_345 | uncharacterized protein LOC106142263 |
| AS007906 | contig_204 | xanthine dehydrogenase-like |
| AS004014 | contig_114 | helicase POLQ-like |
| AS002763 | contig_78 | anaphase-promoting complex subunit 2 |
| AS002762 | contig_78 | ubiquitin-associated and SH3 domain-containing protein |
| AS002764 | contig_78 | probable Rho GTPase-activating protein CG5521 |
| AS013097 | contig_486 | chloride channel 7 |
| AS013098 | contig_486 | hypothetical protein B5V51_12672 |
| AS013096 | contig_486 | unnamed protein product |
| AS010425 | contig_280 | O-palmitoleoyl-L-serine hydrolase |
| AS010424 | contig_280 | PI-PLC X domain-containing protein 3-like |
| AS003378 | contig_86 | nuclear pore complex protein Nup53 |
| AS004057 | contig_114 | gastrin/cholecystokinin type B receptor |
| AS014960 | contig_652 | NA |
| AS013796 | contig_550 | Retrovirus-related Pol polyprotein from transposon 17.6 |
| AS013797 | contig_550 | NA |
| AS013798 | contig_550 | hypothetical protein EVAR_98954_1 |
| AS002904 | contig_81 | voltage-dependent L-type calcium channel subunit beta-3 |
| AS003770 | contig_107 | U6 snRNA-associated Sm-like protein LSm4 |
| AS013486 | contig_510 | tryptophan 5-monooxygenase |
| AS013487 | contig_510 | TBC1 domain family member 24 |
| AS010025 | contig_259 | D(2) dopamine receptor |
| AS011593 | contig_338 | sodium- and chloride-dependent GABA transporter ine |
| AS016805 | contig_1186 | lipase 3-like |
| AS015997 | contig_901 | 40S ribosomal protein SA |
| AS015998 | contig_901 | protein enhancer of sevenless 2B |
| AS015334 | contig_754 | zinc finger MIZ domain-containing protein 1 |
| AS014509 | contig_607 | integrin beta 1 |
| AS006397 | contig_171 | NA |
| AS000245 | contig_6 | ecto-NOX disulfide-thiol exchanger 2-like |
| AS015952 | contig_891 | transformation/transcription domain-associated protein |
| AS013294 | contig_496 | hypothetical protein B5V51_10841 |
| AS016052 | contig_919 | mucin-12-like |
| AS008273 | contig_216 | NA |
| AS006054 | contig_162 | Methyltransferase-like protein 17 |
| AS007907 | contig_204 | ubiquitin-like modifier-activating enzyme ATG7 |
| AS008409 | contig_219 | diacylglycerol kinase (ATP) |
| AS011683 | contig_338 | dynein heavy chain |
| AS016602 | contig_1102 | dynein intermediate chain 2 |
| AS017197 | contig_1449 | heterogeneous nuclear ribonucleoprotein Q |
| AS017198 | contig_1449 | cubilin |
| AS005527 | contig_153 | hypothetical protein B5V51_3405 |
| AS005529 | contig_153 | actin cytoskeleton-regulatory complex protein PAN1-like |
| AS005530 | contig_153 | NA |
| AS005531 | contig_153 | uncharacterized protein LOC110371252 |
| AS005528 | contig_153 | NA |
| AS004973 | contig_140 | pre-60S factor REI1 |
| AS004338 | contig_129 | JHBP domain-containing protein |
| AS004555 | contig_138 | malate dehydrogenase (oxaloacetate-decarboxylating)(NADP+) |
| AS003075 | contig_82 | NA |
| AS009535 | contig_248 | Retrotransposable element |
| AS013076 | contig_486 | uncharacterized protein LOC111359425 |
| AS003359 | contig_85 | methylenetetrahydrofolate dehydrogenase (NADP+) / methenyltetrahydrofolate cyclohydrolase / formyltetrahydrofolate synthetase |
| AS003360 | contig_85 | mannose receptor |
| AS007083 | contig_174 | erythrocyte band 7 integral membrane protein |
| AS007082 | contig_174 | uncharacterized protein LOC110373003 |
| AS017842 | contig_1879 | NA |
| AS013208 | contig_489 | ESF1 homolog |
| AS013207 | contig_489 | DNA (cytosine-5)-methyltransferase PliMCI-like |
| AS013293 | contig_496 | gustatory receptor |
| AS014231 | contig_577 | homeobox domain-containing protein |
| AS002801 | contig_78 | hemicentin-1-like |
| AS001713 | contig_40 | uncharacterized protein LOC110379640 |
| AS013139 | contig_487 | PAN2-PAN3 deadenylation complex catalytic subunit PAN2 |
| AS004336 | contig_129 | hypothetical protein B5V51_2404 |
| AS008262 | contig_216 | inactive ubiquitin carboxyl-terminal hydrolase MINDY-4B |
| AS001718 | contig_40 | uncharacterized protein LOC111357008 |
| AS001504 | contig_37 | fanconi-associated nuclease 1 |
| AS001505 | contig_37 | acetylcholinesterase |
| AS013427 | contig_507 | phospholipid scramblase 2-like |
| AS013426 | contig_507 | phospholipid scramblase 2-like |
| AS006688 | contig_171 | multidrug resistance protein homolog 49-like |
| AS015978 | contig_900 | NA |
| AS015977 | contig_900 | U3 small nucleolar RNA-associated protein 22 |
| AS014017 | contig_566 | NA |
| AS002371 | contig_65 | ATP-binding cassette |
| AS006193 | contig_169 | MFS transporter |
| AS001374 | contig_37 | acilitated trehalose transporter Tret1-like |
| AS008855 | contig_235 | pancreatic lipase-related protein 2-like |
| AS008856 | contig_235 | pancreatic triacylglycerol lipase-like |
| AS001858 | contig_45 | NA |
| AS001859 | contig_45 | eukaryotic translation initiation factor 2-alpha kinase 4 |
| AS003778 | contig_107 | phosphatidic acid-selective&#160phospholipase A1 ; |
| AS003779 | contig_107 | pancreatic lipase 2 |
| AS004971 | contig_140 | Arginase-2, mitochondrial |
| AS004972 | contig_140 | uncharacterized protein LOC110370583 |
| AS006588 | contig_171 | hypothetical protein B5X24_HaOG204786 |
| AS011801 | contig_340 | MFS transporter |
| AS009430 | contig_244 | diapausin B1 |
| AS009431 | contig_244 | 5-hydroxytryptamine receptor |
| AS013401 | contig_507 | obscurin isoform X3 |
| AS006614 | contig_171 | eukaryotic translation initiation factor 4 gamma 3 |
| AS009604 | contig_250 | NA |
| AS003754 | contig_107 | protein LLP homolog |
| AS003755 | contig_107 | uncharacterized protein LOC110380282 |
| AS003756 | contig_107 | NA |
| AS003757 | contig_107 | zinc finger protein 64 homolog |
| AS003753 | contig_107 | uncharacterized protein LOC110380267 |
| AS012408 | contig_374 | RNA-directed DNA polymerase |
| AS012409 | contig_374 | craniofacial development protein 2-like |
| AS012967 | contig_481 | NA |
| AS012968 | contig_481 | 23 kDa integral membrane protein-like |
| AS014279 | contig_601 | heat shock protein 19.8 |
| AS014280 | contig_601 | NA |
| AS014278 | contig_601 | uncharacterized protein LOC110373912 |
| AS000273 | contig_9 | kinase D-interacting substrate of 220 kDa |
| AS000422 | contig_9 | caspase 8 |
| AS000423 | contig_9 | hypothetical protein evm_014177 |
| AS000424 | contig_9 | probable peptide chain release factor C12orf65 homolog |
| AS000425 | contig_9 | hypothetical protein B5V51_1 |
| AS014169 | contig_573 | 5'-AMP-activated protein kinase subunit beta-1 |
| AS007243 | contig_183 | NA |
| AS009594 | contig_250 | NA |
| AS009595 | contig_250 | uncharacterized protein LOC110376362 |
| AS009596 | contig_250 | hypothetical protein B5X24_HaOG202765 |
| AS009597 | contig_250 | unnamed protein product |
| AS015150 | contig_677 | tyrosine-protein phosphatase non-receptor type 13 |
| AS006716 | contig_171 | loricrin-like |
| AS002992 | contig_82 | NA |
| AS002991 | contig_82 | uncharacterized protein LOC110373660 |
| AS016687 | contig_1108 | inhibin beta chain |
| AS005355 | contig_152 | glucuronosyltransferase |
| AS001680 | contig_40 | uncharacterized protein LOC110374640 |
| AS006973 | contig_174 | biogenesis of lysosome-related organelles complex 1 |
| AS006974 | contig_174 | NA |
| AS005850 | contig_159 | NA |
| AS005851 | contig_159 | NA |
| AS012268 | contig_364 | ubiquitin-conjugating enzyme E2 S |
| AS006028 | contig_162 | MFS transporter |
| AS013943 | contig_561 | LIM domain-containing protein jub |
| AS013942 | contig_561 | phosphatidylinositol glycan |
| AS015137 | contig_662 | regulator of nonsense transcripts 1-like |
| AS015136 | contig_662 | arrestin domain-containing protein 17 |
| AS011714 | contig_338 | GTP binding protein |
| AS016218 | contig_944 | tyrosine-protein kinase shark |
| AS014217 | contig_577 | pancreatic triacylglycerol lipase-like |
| AS014218 | contig_577 | pancreatic lipase-related protein 2 |
| AS004628 | contig_138 | tachykinin-like receptor |
| AS003823 | contig_107 | NA |
| AS003822 | contig_107 | protein toll |
| AS000668 | contig_28 | carboxylesterase 8 |
| AS016988 | contig_1276 | AiC5 chymotrypsinogen |
| AS003962 | contig_113 | WD and tetratricopeptide repeats protein 1 |
| AS016140 | contig_933 | unnamed protein product |
| AS016141 | contig_933 | orkhead box protein O |
| AS002049 | contig_58 | putative odorant receptor |
| AS003518 | contig_94 | hypothetical protein B5V51_13340 |
| AS003519 | contig_94 | hypothetical protein B5V51_13341 |
| AS004620 | contig_138 | odorant receptor 94a |
| AS010683 | contig_288 | protein real-time |
| AS014533 | contig_608 | putative E3 ubiquitin-protein ligase SINAT1 |
| AS005533 | contig_153 | hypothetical protein B5V51_9932 |
| AS005532 | contig_153 | inhibitor of Bruton tyrosine kinase |
| AS014740 | contig_633 | NA |
| AS014741 | contig_633 | myosin-light-chain kinase |
| AS017437 | contig_1567 | BRCA2-interacting transcriptional repressor EMSY |
| AS017436 | contig_1567 | aspartate aminotransferase |
| AS014658 | contig_629 | solute carrier family 30 (zinc transporter) |
| AS014660 | contig_629 | 60S ribosomal protein L31 |
| AS014659 | contig_629 | UFM1-specific protease 2 |
| AS005376 | contig_153 | SWI/SNF-related matrix-associated actin-dependent regulator of chromatin subfamily A-like protein 1 |
| AS005621 | contig_154 | acetylcholinesterase |
| AS005620 | contig_154 | putative arylhydrocarbon receptor nuclear translocator protein b |
| AS008355 | contig_218 | fumarate hydratase |
| AS008356 | contig_218 | vacuolar protein sorting-associated protein 3 |
| AS008595 | contig_221 | Ras-related protein Rab-1A |
| AS017642 | contig_1664 | unnamed protein product |
| AS014703 | contig_629 | NA |
| AS004479 | contig_134 | electron transfer flavoprotein-ubiquinone oxidoreductase |
| AS008852 | contig_235 | pancreatic triacylglycerol lipase-like |
| AS008853 | contig_235 | succinate dehydrogenase (ubiquinone) flavoprotein subunit |
| AS008854 | contig_235 | ancreatic triacylglycerol lipase-like |
| AS018218 | contig_1907 | WAS protein family homolog 1 |
| AS001635 | contig_40 | NA |
| AS001636 | contig_40 | hypothetical protein B5V51_5030 |
| AS001634 | contig_40 | hypothetical protein B5V51_5031 |
| AS001100 | contig_34 | exportin-T |
| AS002647 | contig_71 | geranylgeranyl transferase type-2 subunit beta |
| AS002648 | contig_71 | forkhead box protein J1 |
| AS004817 | contig_140 | protein obstructor-E-like |
| AS011984 | contig_345 | fatty alcohol acetyltransferase |
| AS011983 | contig_345 | uncharacterized protein LOC110378016 |
| AS015129 | contig_662 | gustatory receptor |
| AS010623 | contig_288 | alkaline phosphatase, tissue-nonspecific isozyme-like |
| AS009363 | contig_240 | myosin I |
| AS009223 | contig_240 | ATP-binding cassette |
| AS010467 | contig_284 | 7,8-dihydro-8-oxoguanine triphosphatase-like |
| AS010468 | contig_284 | nuclear pore complex protein Nup85 |
| AS016872 | contig_1212 | uncharacterized protein LOC111359698 |
| AS016873 | contig_1212 | NA |
| AS009364 | contig_240 | ATP synthase mitochondrial F1 complex assembly factor 1 |
| AS009366 | contig_240 | actin-like protein 6B |
| AS009365 | contig_240 | cyclin-dependent kinase 2 |
| AS008903 | contig_236 | cAMP-specific phosphodiesterase 4 |
| AS008904 | contig_236 | uncharacterized protein LOC113511545 |
| AS004779 | contig_139 | KRAB domain-containing zinc finger protein |
| AS004780 | contig_139 | chloride channel |
| AS011798 | contig_339 | myb-like protein P |
| AS011799 | contig_339 | NA |
| AS015574 | contig_783 | ras-related protein Rac1 |
| AS012486 | contig_378 | dopamine N-acetyltransferase-like |
| AS001987 | contig_57 | hypothetical protein B5V51_5319 |
| AS001988 | contig_57 | ABC transporter C family member 13 |
| AS010459 | contig_280 | hypothetical protein B5V51_2811 |
| AS010460 | contig_280 | RNA binding protein fox-1 |
| AS006597 | contig_171 | uncharacterized protein LOC110374234 |
| AS002668 | contig_71 | glutaminyl-peptide cyclotransferase |
| AS006793 | contig_171 | JNK-interacting protein 1 |
| AS003520 | contig_94 | hypothetical protein B5V51_13342 |
| AS003521 | contig_94 | KRAB domain-containing zinc finger protein |
| AS012928 | contig_481 | solute carrier family 50 (sugar transporter) |
| AS004058 | contig_114 | exosome complex exonuclease DIS3/RRP44 |
| AS016099 | contig_932 | golgin subfamily A member 2-like |
| AS010129 | contig_263 | desert hedgehog protein B |
| AS000249 | contig_6 | probable protein phosphatase 2C T23F11.1 |
| AS012632 | contig_432 | NA |
| AS012633 | contig_432 | hypothetical protein B5X24_HaOG206867 |
| AS015796 | contig_831 | putative pumilio |
| AS004610 | contig_138 | ATP-dependent RNA helicase DDX18-like |
| AS004611 | contig_138 | protein kinase C substrate 80K-H |
| AS004612 | contig_138 | splicing factor 3A subunit 3 |
| AS014879 | contig_635 | uncharacterized protein LOC111358795 |
| AS003891 | contig_109 | nuclear receptor coactivator 2 |
| AS004468 | contig_134 | sorting nexin-13 |
| AS000426 | contig_9 | uncharacterized protein K02A2.6-like |
| AS000552 | contig_26 | eukaryotic translation initiation factor 4B |
| AS000553 | contig_26 | tyrosine-protein kinase Fer |
| AS002515 | contig_66 | NA |
| AS000537 | contig_25 | aminoacylase |
| AS000538 | contig_25 | 2-hydroxyglutarate dehydrogenase |
| AS011496 | contig_336 | cubilin |
| AS000716 | contig_29 | androgen-dependent TFPI-regulating protein-like |
| AS004337 | contig_129 | uncharacterized protein LOC110377497 |
| AS013923 | contig_561 | protein SSXT |
| AS013924 | contig_561 | 39S ribosomal protein L3 |
| AS001681 | contig_40 | protein SMG7 |
| AS011156 | contig_325 | mucin-2 |
| AS011157 | contig_325 | tRNA-splicing endonuclease subunit Sen15 |
| AS008857 | contig_235 | erythrocyte membrane protein band 4.1 |
| AS006396 | contig_171 | coiled-coil domain-containing protein AGAP005037 |

Notes: NA (not applicable).

**Table S12. Genes of NTC selected region genes identified by *F*_ST_ and π between XJ and NTC**

| **Gene ID** | **Contig** | **Annotation** | |
| --- | --- | --- | --- |
| AS000867 | contig_31 | cytochrome P450 | |
| AS003122 | contig_82 | cytochrome P450 | |
| AS003123 | contig_82 | cytochrome P450 | |
| AS006231 | contig_169 | cytochrome P450 | |
| AS008692 | contig_227 | cytochrome P450 | |
| AS009896 | contig_254 | cytochrome P450 | |
| AS015666 | contig_796 | cytochrome P450 | |
| AS015667 | contig_796 | cytochrome P450 | |
| AS015668 | contig_796 | cytochrome P450 | |
| AS015669 | contig_796 | cytochrome P450 | |
| AS015828 | contig_843 | cytochrome P450 | |
| AS016047 | contig_919 | cytochrome P450 | |
| AS017403 | contig_1549 | cytochrome P450 | |
| AS017404 | contig_1549 | cytochrome P450 | |
| AS000668 | contig_28 | acetylcholinesterase | |
| AS001443 | contig_37 | acetylcholinesterase | |
| AS005365 | contig_152 | glucuronosyltransferase | |
| AS009334 | contig_240 | scavenger receptor class B | |
| AS010222 | contig_265 | carboxylesterase type B (EC:3.1.1.1) | |
| AS004441 | contig_134 | gustatory receptor | |
| AS004442 | contig_134 | gustatory receptor | |
| AS008339 | contig_218 | gustatory receptor | |
| AS012545 | contig_407 | gustatory receptor | |
| AS012546 | contig_407 | gustatory receptor | |
| AS017704 | contig_1672 | gustatory receptor | |
| AS017705 | contig_1672 | gustatory receptor | |
| AS004317 | contig_129 | stearoyl-CoA desaturase (Delta-9 desaturase) | |
| AS016542 | contig_1078 | stearoyl-CoA desaturase (Delta-9 desaturase) | |
| AS018260 | contig_1928 | stearoyl-CoA desaturase (Delta-9 desaturase) | |
| AS005806 | contig_159 | acetyl-CoA carboxylase / biotin carboxylase 1 | |
| AS006303 | contig_169 | fatty acid synthase | |
| AS006305 | contig_169 | fatty acid synthase | |
| AS006306 | contig_169 | fatty acid synthase | |
| AS004051 | contig_114 | elongation of very long chain fatty acids protein 4 | |
| AS005914 | contig_161 | elongation of very long chain fatty acids protein 7 | |
| AS005915 | contig_161 | elongation of very long chain fatty acids protein 7 | |
| AS005923 | contig_161 | elongation of very long chain fatty acids protein 7 | |
| AS017590 | contig_1631 | elongation of very long chain fatty acids protein 7 | |
| AS005892 | contig_159 | alcohol-forming fatty acyl-CoA reductase | |
| AS012539 | contig_380 | alcohol-forming fatty acyl-CoA reductase | |
| AS017019 | contig_1307 | alcohol-forming fatty acyl-CoA reductase | |
| AS017021 | contig_1307 | alcohol-forming fatty acyl-CoA reductase | |
| AS014241 | contig_577 | pyruvate | |
| AS014242 | contig_577 | pyruvate | |
| AS010226 | contig_265 | acetylcholinesterase | |
| AS003129 | contig_82 | aldehyde reductase | |
| AS003275 | contig_82 | TBC1 domain family member 15 | |
| AS003952 | contig_113 | serine/threonine-protein kinase MRCK | |
| AS006637 | contig_171 | protein tyrosine phosphatase type IVA | |
| AS006878 | contig_174 | abelson tyrosine-protein kinase 1 | |
| AS006879 | contig_174 | abelson tyrosine-protein kinase 1 | |
| AS006880 | contig_174 | abelson tyrosine-protein kinase 1 | |
| AS009898 | contig_254 | gonadotropin-releasing hormone receptor | |
| AS009900 | contig_254 | gonadotropin-releasing hormone receptor | |
| AS013017 | contig_481 | nitric-oxide synthase | |
| AS017154 | contig_1400 | phosphoinositide 3-kinase adapter protein 1 | |
| AS017020 | contig_1307 | phosphoinositide-3-kinase | |
| AS008704 | contig_227 | cyclic AMP-responsive element-binding protein 3 | |
| AS017452 | contig_1573 | von Hippel-Lindau disease tumor suppressor | |
| AS002993 | contig_82 | E3 ubiquitin-protein ligase SIAH1 | |
| AS011775 | contig_339 | exportin-1 | |
| AS016543 | contig_1078 | glutamate receptor 2 | |
| AS001437 | contig_37 | protein toll | |
| AS010754 | contig_297 | bile salt-stimulated lipase | |
| AS000265 | contig_9 | uncharacterized protein LOC113230887 |  |
| AS000293 | contig_9 | hypothetical protein EVAR_10313_1 |  |
| AS000294 | contig_9 | LL13 |  |
| AS000868 | contig_31 | anaphase-promoting complex subunit 4 |  |
| AS001329 | contig_36 | NA |  |
| AS001432 | contig_37 | unnamed protein product |  |
| AS001438 | contig_37 | orexin receptor type 1 |  |
| AS001441 | contig_37 | hypothetical protein B5X24_HaOG201003 |  |
| AS001464 | contig_37 | hypothetical protein evm_011754 |  |
| AS001477 | contig_37 | MFS transporter |  |
| AS001478 | contig_37 | MFS transporter |  |
| AS001481 | contig_37 | NA |  |
| AS001487 | contig_37 | ribosome biogenesis protein MAK21 |  |
| AS001490 | contig_37 | uncharacterized protein LOC113508141 |  |
| AS001494 | contig_37 | orexin receptor type 1-like |  |
| AS001501 | contig_37 | hypothetical protein AB894_15215 |  |
| AS001574 | contig_38 | WASH complex subunit 2 |  |
| AS002144 | contig_58 | protein B5V51_407 |  |
| AS002145 | contig_58 | uncharacterized protein LOC114358071 isoform X1 |  |
| AS002470 | contig_66 | leucine-rich repeat-containing G-protein coupled receptor 5 |  |
| AS002712 | contig_76 | oxysterol-binding protein-related protein 3/6/7 |  |
| AS002992 | contig_82 | NA |  |
| AS003180 | contig_82 | dynein heavy chain |  |
| AS003383 | contig_86 | solute carrier family 4 (anion exchanger) |  |
| AS003384 | contig_86 | solute carrier family 4 (anion exchanger) |  |
| AS003385 | contig_86 | solute carrier family 4 (anion exchanger) |  |
| AS003450 | contig_94 | MFS transporter |  |
| AS003451 | contig_94 | uncharacterized protein LOC113492765 |  |
| AS003691 | contig_99 | protein antagonist of like heterochromatin protein 1-like |  |
| AS003692 | contig_99 | hypothetical protein PPYR_15052 |  |
| AS004445 | contig_134 | synaptotagmin-10 |  |
| AS004510 | contig_135 | nose resistant to fluoxetine protein 6-like |  |
| AS004511 | contig_135 | nose resistant to fluoxetine protein 6-like |  |
| AS004516 | contig_135 | nose resistant to fluoxetine protein 6-like |  |
| AS004517 | contig_135 | nose resistant to fluoxetine protein 6-like |  |
| AS004554 | contig_138 | phosphatidylinositol glycan |  |
| AS004628 | contig_138 | hypothetical protein B5X24_HaOG212154 |  |
| AS005303 | contig_152 | nose resistant to fluoxetine protein 6-like |  |
| AS005334 | contig_152 | NA |  |
| AS006104 | contig_163 | NA |  |
| AS006232 | contig_169 | unnamed protein product |  |
| AS006406 | contig_171 | hypothetical protein evm_004341 |  |
| AS006485 | contig_171 | hypothetical protein B5V51_5679 |  |
| AS006615 | contig_171 | MFS transporter |  |
| AS006636 | contig_171 | imaginal disc growth factor-like protein |  |
| AS006658 | contig_171 | period circadian protein |  |
| AS006775 | contig_171 | uncharacterized protein LOC111360675 |  |
| AS006970 | contig_174 | unnamed protein product |  |
| AS006971 | contig_174 | uncharacterized protein LOC108560498 |  |
| AS007228 | contig_183 | protein B5V51_5613 |  |
| AS007229 | contig_183 | early growth response protein 3 |  |
| AS007751 | contig_203 | 357_80_1 protein |  |
| AS007752 | contig_203 | HMG176 isoform A |  |
| AS007842 | contig_204 | uncharacterized protein LOC110372178 |  |
| AS007855 | contig_204 | probable aldehyde oxidase gad-3 |  |
| AS007856 | contig_204 | uncharacterized protein LOC111356580 |  |
| AS008029 | contig_207 | solute carrier family 12 (sodium/potassium/chloride transporter) |  |
| AS008343 | contig_218 | uncharacterized protein LOC113500226 |  |
| AS008608 | contig_223 | NA |  |
| AS008609 | contig_223 | EF-hand domain-containing protein 1-like  DNA-directed RNA polymerase II subunit RPB1 |  |
| AS008693 | contig_227 |  |  |
| AS008695 | contig_227 | sarcosine oxidase / L-pipecolate oxidase |  |
| AS008701 | contig_227 | synaptotagmin-6 |  |
| AS008844 | contig_235 | uncharacterized protein LOC110376641 |  |
| AS009092 | contig_238 | uncharacterized protein LOC114365243 |  |
| AS009095 | contig_238 | protein B5V51_13147 |  |
| AS009505 | contig_247 | alcohol dehydrogenase |  |
| AS009506 | contig_247 | uncharacterized protein LOC113494713 |  |
| AS009507 | contig_247 | alcohol dehydrogenase |  |
| AS009662 | contig_250 | farnesyl diphosphate synthase |  |
| AS009663 | contig_250 | farnesyl diphosphate synthase |  |
| AS009664 | contig_250 | farnesyl diphosphate synthase |  |
| AS009665 | contig_250 | farnesyl diphosphate synthase |  |
| AS009666 | contig_250 | farnesyl diphosphate synthase |  |
| AS009667 | contig_250 | farnesyl diphosphate synthase |  |
| AS009816 | contig_251 | mucin-17 |  |
| AS009899 | contig_254 | hypothetical protein evm_014465 |  |
| AS009922 | contig_256 | uncharacterized protein LOC110371998 |  |
| AS010048 | contig_259 | NA |  |
| AS010049 | contig_259 | NA |  |
| AS010622 | contig_288 | uncharacterized protein LOC110372423 |  |
| AS010778 | contig_300 | PREDICTED: uncharacterized protein LOC106104121 |  |
| AS010779 | contig_300 | neuronal PAS domain-containing protein 1/3 |  |
| AS010780 | contig_300 | protein trachealess-like |  |
| AS010942 | contig_308 | protein B5V51_3074 |  |
| AS011360 | contig_330 | craniofacial development protein 2-like |  |
| AS011483 | contig_336 | craniofacial development protein 2-like |  |
| AS011774 | contig_339 | DNA repair endonuclease XPF |  |
| AS011776 | contig_339 | katanin p80 WD40 repeat-containing subunit B1 |  |
| AS011777 | contig_339 | uncharacterized protein K02A2.6-like |  |
| AS012003 | contig_345 | NA |  |
| AS012407 | contig_374 | NA |  |
| AS012475 | contig_378 | NA |  |
| AS012540 | contig_380 | unnamed protein product |  |
| AS012544 | contig_407 | RNA binding protein fox-1 |  |
| AS012547 | contig_407 | hypothetical protein B5X24_HaOG203342 |  |
| AS012680 | contig_437 | hypothetical protein evm_006094 |  |
| AS012681 | contig_437 | Uncharacterized protein OBRU01_19419 |  |
| AS012769 | contig_447 | probable ATP-dependent RNA helicase DHX35 |  |
| AS012847 | contig_448 | protein MON2 homolog |  |
| AS013121 | contig_487 | meso-butanediol dehydrogenase / (S,S)-butanediol dehydrogenase / diacetyl reductase |  |
| AS013122 | contig_487 | hypothetical protein B5V51_12826 |  |
| AS013123 | contig_487 | meso-butanediol dehydrogenase / (S,S)-butanediol dehydrogenase / diacetyl reductase |  |
| AS013124 | contig_487 | hypothetical protein B5V51_12826 |  |
| AS013373 | contig_505 | odorant binding protein |  |
| AS013374 | contig_505 | domain-containing protein |  |
| AS014290 | contig_601 | twitchin |  |
| AS014656 | contig_629 | uncharacterized protein LOC110378242 |  |
| AS014657 | contig_629 | uncharacterized protein LOC114361369 |  |
| AS015066 | contig_655 | uncharacterized protein LOC111359190 |  |
| AS015225 | contig_688 | NA |  |
| AS015226 | contig_688 | protein B5V51_407 |  |
| AS015737 | contig_809 | NA |  |
| AS015836 | contig_843 | protein FAM50 |  |
| AS015837 | contig_843 | protein B5V51_12645 |  |
| AS015838 | contig_843 | centromeric protein E |  |
| AS015840 | contig_843 | NA |  |
| AS015841 | contig_843 | putative defensin-like protein precursor |  |
| AS015847 | contig_843 | tenascin-like isoform X3 |  |
| AS015848 | contig_843 | uncharacterized protein LOC110371008 |  |
| AS015853 | contig_843 | SWI/SNF-related matrix-associated actin-dependent regulator of chromatin subfamily A-like protein 1 |  |
| AS016035 | contig_917 | solute carrier family 36 (proton-coupled amino acid transporter) |  |
| AS016036 | contig_917 | protein yellow |  |
| AS016048 | contig_919 | protein B5V51_156 |  |
| AS016533 | contig_1078 | protein B5V51_2740 |  |
| AS016540 | contig_1078 | RNA binding protein fox-1 |  |
| AS016541 | contig_1078 | pyrokinin-1 receptor-like |  |
| AS016994 | contig_1285 | NA |  |
| AS017153 | contig_1400 | J domain-containing protein |  |
| AS017193 | contig_1431 | optic atrophy protein 1 |  |
| AS017579 | contig_1624 | SWI/SNF-related matrix-associated actin-dependent regulator of chromatin subfamily A-like protein 1 |  |
| AS017580 | contig_1624 | serine carboxypeptidase 1 |  |
| AS017613 | contig_1664 | thyrostimulin beta-5 subunit-like |  |
| AS017642 | contig_1664 | unnamed protein product |  |
| AS017652 | contig_1664 | uncharacterized protein LOC114361250 |  |
| AS017916 | contig_1887 | hypothetical protein B5X24_HaOG209536 |  |
| AS018347 | contig_1938 | uncharacterized protein LOC113508244 |  |
| AS018348 | contig_1938 | unnamed protein product |  |
| AS018349 | contig_1938 | endonuclease-reverse transcriptase |  |

Notes: NA (not applicable).

**Table S13. Genes of XJ selected region identified by *F*_ST_ and π between XJ and NTC**

| **Gene ID** | **Contig** | **Annotation** |
| --- | --- | --- |
| AS005019 | contig_140 | multidrug resistance-associated protein 4 |
| AS005189 | contig_151 | glutathione S-transferase |
| AS010228 | contig_265 | carboxylesterase type B |
| AS010229 | contig_265 | carboxyl/choline esterase CCE001c |
| AS012575 | contig_414 | cytochrome P450 |
| AS012576 | contig_414 | cytochrome P450 |
| AS017415 | contig_1558 | trypsin |
| AS002026 | contig_58 | glucuronosyltransferase |
| AS002027 | contig_58 | glucuronosyltransferase |
| AS005355 | contig_152 | glucuronosyltransferase |
| AS005143 | contig_149 | trehalose 6-phosphate synthase/phosphatase |
| AS005914 | contig_161 | elongation of very long chain fatty acids protein 7 |
| AS006305 | contig_169 | fatty acid synthase |
| AS006392 | contig_171 | protein nanos 1 |
| AS010434 | contig_280 | hexokinase type 2 isoform X2 |
| AS008409 | contig_219 | diacylglycerol kinase (ATP) |
| AS016538 | contig_1078 | desaturase |
| AS017592 | contig_1631 | elongation of very long chain fatty acids protein 7 |
| AS018126 | contig_1899 | multiple inositol-polyphosphate phosphatase / 2,3-bisphosphoglycerate 3-phosphatase |
| AS000078 | contig_4 | solute carrier family 6 (neurotransmitter transporter) |
| AS000133 | contig_4 | ubiquitin-like modifier-activating enzyme ATG7 |
| AS001089 | contig_34 | prickle |
| AS002593 | contig_70 | transmembrane protease serine 9 |
| AS003603 | contig_96 | sphingomyelin phosphodiesterase acid-like 3 |
| AS003952 | contig_113 | serine/threonine-protein kinase MRCK |
| AS004060 | contig_114 | solute carrier family 36 (proton-coupled amino acid transporter) |
| AS006414 | contig_171 | insulin |
| AS006145 | contig_167 | nicotinate phosphoribosyltransferase |
| AS006146 | contig_167 | KRAB domain-containing zinc finger protein |
| AS006147 | contig_167 | transcriptional coactivator YAP1 |
| AS006180 | contig_168 | inhibitor of apoptosis protein |
| AS006473 | contig_171 | somatostatin receptor 2 |
| AS006804 | contig_171 | tyrosine-protein phosphatase non-receptor type 1 |
| AS006877 | contig_174 | zinc finger protein 107-like |
| AS006878 | contig_174 | abelson tyrosine-protein kinase 1 |
| AS007132 | contig_175 | phosphorylase kinase alpha/beta subunit |
| AS007620 | contig_198 | Ras-related GTP-binding protein C/D |
| AS008532 | contig_219 | disks large protein 1 |
| AS008679 | contig_226 | kallikrein 14 |
| AS008874 | contig_235 | Rad and Gem related GTP binding protein 1 |
| AS011780 | contig_339 | serine/threonine-protein kinase MRCK |
| AS012005 | contig_345 | tyrosine kinase receptor Cad96Ca |
| AS012016 | contig_345 | protein toll |
| AS012329 | contig_372 | G-protein signaling modulator 2 |
| AS013017 | contig_481 | nitric-oxide synthase |
| AS014875 | contig_635 | cyclin-dependent kinase 14 |
| AS015010 | contig_654 | NEDD8-activating enzyme E1 catalytic subunit |
| AS015013 | contig_654 | ubiquinol-cytochrome-c reductase complex assembly factor 1 |
| AS015016 | contig_654 | 60S ribosomal protein L24 |
| AS015017 | contig_654 | sestrin 2 |
| AS015270 | contig_704 | G protein-coupled receptor Mth |
| AS018441 | contig_1963 | myosin IX |
| AS018442 | contig_1972 | G protein-coupled receptor Mth |
| AS015460 | contig_765 | integrin beta 1 |
| AS005320 | contig_152 | dynein heavy chain |
| AS000070 | contig_4 | endocuticle structural glycoprotein SgAbd-2-like |
| AS000413 | contig_9 | estrogen sulfotransferase-like |
| AS000414 | contig_9 | sulfotransferase 1C4-like |
| AS000509 | contig_24 | NA |
| AS000510 | contig_24 | protein B5V51_8355 |
| AS000511 | contig_24 | uncharacterized protein LOC110380247 |
| AS000521 | contig_25 | DNA polymerase |
| AS000544 | contig_26 | vacuolar protein sorting-associated protein 16 |
| AS000717 | contig_29 | androgen-dependent TFPI-regulating protein-like |
| AS000718 | contig_29 | androgen-dependent TFPI-regulating protein-like |
| AS001090 | contig_34 | uncharacterized protein LOC114355939 |
| AS001091 | contig_34 | WD and tetratricopeptide repeats protein 1 |
| AS001092 | contig_34 | intermediate filament protein if |
| AS001174 | contig_34 | NA |
| AS001288 | contig_35 | uncharacterized protein LOC114356249 |
| AS001467 | contig_37 | uncharacterized protein LOC114363995 |
| AS001485 | contig_37 | NA |
| AS001486 | contig_37 | Retrotransposable element |
| AS001488 | contig_37 | NA |
| AS001502 | contig_37 | MFS transporter |
| AS001511 | contig_37 | hypothetical protein AB894_15215 |
| AS001512 | contig_37 | RNA binding protein fox-1 |
| AS001594 | contig_40 | NA |
| AS001976 | contig_57 | hrp65 protein-like |
| AS001977 | contig_57 | uncharacterized protein KIAA1143 homolog |
| AS001978 | contig_57 | transforming growth factor beta regulator 1 |
| AS001980 | contig_57 | NA |
| AS001981 | contig_57 | protein B5V51_5309 |
| AS002712 | contig_76 | oxysterol-binding protein-related protein 3/6/7 |
| AS002713 | contig_76 | PREDICTED: uncharacterized protein LOC106142263 |
| AS002714 | contig_76 | uncharacterized protein LOC111349335 |
| AS002716 | contig_76 | hypothetical protein OBRU01_03615 |
| AS002720 | contig_76 | hypothetical protein AB894_15215 |
| AS002767 | contig_78 | hypothetical protein B5X24_HaOG203025 |
| AS003012 | contig_82 | NA |
| AS003013 | contig_82 | NA |
| AS003037 | contig_82 | protein B5V51_14853 |
| AS003110 | contig_82 | myosin XVIII |
| AS003132 | contig_82 | E3 ubiquitin-protein ligase MYCBP2 |
| AS003286 | contig_82 | uncharacterized protein LOC110370955 |
| AS003287 | contig_82 | uncharacterized protein LOC111360097 |
| AS003291 | contig_82 | uncharacterized protein LOC110370955 |
| AS003292 | contig_82 | uncharacterized protein LOC110370955 |
| AS003293 | contig_82 | uncharacterized protein LOC111360097 |
| AS003328 | contig_85 | lymphocyte antigen 75-like |
| AS003540 | contig_96 | uncharacterized protein LOC111355624 |
| AS003541 | contig_96 | protein B5V51_4716 |
| AS003542 | contig_96 | uncharacterized protein LOC113501421 |
| AS003951 | contig_113 | hypothetical protein evm_001344 |
| AS003953 | contig_113 | uncharacterized protein LOC111362373 |
| AS004126 | contig_120 | NA |
| AS004175 | contig_122 | protein B5V51_5113 |
| AS004335 | contig_129 | uncharacterized protein LOC111357516 |
| AS004468 | contig_134 | uncharacterized protein LOC101736981 |
| AS004600 | contig_138 | NA |
| AS004789 | contig_139 | DNA excision repair protein ERCC-3 |
| AS004859 | contig_140 | unnamed protein product |
| AS005271 | contig_152 | SWI/SNF-related matrix-associated actin-dependent regulator of chromatin subfamily A-like protein 1 |
| AS005299 | contig_152 | NA |
| AS005300 | contig_152 | NA |
| AS005301 | contig_152 | protein B5V51_6442 |
| AS005319 | contig_152 | hypothetical protein B5X24_HaOG208307 |
| AS005566 | contig_154 | uncharacterized protein LOC106132896 |
| AS005878 | contig_159 | Histone-lysine N-methyltransferase SETMAR |
| AS006179 | contig_168 | NA |
| AS006181 | contig_168 | DNA mismatch repair protein MSH2 |
| AS006396 | contig_171 | coiled-coil domain-containing protein AGAP005037 |
| AS006400 | contig_171 | piggyBac transposable element-derived protein 3-like |
| AS006401 | contig_171 | SRC kinase signaling inhibitor 1 |
| AS006403 | contig_171 | basement membrane-specific heparan sulfate proteoglycan core protein |
| AS006423 | contig_171 | paraplegin |
| AS006433 | contig_171 | succinate dehydrogenase (ubiquinone) cytochrome b560 subunit |
| AS006442 | contig_171 | uncharacterized protein LOC106142263 |
| AS006513 | contig_171 | DNA damage-responsive transcriptional repressor RPH1 |
| AS006648 | contig_171 | Kip1 ubiquitination-promoting complex protein 1 |
| AS006649 | contig_171 | RING finger and SPRY domain-containing protein 1-like |
| AS006650 | contig_171 | protein B5V51_1187 |
| AS006737 | contig_171 | craniofacial development protein 2-like |
| AS006738 | contig_171 | endonuclease-reverse transcriptase |
| AS006796 | contig_171 | NA |
| AS006797 | contig_171 | NA |
| AS006812 | contig_171 | NA |
| AS007096 | contig_174 | Arf-GAP with Rho-GAP domain |
| AS007131 | contig_175 | uncharacterized protein LOC111358654 |
| AS007238 | contig_183 | F-type H+-transporting ATPase subunit O |
| AS007239 | contig_183 | C-Myc-binding protein |
| AS007240 | contig_183 | T-complex protein 1 subunit theta |
| AS007242 | contig_183 | uncharacterized protein LOC111357700 |
| AS007244 | contig_183 | uncharacterized protein LOC113505275 |
| AS007569 | contig_197 | uncharacterized protein LOC111349371 |
| AS007570 | contig_197 | uncharacterized protein LOC111357833 |
| AS007676 | contig_201 | uncharacterized protein LOC110379761 |
| AS007837 | contig_204 | uncharacterized protein LOC113503851 |
| AS007838 | contig_204 | uncharacterized protein LOC113503851 |
| AS007979 | contig_206 | protein B5V51_407 |
| AS007980 | contig_206 | spherulin-2A-like |
| AS007981 | contig_206 | protein B5V51_407 |
| AS007982 | contig_206 | uncharacterized protein LOC110380165 |
| AS007987 | contig_206 | protein B5V51_407 |
| AS008017 | contig_207 | retinol dehydrogenase 12-like |
| AS008347 | contig_218 | cellular nucleic acid-binding protein |
| AS008531 | contig_219 | uncharacterized protein LOC112044007 |
| AS008699 | contig_227 | venom dipeptidyl peptidase 4-like isoform X1 |
| AS008763 | contig_231 | calpain |
| AS009028 | contig_237 | NA |
| AS009034 | contig_237 | pickpocket protein 28-like |
| AS009038 | contig_237 | NA |
| AS009039 | contig_237 | hypothetical protein evm_011278 |
| AS009080 | contig_238 | putative odorant receptor |
| AS009323 | contig_240 | peptidyl-prolyl isomerase G (cyclophilin G) |
| AS009460 | contig_245 | hypothetical protein B5X24_HaOG210857 |
| AS009520 | contig_247 | NA |
| AS009560 | contig_250 | eukaryotic translation elongation factor 1 epsilon-1 |
| AS009561 | contig_250 | NA |
| AS009562 | contig_250 | odorant binding protein |
| AS009563 | contig_250 | NA |
| AS009564 | contig_250 | eukaryotic translation elongation factor 1 epsilon-1 |
| AS009567 | contig_250 | NA |
| AS009568 | contig_250 | NA |
| AS009604 | contig_250 | protein B5V51_9017 |
| AS009708 | contig_251 | Xaa-Pro aminopeptidase |
| AS009709 | contig_251 | adenylyltransferase and sulfurtransferase |
| AS009902 | contig_254 | DNA polymerase |
| AS009903 | contig_254 | DNA polymerase |
| AS009904 | contig_254 | NA |
| AS010037 | contig_259 | uncharacterized protein LOC111355694 |
| AS010067 | contig_260 | hypothetical protein B5X24_HaOG209515 |
| AS010181 | contig_264 | uncharacterized protein LOC110370142 |
| AS010230 | contig_265 | odorant degrading enzyme CXE14 |
| AS010255 | contig_268 | integrin alpha-PS4-like isoform X2 |
| AS010371 | contig_270 | NA |
| AS010372 | contig_270 | uncharacterized protein LOC112043017 |
| AS010373 | contig_270 | sentrin-specific protease 1 |
| AS010431 | contig_280 | BRISC complex subunit FAM175B-like isoform X1 |
| AS010432 | contig_280 | Hermansky-Pudlak syndrome 1 protein |
| AS010433 | contig_280 | dihydroorotate dehydrogenase |
| AS010435 | contig_280 | NA |
| AS010676 | contig_288 | protein B5V51_5825 |
| AS010908 | contig_308 | NA |
| AS010909 | contig_308 | NA |
| AS010910 | contig_308 | uncharacterized protein LOC110370346 |
| AS011035 | contig_319 | estrogen-related receptor |
| AS011197 | contig_325 | uncharacterized protein LOC117175265 |
| AS011198 | contig_325 | unnamed protein product |
| AS011390 | contig_333 | NA |
| AS011476 | contig_336 | nephrin-like |
| AS011480 | contig_336 | putative nuclease HARBI1 isoform X1 |
| AS011481 | contig_336 | uncharacterized protein PB18E9.04c-like |
| AS011800 | contig_339 | histone H2B |
| AS012020 | contig_345 | uncharacterized protein LOC106142263 |
| AS012328 | contig_372 | uncharacterized protein LOC111349843 |
| AS012330 | contig_372 | NA |
| AS012331 | contig_372 | unnamed protein product |
| AS012332 | contig_372 | inner centromere protein A-like |
| AS012333 | contig_372 | cycle protein |
| AS012540 | contig_380 | unnamed protein product |
| AS012643 | contig_432 | uncharacterized protein LOC111362300 |
| AS012654 | contig_434 | NA |
| AS012672 | contig_437 | NA |
| AS012673 | contig_437 | intermediate filament protein if |
| AS012674 | contig_437 | phosphoribosylaminoimidazole carboxylase / phosphoribosylaminoimidazole-succinocarboxamide synthase |
| AS012675 | contig_437 | Transposon Tf2-9 polyprotein |
| AS012680 | contig_437 | hypothetical protein evm_006094 |
| AS012681 | contig_437 | Uncharacterized protein OBRU01_19419 |
| AS012682 | contig_437 | Uncharacterized protein OBRU01_04723 |
| AS012683 | contig_437 | protein B5V51_8997 |
| AS012860 | contig_462 | NA |
| AS012861 | contig_462 | uncharacterized protein LOC114351244 |
| AS013001 | contig_481 | rho GTPase-activating protein 21 |
| AS013301 | contig_496 | zwei Ig domain protein zig-8-like isoform X5 |
| AS013302 | contig_496 | Uncharacterized protein OBRU01_04723 |
| AS013369 | contig_505 | takeout/juvenile hormone binding-like protein |
| AS013370 | contig_505 | odorant binding protein |
| AS013371 | contig_505 | odorant binding protein |
| AS013372 | contig_505 | odorant binding protein |
| AS013373 | contig_505 | odorant binding protein |
| AS013455 | contig_508 | NA |
| AS013623 | contig_520 | membrane-associated progesterone receptor component |
| AS013624 | contig_520 | uncharacterized protein LOC111358188 |
| AS013636 | contig_523 | DNA excision repair protein ERCC-3 |
| AS013637 | contig_523 | SWI/SNF-related matrix-associated actin-dependent regulator of chromatin subfamily A-like protein 1 |
| AS013638 | contig_523 | NA |
| AS014652 | contig_629 | bestrophin-3 |
| AS014991 | contig_654 | uncharacterized protein LOC116777793 |
| AS015009 | contig_654 | uncharacterized protein LOC110374271 |
| AS015011 | contig_654 | uncharacterized protein LOC110374329 |
| AS015012 | contig_654 | F-type H+-transporting ATPase subunit beta |
| AS015014 | contig_654 | 60S ribosomal export protein NMD3 |
| AS015015 | contig_654 | ATP synthase subunit beta |
| AS015101 | contig_657 | hypothetical protein B5X24_HaOG212154 |
| AS015105 | contig_657 | NA |
| AS015200 | contig_683 | uncharacterized protein LOC110381480 |
| AS015851 | contig_843 | hypothetical protein AB894_15215 |
| AS016539 | contig_1078 | NA |
| AS016565 | contig_1094 | protein enabled homolog isoform X1 |
| AS016566 | contig_1094 | probable 26S proteasome non-ATPase regulatory subunit 3 |
| AS016567 | contig_1094 | aconitate hydratase |
| AS017005 | contig_1285 | dmX-like protein 2 isoform X4 |
| AS017381 | contig_1535 | high mobility group protein HMG-I/HMG-Y-like |
| AS017452 | contig_1573 | uncharacterized protein LOC110380748 |
| AS017591 | contig_1631 | NA |
| AS017699 | contig_1666 | uncharacterized protein LOC114355925 |
| AS017797 | contig_1837 | proline-rich protein 36 |
| AS017929 | contig_1887 | nuclear factor of activated T-cells 5-like isoform X1 |
| AS018038 | contig_1893 | hypothetical protein B5X24_HaOG216709 |
| AS018124 | contig_1899 | NA |
| AS018125 | contig_1899 | NA |
| AS018443 | contig_1972 | protein B5V51_3838 |
| AS018442 | contig_1972 | G protein-coupled receptor Mth (Methuselah protein) |
| AS018443 | contig_1972 | hypothetical protein |

Notes: NA (not applicable).

**Table S14. Function and mutation types of four genes in the selected region**

| **Gene ID** | **Contig** | **Function** | **Mutation** | |
| --- | --- | --- | --- | --- |
|  |  |  | **Synonymous** | **Missense** |
| AS015666 | contig_796 | cytochrome P450 | 31 | 21 |
| AS015667 | contig_796 |  | 49 | 10 |
| AS015668 | contig_796 |  | 9 | 2 |
| AS015669 | contig_796 |  | 46 | 16 |
| AS014180 | contig_573 | glycogen phosphorylase | 44 | 2 |
| AS005143 | contig_149 | trehalose 6-phosphate synthase | 66 | 5 |

**Table S15. Genes of NEC selected region identified by *F*_ST_ and π between STC and NEC**

| **Gene ID** | **Contig** | **Annotation** |
| --- | --- | --- |
| AS000036 | contig_1 | NA |
| AS000038 | contig_1 | hypothetical protein AB894_15215 |
| AS000039 | contig_1 | RNA binding protein fox-1 |
| AS000040 | contig_1 | hypothetical protein B5V51_10841 |
| AS000383 | contig_9 | uncharacterized protein LOC115445367 |
| AS000384 | contig_9 | Macrophage mannose receptor 1 |
| AS000453 | contig_10 | ATP-binding cassette sub-family G member 4-like |
| AS000640 | contig_28 | polyprotein |
| AS000867 | contig_31 | cytochrome P450 |
| AS001438 | contig_37 | orexin receptor type 1 |
| AS001509 | contig_37 | uncharacterized protein LOC111355311 |
| AS001510 | contig_37 | NA |
| AS001536 | contig_37 | dipeptidyl-peptidase 4 |
| AS001904 | contig_45 | Uncharacterized protein OBRU01_26018 |
| AS001905 | contig_45 | hypothetical protein evm_011729 |
| AS001906 | contig_45 | calcium-sensing receptor |
| AS002025 | contig_58 | UDP-glycosyltransferase 40F3 |
| AS002026 | contig_58 | UDP-glucuronosyltransferase 40F3 |
| AS002399 | contig_65 | 27 kDa glycoprotein-like isoform X2 |
| AS002401 | contig_65 | protein FAM13A isoform X1 |
| AS002498 | contig_66 | uncharacterized protein LOC106710892 |
| AS002514 | contig_66 | Uncharacterized protein OBRU01_06965 |
| AS002641 | contig_71 | unnamed protein product |
| AS002642 | contig_71 | uncharacterized protein LOC111359584 isoform X1 |
| AS002651 | contig_71 | alpha,alpha-trehalase |
| AS002652 | contig_71 | soluble trehalase |
| AS002653 | contig_71 | soluble trehalase |
| AS003186 | contig_82 | transmembrane protease serine 9-like isoform X2 |
| AS003264 | contig_82 | nardilysin-like |
| AS003265 | contig_82 | cell cycle control protein 50A |
| AS003345 | contig_85 | ubiquitin-like modifier-activating enzyme ATG7 |
| AS004192 | contig_123 | THO complex subunit 7 |
| AS004193 | contig_123 | phospholipase D3/4 |
| AS004222 | contig_128 | pre-mRNA-splicing factor ATP-dependent RNA helicase DHX38/PRP16 |
| AS004248 | contig_129 | oligoribonuclease |
| AS004249 | contig_129 | LETM1 and EF-hand domain-containing protein 1 |
| AS004310 | contig_129 | unnamed protein product |
| AS004311 | contig_129 | unnamed protein product |
| AS004329 | contig_129 | NA |
| AS004330 | contig_129 | hypothetical protein B5V51_2396 |
| AS004497 | contig_135 | uncharacterized protein LOC110377940 isoform X2 |
| AS004506 | contig_135 | uncharacterized protein LOC110377940 isoform X2 |
| AS004633 | contig_138 | NA |
| AS004634 | contig_138 | mediator of RNA polymerase II transcription subunit 4 |
| AS004635 | contig_138 | putative inner dynein arm light chain |
| AS004636 | contig_138 | protein lap4 isoform X12 |
| AS004931 | contig_140 | high affinity copper uptake protein 1-like |
| AS004932 | contig_140 | bromodomain-containing protein 3 |
| AS005001 | contig_140 | uncharacterized protein LOC113492658 |
| AS005002 | contig_140 | mitochondrial chaperone BCS1 |
| AS005143 | contig_149 | trehalose 6-phosphate synthase/phosphatase |
| AS005144 | contig_149 | hypothetical protein B5V51_14012 |
| AS005145 | contig_149 | anaphase-promoting complex subunit 11 |
| AS005146 | contig_149 | hypothetical protein B5V51_14010 |
| AS005189 | contig_151 | glutathione S-transferase |
| AS005303 | contig_152 | nose resistant to fluoxetine protein 6-like |
| AS005730 | contig_156 | odorant binding protein 2 |
| AS005913 | contig_161 | elongation of very long chain fatty acids protein 7 |
| AS005914 | contig_161 | elongation of very long chain fatty acids protein 7 |
| AS005919 | contig_161 | elongation of very long chain fatty acids protein 4 |
| AS005924 | contig_161 | elongation of very long chain fatty acids protein 4 |
| AS005929 | contig_162 | ATP-binding cassette |
| AS005930 | contig_162 | unnamed protein product |
| AS005991 | contig_162 | uncharacterized protein LOC111351402 |
| AS005992 | contig_162 | hypothetical protein B5V51_5265 |
| AS005993 | contig_162 | NA |
| AS005994 | contig_162 | optic atrophy protein 1 |
| AS005995 | contig_162 | uncharacterized protein LOC111351402 |
| AS006171 | contig_168 | NA |
| AS006185 | contig_168 | hypothetical protein B5X24_HaOG208466 |
| AS006305 | contig_169 | fatty acid synthase |
| AS006306 | contig_169 | fatty acid synthase |
| AS006338 | contig_169 | hypothetical protein B5V51_7751 |
| AS006433 | contig_171 | succinate dehydrogenase (ubiquinone) cytochrome b560 subunit |
| AS006485 | contig_171 | hypothetical protein B5V51_5679 |
| AS006556 | contig_171 | calcitonin gene-related peptide type 1 receptor-like isoform X1 |
| AS006619 | contig_171 | serine/threonine-protein kinase OSR1/STK39 |
| AS006655 | contig_171 | uncharacterized protein LOC110374481 isoform X3 |
| AS006811 | contig_171 | uncharacterized protein LOC111355311 |
| AS006812 | contig_171 | NA |
| AS006901 | contig_174 | NA |
| AS006934 | contig_174 | hypothetical protein EVAR_38995_1 |
| AS007081 | contig_174 | transient receptor potential cation channel subfamily A member 1 isoform X3 |
| AS007129 | contig_175 | adenylate cyclase type 9 isoform X1 |
| AS007130 | contig_175 | hypothetical protein evm_013249 |
| AS007131 | contig_175 | uncharacterized protein LOC111358654 |
| AS007132 | contig_175 | phosphorylase kinase alpha/beta subunit |
| AS007244 | contig_183 | uncharacterized protein LOC113505275 |
| AS007246 | contig_183 | D-beta-hydroxybutyrate dehydrogenase |
| AS007546 | contig_196 | NA |
| AS007563 | contig_197 | receptor-type tyrosine-protein phosphatase R |
| AS007566 | contig_197 | multiple inositol-polyphosphate phosphatase / 2,3-bisphosphoglycerate 3-phosphatase |
| AS007687 | contig_202 | ATP-binding cassette |
| AS008107 | contig_208 | cuticle protein 19-like |
| AS008108 | contig_208 | hypothetical protein B5X24_HaOG216945 |
| AS008129 | contig_208 | PREDICTED: piggyBac transposable element-derived protein 3-like |
| AS008206 | contig_216 | NA |
| AS008336 | contig_218 | fatty acid synthase-like |
| AS008337 | contig_218 | fatty acid synthase-like |
| AS008338 | contig_218 | hypothetical protein B5X24_HaOG214498 |
| AS008437 | contig_219 | NA |
| AS008438 | contig_219 | optic atrophy protein 1 |
| AS008519 | contig_219 | protein toll |
| AS008688 | contig_226 | leucine-rich repeat-containing protein 15-like |
| AS008805 | contig_235 | neuropilin and tolloid-like protein 2 |
| AS009028 | contig_237 | NA |
| AS009080 | contig_238 | putative odorant receptor |
| AS009155 | contig_239 | cGMP-specific 3',5'-cyclic phosphodiesterase-like |
| AS009417 | contig_244 | NA |
| AS009548 | contig_250 | sex peptide receptor-like |
| AS009589 | contig_250 | chemosensory protein 3 |
| AS009635 | contig_250 | thrombospondin type-1 domain-containing protein 4-like |
| AS009663 | contig_250 | farnesyl diphosphate synthase |
| AS009859 | contig_252 | uncharacterized protein LOC113240557 |
| AS009922 | contig_256 | uncharacterized protein LOC110371998 |
| AS010025 | contig_259 | D(2) dopamine receptor |
| AS010067 | contig_260 | hypothetical protein B5X24_HaOG209515 |
| AS010082 | contig_261 | uncharacterized protein LOC111349511 isoform X1 |
| AS010222 | contig_265 | carboxylesterase type B |
| AS010223 | contig_265 | hypothetical protein AB894_15215 |
| AS010255 | contig_268 | integrin alpha-X-like |
| AS010433 | contig_280 | dihydroorotate dehydrogenase (quinone) |
| AS010434 | contig_280 | hexokinase |
| AS010435 | contig_280 | NA |
| AS010621 | contig_288 | receptor-type guanylate cyclase gcy-1 |
| AS010622 | contig_288 | uncharacterized protein LOC110372423 |
| AS010627 | contig_288 | carboxylesterase |
| AS011411 | contig_333 | mitogen-activated protein kinase 14B |
| AS011412 | contig_333 | NADH dehydrogenase (ubiquinone) 1 beta subcomplex subunit 6 |
| AS011413 | contig_333 | 18S rRNA-dimethyltransferase |
| AS011414 | contig_333 | transportin-1 |
| AS011539 | contig_337 | fatty alcohol acetyltransferase |
| AS011661 | contig_338 | fatty alcohol acetyltransferase |
| AS011662 | contig_338 | fatty alcohol acetyltransferase |
| AS011775 | contig_339 | exportin-1 |
| AS012002 | contig_345 | carbonic anhydrase 6-like |
| AS012035 | contig_345 | arylsulfatase B |
| AS012042 | contig_345 | uncharacterized protein LOC113507453 |
| AS012043 | contig_345 | uncharacterized protein LOC111355549 isoform X3 |
| AS012175 | contig_359 | solute carrier family 41 member 1-like |
| AS012329 | contig_372 | G-protein signaling modulator 2 |
| AS012477 | contig_378 | uncharacterized protein LOC113501428 |
| AS012745 | contig_446 | odorant receptor 46a-like |
| AS012746 | contig_446 | multiple inositol polyphosphate phosphatase 1-like |
| AS012781 | contig_448 | hypothetical protein B5V51_2174 |
| AS012841 | contig_448 | uncharacterized protein LOC111363452 isoform X3 |
| AS013042 | contig_484 | NA |
| AS013079 | contig_486 | glucose dehydrogenase [FAD, quinone]-like |
| AS013374 | contig_505 | JHBP domain-containing protein |
| AS013375 | contig_505 | hypothetical protein B5X24_HaOG207672 |
| AS013376 | contig_505 | NA |
| AS013481 | contig_510 | uncharacterized protein LOC111357108 isoform X5 |
| AS013482 | contig_510 | uncharacterized protein LOC111355311 |
| AS013512 | contig_512 | Retrotransposable element |
| AS013513 | contig_512 | probable sulfite oxidase, mitochondrial isoform X1 |
| AS013691 | contig_530 | hypothetical protein B5V51_5077 |
| AS013860 | contig_552 | serine/threonine-protein kinase MRCK |
| AS013972 | contig_561 | hypothetical protein B5V51_7607 |
| AS014174 | contig_573 | breast cancer type 1 susceptibility protein-like isoform X1 |
| AS014175 | contig_573 | ATP synthase subunit delta, mitochondrial |
| AS014180 | contig_573 | glycogen phosphorylase |
| AS014181 | contig_573 | unnamed protein product |
| AS014525 | contig_608 | reverse transcriptase |
| AS014777 | contig_633 | 60S ribosome subunit biogenesis protein NIP7 |
| AS014860 | contig_635 | cytochrome c1-2, heme protein |
| AS014861 | contig_635 | hypothetical protein B5V51_14976 |
| AS014862 | contig_635 | protein LSM14 homolog A |
| AS014863 | contig_635 | kelch-like protein 10 |
| AS014864 | contig_635 | 15-hydroxyprostaglandin dehydrogenase [NAD (+)]-like |
| AS014879 | contig_635 | uncharacterized protein LOC111358795 |
| AS014880 | contig_635 | hypothetical protein B5X24_HaOG212560 |
| AS014937 | contig_635 | dual specificity protein phosphatase 22-A-like |
| AS014999 | contig_654 | NA |
| AS015367 | contig_757 | uncharacterized protein LOC111354007 |
| AS015660 | contig_788 | fatty acyl reductase 12 |
| AS015661 | contig_788 | putative fatty acyl-CoA reductase CG5065 |
| AS015865 | contig_844 | retinaldehyde-binding protein 1 |
| AS015913 | contig_891 | long-chain fatty acid transport protein 4 |
| AS016130 | contig_932 | collagen alpha-1(IV) chain |
| AS016141 | contig_933 | forkhead box protein O isoform X2 |
| AS016163 | contig_933 | uncharacterized protein LOC111355610 |
| AS016763 | contig_1123 | hypothetical protein B5X24_HaOG208643 |
| AS017029 | contig_1307 | fatty acyl reductase |
| AS017030 | contig_1307 | uncharacterized protein LOC115445367 |
| AS017084 | contig_1342 | hypothetical protein B5V51_4081 |
| AS017157 | contig_1400 | uncharacterized protein LOC114352369 |
| AS017158 | contig_1400 | hypothetical protein B5V51_13622 |
| AS017159 | contig_1400 | T-cell activation inhibitor, mitochondrial-like |
| AS017167 | contig_1420 | juvenile hormone esterase-like |
| AS017168 | contig_1420 | juvenile hormone esterase related protein transcript variant |
| AS017352 | contig_1526 | globin-2 A chain-like |
| AS017353 | contig_1526 | deoxyhypusine monooxygenase |
| AS017354 | contig_1526 | hypothetical protein B5X24_HaOG211462 |
| AS017566 | contig_1624 | proton-coupled folate transporter |
| AS017567 | contig_1624 | proton-coupled folate transporter |
| AS017590 | contig_1631 | elongation of very long chain fatty acids protein 7 |
| AS017655 | contig_1664 | multidrug resistance-associated protein 1-like |
| AS017783 | contig_1788 | ATP synthase subunit alpha, mitochondrial |
| AS017784 | contig_1788 | hypothetical protein B5V51_13982 |
| AS017786 | contig_1792 | monocarboxylate transporter 14-like |
| AS017899 | contig_1887 | sodium/potassium-transporting ATPase subunit beta-1-like |
| AS017928 | contig_1887 | prolactin regulatory element-binding protein |
| AS017929 | contig_1887 | nuclear factor of activated T-cells 5 |
| AS018055 | contig_1893 | trypsin CFT-1-like |
| AS018056 | contig_1893 | WNK lysine deficient protein kinase |
| AS018173 | contig_1899 | NA |
| AS018239 | contig_1908 | zinc/cadmium resistance protein isoform X1 |
| AS018341 | contig_1938 | NA |
| AS018342 | contig_1938 | NA |
| AS018347 | contig_1938 | uncharacterized protein LOC113508244 |
| AS018348 | contig_1938 | unnamed protein product |
| AS018349 | contig_1938 | endonuclease-reverse transcriptase |
| AS018405 | contig_1957 | hypothetical protein B5V51_4935 |
| AS018406 | contig_1957 | protein yellow |

Notes: NA (not applicable).

**Table S16. Genes of STC selected region identified by *F*_ST_ and π between STC and NEC**

| **Gene ID** | **Contig** | **Annotation** |
| --- | --- | --- |
| AS000255 | contig_6 | RecName: Full=FMRFamide-related peptides |
| AS000517 | contig_25 | hypothetical protein B5V51_14463 |
| AS000518 | contig_25 | hypothetical protein B5V51_7957 |
| AS000697 | contig_28 | muscarinic acetylcholine receptor DM1 |
| AS000726 | contig_29 | dynein heavy chain |
| AS000727 | contig_29 | probable chitinase 2 |
| AS001438 | contig_37 | orexin receptor type 1 |
| AS001572 | contig_38 | ribosomal RNA-processing protein 1 |
| AS001573 | contig_38 | ribosomal RNA-processing protein 1 |
| AS001574 | contig_38 | WASH complex subunit FAM21 |
| AS001848 | contig_45 | PiggyBac transposable element-derived protein 3 |
| AS001978 | contig_57 | transforming growth factor beta regulator 1 |
| AS001979 | contig_57 | menin |
| AS001980 | contig_57 | NA |
| AS001981 | contig_57 | hypothetical protein B5V51_5309 |
| AS002423 | contig_66 | phosphopantothenate-cysteine ligase |
| AS002522 | contig_66 | hypothetical protein B5X24_HaOG209640 |
| AS002712 | contig_76 | oxysterol-binding protein-related protein 3/6/7 |
| AS002715 | contig_76 | hypothetical protein EVAR_35820_1 |
| AS002717 | contig_76 | WD and tetratricopeptide repeats protein 1 |
| AS002718 | contig_76 | NA |
| AS002767 | contig_78 | hypothetical protein B5X24_HaOG203025 |
| AS003254 | contig_82 | uncharacterized protein LOC110370821 |
| AS003255 | contig_82 | uncharacterized protein LOC110370822 |
| AS003370 | contig_86 | prickle |
| AS003518 | contig_94 | hypothetical protein B5V51_13340 |
| AS003737 | contig_102 | vacuolar protein sorting-associated protein 72 |
| AS003738 | contig_102 | uncharacterized protein LOC113240557 |
| AS003820 | contig_107 | unnamed protein product |
| AS003899 | contig_109 | trissin receptor-like |
| AS003997 | contig_114 | carbohydrate sulfotransferase 4-like |
| AS003998 | contig_114 | hypothetical protein B5V51_947 |
| AS004085 | contig_115 | hemicentin-1-like |
| AS004175 | contig_122 | hypothetical protein B5V51_5113 |
| AS004176 | contig_122 | uncharacterized protein LOC105383033 |
| AS004220 | contig_128 | adenylate cyclase 2 |
| AS004271 | contig_129 | intermediate filament protein if |
| AS004343 | contig_129 | uncharacterized protein LOC110377500 |
| AS004344 | contig_129 | hypothetical protein evm_014177 |
| AS004620 | contig_138 | odorant receptor 94a |
| AS004621 | contig_138 | uncharacterized protein LOC114360919 |
| AS004624 | contig_138 | uncharacterized protein LOC116770131 |
| AS004628 | contig_138 | tachykinin-like receptor |
| AS005019 | contig_140 | multidrug resistance-associated protein 4 isoform X2 |
| AS005351 | contig_152 | intermediate filament protein if |
| AS005730 | contig_156 | odorant binding protein 2 |
| AS006305 | contig_169 | fatty acid synthase |
| AS006407 | contig_171 | calcitonin receptor |
| AS006648 | contig_171 | Kip1 ubiquitination-promoting complex protein 1 |
| AS006650 | contig_171 | hypothetical protein B5V51_1187 |
| AS006668 | contig_171 | NA |
| AS006731 | contig_171 | piggyBac transposable element-derived protein 4-like isoform X1 |
| AS006780 | contig_171 | uncharacterized protein LOC113521451 |
| AS006790 | contig_171 | uncharacterized protein LOC111355311, |
| AS006804 | contig_171 | tyrosine-protein phosphatase non-receptor type 1 |
| AS006808 | contig_171 | mitogen-activated protein kinase 13 |
| AS006837 | contig_171 | transmembrane channel-like protein 2 isoform X1 |
| AS006838 | contig_171 | NA |
| AS006839 | contig_171 | laminin subunit alpha |
| AS006396 | contig_171 | coiled-coil domain-containing protein AGAP005037 |
| AS006397 | contig_171 | NA |
| AS006516 | contig_171 | solute carrier family 7 (cationic amino acid transporter) |
| AS006517 | contig_171 | uncharacterized protein LOC114355921 |
| AS006402 | contig_171 | solute carrier family 35 (UDP-galactose transporter) |
| AS006553 | contig_171 | hypothetical protein B5V51_13177 |
| AS006563 | contig_171 | EF-hand calcium-binding domain-containing protein 1-like |
| AS006403 | contig_171 | basement membrane-specific heparan sulfate proteoglycan core protein |
| AS006568 | contig_171 | hypothetical protein B5V51_14513 |
| AS006584 | contig_171 | hypothetical protein B5V51_9932 |
| AS006623 | contig_171 | neuronal PAS domain-containing protein 2-like isoform X2 |
| AS006624 | contig_171 | unnamed protein product |
| AS006406 | contig_171 | hypothetical protein evm_004341 |
| AS006927 | contig_174 | retrovirus-related pol polyprotein from transposon tnt 1-94 |
| AS007096 | contig_174 | Arf-GAP with Rho-GAP domain |
| AS006893 | contig_174 | prickle |
| AS006894 | contig_174 | hypothetical protein B5X24_HaOG206296 |
| AS006895 | contig_174 | ephrin type-B receptor 1-B |
| AS007268 | contig_183 | lysine-specific histone demethylase 1A |
| AS007723 | contig_203 | hypothetical protein B5X24_HaOG203521 |
| AS007991 | contig_206 | hypothetical protein B5V51_407 |
| AS008146 | contig_214 | NA |
| AS008147 | contig_214 | RYamide receptor-like |
| AS008265 | contig_216 | carboxypeptidase inhibitor |
| AS009032 | contig_237 | neuropeptide FF receptor 2 |
| AS009033 | contig_237 | pickpocket protein 28-like |
| AS009034 | contig_237 | pickpocket protein 28-like |
| AS009093 | contig_238 | uncharacterized protein LOC113503959 |
| AS009094 | contig_238 | hypothetical protein B5X24_HaOG212193 |
| AS009095 | contig_238 | hypothetical protein B5V51_13147 |
| AS009076 | contig_238 | histone acetyltransferase MYST4 |
| AS009077 | contig_238 | F-box/LRR-repeat protein 15 |
| AS009078 | contig_238 | cytochrome P450 |
| AS009086 | contig_238 | Ig-like and fibronectin type-III domain-containing protein 2 isoform X3 |
| AS009176 | contig_239 | hypothetical protein B5X24_HaOG204559 |
| AS009369 | contig_241 | mannose receptor |
| AS009370 | contig_241 | mannose receptor |
| AS009441 | contig_244 | calmodulin-binding transcription activator 2 |
| AS009455 | contig_244 | phosphatidylinositol-glycan biosynthesis class X protein |
| AS009599 | contig_250 | uncharacterized protein LOC111349709 |
| AS009549 | contig_250 | chemosensory protein 12 |
| AS009667 | contig_250 | farnesyl diphosphate synthase |
| AS009567 | contig_250 | NA |
| AS009568 | contig_250 | NA |
| AS009818 | contig_251 | uncharacterized protein LOC111361297 |
| AS009819 | contig_251 | hypothetical protein B5V51_679 |
| AS009863 | contig_252 | NADH dehydrogenase (ubiquinone) complex I, assembly factor 6 isoform X1 |
| AS009917 | contig_256 | NA |
| AS009918 | contig_256 | NA |
| AS010189 | contig_264 | NA |
| AS010190 | contig_264 | pupal cuticle protein C1B-like |
| AS010191 | contig_264 | pupal cuticle protein C1B-like |
| AS010192 | contig_264 | NA |
| AS010193 | contig_264 | NA |
| AS010194 | contig_264 | NA |
| AS010292 | contig_269 | solute carrier family 24 (sodium/potassium/calcium exchanger) |
| AS011228 | contig_325 | uncharacterized protein LOC113502759 |
| AS011378 | contig_330 | neuropeptide Y receptor |
| AS011379 | contig_330 | neuropeptide Y receptor |
| AS011479 | contig_336 | calcium-sensing |
| AS011481 | contig_336 | uncharacterized protein PB18E9.04c-like |
| AS011743 | contig_338 | hypothetical protein B5V51_2643 |
| AS011779 | contig_339 | putative endonuclease/reverse transcriptase |
| AS011772 | contig_339 | protein-L-isoaspartate(D-aspartate) O-methyltransferase |
| AS011795 | contig_339 | vacuolar protein sorting-associated protein 54 |
| AS011796 | contig_339 | exocyst complex component 3 |
| AS011774 | contig_339 | DNA repair endonuclease XPF |
| AS011812 | contig_340 | succinate dehydrogenase (ubiquinone) flavoprotein subunit |
| AS012002 | contig_345 | carbonic anhydrase |
| AS012016 | contig_345 | protein toll |
| AS012020 | contig_345 | uncharacterized protein LOC106142263 |
| AS012058 | contig_352 | V-type H+-transporting ATPase subunit |
| AS012261 | contig_364 | brahma-associated protein of 60 kDa |
| AS012262 | contig_364 | aquaporin-like |
| AS012269 | contig_364 | uncharacterized protein LOC110383465 |
| AS012270 | contig_364 | hypothetical protein B5V51_10673 |
| AS012341 | contig_372 | hepatic leukemia factor isoform X6 |
| AS012342 | contig_372 | proton-coupled amino acid transporter-like protein CG1139 |
| AS012570 | contig_414 | V-type H+-transporting ATPase S1 subunit |
| AS012769 | contig_447 | probable ATP-dependent RNA helicase DHX35 |
| AS012792 | contig_448 | hypothetical protein B5X24_HaOG211111 |
| AS012793 | contig_448 | NA |
| AS013145 | contig_487 | endonuclease-reverse transcriptase |
| AS013211 | contig_489 | NA |
| AS013503 | contig_512 | gamma-1-syntrophin |
| AS013506 | contig_512 | NA |
| AS013507 | contig_512 | uncharacterized protein LOC105669866 |
| AS013652 | contig_523 | uncharacterized protein LOC111354551 |
| AS013704 | contig_530 | protein jagunal isoform X2 |
| AS013705 | contig_530 | homeobox protein notochord |
| AS013997 | contig_566 | NA |
| AS013998 | contig_566 | NA |
| AS015101 | contig_657 | hypothetical protein B5X24_HaOG212154 |
| AS015183 | contig_681 | NA |
| AS015281 | contig_704 | hypothetical protein B5V51_3484 |
| AS015599 | contig_783 | alkyldihydroxyacetonephosphate synthase |
| AS015580 | contig_783 | NA |
| AS015659 | contig_788 | hypothetical protein B5V51_13662 |
| AS015740 | contig_811 | TATA-box-binding protein |
| AS015741 | contig_811 | eukaryotic translation initiation factor 3 subunit K |
| AS015739 | contig_811 | WASH complex subunit 4 |
| AS015761 | contig_814 | transmembrane protein 135-like |
| AS015866 | contig_845 | protein unc-13 homolog A isoform X3 |
| AS016126 | contig_932 | hypothetical protein B5V51_14479 |
| AS016127 | contig_932 | PITH domain-containing protein GA19395 |
| AS016128 | contig_932 | collagen |
| AS016500 | contig_1071 | zinc finger protein chinmo isoform X2 |
| AS016521 | contig_1072 | alkaline phosphatase D |
| AS016537 | contig_1078 | NA |
| AS016538 | contig_1078 | desaturase |
| AS016540 | contig_1078 | RNA binding protein fox-1 |
| AS016541 | contig_1078 | pyrokinin-1 receptor-like |
| AS016571 | contig_1094 | alpha-methyldopa hypersensitive protein-like |
| AS016625 | contig_1102 | probable splicing factor |
| AS016626 | contig_1102 | cilia- and flagella-associated protein 58-like |
| AS016627 | contig_1102 | dynein heavy chain 12, axonemal |
| AS017126 | contig_1363 | unnamed protein product |
| AS017135 | contig_1374 | homeobox protein goosecoid |
| AS017136 | contig_1374 | homeobox protein goosecoid |
| AS017254 | contig_1463 | hypothetical protein DMN91_010341 |
| AS017255 | contig_1463 | hypothetical protein B5V51_1084 |
| AS017256 | contig_1463 | NA |
| AS017249 | contig_1463 | hypothetical protein AB894_15215 |
| AS017250 | contig_1463 | uncharacterized protein LOC116770131 |
| AS017376 | contig_1535 | hypothetical protein evm_015021 |
| AS017389 | contig_1548 | hypothetical protein B5V51_239 |
| AS017403 | contig_1549 | cytochrome P450 |
| AS017404 | contig_1549 | cytochrome P450 |
| AS017405 | contig_1549 | hemicentin |
| AS017591 | contig_1631 | NA |
| AS017592 | contig_1631 | elongation of very long chain fatty acids protein 7 |
| AS017615 | contig_1664 | hypothetical protein B5V51_2691 |
| AS017616 | contig_1664 | uncharacterized protein LOC110384379 |
| AS017617 | contig_1664 | aminoacylase |
| AS017656 | contig_1664 | ABCC1 protein |
| AS017618 | contig_1664 | NADH dehydrogenase (ubiquinone) 1 subunit C2 |
| AS017619 | contig_1664 | hypothetical protein AB894_15215 |
| AS017632 | contig_1664 | tyrosine-protein kinase transmembrane receptor Ror |
| AS017633 | contig_1664 | hypothetical protein B5V51_3105 |
| AS017642 | contig_1664 | unnamed protein product |
| AS017700 | contig_1666 | NA |
| AS017701 | contig_1666 | NA |
| AS017785 | contig_1788 | hypothetical protein B5V51_13981 |
| AS017800 | contig_1845 | hypothetical protein KGM_211223 |
| AS017968 | contig_1889 | uncharacterized protein OBRU01_24794 |
| AS017969 | contig_1889 | NA |
| AS018262 | contig_1928 | uncharacterized protein LOC111362396 |
| AS018263 | contig_1928 | ionotropic receptor 3 |
| AS018442 | contig_1972 | ecdysone-responsive G-protein coupled protein-2 |
| AS018443 | contig_1972 | hypothetical protein B5V51_3838 |
| AS018477 | contig_2023 | short neuropeptide F isoform X2 |

Notes: NA (not applicable).

**Table S17. Genes of XJ selected region identified by *F*_ST_ and π between STC and XJ**

| **Gene ID** | **Contig** | **Annotation** |
| --- | --- | --- |
| AS000024 | contig_1 | hypothetical protein B5V51_12829 |
| AS000040 | contig_1 | hypothetical protein B5V51_10841 |
| AS000071 | contig_4 | uncharacterized protein LOC106140195 |
| AS000261 | contig_9 | uncharacterized protein LOC111356792 |
| AS000413 | contig_9 | estrogen sulfotransferase-like |
| AS000414 | contig_9 | sulfotransferase 1C4-like |
| AS000483 | contig_12 | uncharacterized protein LOC113231398 isoform X1 |
| AS000491 | contig_12 | NA |
| AS000544 | contig_26 | vacuolar protein sorting-associated protein 16 |
| AS001009 | contig_34 | Zinc finger MYM-type protein 1 |
| AS001010 | contig_34 | uncharacterized protein LOC106100064 |
| AS001089 | contig_34 | prickle |
| AS001174 | contig_34 | NA |
| AS001289 | contig_35 | Rho guanine nucleotide exchange factor 4 |
| AS001485 | contig_37 | NA |
| AS001486 | contig_37 | inner centromere protein A-like |
| AS001488 | contig_37 | NA |
| AS001493 | contig_37 | hypothetical protein B5V51_10841 |
| AS001496 | contig_37 | uncharacterized protein LOC113234039 |
| AS001497 | contig_37 | hypothetical protein evm_009240 |
| AS001507 | contig_37 | orexin receptor type 1-like isoform X1 |
| AS001511 | contig_37 | hypothetical protein AB894_15215 |
| AS001512 | contig_37 | RNA binding protein fox-1 |
| AS002162 | contig_58 | uncharacterized protein LOC113501675 |
| AS002170 | contig_58 | hypothetical protein B5X24_HaOG204312 |
| AS002296 | contig_64 | trypsin-1-like |
| AS002297 | contig_64 | NA |
| AS002393 | contig_65 | calcium-dependent protein kinase |
| AS002437 | contig_66 | NA |
| AS002642 | contig_71 | uncharacterized protein LOC111359584 |
| AS002643 | contig_71 | guanylate cyclase |
| AS002644 | contig_71 | guanylate cyclase |
| AS002712 | contig_76 | oxysterol-binding protein-related protein 3/6/7 |
| AS002713 | contig_76 | uncharacterized protein LOC106142263 |
| AS002714 | contig_76 | uncharacterized protein LOC111349335 |
| AS002716 | contig_76 | hypothetical protein OBRU01_03615 |
| AS002745 | contig_78 | transcription factor IIIB 90 kDa subunit |
| AS003037 | contig_82 | hypothetical protein B5V51_14853 |
| AS003110 | contig_82 | unconventional myosin-XVIIIa isoform X3 |
| AS003134 | contig_82 | uncharacterized protein LOC113493276 |
| AS003181 | contig_82 | proclotting enzyme-like isoform X8 |
| AS003291 | contig_82 | uncharacterized protein LOC110370955 |
| AS003292 | contig_82 | uncharacterized protein LOC110370955 |
| AS003293 | contig_82 | uncharacterized protein LOC111360097 |
| AS003412 | contig_92 | NA |
| AS003413 | contig_92 | NA |
| AS003497 | contig_94 | 3-methylcrotonyl-CoA carboxylase alpha subunit |
| AS003706 | contig_101 | uncharacterized protein K02A2.6-like |
| AS003952 | contig_113 | serine/threonine-protein kinase MRCK |
| AS003953 | contig_113 | uncharacterized protein LOC113508094 |
| AS004058 | contig_114 | hypothetical protein B5X24_HaOG214358 |
| AS004060 | contig_114 | proton-coupled amino acid transporter-like protein pathetic |
| AS004248 | contig_129 | oligoribonuclease |
| AS004249 | contig_129 | LETM1 and EF-hand domain-containing protein 1 |
| AS004392 | contig_132 | hypothetical protein B5X24_HaOG202753 |
| AS004452 | contig_134 | uncharacterized protein LOC105383034 |
| AS004632 | contig_138 | peroxidase-like |
| AS004633 | contig_138 | NA |
| AS004634 | contig_138 | mediator of RNA polymerase II transcription subunit 4 |
| AS004635 | contig_138 | putative inner dynein arm light chain |
| AS004636 | contig_138 | protein lap4 isoform X12 |
| AS004661 | contig_138 | hypothetical protein AB894_15215 |
| AS004762 | contig_139 | mitogen-activated protein kinase 5 |
| AS004859 | contig_140 | methylenetetrahydrofolate dehydrogenase (NADP+) |
| AS005019 | contig_140 | multidrug resistance-associated protein 4 isoform X2 |
| AS005143 | contig_149 | trehalose 6-phosphate synthase/phosphatase |
| AS005144 | contig_149 | protein transport protein YIP1 |
| AS005145 | contig_149 | anaphase-promoting complex subunit 11 |
| AS005146 | contig_149 | hypothetical protein B5V51_14010 |
| AS005147 | contig_149 | putative uncharacterized protein DDB_G0291608 isoform X1 |
| AS005189 | contig_151 | glutathione S-transferase |
| AS005212 | contig_151 | hypothetical protein B5V51_14045, partial |
| AS005218 | contig_151 | UNC93-like protein |
| AS005319 | contig_152 | hypothetical protein B5X24_HaOG208307 |
| AS005320 | contig_152 | chymotrypsin-like elastase family member 2A |
| AS005343 | contig_152 | putative ATP-dependent RNA helicase DHX30 |
| AS005355 | contig_152 | glucuronosyltransferase |
| AS005412 | contig_153 | oxysterol-binding protein-related protein 9 |
| AS005566 | contig_154 | uncharacterized protein LOC106132896 |
| AS005652 | contig_154 | phosphatidylinositol glycan |
| AS005653 | contig_154 | facilitated trehalose transporter |
| AS005819 | contig_159 | glycogen(starch) synthase |
| AS005838 | contig_159 | uncharacterized protein LOC113498611 |
| AS005878 | contig_159 | histone-lysine N-methyltransferase SETMAR |
| AS005906 | contig_160 | calcium/calmodulin-dependent 3',5'-cyclic nucleotide phosphodiesterase |
| AS006036 | contig_162 | hypothetical protein B5V51_10303 |
| AS006100 | contig_163 | trehalase-2 |
| AS006114 | contig_167 | Probable RNA-directed DNA polymerase from transposon X-element |
| AS006145 | contig_167 | nicotinate phosphoribosyltransferase |
| AS006146 | contig_167 | gastrula zinc finger protein XlCGF46.1-like isoform X1 |
| AS006147 | contig_167 | transcriptional coactivator YAP1 |
| AS006179 | contig_168 | NA |
| AS006180 | contig_168 | inhibitor of apoptosis protein |
| AS006181 | contig_168 | DNA mismatch repair protein MSH2 |
| AS006305 | contig_169 | fatty acid synthase |
| AS006392 | contig_171 | phytanoyl-CoA dioxygenase domain-containing protein 1-like |
| AS006396 | contig_171 | coiled-coil domain-containing protein AGAP005037 |
| AS006422 | contig_171 | hypothetical protein B5V51_12365 |
| AS006433 | contig_171 | succinate dehydrogenase (ubiquinone) cytochrome b560 subunit |
| AS006441 | contig_171 | hypothetical protein B5X24_HaOG212193 |
| AS006442 | contig_171 | uncharacterized protein LOC106142263 |
| AS006472 | contig_171 | protein furry |
| AS006473 | contig_171 | somatostatin receptor 2 |
| AS006490 | contig_171 | sensory neuron membrane protein 2 |
| AS006648 | contig_171 | Kip1 ubiquitination-promoting complex protein 1 |
| AS006804 | contig_171 | tyrosine-protein phosphatase non-receptor type 1 |
| AS006810 | contig_171 | titin homolog |
| AS006811 | contig_171 | uncharacterized protein LOC111355311 |
| AS006812 | contig_171 | NA |
| AS006815 | contig_171 | uncharacterized protein LOC111360635 |
| AS006816 | contig_171 | pyruvate dehydrogenase phosphatase regulatory subunit |
| AS006877 | contig_174 | zinc finger protein 107-like |
| AS006997 | contig_174 | MFS transporter |
| AS007096 | contig_174 | Arf-GAP with Rho-GAP domain |
| AS007100 | contig_174 | hypothetical protein B5X24_HaOG202088 |
| AS007131 | contig_175 | uncharacterized protein LOC111358654 |
| AS007132 | contig_175 | phosphorylase kinase alpha/beta subunit |
| AS007203 | contig_177 | gustatory receptor |
| AS007238 | contig_183 | F-type H+-transporting ATPase subunit O |
| AS007239 | contig_183 | C-Myc-binding protein |
| AS007240 | contig_183 | hypothetical protein B5V51_5404 |
| AS007244 | contig_183 | uncharacterized protein LOC113505275 |
| AS007572 | contig_197 | hypothetical protein B5V51_4040 |
| AS007620 | contig_198 | Ras-related GTP-binding protein C/D |
| AS007678 | contig_201 | NA |
| AS008021 | contig_207 | neither inactivation nor afterpotential protein G |
| AS008082 | contig_208 | histidine-rich glycoprotein-like |
| AS008083 | contig_208 | pupal cuticle protein Edg-84A-like |
| AS008108 | contig_208 | hypothetical protein B5X24_HaOG216945 |
| AS008379 | contig_218 | gustatory receptor |
| AS008380 | contig_218 | uncharacterized protein LOC111357782 |
| AS008505 | contig_219 | unnamed protein product |
| AS008506 | contig_219 | NA |
| AS008531 | contig_219 | uncharacterized protein LOC112044007 |
| AS008532 | contig_219 | disks large protein 1 |
| AS008679 | contig_226 | transmembrane protease serine 12 |
| AS008680 | contig_226 | Uncharacterized protein LOC110384122 |
| AS008757 | contig_231 | cGMP-dependent protein kinase 1 |
| AS008787 | contig_235 | lactase-phlorizin hydrolase |
| AS008788 | contig_235 | hypothetical protein B5V51_9637 |
| AS008805 | contig_235 | neuropilin and tolloid-like protein 2 |
| AS009034 | contig_237 | pickpocket protein 28-like |
| AS009038 | contig_237 | NA |
| AS009039 | contig_237 | RNA-directed DNA polymerase |
| AS009080 | contig_238 | putative odorant receptor |
| AS009086 | contig_238 | Ig-like and fibronectin type-III domain-containing protein 2 isoform X3 |
| AS009460 | contig_245 | hypothetical protein B5X24_HaOG210857 |
| AS009848 | contig_251 | solute carrier family 35 (adenosine 3'-phospho 5'-phosphosulfate transporter) |
| AS009849 | contig_251 | NA |
| AS009859 | contig_252 | uncharacterized protein LOC113240557 |
| AS009936 | contig_256 | hypothetical protein B5X24_HaOG205555 |
| AS009937 | contig_256 | succinate dehydrogenase (ubiquinone) flavoprotein subunit |
| AS010006 | contig_258 | aldo-keto reductase AKR2E4-like isoform X1 |
| AS010033 | contig_259 | hypothetical protein B5V51_14410 |
| AS010034 | contig_259 | RNA-directed DNA polymerase |
| AS010035 | contig_259 | phosphoinositide-3-kinase |
| AS010036 | contig_259 | NA |
| AS010037 | contig_259 | uncharacterized protein LOC111355694 |
| AS010228 | contig_265 | carboxylesterase type B |
| AS010355 | contig_270 | NA |
| AS010356 | contig_270 | NA |
| AS010371 | contig_270 | NA |
| AS010372 | contig_270 | uncharacterized protein LOC112043017 |
| AS010373 | contig_270 | hypothetical protein evm_006790 |
| AS010374 | contig_270 | signal transducer and activator of transcription 5B |
| AS010432 | contig_280 | Hermansky-Pudlak syndrome 1 protein |
| AS010433 | contig_280 | dihydroorotate dehydrogenase |
| AS010434 | contig_280 | hexokinase |
| AS010435 | contig_280 | NA |
| AS010603 | contig_286 | CLIP domain-containing serine protease 2-like |
| AS010604 | contig_286 | fibrinogen silencer-binding protein-like |
| AS010605 | contig_286 | putative nuclease HARBI1 |
| AS010606 | contig_286 | transmembrane protease serine 9 |
| AS010625 | contig_288 | guanylate cyclase 2F |
| AS010626 | contig_288 | NA |
| AS010627 | contig_288 | carboxylesterase |
| AS010715 | contig_293 | NA |
| AS010716 | contig_293 | uncharacterized protein LOC116770084 |
| AS010908 | contig_308 | NA |
| AS010909 | contig_308 | NA |
| AS010910 | contig_308 | uncharacterized protein LOC110370346 |
| AS011035 | contig_319 | estrogen-related receptor ERR |
| AS011484 | contig_336 | uncharacterized protein LOC116770084 |
| AS011485 | contig_336 | uncharacterized protein LOC115454967 |
| AS011663 | contig_338 | fatty alcohol acetyltransferase |
| AS011664 | contig_338 | fatty alcohol acetyltransferase |
| AS011800 | contig_339 | hypothetical protein B5X24_HaOG213397 |
| AS011893 | contig_343 | ankyrin repeat domain-containing protein 50 isoform X3 |
| AS012020 | contig_345 | uncharacterized protein LOC106142263 |
| AS012107 | contig_352 | ATP-binding cassette |
| AS012108 | contig_352 | protein phosphatase 1 regulatory subunit 3A/B/C/D/E |
| AS012154 | contig_353 | acetate esterase 13 |
| AS012167 | contig_359 | serine protease snake-like |
| AS012170 | contig_359 | NA |
| AS012175 | contig_359 | solute carrier family 41 |
| AS012196 | contig_363 | uncharacterized protein LOC111364164 |
| AS012223 | contig_363 | SWI/SNF-related matrix-associated actin-dependent regulator of chromatin subfamily A member 2/4 |
| AS012279 | contig_365 | glutamate receptor-interacting protein 2 isoform X6 |
| AS012311 | contig_372 | Retrovirus-related Pol polyprotein from transposon TNT 1-94 |
| AS012312 | contig_372 | ATP-binding cassette |
| AS012321 | contig_372 | glycine cleavage system H protein |
| AS012322 | contig_372 | ATP synthase subunit gamma, mitochondrial-like isoform X1 |
| AS012323 | contig_372 | protein henna |
| AS012328 | contig_372 | uncharacterized protein LOC111349843 |
| AS012329 | contig_372 | G-protein signaling modulator 2 |
| AS012330 | contig_372 | NA |
| AS012331 | contig_372 | unnamed protein product |
| AS012332 | contig_372 | inner centromere protein A-like |
| AS012333 | contig_372 | hypothetical protein B5X24_HaOG207301 |
| AS012449 | contig_378 | uncharacterized protein LOC116853148 isoform X1 |
| AS012460 | contig_378 | uncharacterized protein LOC113522127 |
| AS012477 | contig_378 | uncharacterized protein LOC113501428 |
| AS012608 | contig_430 | hypothetical protein B5X24_HaOG203342 |
| AS012860 | contig_462 | NA |
| AS012861 | contig_462 | uncharacterized protein LOC114351244 |
| AS012872 | contig_462 | gustatory receptor |
| AS012873 | contig_462 | hypothetical protein B5V51_10841 |
| AS013001 | contig_481 | formin-2-like, partial |
| AS013017 | contig_481 | nitric-oxide synthase |
| AS013370 | contig_505 | odorant binding protein |
| AS013371 | contig_505 | odorant binding protein |
| AS013372 | contig_505 | odorant binding protein |
| AS013962 | contig_561 | NA |
| AS014016 | contig_566 | uncharacterized protein LOC111357782 |
| AS014022 | contig_566 | prickle |
| AS014180 | contig_573 | glycogen phosphorylase |
| AS014250 | contig_577 | hypothetical protein B5V51_14628 |
| AS014251 | contig_577 | hypothetical protein EVAR_24085_1 |
| AS014261 | contig_577 | guanine nucleotide exchange factor DBS-like isoform X1 |
| AS014309 | contig_601 | dual specificity tyrosine-phosphorylation-regulated kinase 2/3/4 |
| AS014395 | contig_604 | hypothetical protein B5V51_10846 |
| AS014541 | contig_609 | uncharacterized protein LOC106710158 |
| AS014544 | contig_609 | uncharacterized protein LOC114240369 |
| AS014545 | contig_609 | hypothetical protein evm_013355 |
| AS014649 | contig_629 | hypothetical protein B5V51_8457 |
| AS014650 | contig_629 | thyroid transcription factor 1-associated protein 26 homolog |
| AS014651 | contig_629 | hypothetical protein B5V51_8460 |
| AS014652 | contig_629 | bestrophin 1a |
| AS014784 | contig_633 | WD and tetratricopeptide repeats protein 1 |
| AS014860 | contig_635 | cytochrome c1-2, heme protein, mitochondrial |
| AS014861 | contig_635 | hypothetical protein B5V51_14976 |
| AS014862 | contig_635 | protein LSM14 homolog A |
| AS014863 | contig_635 | kelch-like protein 10 |
| AS014999 | contig_654 | NA |
| AS015009 | contig_654 | uncharacterized protein LOC110374271 |
| AS015010 | contig_654 | NEDD8-activating enzyme E1 catalytic subunit |
| AS015011 | contig_654 | uncharacterized protein LOC110374329 |
| AS015012 | contig_654 | F-type H+-transporting ATPase subunit beta |
| AS015100 | contig_657 | NA |
| AS015105 | contig_657 | NA |
| AS015200 | contig_683 | uncharacterized protein LOC110381480 |
| AS015303 | contig_711 | NA |
| AS015308 | contig_711 | methylenetetrahydrofolate dehydrogenase (NADP+) |
| AS015460 | contig_765 | hypothetical protein B5V51_11515 |
| AS015590 | contig_783 | uncharacterized protein LOC111359490 |
| AS015619 | contig_787 | putative inorganic phosphate cotransporter |
| AS015887 | contig_868 | hypothetical protein B5V51_14909 |
| AS016141 | contig_933 | forkhead box protein O3 |
| AS016281 | contig_969 | mediator of RNA polymerase II transcription subunit 1 |
| AS016538 | contig_1078 | desaturase |
| AS016539 | contig_1078 | NA |
| AS016582 | contig_1095 | NA |
| AS016744 | contig_1110 | tripartite motif-containing protein 45 |
| AS017699 | contig_1666 | uncharacterized protein LOC114355925 |
| AS017783 | contig_1788 | ATP synthase subunit alpha, mitochondrial |
| AS017784 | contig_1788 | hypothetical protein B5V51_13982 |
| AS017797 | contig_1837 | proline-rich protein 36 |
| AS017824 | contig_1873 | uncharacterized protein LOC116770131 |
| AS017825 | contig_1873 | hypothetical protein B5X24_HaOG204786 |
| AS017826 | contig_1873 | NA |
| AS017886 | contig_1886 | uncharacterized protein K02A2.6-like isoform X1 |
| AS017928 | contig_1887 | prolactin regulatory element-binding protein |
| AS017929 | contig_1887 | nuclear factor of activated T-cells 5 |
| AS017981 | contig_1892 | MOXD1 homolog 1-like |
| AS017982 | contig_1892 | carboxylesterase 1 |
| AS018341 | contig_1938 | NA |
| AS018342 | contig_1938 | NA |
| AS018443 | contig_1972 | hypothetical protein B5V51_3838 |
| AS018446 | contig_1978 | uncharacterized protein LOC111355046 |

Notes: NA (not applicable).

**Table S18. Genes of STC selected region identified by *F*_ST_ and π between STC and XJ**

| **Gene ID** | **Contig** | **Annotation** |
| --- | --- | --- |
| AS000190 | contig_4 | uncharacterized protein LOC113505673 isoform X1 |
| AS000191 | contig_4 | hypothetical protein B5X24_HaOG212902 |
| AS000457 | contig_10 | protein expanded |
| AS000458 | contig_10 | solute carrier family 3 (neutral and basic amino acid transporter) |
| AS000459 | contig_10 | fatty alcohol acetyltransferase |
| AS000469 | contig_10 | juvenile hormone acid methyltransferase |
| AS000470 | contig_10 | juvenile hormone acid methyltransferase |
| AS000514 | contig_25 | uncharacterized protein LOC111348539 |
| AS000517 | contig_25 | hypothetical protein B5V51_14463 |
| AS000518 | contig_25 | hypothetical protein B5V51_7957 |
| AS001326 | contig_36 | NA |
| AS001329 | contig_36 | NA |
| AS001410 | contig_37 | uncharacterized protein LOC110379930 |
| AS001437 | contig_37 | protein toll |
| AS001438 | contig_37 | orexin receptor type 1 |
| AS001451 | contig_37 | uncharacterized protein LOC111351052 |
| AS001464 | contig_37 | hypothetical protein evm_011754 |
| AS001467 | contig_37 | uncharacterized protein LOC114363995 |
| AS001468 | contig_37 | prickle |
| AS001481 | contig_37 | NA |
| AS001494 | contig_37 | orexin receptor type 1-like |
| AS001533 | contig_37 | uncharacterized protein LOC111350740 |
| AS001848 | contig_45 | PiggyBac transposable element-derived protein 3 |
| AS001974 | contig_57 | protein turtle isoform X2 |
| AS001975 | contig_57 | lipase member H-like |
| AS002004 | contig_57 | protocadherin Fat 4 |
| AS002046 | contig_58 | glutathione S-transferase |
| AS002144 | contig_58 | hypothetical protein B5V51_407 |
| AS002408 | contig_65 | uncharacterized protein LOC116158895 |
| AS002409 | contig_65 | NA |
| AS002689 | contig_74 | uncharacterized protein LOC113495257 |
| AS002690 | contig_74 | ORF B |
| AS002712 | contig_76 | oxysterol-binding protein-related protein 3/6/7 |
| AS002717 | contig_76 | WD and tetratricopeptide repeats protein 1 |
| AS002718 | contig_76 | NA |
| AS002727 | contig_76 | zinc finger protein ZFMSA12A-like isoform X1 |
| AS002951 | contig_82 | uncharacterized protein LOC111360005 |
| AS002952 | contig_82 | uncharacterized protein LOC110373676 |
| AS003383 | contig_86 | solute carrier family 4 (anion exchanger) |
| AS003384 | contig_86 | solute carrier family 4 (anion exchanger) |
| AS003385 | contig_86 | solute carrier family 4 (anion exchanger) |
| AS004055 | contig_114 | gastrin/cholecystokinin type B receptor-like isoform X1 |
| AS004057 | contig_114 | gastrin/cholecystokinin type B receptor-like |
| AS004401 | contig_132 | calsequestrin-1-like |
| AS004624 | contig_138 | uncharacterized protein LOC116770131 |
| AS004628 | contig_138 | hypothetical protein B5X24_HaOG212154 |
| AS005791 | contig_159 | lachesin |
| AS005892 | contig_159 | fatty-acyl CoA reductase 6 |
| AS005893 | contig_159 | uncharacterized protein LOC114358071 |
| AS005940 | contig_162 | ATP-binding cassette |
| AS006235 | contig_169 | cytochrome P450 |
| AS006236 | contig_169 | cytochrome P450 |
| AS006305 | contig_169 | fatty acid synthase |
| AS006362 | contig_169 | glucose dehydrogenase [FAD, quinone]-like |
| AS006396 | contig_171 | coiled-coil domain-containing protein AGAP005037 |
| AS006397 | contig_171 | NA |
| AS006402 | contig_171 | solute carrier family 35 (UDP-galactose transporter) |
| AS006403 | contig_171 | basement membrane-specific heparan sulfate proteoglycan core protein |
| AS006405 | contig_171 | NA |
| AS006406 | contig_171 | hypothetical protein evm_004341 |
| AS006407 | contig_171 | hypothetical protein B5X24_HaOG205330 |
| AS006649 | contig_171 | NA |
| AS006655 | contig_171 | uncharacterized protein LOC110374481 isoform X3 |
| AS006706 | contig_171 | hypothetical protein B5X24_HaOG214709 |
| AS006760 | contig_171 | hypothetical protein B5V51_4741 |
| AS006761 | contig_171 | peptidoglycan-recognition protein SB2-like isoform X1 |
| AS006790 | contig_171 | uncharacterized protein LOC111355311 |
| AS006837 | contig_171 | transmembrane channel-like protein 2 isoform X1 |
| AS007096 | contig_174 | Arf-GAP with Rho-GAP domain |
| AS007101 | contig_174 | protein dpy-30 |
| AS007102 | contig_174 | NA |
| AS007853 | contig_204 | uncharacterized protein LOC111356548 |
| AS007854 | contig_204 | probable aldehyde oxidase gad-3 |
| AS008146 | contig_214 | NA |
| AS008147 | contig_214 | RYamide receptor-like |
| AS008343 | contig_218 | unnamed protein product |
| AS008670 | contig_226 | hypothetical protein B5X24_HaOG201920 |
| AS009033 | contig_237 | pickpocket protein 28-like |
| AS009034 | contig_237 | pickpocket protein 28-like |
| AS009086 | contig_238 | Ig-like and fibronectin type-III domain-containing protein 2 isoform X3 |
| AS009091 | contig_238 | uncharacterized protein LOC110382069 |
| AS009092 | contig_238 | uncharacterized protein LOC114365243 |
| AS009093 | contig_238 | uncharacterized protein LOC113503959, partial |
| AS009094 | contig_238 | hypothetical protein B5X24_HaOG212193 |
| AS009095 | contig_238 | hypothetical protein B5V51_13147 |
| AS009546 | contig_250 | RNA-directed DNA polymerase from mobile element jockey-like |
| AS009606 | contig_250 | putative glycerol kinase 5 |
| AS009667 | contig_250 | farnesyl pyrophosphate synthase 2-like |
| AS010226 | contig_265 | acetylcholinesterase |
| AS010609 | contig_286 | transient receptor potential channel pyrexia |
| AS010610 | contig_286 | ubiquinone biosynthesis monooxygenase Coq6 |
| AS010778 | contig_300 | uncharacterized protein LOC106104121 |
| AS011306 | contig_327 | uncharacterized protein LOC114245972 |
| AS011378 | contig_330 | neuropeptide Y receptor |
| AS011379 | contig_330 | neuropeptide Y receptor |
| AS011479 | contig_336 | calcium-sensing receptor |
| AS011770 | contig_339 | serpin B8-like |
| AS011771 | contig_339 | male-enhanced antigen 1 |
| AS011772 | contig_339 | protein-L-isoaspartate(D-aspartate) O-methyltransferase |
| AS011773 | contig_339 | hypothetical protein B5V51_9932 |
| AS011774 | contig_339 | DNA repair endonuclease XPF |
| AS011775 | contig_339 | exportin-1 |
| AS011776 | contig_339 | katanin p80 WD40 repeat-containing subunit B1 |
| AS011777 | contig_339 | NA |
| AS011779 | contig_339 | putative endonuclease/reverse transcriptase |
| AS011786 | contig_339 | histidine-rich glycoprotein-like |
| AS011787 | contig_339 | histidine-rich glycoprotein-like |
| AS011788 | contig_339 | NA |
| AS011792 | contig_339 | E3 ubiquitin-protein ligase HECTD1 |
| AS011793 | contig_339 | cytoplasmic dynein 2 light intermediate chain 1 |
| AS011794 | contig_339 | tetratricopeptide repeat protein 8 |
| AS011795 | contig_339 | vacuolar protein sorting-associated protein 54 |
| AS011796 | contig_339 | exocyst complex component 3 |
| AS011798 | contig_339 | myb-like protein P |
| AS011799 | contig_339 | NA |
| AS012016 | contig_345 | protein toll |
| AS012186 | contig_363 | cytochrome P450 |
| AS012341 | contig_372 | hepatic leukemia factor |
| AS013121 | contig_487 | meso-butanediol dehydrogenase / (S,S)-butanediol dehydrogenase / diacetyl reductase |
| AS013122 | contig_487 | hypothetical protein B5V51_12826 |
| AS013123 | contig_487 | meso-butanediol dehydrogenase / (S,S)-butanediol dehydrogenase / diacetyl reductase |
| AS013124 | contig_487 | meso-butanediol dehydrogenase / (S,S)-butanediol dehydrogenase / diacetyl reductase |
| AS013506 | contig_512 | NA |
| AS013507 | contig_512 | uncharacterized protein LOC105669866 |
| AS013997 | contig_566 | NA |
| AS013998 | contig_566 | NA |
| AS014014 | contig_566 | endonuclease-reverse transcriptase |
| AS014021 | contig_566 | fatty acid synthase |
| AS014456 | contig_604 | reverse transcriptase |
| AS015101 | contig_657 | hypothetical protein B5X24_HaOG212154 |
| AS015102 | contig_657 | NA |
| AS015225 | contig_688 | NA |
| AS015226 | contig_688 | hypothetical protein B5V51_407 |
| AS015227 | contig_688 | hypothetical protein B5V51_407 |
| AS015228 | contig_688 | hypothetical protein B5V51_407 |
| AS015659 | contig_788 | hypothetical protein B5V51_13662 |
| AS015667 | contig_796 | cytochrome P450 |
| AS015668 | contig_796 | cytochrome P450 |
| AS015669 | contig_796 | cytochrome P450 |
| AS016126 | contig_932 | hypothetical protein B5V51_14479 |
| AS016127 | contig_932 | PITH domain-containing protein GA19395 |
| AS016128 | contig_932 | collagen |
| AS016359 | contig_1024 | parkin coregulated gene protein homolog |
| AS016535 | contig_1078 | uncharacterized protein LOC110371643 |
| AS016536 | contig_1078 | delta-9 desaturase 14-26 |
| AS016537 | contig_1078 | NA |
| AS016540 | contig_1078 | RNA binding protein fox-1 |
| AS016541 | contig_1078 | pyrokinin-1 receptor-like |
| AS016543 | contig_1078 | glutamate receptor 2 |
| AS017135 | contig_1374 | homeobox protein goosecoid |
| AS017136 | contig_1374 | homeobox protein goosecoid |
| AS017137 | contig_1374 | WW domain-binding protein 4 |
| AS017138 | contig_1374 | putative ATP-dependent RNA helicase me31b |
| AS017153 | contig_1400 | J domain-containing protein |
| AS017164 | contig_1401 | calcium/calmodulin-dependent protein kinase I |
| AS017241 | contig_1458 | serine/threonine-protein kinase MRCK |
| AS017246 | contig_1463 | hypothetical protein B5V51_2244 |
| AS017249 | contig_1463 | hypothetical protein AB894_15215 |
| AS017250 | contig_1463 | uncharacterized protein LOC116770131 |
| AS017254 | contig_1463 | hypothetical protein DMN91_010341 |
| AS017255 | contig_1463 | hypothetical protein B5V51_10841 |
| AS017256 | contig_1463 | NA |
| AS017403 | contig_1549 | cytochrome P450 |
| AS017404 | contig_1549 | cytochrome P450 |
| AS017405 | contig_1549 | hemicentin |
| AS017613 | contig_1664 | thyrostimulin beta-5 subunit-like |
| AS017615 | contig_1664 | hypothetical protein B5V51_2691 |
| AS017616 | contig_1664 | uncharacterized protein LOC110384379 |
| AS017617 | contig_1664 | unnamed protein product |
| AS017618 | contig_1664 | NADH dehydrogenase (ubiquinone) 1 subunit C2 |
| AS017619 | contig_1664 | hypothetical protein AB894_15215 |
| AS017623 | contig_1664 | facilitated trehalose transporter |
| AS017625 | contig_1664 | nuclear pore complex protein Nup53 |
| AS017626 | contig_1664 | uncharacterized protein LOC111348605 |
| AS017632 | contig_1664 | tyrosine-protein kinase transmembrane receptor |
| AS017633 | contig_1664 | hypothetical protein B5V51_3105 |
| AS017641 | contig_1664 | alpha-aminoadipic semialdehyde synthase |
| AS017642 | contig_1664 | unnamed protein product |
| AS017643 | contig_1664 | hypothetical protein B5V51_9870 |
| AS017651 | contig_1664 | hypothetical protein evm_010277 |
| AS017656 | contig_1664 | ABCC1 protein |
| AS017700 | contig_1666 | NA |
| AS017701 | contig_1666 | NA |
| AS018442 | contig_1972 | ecdysone-responsive G-protein coupled protein-2 |

Notes: NA (not applicable).

**Table S19. Genes of NEC selected region identified by *F*_ST_ and π between XJ and NEC**

| **Gene ID** | **Contig** | **Annotation** |
| --- | --- | --- |
| AS000036 | contig_1 | NA |
| AS000755 | contig_29 | Retrovirus-related Pol polyprotein from transposon TNT 1-94 |
| AS000756 | contig_29 | optic atrophy protein 1 |
| AS000867 | contig_31 | cytochrome P450 |
| AS001329 | contig_36 | NA |
| AS001438 | contig_37 | orexin receptor type 1 |
| AS001441 | contig_37 | hypothetical protein B5X24_HaOG201003 |
| AS001442 | contig_37 | uncharacterized protein LOC110374952 |
| AS001509 | contig_37 | uncharacterized protein LOC111355311 |
| AS001510 | contig_37 | NA |
| AS001833 | contig_45 | uncharacterized protein LOC110381944 |
| AS001834 | contig_45 | intraflagellar transport protein 46 |
| AS002003 | contig_57 | pancreatic lipase-related protein 3 |
| AS002399 | contig_65 | 27 kDa glycoprotein-like isoform X2 |
| AS002712 | contig_76 | oxysterol-binding protein-related protein 3/6/7 |
| AS002730 | contig_76 | uncharacterized protein LOC110384446 |
| AS003122 | contig_82 | cytochrome P450 |
| AS003123 | contig_82 | cytochrome P450 |
| AS003276 | contig_82 | peptidoglycan recognition protein D |
| AS003383 | contig_86 | solute carrier family 4 (anion exchanger) |
| AS003384 | contig_86 | solute carrier family 4 (anion exchanger) |
| AS003385 | contig_86 | solute carrier family 4 (anion exchanger) |
| AS003892 | contig_109 | pyruvate kinase |
| AS003893 | contig_109 | serine-protein kinase ATM |
| AS004192 | contig_123 | THO complex subunit 7 |
| AS004193 | contig_123 | phospholipase D3/4 |
| AS004497 | contig_135 | uncharacterized protein LOC110377940 |
| AS004503 | contig_135 | uncharacterized protein LOC111352141 |
| AS004510 | contig_135 | nose resistant to fluoxetine protein 6-like |
| AS004511 | contig_135 | nose resistant to fluoxetine protein 6-like |
| AS004931 | contig_140 | high affinity copper uptake protein 1-like |
| AS004932 | contig_140 | bromodomain-containing protein 3 |
| AS004986 | contig_140 | presenilins-associated rhomboid-like protein, mitochondrial |
| AS005099 | contig_142 | lysozyme-like |
| AS005100 | contig_142 | I-type lysozyme 2 |
| AS005159 | contig_149 | glyceronephosphate O-acyltransferase |
| AS005303 | contig_152 | nose resistant to fluoxetine protein 6-like |
| AS005791 | contig_159 | Lachesin |
| AS005892 | contig_159 | fatty-acyl CoA reductase 6 |
| AS005923 | contig_161 | elongation of very long chain fatty acids protein 7 |
| AS005929 | contig_162 | ATP-binding cassette |
| AS005930 | contig_162 | unnamed protein product |
| AS005991 | contig_162 | uncharacterized protein LOC111351402 |
| AS005992 | contig_162 | hypothetical protein B5V51_5265 |
| AS005993 | contig_162 | NA |
| AS006186 | contig_168 | glutamate receptor |
| AS007129 | contig_175 | adenylate cyclase 9 |
| AS007130 | contig_175 | hypothetical protein evm_013249 |
| AS007131 | contig_175 | uncharacterized protein LOC111358654 |
| AS007241 | contig_183 | hypothetical protein B5X24_HaOG205854 |
| AS007243 | contig_183 | NA |
| AS007915 | contig_204 | xanthine dehydrogenase/oxidase |
| AS008129 | contig_208 | piggyBac transposable element-derived protein 3-like |
| AS008381 | contig_218 | NA |
| AS008519 | contig_219 | uncharacterized protein LOC113502962 isoform X1 |
| AS008805 | contig_235 | neuropilin and tolloid-like protein 2 |
| AS009177 | contig_239 | Octopamine receptor |
| AS009896 | contig_254 | cytochrome P450 |
| AS009922 | contig_256 | uncharacterized protein LOC110371998 |
| AS010082 | contig_261 | uncharacterized protein LOC111349511 |
| AS010408 | contig_274 | hypothetical protein B5V51_12759 |
| AS010778 | contig_300 | uncharacterized protein LOC106104121 |
| AS011179 | contig_325 | juvenile hormone epoxide hydrolase |
| AS011180 | contig_325 | juvenile hormone epoxide hydrolase |
| AS011411 | contig_333 | mitogen-activated protein kinase 14B |
| AS011412 | contig_333 | NADH dehydrogenase (ubiquinone) 1 beta subcomplex subunit 6 |
| AS011413 | contig_333 | 18S rRNA-dimethyltransferase |
| AS011414 | contig_333 | transportin-1 |
| AS011503 | contig_336 | ncharacterized protein LOC114245972 |
| AS011504 | contig_336 | phospholipid-translocating ATPase |
| AS011505 | contig_336 | Rab GDP dissociation inhibitor |
| AS011506 | contig_336 | acyl-CoA synthetase |
| AS011541 | contig_337 | microtubule-associated serine/threonine kinase |
| AS011542 | contig_337 | hypothetical protein B5V51_14410 |
| AS011661 | contig_338 | fatty alcohol acetyltransferase |
| AS011767 | contig_339 | uncharacterized protein LOC111362874 |
| AS011768 | contig_339 | uncharacterized protein LOC114242079 |
| AS011769 | contig_339 | unnamed protein product |
| AS011770 | contig_339 | serpin B8-like |
| AS011774 | contig_339 | DNA repair endonuclease XPF |
| AS011775 | contig_339 | exportin-1 |
| AS011776 | contig_339 | hypothetical protein B5V51_5974 |
| AS011777 | contig_339 | NA |
| AS011784 | contig_339 | uncharacterized protein LOC111348596 |
| AS011872 | contig_340 | histone acetyltransferase MYST2 |
| AS012002 | contig_345 | carbonic anhydrase |
| AS012009 | contig_345 | tachykinin-like receptor |
| AS012301 | contig_372 | phytanoyl-CoA dioxygenase domain-containing protein 1 |
| AS012302 | contig_372 | NA |
| AS012303 | contig_372 | NA |
| AS013373 | contig_505 | odorant binding protein |
| AS013374 | contig_505 | JHBP domain-containing protein |
| AS013375 | contig_505 | hypothetical protein B5X24_HaOG207672 |
| AS013376 | contig_505 | NA |
| AS013426 | contig_507 | phospholipid scramblase 2-like |
| AS013427 | contig_507 | phospholipid scramblase 2-like |
| AS013428 | contig_507 | phospholipid scramblase 2-like |
| AS013453 | contig_508 | NA |
| AS013512 | contig_512 | Retrotransposable element |
| AS013513 | contig_512 | sulfite oxidase |
| AS013705 | contig_530 | homeobox protein HEX |
| AS014164 | contig_573 | NA |
| AS014169 | contig_573 | 5'-AMP-activated protein kinase |
| AS014170 | contig_573 | proton-coupled amino acid transporter-like protein pathetic isoform X1 |
| AS014656 | contig_629 | uncharacterized protein LOC110378242 |
| AS014657 | contig_629 | uncharacterized protein LOC114361369 |
| AS015066 | contig_655 | uncharacterized protein LOC111364526 |
| AS015614 | contig_787 | Retrovirus-related Pol polyprotein from transposon TNT 1-94-like Protein |
| AS016048 | contig_919 | MFS transporter |
| AS016130 | contig_932 | collagen alpha-1(IV) chain |
| AS016533 | contig_1078 | hypothetical protein B5V51_2740 |
| AS016540 | contig_1078 | RNA binding protein fox-1 |
| AS016767 | contig_1139 | hypothetical protein B5V51_14467 |
| AS017013 | contig_1286 | cytochrome P450 |
| AS017134 | contig_1368 | uncharacterized protein LOC111362874 isoform X1 |
| AS017136 | contig_1374 | unnamed protein product |
| AS017137 | contig_1374 | WW domain-binding protein 4 |
| AS017138 | contig_1374 | ATP-dependent RNA helicase DDX6/DHH1 |
| AS017157 | contig_1400 | uncharacterized protein LOC114352369 |
| AS017158 | contig_1400 | hypothetical protein B5V51_13622 |
| AS017159 | contig_1400 | T-cell activation inhibitor, mitochondrial-like |
| AS017581 | contig_1624 | hypothetical protein B5X24_HaOG217181 |
| AS017590 | contig_1631 | elongation of very long chain fatty acids protein 7 |
| AS018339 | contig_1938 | NA |

Notes: NA (not applicable).

**Table S20. Genes of XJ selected region identified by *F*_ST_ and π between XJ and NEC**

| **Gene ID** | **Contig** | **Annotation** |
| --- | --- | --- |
| AS000413 | contig_9 | estrone sulfotransferase |
| AS000414 | contig_9 | sulfotransferase 1C4-like |
| AS000509 | contig_24 | NA |
| AS000510 | contig_24 | hypothetical protein B5V51_8355 |
| AS000511 | contig_24 | uncharacterized protein LOC110380247 |
| AS000544 | contig_26 | vacuolar protein sorting-associated protein 16 |
| AS000701 | contig_28 | Retrovirus-related Pol polyprotein from transposon TNT 1-94 |
| AS000726 | contig_29 | uncharacterized protein LOC110384684 |
| AS000727 | contig_29 | chitinase |
| AS000738 | contig_29 | NA |
| AS000851 | contig_31 | reverse transcriptase |
| AS001004 | contig_34 | NA |
| AS001005 | contig_34 | uncharacterized protein K02A2.6-like |
| AS001006 | contig_34 | proprotein convertase subtilisin/kexin type 5 |
| AS001009 | contig_34 | Zinc finger MYM-type protein 1 |
| AS001010 | contig_34 | uncharacterized protein LOC106100064 |
| AS001089 | contig_34 | prickle |
| AS001090 | contig_34 | uncharacterized protein LOC114355939 |
| AS001174 | contig_34 | NA |
| AS001493 | contig_37 | hypothetical protein B5V51_10841 |
| AS001511 | contig_37 | hypothetical protein AB894_15215 |
| AS001512 | contig_37 | RNA binding protein fox-1 |
| AS001540 | contig_37 | hypothetical protein B5V51_5113 |
| AS001602 | contig_40 | ATP-binding cassette |
| AS001603 | contig_40 | uncharacterized protein LOC105841589 |
| AS001604 | contig_40 | NA |
| AS001605 | contig_40 | cell division control protein 7 |
| AS001701 | contig_40 | hypothetical protein EVAR_1020_1 |
| AS001702 | contig_40 | uncharacterized protein LOC110371536 |
| AS001762 | contig_41 | neurobeachin |
| AS001786 | contig_41 | yemanuclein-like isoform X1 |
| AS001976 | contig_57 | hrp65 protein-like |
| AS001977 | contig_57 | uncharacterized protein KIAA1143 homolog |
| AS001978 | contig_57 | transforming growth factor beta regulator 1 |
| AS001979 | contig_57 | menin |
| AS001980 | contig_57 | NA |
| AS001981 | contig_57 | hypothetical protein B5V51_5309 |
| AS002054 | contig_58 | Dynein heavy chain 3, axonemal |
| AS002162 | contig_58 | exosome complex component RRP4 |
| AS002167 | contig_58 | synapse-associated protein of 47 kDa isoform X4 |
| AS002203 | contig_62 | hypothetical protein B5X24_HaOG209551 |
| AS002275 | contig_64 | NA |
| AS002290 | contig_64 | hypothetical protein EVAR_35820_1 |
| AS002423 | contig_66 | phosphopantothenate-cysteine ligase |
| AS002713 | contig_76 | uncharacterized protein LOC106142263 |
| AS002714 | contig_76 | uncharacterized protein LOC111349335 |
| AS002716 | contig_76 | hypothetical protein OBRU01_03615 |
| AS002745 | contig_78 | transcription factor IIIB 90 kDa subunit |
| AS002803 | contig_78 | synaptogenesis protein syg-2-like |
| AS002926 | contig_82 | alpha-ketoglutarate-dependent dioxygenase alkB homolog 7 |
| AS002953 | contig_82 | hypothetical protein B5V51_13448 |
| AS003013 | contig_82 | NA |
| AS003045 | contig_82 | apyrase-like |
| AS003286 | contig_82 | uncharacterized protein LOC110370955 |
| AS003292 | contig_82 | uncharacterized protein LOC110370955 |
| AS003293 | contig_82 | uncharacterized protein LOC111360097 |
| AS003425 | contig_92 | hypothetical protein B5V51_9932 |
| AS003536 | contig_96 | uncharacterized protein LOC111355311 |
| AS003597 | contig_96 | uncharacterized protein LOC110374333 isoform X2 |
| AS003603 | contig_96 | sphingomyelin phosphodiesterase acid-like 3 |
| AS003897 | contig_109 | hypothetical protein B5X24_HaOG207692 |
| AS003958 | contig_113 | endonuclease-reverse transcriptase |
| AS004175 | contig_122 | hypothetical protein B5V51_5113 |
| AS004335 | contig_129 | uncharacterized protein LOC111357516 |
| AS004349 | contig_129 | uncharacterized protein LOC110377482 |
| AS004407 | contig_134 | unnamed protein product |
| AS004411 | contig_134 | uncharacterized protein LOC115454558 |
| AS004468 | contig_134 | uncharacterized protein LOC101736981 isoform X1 |
| AS004541 | contig_138 | suppressor of lurcher protein 1 |
| AS004562 | contig_138 | NA |
| AS004630 | contig_138 | peroxidase |
| AS004662 | contig_138 | NA |
| AS004665 | contig_138 | uncharacterized protein LOC114362701 |
| AS005019 | contig_140 | multidrug resistance-associated protein 4 isoform X2 |
| AS005320 | contig_152 | chymotrypsin-like elastase family member 2A |
| AS005351 | contig_152 | intermediate filament protein if |
| AS005355 | contig_152 | glucuronosyltransferase |
| AS005435 | contig_153 | uncharacterized protein LOC113225924 |
| AS005499 | contig_153 | L-2-hydroxycarboxylate dehydrogenase (NAD+) |
| AS005566 | contig_154 | uncharacterized protein LOC106132896 |
| AS005838 | contig_159 | uncharacterized protein LOC113498611 |
| AS005878 | contig_159 | histone-lysine N-methyltransferase SETMAR |
| AS006105 | contig_163 | hypothetical protein B5X24_HaOG212193 |
| AS006179 | contig_168 | NA |
| AS006221 | contig_169 | serine/threonine-protein phosphatase PP1 catalytic subunit |
| AS006226 | contig_169 | proline dehydrogenase |
| AS006305 | contig_169 | fatty acid synthase |
| AS006396 | contig_171 | coiled-coil domain-containing protein AGAP005037 |
| AS006400 | contig_171 | piggyBac transposable element-derived protein 3-like |
| AS006401 | contig_171 | SRC kinase signaling inhibitor 1 |
| AS006403 | contig_171 | basement membrane-specific heparan sulfate proteoglycan core protein |
| AS006423 | contig_171 | spastic paraplegia 7 |
| AS006473 | contig_171 | somatostatin receptor 2 |
| AS006490 | contig_171 | sensory neuron membrane protein 2 |
| AS006516 | contig_171 | solute carrier family 7 (cationic amino acid transporter) |
| AS006517 | contig_171 | uncharacterized protein LOC114355921 |
| AS006563 | contig_171 | EF-hand calcium-binding domain-containing protein 1-like |
| AS006650 | contig_171 | hypothetical protein B5V51_1187 |
| AS006725 | contig_171 | MAGUK p55 subfamily member 5 |
| AS006726 | contig_171 | insulin-like growth factor 2 mRNA-binding protein 1 |
| AS006737 | contig_171 | craniofacial development protein 2-like |
| AS006738 | contig_171 | endonuclease-reverse transcriptase |
| AS006796 | contig_171 | NA |
| AS006797 | contig_171 | NA |
| AS006804 | contig_171 | tyrosine-protein phosphatase non-receptor type 1 |
| AS006812 | contig_171 | NA |
| AS006877 | contig_174 | zinc finger protein 107 |
| AS006878 | contig_174 | tyrosine-protein kinase Abl |
| AS007197 | contig_176 | NA |
| AS007203 | contig_177 | gustatory receptor |
| AS007569 | contig_197 | uncharacterized protein LOC111349371 |
| AS007570 | contig_197 | uncharacterized protein LOC111357833 |
| AS007572 | contig_197 | hypothetical protein B5V51_4040 |
| AS007620 | contig_198 | Ras-related GTP-binding protein C/D |
| AS007621 | contig_198 | hypothetical protein B5V51_13509 |
| AS007622 | contig_198 | GPI transamidase component PIG-T |
| AS007623 | contig_198 | ubiquitin carboxyl-terminal hydrolase L3 |
| AS007624 | contig_198 | major facilitator superfamily domain-containing protein 12-like isoform X1 |
| AS007661 | contig_201 | uncharacterized protein LOC111357967 |
| AS007662 | contig_201 | hypothetical protein B5X24_HaOG212973 |
| AS007663 | contig_201 | uncharacterized protein LOC111357967 |
| AS007882 | contig_204 | uncharacterized protein LOC111356386 isoform X1 |
| AS007901 | contig_204 | synaptic vesicle glycoprotein 2B |
| AS008140 | contig_211 | NA |
| AS008240 | contig_216 | uncharacterized protein LOC111357782 |
| AS008347 | contig_218 | hypothetical protein B5V51_10841 |
| AS008505 | contig_219 | unnamed protein product |
| AS008506 | contig_219 | NA |
| AS008507 | contig_219 | protein toll |
| AS008531 | contig_219 | uncharacterized protein LOC112044007 |
| AS008532 | contig_219 | disks large protein 1 |
| AS008573 | contig_220 | hypothetical protein B5V51_3220 |
| AS008679 | contig_226 | transmembrane protease serine 12 |
| AS008685 | contig_226 | RYamide receptor-like |
| AS008870 | contig_235 | secretory phospholipase A2 |
| AS008871 | contig_235 | Rad and Gem related GTP binding protein 1 |
| AS008874 | contig_235 | Rad and Gem related GTP binding protein 1 |
| AS009080 | contig_238 | putative odorant receptor |
| AS009104 | contig_238 | NA |
| AS009187 | contig_239 | dedicator of cytokinesis protein 1 isoform X3 |
| AS009279 | contig_240 | organic cation/carnitine transporter 7-like isoform X1 |
| AS009291 | contig_240 | growth blocking peptide binding protein |
| AS009323 | contig_240 | hypothetical protein B5V51_490 |
| AS009434 | contig_244 | unnamed protein product |
| AS009437 | contig_244 | fatty acid synthase |
| AS009460 | contig_245 | hypothetical protein B5X24_HaOG210857 |
| AS009461 | contig_245 | hypothetical protein B5X24_HaOG210857 |
| AS009520 | contig_247 | NA |
| AS009567 | contig_250 | NA |
| AS009818 | contig_251 | uncharacterized protein LOC111361297 |
| AS009819 | contig_251 | hypothetical protein B5V51_679 |
| AS009902 | contig_254 | RNA-directed DNA polymerase |
| AS009903 | contig_254 | RNA-directed DNA polymerase |
| AS009904 | contig_254 | NA |
| AS009905 | contig_254 | hypothetical protein AB894_15215 |
| AS009917 | contig_256 | NA |
| AS009918 | contig_256 | NA |
| AS009937 | contig_256 | succinate dehydrogenase (ubiquinone) flavoprotein subunit |
| AS010061 | contig_260 | hypothetical protein B5V51_2600 |
| AS010067 | contig_260 | hypothetical protein B5X24_HaOG209515 |
| AS010071 | contig_261 | hypothetical protein B5V51_13147 |
| AS010076 | contig_261 | glutathione S-transferase |
| AS010228 | contig_265 | carboxylesterase type B |
| AS010343 | contig_270 | NA |
| AS010371 | contig_270 | NA |
| AS010372 | contig_270 | uncharacterized protein LOC112043017 |
| AS010373 | contig_270 | sentrin-specific protease 1 |
| AS010432 | contig_280 | Hermansky-Pudlak syndrome 1 protein |
| AS010433 | contig_280 | dihydroorotate dehydrogenase |
| AS010434 | contig_280 | hexokinase |
| AS010459 | contig_280 | hypothetical protein B5V51_2811 |
| AS010460 | contig_280 | RNA binding protein fox-1 |
| AS010551 | contig_286 | hypothetical protein B5V51_461 |
| AS010676 | contig_288 | hypothetical protein B5V51_5825 |
| AS010681 | contig_288 | protein real-time |
| AS010682 | contig_288 | uncharacterized protein LOC110377889 |
| AS010721 | contig_293 | DNA excision repair protein ERCC-3 |
| AS010857 | contig_308 | glucuronosyltransferase |
| AS010908 | contig_308 | NA |
| AS010909 | contig_308 | NA |
| AS010910 | contig_308 | uncharacterized protein LOC110370346 |
| AS011035 | contig_319 | estrogen-related receptor |
| AS011050 | contig_319 | NA |
| AS011051 | contig_319 | hypothetical protein B5V51_9932 |
| AS011263 | contig_327 | NA |
| AS011264 | contig_327 | hypothetical protein B5V51_10841 |
| AS011265 | contig_327 | RNA binding protein fox-1 |
| AS011283 | contig_327 | uncharacterized protein LOC111507734 |
| AS011284 | contig_327 | fatty acid synthase |
| AS011390 | contig_333 | NA |
| AS011475 | contig_336 | NA |
| AS011481 | contig_336 | uncharacterized protein PB18E9.04c-like |
| AS011812 | contig_340 | succinate dehydrogenase (ubiquinone) flavoprotein subunit |
| AS011839 | contig_340 | NA |
| AS011927 | contig_343 | alpha-1,6-mannosyltransferase |
| AS011967 | contig_345 | waprin-Phi1-like |
| AS012020 | contig_345 | uncharacterized protein LOC106142263 |
| AS012196 | contig_363 | uncharacterized protein LOC111364164 |
| AS012209 | contig_363 | gustatory receptor |
| AS012266 | contig_364 | prickle |
| AS012267 | contig_364 | uncharacterized protein LOC112056575 |
| AS012268 | contig_364 | ubiquitin-conjugating enzyme E2 S |
| AS012269 | contig_364 | uncharacterized protein LOC110383465 |
| AS012270 | contig_364 | hypothetical protein B5V51_10673 |
| AS012281 | contig_365 | Retrovirus-related Pol polyprotein from transposon TNT 1-94 |
| AS012282 | contig_365 | glutamate receptor-interacting protein |
| AS012331 | contig_372 | unnamed protein product |
| AS012332 | contig_372 | inner centromere protein A-like |
| AS012342 | contig_372 | proton-coupled amino acid transporter-like protein CG1139 |
| AS012540 | contig_380 | unnamed protein product |
| AS012579 | contig_414 | fengycin family lipopeptide synthetase D |
| AS012608 | contig_430 | hypothetical protein B5X24_HaOG203342 |
| AS012672 | contig_437 | NA |
| AS012673 | contig_437 | intermediate filament protein if |
| AS012674 | contig_437 | uncharacterized protein LOC113233491 |
| AS012675 | contig_437 | Transposon Tf2-9 polyprotein |
| AS012879 | contig_481 | mucin-5AC |
| AS013001 | contig_481 | formin-2-like |
| AS013005 | contig_481 | NA |
| AS013017 | contig_481 | nitric-oxide synthase |
| AS013177 | contig_489 | hypothetical protein B5X24_HaOG209551 |
| AS013195 | contig_489 | solute carrier family 35 (adenosine 3'-phospho 5'-phosphosulfate transporter) |
| AS013211 | contig_489 | NA |
| AS013370 | contig_505 | odorant binding protein |
| AS013371 | contig_505 | odorant binding protein |
| AS013372 | contig_505 | odorant binding protein |
| AS013373 | contig_505 | odorant binding protein |
| AS013586 | contig_515 | uncharacterized protein LOC115454967 |
| AS013625 | contig_523 | uncharacterized protein LOC113500956 |
| AS013626 | contig_523 | NA |
| AS013636 | contig_523 | DNA excision repair protein ERCC-3 |
| AS013637 | contig_523 | SWI/SNF-related matrix-associated actin-dependent regulator of chromatin subfamily A-like protein 1 |
| AS013638 | contig_523 | NA |
| AS013740 | contig_538 | probable serine hydrolase |
| AS014080 | contig_567 | organic cation transporter protein-like isoform X2 |
| AS014081 | contig_567 | MFS transporter |
| AS014082 | contig_567 | solute carrier family 22 member 7-like |
| AS014083 | contig_567 | organic cation transporter protein-like |
| AS014107 | contig_569 | uncharacterized protein LOC113522127 |
| AS014110 | contig_569 | uncharacterized protein LOC114331484 |
| AS014114 | contig_569 | inositol-1,4,5-trisphosphate 5-phosphatase |
| AS014129 | contig_569 | hypothetical protein B5X24_HaOG205504 |
| AS014274 | contig_601 | plasma membrane calcium-transporting ATPase 2 isoform X1 |
| AS014297 | contig_601 | hypothetical protein EVAR_34654_1 |
| AS014309 | contig_601 | dual specificity tyrosine-phosphorylation-regulated kinase 2/3/4 |
| AS014652 | contig_629 | bestrophin 1a |
| AS014721 | contig_633 | hypothetical protein B5V51_11412 |
| AS014789 | contig_633 | hypothetical protein B5X24_HaOG209959 |
| AS014875 | contig_635 | cyclin-dependent kinase 14 |
| AS014955 | contig_652 | uncharacterized protein LOC103579088 |
| AS014956 | contig_652 | glucose dehydrogenase [FAD, quinone] |
| AS014990 | contig_654 | uncharacterized protein LOC115447671 |
| AS014991 | contig_654 | uncharacterized protein LOC116777793 |
| AS015011 | contig_654 | uncharacterized protein LOC110374329 |
| AS015012 | contig_654 | F-type H+-transporting ATPase subunit beta |
| AS015013 | contig_654 | ubiquinol-cytochrome-c reductase complex assembly factor 1 |
| AS015014 | contig_654 | 60S ribosomal export protein NMD3 |
| AS015088 | contig_657 | hypothetical protein B5V51_12976 |
| AS015100 | contig_657 | NA |
| AS015101 | contig_657 | hypothetical protein B5X24_HaOG212154 |
| AS015105 | contig_657 | NA |
| AS015128 | contig_662 | uncharacterized protein LOC116853148 isoform X1 |
| AS015143 | contig_675 | NA |
| AS015200 | contig_683 | uncharacterized protein LOC110381480 |
| AS015217 | contig_685 | hypothetical protein B5V51_6803 |
| AS015257 | contig_688 | synaptogenesis protein syg-2-like |
| AS015270 | contig_704 | G-protein coupled receptor Mth-like isoform X2 |
| AS015299 | contig_711 | NA |
| AS015305 | contig_711 | cadherin-89D isoform X1 |
| AS015460 | contig_765 | integrin beta 1 |
| AS015540 | contig_773 | optic atrophy protein 1 |
| AS015599 | contig_783 | alkyldihydroxyacetonephosphate synthase |
| AS015648 | contig_788 | hypothetical protein B5V51_13663 |
| AS015679 | contig_801 | solute carrier family 26 (sodium-independent sulfate anion transporter) |
| AS015710 | contig_809 | a disintegrin and metalloproteinase with thrombospondin motifs 3 |
| AS015751 | contig_814 | unnamed protein product |
| AS015761 | contig_814 | transmembrane protein 135-like |
| AS016023 | contig_910 | LIM domain only protein 3-like isoform X2 |
| AS016085 | contig_919 | trypsin-7-like |
| AS016538 | contig_1078 | desaturase |
| AS016539 | contig_1078 | NA |
| AS016553 | contig_1090 | SWI/SNF-related matrix-associated actin-dependent regulator of chromatin subfamily A-like protein 1 |
| AS016625 | contig_1102 | probable splicing factor, arginine/serine-rich 7 isoform X1 |
| AS016626 | contig_1102 | cilia- and flagella-associated protein 58-like |
| AS016627 | contig_1102 | dynein heavy chain |
| AS016675 | contig_1107 | receptor-type tyrosine-protein phosphatase kappa |
| AS016706 | contig_1108 | polyprotein |
| AS016707 | contig_1108 | xanthine dehydrogenase/oxidase |
| AS016784 | contig_1154 | polypyrimidine tract-binding protein 2 |
| AS016811 | contig_1186 | unnamed protein product |
| AS017170 | contig_1420 | high affinity copper uptake protein 1-like |
| AS017381 | contig_1535 | NA |
| AS017591 | contig_1631 | NA |
| AS017592 | contig_1631 | elongation of very long chain fatty acids protein 7 |
| AS017699 | contig_1666 | uncharacterized protein LOC114355925 |
| AS017822 | contig_1873 | uncharacterized protein LOC111354912 |
| AS017886 | contig_1886 | uncharacterized protein K02A2.6-like isoform X1 |
| AS017993 | contig_1892 | NA |
| AS018099 | contig_1896 | serine/threonine-protein kinase MRCK |
| AS018100 | contig_1896 | potassium voltage-gated channel Shaw-related subfamily C member 1 |
| AS018106 | contig_1896 | WD and tetratricopeptide repeats protein 1 |
| AS018107 | contig_1896 | potassium voltage-gated channel Shaw-related subfamily C member 1 |
| AS018124 | contig_1899 | NA |
| AS018125 | contig_1899 | NA |
| AS018126 | contig_1899 | multiple inositol-polyphosphate phosphatase |
| AS018234 | contig_1908 | transmembrane and TPR repeat-containing protein CG4050-like |
| AS018253 | contig_1917 | uncharacterized protein LOC114243001 isoform X2 |
| AS018255 | contig_1917 | diamine N-acetyltransferase |
| AS018442 | contig_1972 | ecdysone-responsive G-protein coupled protein-2 |
| AS018443 | contig_1972 | hypothetical protein B5V51_3838 |
| AS018446 | contig_1978 | uncharacterized protein LOC111355046 |
| AS018510 | contig_2039 | unnamed protein product |

Notes: NA (not applicable).

**Table S21. Genes of NTC selected region identified by *F*_ST_ and π between STC and NTC**

| **Gene ID** | **Contig** | **Annotation** |
| --- | --- | --- |
| AS000036 | contig_1 | NA |
| AS000250 | contig_6 | transient-receptor-potential-like protein |
| AS000256 | contig_6 | uncharacterized protein LOC105395524 |
| AS000261 | contig_9 | uncharacterized protein LOC111356792 |
| AS000413 | contig_9 | estrone sulfotransferase |
| AS000414 | contig_9 | sulfotransferase 1C4-like |
| AS000450 | contig_9 | integrin alpha 4 |
| AS000493 | contig_12 | uncharacterized protein LOC114358071 |
| AS000747 | contig_29 | PTB domain-containing adapter protein ced-6 isoform X2 |
| AS000765 | contig_29 | protein toll |
| AS000853 | contig_31 | dopamine receptor 1 |
| AS000867 | contig_31 | cytochrome P450 |
| AS000934 | contig_31 | NA |
| AS000949 | contig_31 | attractin-like protein 1 |
| AS000958 | contig_34 | hypothetical protein B5V51_9465 |
| AS000977 | contig_34 | hypothetical protein B5V51_6482 |
| AS000978 | contig_34 | uncharacterized protein LOC113504589 |
| AS001113 | contig_34 | hypothetical protein AB894_15215 |
| AS001124 | contig_34 | Uncharacterized protein OBRU01_07853 |
| AS001125 | contig_34 | NA |
| AS001142 | contig_34 | uncharacterized protein LOC111364652 |
| AS001189 | contig_34 | optic atrophy protein 1 |
| AS001220 | contig_34 | hypothetical protein B5X24_HaOG212154 |
| AS001221 | contig_34 | hypothetical protein B5V51_10841 |
| AS001222 | contig_34 | putative bel12 ag transposon polyprotein |
| AS001223 | contig_34 | solute carrier family 35 (adenosine 3'-phospho 5'-phosphosulfate transporter) |
| AS001226 | contig_34 | uncharacterized protein LOC106711549 |
| AS001257 | contig_34 | hypothetical protein evm_013751 |
| AS001258 | contig_34 | NA |
| AS001394 | contig_37 | ATP-dependent DNA helicase PIF1 |
| AS001574 | contig_38 | WASH complex subunit 2 |
| AS001584 | contig_38 | NA |
| AS001585 | contig_38 | hypothetical protein B5V51_711 |
| AS001670 | contig_40 | histone-lysine N-methyltransferase EHMT1 |
| AS001679 | contig_40 | uncharacterized protein LOC110374652 |
| AS001752 | contig_41 | NA |
| AS001753 | contig_41 | NA |
| AS001884 | contig_45 | hypothetical protein AB894_15215 |
| AS001885 | contig_45 | peroxidase-like protein 3 |
| AS001886 | contig_45 | gustatory receptor |
| AS002039 | contig_58 | hypothetical protein evm_006094 |
| AS002099 | contig_58 | putative nuclease HARBI1 |
| AS002144 | contig_58 | hypothetical protein B5V51_407 |
| AS002145 | contig_58 | uncharacterized protein LOC114358071 |
| AS002258 | contig_62 | uncharacterized protein LOC111352853 |
| AS002296 | contig_64 | trypsin-1-like |
| AS002297 | contig_64 | NA |
| AS002498 | contig_66 | PREDICTED: uncharacterized protein LOC106710892 |
| AS002625 | contig_71 | peroxidase |
| AS002626 | contig_71 | RNA-directed DNA polymerase |
| AS002627 | contig_71 | hypothetical protein B5V51_12295 |
| AS002636 | contig_71 | dishevelled associated activator of morphogenesis |
| AS002653 | contig_71 | soluble trehalase |
| AS002825 | contig_80 | ATP synthase subunit beta, mitochondrial-like |
| AS002854 | contig_80 | uncharacterized protein LOC113240557 |
| AS002944 | contig_82 | calcium/calmodulin-dependent protein kinase |
| AS003030 | contig_82 | NA |
| AS003031 | contig_82 | NA |
| AS003122 | contig_82 | cytochrome P450 |
| AS003123 | contig_82 | cytochrome P450 |
| AS003135 | contig_82 | NA |
| AS003136 | contig_82 | uncharacterized protein LOC111352853 |
| AS003181 | contig_82 | proclotting enzyme-like isoform X8 |
| AS003381 | contig_86 | unnamed protein product |
| AS003415 | contig_92 | choline dehydrogenase |
| AS003416 | contig_92 | phosphoinositide-3-kinase |
| AS003535 | contig_96 | NA |
| AS003624 | contig_96 | calcium-activated potassium channel slowpoke isoform X4 |
| AS003632 | contig_98 | endonuclease-reverse transcriptase |
| AS003633 | contig_98 | phosphoinositide-3-kinase |
| AS003667 | contig_98 | hypothetical protein B5V51_5129 |
| AS003808 | contig_107 | optic atrophy protein 1 |
| AS003899 | contig_109 | trissin receptor-like |
| AS004122 | contig_120 | NA |
| AS004261 | contig_129 | uncharacterized protein LOC113505412 |
| AS004335 | contig_129 | uncharacterized protein LOC111357516 |
| AS004336 | contig_129 | hypothetical protein B5V51_2404 |
| AS004337 | contig_129 | uncharacterized protein LOC110377497 |
| AS004357 | contig_130 | unnamed protein product |
| AS004384 | contig_132 | RNA-directed DNA polymerase from mobile element jockey isoform X1 |
| AS004385 | contig_132 | uncharacterized protein LOC111354622 |
| AS004392 | contig_132 | peptide alpha-N-acetyltransferase |
| AS004466 | contig_134 | uncharacterized protein LOC110374163 |
| AS004516 | contig_135 | nose resistant to fluoxetine protein 6-like |
| AS004517 | contig_135 | nose resistant to fluoxetine protein 6-like |
| AS004570 | contig_138 | uncharacterized protein LOC111349283 |
| AS004632 | contig_138 | peroxidase |
| AS004633 | contig_138 | NA |
| AS004634 | contig_138 | mediator of RNA polymerase II transcription subunit 4 |
| AS004635 | contig_138 | putative inner dynein arm light chain |
| AS004636 | contig_138 | protein scribble |
| AS004761 | contig_139 | uncharacterized protein LOC108758204 |
| AS004762 | contig_139 | mitogen-activated protein kinase 5 |
| AS004813 | contig_140 | protein obstructor-E |
| AS004814 | contig_140 | protein obstructor-E |
| AS004815 | contig_140 | protein obstructor-E |
| AS005035 | contig_142 | hypothetical protein B5V51_14228 |
| AS005143 | contig_149 | trehalose 6-phosphate synthase/phosphatase |
| AS005144 | contig_149 | protein transport protein YIP1 |
| AS005199 | contig_151 | G protein-coupled receptor Mth (Methuselah protein) |
| AS005200 | contig_151 | solute carrier family 35 (adenosine 3'-phospho 5'-phosphosulfate transporter) |
| AS005212 | contig_151 | hypothetical protein B5V51_14045 |
| AS005214 | contig_151 | uncharacterized protein LOC114351244 |
| AS005215 | contig_151 | NA |
| AS005271 | contig_152 | SWI/SNF-related matrix-associated actin-dependent regulator of chromatin subfamily A-like protein 1 |
| AS005280 | contig_152 | titin-like |
| AS005285 | contig_152 | actin-binding LIM protein |
| AS005306 | contig_152 | uncharacterized protein LOC110371177 |
| AS005321 | contig_152 | uncharacterized protein LOC114356849 |
| AS005412 | contig_153 | oxysterol-binding protein-related protein 9 |
| AS005554 | contig_153 | UDP-glucose 4-epimerase |
| AS005641 | contig_154 | serine/threonine-protein kinase PRP4 homolog |
| AS005642 | contig_154 | alcohol-forming fatty acyl-CoA reductase |
| AS005743 | contig_156 | RNA-directed DNA polymerase |
| AS005781 | contig_156 | gustatory receptor |
| AS005782 | contig_156 | hypothetical protein B5V51_10841 |
| AS005783 | contig_156 | endonuclease-reverse transcriptase |
| AS005784 | contig_156 | RNA-directed DNA polymerase |
| AS005785 | contig_156 | RNA-directed DNA polymerase |
| AS005786 | contig_156 | RNA-directed DNA polymerase |
| AS005828 | contig_159 | GTPase-activating protein CdGAPr isoform X2 |
| AS005845 | contig_159 | lipase 3-like |
| AS005913 | contig_161 | elongation of very long chain fatty acids protein 7 |
| AS005914 | contig_161 | elongation of very long chain fatty acids protein 7 |
| AS005915 | contig_161 | elongation of very long chain fatty acids protein 7 |
| AS005916 | contig_161 | glutathione S-transferase |
| AS006024 | contig_162 | putative uncharacterized protein DDB_G0282133 |
| AS006088 | contig_163 | lysine-specific demethylase PHF2 |
| AS006095 | contig_163 | tyrosine-protein phosphatase non-receptor type 12/18/22 |
| AS006220 | contig_169 | bicaudal D-related protein homolog |
| AS006241 | contig_169 | phosphatidylinositol glycan |
| AS006242 | contig_169 | MFS transporter |
| AS006252 | contig_169 | uncharacterized protein CG3556 |
| AS006278 | contig_169 | uncharacterized protein LOC114251051 |
| AS006281 | contig_169 | uncharacterized protein LOC111354683 |
| AS006282 | contig_169 | uncharacterized protein LOC111360256 |
| AS006305 | contig_169 | fatty acid synthase |
| AS006306 | contig_169 | fatty acid synthase |
| AS006441 | contig_171 | hypothetical protein B5X24_HaOG212193 |
| AS006442 | contig_171 | uncharacterized protein LOC106142263 |
| AS006465 | contig_171 | unnamed protein product |
| AS006485 | contig_171 | hypothetical protein B5V51_5679 |
| AS006516 | contig_171 | solute carrier family 7 (cationic amino acid transporter) |
| AS006542 | contig_171 | failed axon connections |
| AS006561 | contig_171 | uncharacterized protein LOC112046824 |
| AS006562 | contig_171 | hypothetical protein B5X24_HaOG207319 |
| AS006615 | contig_171 | MFS transporter |
| AS006618 | contig_171 | uncharacterized protein LOC111360656 |
| AS006658 | contig_171 | period circadian protein |
| AS006810 | contig_171 | titin homolog |
| AS006811 | contig_171 | NA |
| AS006812 | contig_171 | NA |
| AS006971 | contig_174 | uncharacterized protein LOC108560498 |
| AS006982 | contig_174 | glutamate receptor 3 |
| AS006997 | contig_174 | MFS transporter |
| AS007119 | contig_175 | unnamed protein product |
| AS007303 | contig_185 | large neutral amino acids transporter small subunit 1 |
| AS007396 | contig_193 | hypothetical protein evm_003375 |
| AS007523 | contig_196 | endocuticle structural glycoprotein ABD-4-like |
| AS007564 | contig_197 | Ras-related protein Rab-3C |
| AS007676 | contig_201 | uncharacterized protein LOC110379761 isoform X1 |
| AS007677 | contig_201 | KRAB domain-containing zinc finger protein |
| AS007683 | contig_201 | uncharacterized protein LOC105382004 |
| AS007724 | contig_203 | uncharacterized protein LOC110379761 isoform X1 |
| AS007787 | contig_203 | phosphoinositide-3-kinase |
| AS007788 | contig_203 | stromal membrane-associated protein |
| AS007827 | contig_204 | endothelin-converting enzyme 2-like isoform X2 |
| AS007828 | contig_204 | uncharacterized protein LOC105383368 |
| AS007858 | contig_204 | uncharacterized protein LOC111356580 |
| AS008108 | contig_208 | hypothetical protein B5X24_HaOG216945 |
| AS008133 | contig_211 | ryanodine receptor 2 |
| AS008194 | contig_216 | Retrovirus-related Pol polyprotein from transposon TNT 1-94 |
| AS008437 | contig_219 | NA |
| AS008438 | contig_219 | optic atrophy protein 1 |
| AS008498 | contig_219 | reverse transcriptase |
| AS008499 | contig_219 | SWI/SNF-related matrix-associated actin-dependent regulator of chromatin subfamily A-like protein 1 |
| AS008531 | contig_219 | nuclear factor I |
| AS008677 | contig_226 | NA |
| AS008678 | contig_226 | SWI/SNF-related matrix-associated actin-dependent regulator of chromatin subfamily A-like protein 1 |
| AS008692 | contig_227 | cytochrome P450 |
| AS008693 | contig_227 | DNA-directed RNA polymerase II subunit RPB1 |
| AS008963 | contig_236 | hypothetical protein B5V51_3501 |
| AS009031 | contig_237 | endonuclease-reverse transcriptase |
| AS009167 | contig_239 | MFS transporter |
| AS009343 | contig_240 | NA |
| AS009564 | contig_250 | eukaryotic translation elongation factor 1 epsilon-1 |
| AS009565 | contig_250 | NA |
| AS009663 | contig_250 | farnesyl diphosphate synthase |
| AS009664 | contig_250 | farnesyl diphosphate synthase |
| AS009665 | contig_250 | farnesyl diphosphate synthase |
| AS009666 | contig_250 | farnesyl diphosphate synthase |
| AS009716 | contig_251 | oxysterol-binding protein-related protein 5/8 |
| AS009737 | contig_251 | NA |
| AS009738 | contig_251 | protein artemis-like |
| AS009848 | contig_251 | solute carrier family 35 (adenosine 3'-phospho 5'-phosphosulfate transporter) |
| AS009849 | contig_251 | NA |
| AS009896 | contig_254 | cytochrome P450 |
| AS009897 | contig_254 | cytochrome P450 |
| AS009922 | contig_256 | uncharacterized protein LOC110371998 |
| AS009969 | contig_258 | uncharacterized protein LOC111350041 |
| AS010074 | contig_261 | glutathione S-transferase |
| AS010075 | contig_261 | glutathione S-transferase |
| AS010282 | contig_269 | NA |
| AS010364 | contig_270 | ectonucleotide pyrophosphatase/phosphodiesterase family member 7 |
| AS010365 | contig_270 | NA |
| AS010403 | contig_274 | pancreatic triacylglycerol lipase-like |
| AS010621 | contig_288 | receptor-type guanylate cyclase gcy-1 |
| AS010622 | contig_288 | uncharacterized protein LOC110372423 |
| AS010637 | contig_288 | nitrogen permease regulator 2-like protein |
| AS010651 | contig_288 | hypothetical protein B5X24_HaOG215196 |
| AS010664 | contig_288 | hypothetical protein B5V51_2131 |
| AS010665 | contig_288 | NA |
| AS010666 | contig_288 | annexin B10 isoform X2 |
| AS010715 | contig_293 | NA |
| AS010716 | contig_293 | uncharacterized protein LOC116770084 |
| AS010769 | contig_300 | endoribonuclease Dicer |
| AS010825 | contig_308 | furin |
| AS010851 | contig_308 | odorant receptor |
| AS011241 | contig_325 | endonuclease-reverse transcriptase |
| AS011390 | contig_333 | NA |
| AS011736 | contig_338 | Ras-related protein Rab-37 |
| AS011812 | contig_340 | succinate dehydrogenase (ubiquinone) flavoprotein subunit |
| AS011915 | contig_343 | scavenger receptor class B |
| AS011970 | contig_345 | intermediate filament protein if |
| AS011989 | contig_345 | NA |
| AS012111 | contig_352 | phosphatidylinositol phospholipase C |
| AS012170 | contig_359 | NA |
| AS012175 | contig_359 | solute carrier family 41 |
| AS012279 | contig_365 | glutamate receptor-interacting protein 2 isoform X6 |
| AS012430 | contig_377 | uncharacterized protein LOC116777582 |
| AS012449 | contig_378 | uncharacterized protein LOC116853148 |
| AS012475 | contig_378 | NA |
| AS012610 | contig_430 | hypothetical protein B5V51_12295 |
| AS012651 | contig_433 | ADP-ribosylation factor-like protein 6 |
| AS012684 | contig_437 | MFS transporter |
| AS012685 | contig_437 | uncharacterized protein LOC113496015 |
| AS012757 | contig_447 | NA |
| AS012781 | contig_448 | hypothetical protein B5V51_2174 |
| AS012803 | contig_448 | uncharacterized protein LOC106720343 |
| AS013002 | contig_481 | hypothetical protein B5V51_14410 |
| AS013039 | contig_484 | NA |
| AS013041 | contig_484 | uncharacterized protein LOC113507375 |
| AS013042 | contig_484 | NA |
| AS013056 | contig_484 | transmembrane protease serine 9 |
| AS013070 | contig_486 | Retrovirus-related Pol polyprotein from transposon 17.6 |
| AS013386 | contig_505 | NA |
| AS013387 | contig_505 | NA |
| AS013391 | contig_505 | NA |
| AS013480 | contig_510 | hypothetical protein B5X24_HaOG209701 |
| AS013481 | contig_510 | uncharacterized protein LOC111357108 |
| AS013482 | contig_510 | uncharacterized protein LOC111355311 |
| AS013670 | contig_530 | COMM domain-containing protein 4 isoform X1 |
| AS013766 | contig_547 | hypothetical protein B5V51_3395 |
| AS013776 | contig_549 | fatty acid synthase |
| AS013860 | contig_552 | serine/threonine-protein kinase MRCK |
| AS013891 | contig_557 | lachesin-like isoform X2 |
| AS014110 | contig_569 | uncharacterized protein LOC114331484 |
| AS014129 | contig_569 | hypothetical protein B5X24_HaOG205504 |
| AS014143 | contig_570 | hypothetical protein B5V51_14228 |
| AS014241 | contig_577 | pyruvate |
| AS014242 | contig_577 | pyruvate |
| AS014274 | contig_601 | plasma membrane calcium-transporting ATPase 2 |
| AS014296 | contig_601 | hypothetical protein B5V51_10801 |
| AS014532 | contig_608 | hypothetical protein B5V51_8997 |
| AS014576 | contig_620 | mannosyl-oligosaccharide alpha-1,2-mannosidase |
| AS014784 | contig_633 | Uncharacterized protein OBRU01_10652 |
| AS014873 | contig_635 | NA |
| AS014982 | contig_652 | NA |
| AS014992 | contig_654 | optic atrophy protein 1 |
| AS014994 | contig_654 | ribosome production factor 2 |
| AS014995 | contig_654 | endonuclease-reverse transcriptase |
| AS014996 | contig_654 | optic atrophy protein 1 |
| AS015104 | contig_657 | phosphatidylinositol glycan |
| AS015105 | contig_657 | NA |
| AS015193 | contig_683 | putative integrase core domain protein |
| AS015194 | contig_683 | growth hormone secretagogue receptor |
| AS015195 | contig_683 | hypothetical protein B5X24_HaOG213278 |
| AS015243 | contig_688 | serine/threonine-protein kinase MRCK |
| AS015253 | contig_688 | gustatory receptor |
| AS015279 | contig_704 | sodium/hydrogen exchanger 9B1-like isoform X2 |
| AS015363 | contig_757 | Uncharacterized protein OBRU01_02174 |
| AS015584 | contig_783 | NA |
| AS015617 | contig_787 | putative inorganic phosphate cotransporter |
| AS015619 | contig_787 | putative inorganic phosphate cotransporter |
| AS015623 | contig_788 | NA |
| AS015682 | contig_804 | prominin 1 |
| AS015749 | contig_814 | serine/threonine-protein kinase MRCK |
| AS015828 | contig_843 | cytochrome P450 |
| AS015838 | contig_843 | centromeric protein E |
| AS015839 | contig_843 | protein MAK16 |
| AS015992 | contig_901 | inactive rhomboid protein 1 isoform X1 |
| AS016003 | contig_901 | tissue factor pathway inhibitor |
| AS016044 | contig_919 | MFS transporter |
| AS016045 | contig_919 | cytochrome P450 |
| AS016046 | contig_919 | cytochrome P450 |
| AS016141 | contig_933 | forkhead box protein O3 |
| AS016165 | contig_933 | NA |
| AS016166 | contig_933 | NA |
| AS016167 | contig_933 | NA |
| AS016168 | contig_933 | endonuclease-reverse transcriptase |
| AS016560 | contig_1093 | uncharacterized protein LOC111513886 |
| AS016597 | contig_1102 | potassium/sodium hyperpolarization-activated cyclic nucleotide-gated channel 2 isoform X4 |
| AS016676 | contig_1107 | NA |
| AS016710 | contig_1108 | pyruvate carboxylase |
| AS016732 | contig_1110 | NA |
| AS016744 | contig_1110 | tripartite motif-containing protein 45 |
| AS016854 | contig_1203 | disks large protein 1 |
| AS017352 | contig_1526 | globin-2 A chain-like |
| AS017354 | contig_1526 | hypothetical protein B5X24_HaOG211462 |
| AS017451 | contig_1573 | solute carrier family 6 (neurotransmitter transporter) |
| AS017590 | contig_1631 | elongation of very long chain fatty acids protein 7 |
| AS017676 | contig_1665 | aldo-keto reductase |
| AS017677 | contig_1665 | aldo-keto reductase AKR2E4-like isoform X1 |
| AS017856 | contig_1879 | glucose-6-phosphate isomerase |
| AS017861 | contig_1880 | uncharacterized protein LOC113500251 |
| AS017888 | contig_1886 | uncharacterized protein LOC111351166 |
| AS017928 | contig_1887 | prolactin regulatory element-binding protein |
| AS017929 | contig_1887 | hypothetical protein B5V51_9823 |
| AS018057 | contig_1893 | protein MON2 homolog |
| AS018256 | contig_1926 | hypothetical protein B5X24_HaOG212902 |
| AS018291 | contig_1933 | uncharacterized protein LOC111348907 |
| AS018347 | contig_1938 | uncharacterized protein LOC113508244 |
| AS018348 | contig_1938 | unnamed protein product |
| AS018349 | contig_1938 | endonuclease-reverse transcriptase |
| AS018389 | contig_1951 | NA |
| AS018408 | contig_1957 | ubiquitin carboxyl-terminal hydrolase 31 |
| AS018409 | contig_1957 | ATP-binding cassette |

Notes: NA (not applicable).

**Table S22. Genes of STC selected region identified by *F*_ST_ and π between STC and NTC**

| **Gene ID** | **Contig** | **Annotation** |
| --- | --- | --- |
| AS000187 | contig_4 | sorting nexin-13 |
| AS000188 | contig_4 | uncharacterized protein LOC113505673 |
| AS000189 | contig_4 | unnamed protein product |
| AS000197 | contig_4 | NA |
| AS000198 | contig_4 | phosphoinositide-3-kinase |
| AS000199 | contig_4 | piggyBac transposable element-derived protein 4-like |
| AS000200 | contig_4 | uncharacterized protein LOC111000509 |
| AS000537 | contig_25 | modifier of mdg4-like isoform X1 |
| AS000538 | contig_25 | 2-hydroxyglutarate dehydrogenase |
| AS000517 | contig_25 | hypothetical protein B5V51_14463 |
| AS000518 | contig_25 | hypothetical protein B5V51_7957 |
| AS000922 | contig_31 | NA |
| AS000923 | contig_31 | piggyBac transposable element-derived protein 3-like |
| AS001437 | contig_37 | protein toll |
| AS001848 | contig_45 | phosphatidylinositol 3-kinase |
| AS001974 | contig_57 | protein turtle isoform X2 |
| AS001975 | contig_57 | lipase member H-like |
| AS001980 | contig_57 | NA |
| AS001981 | contig_57 | hypothetical protein B5V51_5309 |
| AS002026 | contig_58 | UDP-glucuronosyltransferase |
| AS002027 | contig_58 | UDP-glucuronosyltransferase |
| AS002712 | contig_76 | oxysterol-binding protein-related protein |
| AS002715 | contig_76 | hypothetical protein EVAR_35820_1 |
| AS002717 | contig_76 | uncharacterized protein LOC106710656 |
| AS002718 | contig_76 | NA |
| AS003287 | contig_82 | uncharacterized protein LOC111360097 |
| AS003288 | contig_82 | proteasome subunit beta type-3 |
| AS002951 | contig_82 | uncharacterized protein LOC111360005 |
| AS002952 | contig_82 | uncharacterized protein LOC110373676 |
| AS003747 | contig_102 | NA |
| AS003899 | contig_109 | trissin receptor-like |
| AS003955 | contig_113 | hypothetical protein B5X24_HaOG204683 |
| AS004172 | contig_122 | transient receptor potential cation channel subfamily M member 3 |
| AS004220 | contig_128 | adenylate cyclase 2 |
| AS004624 | contig_138 | uncharacterized protein LOC116770131 |
| AS004628 | contig_138 | hypothetical protein B5X24_HaOG212154 |
| AS004827 | contig_140 | uncharacterized protein LOC116770084 |
| AS004843 | contig_140 | putative nuclease HARBI1 |
| AS005443 | contig_153 | hypothetical protein B5V51_3501 |
| AS006305 | contig_169 | fatty acid synthase |
| AS006407 | contig_171 | hypothetical protein B5X24_HaOG205330 |
| AS006648 | contig_171 | Kip1 ubiquitination-promoting complex protein 1 |
| AS006650 | contig_171 | hypothetical protein B5V51_1187 |
| AS006655 | contig_171 | uncharacterized protein LOC110374481 |
| AS006668 | contig_171 | NA |
| AS006683 | contig_171 | peptide transporter family 1 isoform X2 |
| AS006687 | contig_171 | clock |
| AS006706 | contig_171 | hypothetical protein B5X24_HaOG214709 |
| AS006790 | contig_171 | uncharacterized protein LOC111355311 |
| AS006792 | contig_171 | gamma-aminobutyric acid receptor subunit beta-like |
| AS006793 | contig_171 | JNK-interacting protein 1 |
| AS006804 | contig_171 | tyrosine-protein phosphatase non-receptor type 1 |
| AS006808 | contig_171 | mitogen-activated protein kinase 13 |
| AS006809 | contig_171 | hypothetical protein B5X24_HaOG204423 |
| AS006837 | contig_171 | transmembrane channel-like protein 2 isoform X1 |
| AS006838 | contig_171 | NA |
| AS006839 | contig_171 | laminin |
| AS006396 | contig_171 | coiled-coil domain-containing protein AGAP005037 |
| AS006490 | contig_171 | sensory neuron membrane protein 2 |
| AS006397 | contig_171 | NA |
| AS006491 | contig_171 | hypothetical protein B5V51_7144 |
| AS006399 | contig_171 | NA |
| AS006400 | contig_171 | piggyBac transposable element-derived protein 3-like |
| AS006401 | contig_171 | SRC kinase signaling inhibitor 1 |
| AS006402 | contig_171 | solute carrier family 35 (UDP-galactose transporter) |
| AS006553 | contig_171 | hypothetical protein B5V51_13177 |
| AS006561 | contig_171 | uncharacterized protein LOC112046824 |
| AS006562 | contig_171 | hypothetical protein B5X24_HaOG207319 |
| AS006403 | contig_171 | basement membrane-specific heparan sulfate proteoglycan core protein |
| AS006564 | contig_171 | NA |
| AS006566 | contig_171 | SCY1-like protein 2 |
| AS006568 | contig_171 | hypothetical protein B5V51_14513 |
| AS006569 | contig_171 | large proline-rich protein BAG6 isoform X5 |
| AS006623 | contig_171 | neuronal PAS domain-containing protein 2-like isoform X2 |
| AS006624 | contig_171 | unnamed protein product |
| AS007096 | contig_174 | Arf-GAP with Rho-GAP domain |
| AS007723 | contig_203 | NA |
| AS008129 | contig_208 | NA |
| AS008130 | contig_208 | gustatory receptor |
| AS008144 | contig_211 | protein cueball-like |
| AS008146 | contig_214 | NA |
| AS008147 | contig_214 | RYamide receptor-like |
| AS008607 | contig_223 | EF-hand domain-containing protein 1-like |
| AS008706 | contig_227 | hypothetical protein B5V51_8949 |
| AS008699 | contig_227 | venom dipeptidyl peptidase 4-like isoform X1 |
| AS008902 | contig_236 | unnamed protein product |
| AS009033 | contig_237 | pickpocket protein 28-like |
| AS009034 | contig_237 | pickpocket protein 28-like |
| AS009093 | contig_238 | uncharacterized protein LOC113503959 |
| AS009094 | contig_238 | hypothetical protein B5X24_HaOG212193 |
| AS009095 | contig_238 | hypothetical protein B5V51_13147 |
| AS009076 | contig_238 | histone acetyltransferase MYST4 |
| AS009077 | contig_238 | F-box and leucine-rich repeat protein 15 |
| AS009078 | contig_238 | cytochrome P450 |
| AS009083 | contig_238 | gustatory receptor |
| AS009084 | contig_238 | putative odorant receptor |
| AS009086 | contig_238 | lg-like and fibronectin type-III domain-containing protein 2 isoform X3 |
| AS009176 | contig_239 | hypothetical protein B5X24_HaOG204559 |
| AS009459 | contig_244 | myosin heavy chain 6/7 |
| AS009511 | contig_247 | NA |
| AS009549 | contig_250 | chemosensory protein 12 |
| AS009667 | contig_250 | farnesyl diphosphate synthase |
| AS009567 | contig_250 | NA |
| AS009568 | contig_250 | NA |
| AS009569 | contig_250 | glycine-rich protein 2-like |
| AS009863 | contig_252 | NADH dehydrogenase 1 alpha subcomplex assembly factor 6 |
| AS009917 | contig_256 | NA |
| AS009918 | contig_256 | NA |
| AS010255 | contig_268 | integrin alpha-X-like |
| AS010834 | contig_308 | uncharacterized protein LOC110370404 |
| AS011378 | contig_330 | hypothetical protein B5X24_HaOG216703 |
| AS011379 | contig_330 | RYamide receptor-like |
| AS011489 | contig_336 | nephrin-like isoform X2 |
| AS011479 | contig_336 | calcium-sensing receptor |
| AS011481 | contig_336 | uncharacterized protein PB18E9.04c-like |
| AS011777 | contig_339 | NA |
| AS011779 | contig_339 | putative endonuclease/reverse transcriptase |
| AS011770 | contig_339 | serpin B8-like |
| AS011771 | contig_339 | male-enhanced antigen 1 |
| AS011786 | contig_339 | histidine-rich glycoprotein-like |
| AS011788 | contig_339 | NA |
| AS011772 | contig_339 | protein-L-isoaspartate(D-aspartate) O-methyltransferase |
| AS011792 | contig_339 | E3 ubiquitin-protein ligase HECTD1 |
| AS011794 | contig_339 | tetratricopeptide repeat protein 8 |
| AS011795 | contig_339 | vacuolar protein sorting-associated protein 54 |
| AS011796 | contig_339 | exocyst complex component 3 |
| AS011773 | contig_339 | hypothetical protein B5V51_9932 |
| AS011774 | contig_339 | DNA repair endonuclease XPF |
| AS012002 | contig_345 | carbonic anhydrase |
| AS012020 | contig_345 | NA |
| AS012341 | contig_372 | hepatic leukemia factor |
| AS012342 | contig_372 | proton-coupled amino acid transporter-like protein CG1139 |
| AS012570 | contig_414 | uncharacterized protein LOC111357026 isoform X2 |
| AS012643 | contig_432 | uncharacterized protein LOC111362300 |
| AS012760 | contig_447 | uncharacterized protein LOC110382640 |
| AS013503 | contig_512 | gamma-1-syntrophin |
| AS013506 | contig_512 | NA |
| AS014407 | contig_604 | protein lifeguard 1 isoform X4 |
| AS014886 | contig_635 | hypothetical protein B5V51_12025 |
| AS014989 | contig_654 | neurotrimin-like isoform X1 |
| AS015101 | contig_657 | hypothetical protein B5X24_HaOG212154 |
| AS015183 | contig_681 | NA |
| AS015740 | contig_811 | TATA-box-binding protein |
| AS015741 | contig_811 | eukaryotic translation initiation factor 3 subunit K |
| AS015742 | contig_811 | histidine-rich glycoprotein-like |
| AS015743 | contig_811 | NA |
| AS015739 | contig_811 | WASH complex subunit 7 |
| AS016126 | contig_932 | hypothetical protein B5V51_14479 |
| AS016127 | contig_932 | PITH domain-containing protein GA19395 |
| AS016128 | contig_932 | collagen |
| AS016258 | contig_969 | uncharacterized protein LOC106718269 |
| AS016500 | contig_1071 | zinc finger protein chinmo isoform X2 |
| AS016537 | contig_1078 | NA |
| AS016538 | contig_1078 | desaturase |
| AS016540 | contig_1078 | uncharacterized protein LOC111353699 |
| AS016541 | contig_1078 | pyrokinin-1 receptor-like |
| AS016862 | contig_1203 | hypothetical protein B5V51_12295 |
| AS017136 | contig_1374 | unnamed protein product |
| AS017246 | contig_1463 | large subunit ribosomal protein L38 |
| AS017254 | contig_1463 | hypothetical protein DMN91_010341 |
| AS017255 | contig_1463 | hypothetical protein B5V51_10841 |
| AS017256 | contig_1463 | NA |
| AS017249 | contig_1463 | hypothetical protein AB894_15215 |
| AS017250 | contig_1463 | uncharacterized protein LOC116770131 |
| AS017389 | contig_1548 | hypothetical protein B5V51_239 |
| AS017591 | contig_1631 | NA |
| AS017592 | contig_1631 | elongation of very long chain fatty acids protein 7 |
| AS017651 | contig_1664 | ketohexokinase |
| AS017617 | contig_1664 | unnamed protein product |
| AS017656 | contig_1664 | ABCC1 protein |
| AS017618 | contig_1664 | NADH dehydrogenase (ubiquinone) 1 subunit C2 |
| AS017619 | contig_1664 | NA |
| AS017625 | contig_1664 | nuclear pore complex protein Nup53 |
| AS017631 | contig_1664 | NA |
| AS017632 | contig_1664 | receptor tyrosine kinase-like orphan receptor 1 |
| AS017633 | contig_1664 | NA |
| AS017641 | contig_1664 | alpha-aminoadipic semialdehyde synthase |
| AS017642 | contig_1664 | unnamed protein product |
| AS017700 | contig_1666 | NA |
| AS017701 | contig_1666 | NA |
| AS017699 | contig_1666 | uncharacterized protein LOC114355925 |
| AS018065 | contig_1895 | hypothetical protein B5V51_1304 |
| AS018290 | contig_1932 | Cholecystokinin receptor |

Notes: NA (not applicable).

**Table S23. Genes of NEC selected region identified by *F*_ST_ and π between NTC and NEC**

| **Gene ID** | **Contig** | **Annotation** |
| --- | --- | --- |
| AS000382 | contig_9 | troponin C-like isoform X1 |
| AS000383 | contig_9 | uncharacterized protein LOC115445367 |
| AS000384 | contig_9 | Macrophage mannose receptor 1 |
| AS000385 | contig_9 | uncharacterized protein LOC110372611 |
| AS000386 | contig_9 | hypothetical protein B5V51_756 |
| AS000828 | contig_31 | methenyltetrahydrofolate synthase domain-containing protein isoform X1 |
| AS001165 | contig_34 | NA |
| AS001833 | contig_45 | uncharacterized protein LOC110381944 |
| AS001834 | contig_45 | intraflagellar transport protein 46 homolog |
| AS002401 | contig_65 | protein FAM13A isoform X1 |
| AS002514 | contig_66 | Uncharacterized protein OBRU01_06965 |
| AS002432 | contig_66 | zwei Ig domain protein zig-8-like |
| AS003276 | contig_82 | peptidoglycan recognition protein D |
| AS003114 | contig_82 | myosin XVIII |
| AS003371 | contig_86 | NA |
| AS003882 | contig_109 | hypothetical protein evm_005326 |
| AS003892 | contig_109 | pyruvate kinase-like |
| AS003883 | contig_109 | mediator of RNA polymerase II transcription subunit 11 |
| AS003893 | contig_109 | serine-protein kinase ATM |
| AS003884 | contig_109 | hypothetical protein B5V51_7499 |
| AS003885 | contig_109 | diacylglycerol O-acyltransferase 1 |
| AS004191 | contig_123 | solute carrier family 35 |
| AS004192 | contig_123 | THO complex subunit 7 |
| AS004193 | contig_123 | phospholipase D3-like isoform X1 |
| AS004285 | contig_129 | NA |
| AS004297 | contig_129 | hypothetical protein B5X24_HaOG201630 |
| AS004299 | contig_129 | uncharacterized protein LOC110373197 |
| AS004310 | contig_129 | unnamed protein product |
| AS004311 | contig_129 | unnamed protein product |
| AS004624 | contig_138 | uncharacterized protein LOC116770131 |
| AS004843 | contig_140 | putative nuclease HARBI1 |
| AS004931 | contig_140 | high affinity copper uptake protein 1-like |
| AS004932 | contig_140 | bromodomain-containing protein 3 |
| AS005001 | contig_140 | uncharacterized protein LOC113492658 |
| AS005002 | contig_140 | mitochondrial chaperone BCS1 |
| AS005257 | contig_151 | bifunctional coenzyme A synthase isoform X1 |
| AS005258 | contig_151 | G-protein coupled receptor Mth2-like isoform X2 |
| AS005189 | contig_151 | glutathione S-transferase |
| AS005906 | contig_160 | calcium/calmodulin-dependent 3',5'-cyclic nucleotide phosphodiesterase |
| AS005930 | contig_162 | unnamed protein product |
| AS005991 | contig_162 | uncharacterized protein LOC111351402 |
| AS005992 | contig_162 | hypothetical protein B5V51_5265 |
| AS005993 | contig_162 | NA |
| AS005994 | contig_162 | optic atrophy protein 1 |
| AS005995 | contig_162 | uncharacterized protein LOC111351402 |
| AS005941 | contig_162 | uncharacterized protein LOC111351402 |
| AS005942 | contig_162 | NA |
| AS005931 | contig_162 | double stranded RNA-specific editase B |
| AS005929 | contig_162 | ATP-binding cassette |
| AS006171 | contig_168 | NA |
| AS006491 | contig_171 | hypothetical protein B5V51_7144 |
| AS007129 | contig_175 | adenylate cyclase 9 |
| AS007130 | contig_175 | hypothetical protein evm_013249 |
| AS007131 | contig_175 | uncharacterized protein LOC111358654 |
| AS007132 | contig_175 | phosphorylase kinase alpha/beta subunit |
| AS007164 | contig_175 | hypothetical protein B5V51_13090 |
| AS007239 | contig_183 | arginine kinase |
| AS007240 | contig_183 | hypothetical protein B5V51_5404 |
| AS007241 | contig_183 | hypothetical protein B5X24_HaOG205854 |
| AS007244 | contig_183 | uncharacterized protein LOC113505275 |
| AS007246 | contig_183 | D-beta-hydroxybutyrate dehydrogenase |
| AS007253 | contig_183 | MFS transporter |
| AS007281 | contig_185 | hypothetical protein evm_012276 |
| AS007440 | contig_194 | uncharacterized protein LOC114245972 |
| AS007441 | contig_194 | sorting nexin-12 |
| AS007442 | contig_194 | pre-mRNA-processing factor SLU7 |
| AS007532 | contig_196 | WD and tetratricopeptide repeats protein 1 |
| AS007533 | contig_196 | flexible cuticle protein 12-like |
| AS007534 | contig_196 | flexible cuticle protein 12-like |
| AS007546 | contig_196 | NA |
| AS007563 | contig_197 | receptor-type tyrosine-protein phosphatase R |
| AS007687 | contig_202 | ATP-binding cassette |
| AS007915 | contig_204 | xanthine dehydrogenase/oxidase |
| AS008118 | contig_208 | fatty alcohol acetyltransferase |
| AS008119 | contig_208 | hypothetical protein B5V51_11035 |
| AS008120 | contig_208 | traB domain-containing protein-like |
| AS008491 | contig_219 | RNA-directed DNA polymerase |
| AS008519 | contig_219 | uncharacterized protein LOC113502962 |
| AS008688 | contig_226 | leucine-rich repeat-containing protein 15-like |
| AS008805 | contig_235 | neuropilin and tolloid-like protein 2 |
| AS008874 | contig_235 | Rad and Gem related GTP binding protein 1 |
| AS008791 | contig_235 | NA |
| AS009028 | contig_237 | NA |
| AS009030 | contig_237 | uncharacterized protein LOC114363178 |
| AS009162 | contig_239 | cytochrome P450 |
| AS009163 | contig_239 | cytochrome P450 |
| AS009164 | contig_239 | cytochrome P450 |
| AS009166 | contig_239 | cytochrome P450 |
| AS009173 | contig_239 | hypothetical protein B5V51_4400 |
| AS009177 | contig_239 | octopamine receptor Oamb isoform X1 |
| AS009623 | contig_250 | hypothetical protein AB894_15215 |
| AS009628 | contig_250 | hypothetical protein B5X24_HaOG207006 |
| AS009868 | contig_252 | activating molecule in BECN1-regulated autophagy protein 1 |
| AS009869 | contig_252 | activating molecule in BECN1-regulated autophagy protein 1 |
| AS009870 | contig_252 | NA |
| AS009969 | contig_258 | uncharacterized protein LOC111350041 |
| AS010067 | contig_260 | hypothetical protein B5X24_HaOG209515 |
| AS010082 | contig_261 | uncharacterized protein LOC111349511 |
| AS010142 | contig_263 | opsin, ultraviolet-sensitive-like isoform X1 |
| AS010255 | contig_268 | integrin alpha-X-like |
| AS010823 | contig_306 | uncharacterized protein LOC111348910 |
| AS010932 | contig_308 | uncharacterized protein LOC111362952 |
| AS011179 | contig_325 | juvenile hormone epoxide hydrolase |
| AS011180 | contig_325 | juvenile hormone epoxide hydrolase |
| AS011340 | contig_328 | hypothetical protein RF55_22593 |
| AS011406 | contig_333 | ornithine--oxo-acid transaminase |
| AS011407 | contig_333 | NADH dehydrogenase 1 alpha subcomplex assembly factor 2 |
| AS011408 | contig_333 | uncharacterized protein C45G9.7 |
| AS011411 | contig_333 | mitogen-activated protein kinase 14B |
| AS011412 | contig_333 | NADH dehydrogenase (ubiquinone) 1 beta subcomplex subunit 6 |
| AS011413 | contig_333 | 18S rRNA-dimethyltransferase |
| AS011414 | contig_333 | transportin-1 |
| AS011417 | contig_334 | unnamed protein product |
| AS011503 | contig_336 | uncharacterized protein LOC114245972 |
| AS011504 | contig_336 | phospholipid-translocating ATPase |
| AS011505 | contig_336 | hypothetical protein B5X24_HaOG216889 |
| AS011506 | contig_336 | acyl-CoA synthetase |
| AS011541 | contig_337 | hypothetical protein B5X24_HaOG210098 |
| AS011542 | contig_337 | actin-binding LIM protein |
| AS011661 | contig_338 | fatty alcohol acetyltransferase |
| AS011662 | contig_338 | fatty alcohol acetyltransferase |
| AS011768 | contig_339 | uncharacterized protein LOC114242079 |
| AS011769 | contig_339 | unnamed protein product |
| AS011770 | contig_339 | serpin B8-like |
| AS011767 | contig_339 | uncharacterized protein LOC111362874 |
| AS012009 | contig_345 | tachykinin-like receptor |
| AS012029 | contig_345 | uncharacterized protein LOC114354545 |
| AS012030 | contig_345 | hypothetical protein B5V51_14296 |
| AS012035 | contig_345 | arylsulfatase B |
| AS012036 | contig_345 | arylsulfatase B |
| AS012572 | contig_414 | uncharacterized protein LOC111357161 |
| AS012717 | contig_446 | disks large protein 1 |
| AS013079 | contig_486 | glucose dehydrogenase [FAD, quinone]-like |
| AS013080 | contig_486 | NA |
| AS013207 | contig_489 | DNA (cytosine-5)-methyltransferase 1 |
| AS013208 | contig_489 | ESF1 homolog |
| AS013209 | contig_489 | TP53-regulated inhibitor of apoptosis 1-like |
| AS013210 | contig_489 | transcription elongation factor SPT4 |
| AS013211 | contig_489 | NA |
| AS013341 | contig_498 | NA |
| AS013374 | contig_505 | JHBP domain-containing protein |
| AS013375 | contig_505 | hypothetical protein B5X24_HaOG207672 |
| AS013376 | contig_505 | NA |
| AS013419 | contig_507 | NA |
| AS013691 | contig_530 | hypothetical protein B5V51_5077 |
| AS013749 | contig_538 | cytochrome P450 |
| AS013896 | contig_561 | annulin isoform X2 |
| AS014003 | contig_566 | CUG-BP- and ETR3-like factor |
| AS014111 | contig_569 | hypothetical protein B5X24_HaOG215544 |
| AS014114 | contig_569 | inositol-1,4,5-trisphosphate 5-phosphatase |
| AS014164 | contig_573 | NA |
| AS014283 | contig_601 | receptor-type tyrosine-protein phosphatase gamma |
| AS014525 | contig_608 | reverse transcriptase |
| AS015253 | contig_688 | gustatory receptor |
| AS015614 | contig_787 | Retrovirus-related Pol polyprotein from transposon TNT 1-94-like Protein |
| AS015789 | contig_831 | uncharacterized protein LOC111351250 isoform X1 |
| AS016194 | contig_944 | protein rolling stone-like |
| AS016195 | contig_944 | hypothetical protein B5V51_11315 |
| AS016767 | contig_1139 | hypothetical protein B5V51_14467 |
| AS016997 | contig_1285 | protein prenyltransferase alpha subunit repeat containing protein 1 |
| AS017001 | contig_1285 | 15-hydroxyprostaglandin dehydrogenase (NAD) |
| AS017013 | contig_1286 | cytochrome P450 |
| AS017133 | contig_1368 | multidrug resistance-associated protein 1 isoform X3 |
| AS017134 | contig_1368 | uncharacterized protein LOC111362874 isoform X1 |
| AS017136 | contig_1374 | unnamed protein product |
| AS017166 | contig_1420 | serine/threonine-protein kinase D1 |
| AS017167 | contig_1420 | acetylcholinesterase |
| AS017191 | contig_1428 | hypothetical protein B5V51_3833 |
| AS017326 | contig_1517 | glycogen-binding subunit 76A isoform X2 |
| AS017325 | contig_1517 | heparan sulfate 2-O-sulfotransferase HS2ST1 |
| AS017522 | contig_1602 | TATA element modulatory factor |
| AS017783 | contig_1788 | ATP synthase subunit alpha, mitochondrial |
| AS017784 | contig_1788 | PX domain-containing protein kinase-like protein |
| AS018239 | contig_1908 | zinc/cadmium resistance protein isoform X1 |
| AS018339 | contig_1938 | NA |
| AS018340 | contig_1938 | NA |
| AS018341 | contig_1938 | NA |
| AS018342 | contig_1938 | NA |

Notes: NA (not applicable).

**Table S24. Genes of NTC selected region identified by *F*_ST_ and π between NTC and NEC**

| **Gene ID** | **Contig** | **Annotation** |
| --- | --- | --- |
| AS000222 | contig_6 | uncharacterized protein LOC106711886 |
| AS000241 | contig_6 | hypothetical protein B5V51_3436 |
| AS000411 | contig_9 | estrone sulfotransferase |
| AS000446 | contig_9 | hypothetical protein OBRU01_11432 |
| AS000278 | contig_9 | Ca (2+)/calmodulin-responsive adenylate cyclase |
| AS000489 | contig_12 | uncharacterized protein LOC113492498 |
| AS000738 | contig_29 | NA |
| AS000758 | contig_29 | hypothetical protein B5V51_1613 |
| AS000765 | contig_29 | hypothetical protein B5X24_HaOG205376 |
| AS000716 | contig_29 | androgen-dependent TFPI-regulating protein-like |
| AS000879 | contig_31 | NA |
| AS000949 | contig_31 | attractin-like protein 1 |
| AS001041 | contig_34 | hypothetical protein B5V51_4896 |
| AS001042 | contig_34 | MFS transporter |
| AS000962 | contig_34 | NA |
| AS000963 | contig_34 | hypothetical protein B5V51_6155 |
| AS000964 | contig_34 | hypothetical protein evm_012717 |
| AS001085 | contig_34 | unnamed protein product |
| AS001086 | contig_34 | uncharacterized protein LOC113505908 |
| AS001087 | contig_34 | Retrotransposable element |
| AS001113 | contig_34 | hypothetical protein AB894_15215 |
| AS001168 | contig_34 | hypothetical protein evm_007716 |
| AS001169 | contig_34 | hypothetical protein B5X24_HaOG201365 |
| AS001198 | contig_34 | ATP-dependent DNA helicase PIF1 |
| AS001199 | contig_34 | unnamed protein product |
| AS001239 | contig_34 | uncharacterized protein LOC111352853 |
| AS001261 | contig_34 | hypothetical protein evm_013477 |
| AS001009 | contig_34 | Zinc finger MYM-type protein 1 |
| AS001010 | contig_34 | uncharacterized protein LOC106100064 |
| AS001288 | contig_35 | uncharacterized protein LOC114356249 |
| AS001314 | contig_36 | thyroid transcription factor 1-like |
| AS001329 | contig_36 | NA |
| AS001392 | contig_37 | inactive dipeptidyl peptidase 10 |
| AS001432 | contig_37 | disintegrin and metalloproteinase domain-containing protein 10 |
| AS001379 | contig_37 | aldehyde dehydrogenase (NAD+) |
| AS001736 | contig_41 | hypothetical protein B5V51_12791 |
| AS001794 | contig_41 | ubiquitin-like modifier-activating enzyme ATG7 |
| AS001752 | contig_41 | NA |
| AS001753 | contig_41 | NA |
| AS001866 | contig_45 | Uncharacterized protein OBRU01_24464 |
| AS001821 | contig_45 | proline-rich protein 4-like |
| AS001848 | contig_45 | PiggyBac transposable element-derived protein 3 |
| AS001849 | contig_45 | NA |
| AS001822 | contig_45 | atypical dual specificity phosphatase |
| AS001978 | contig_57 | transforming growth factor beta regulator 1 |
| AS001979 | contig_57 | menin |
| AS001980 | contig_57 | NA |
| AS002053 | contig_58 | unnamed protein product |
| AS002054 | contig_58 | Dynein heavy chain 3, axonemal |
| AS002167 | contig_58 | synapse-associated protein of 47 kDa isoform X4 |
| AS002182 | contig_58 | uncharacterized protein LOC111349066 |
| AS002313 | contig_64 | unnamed protein product |
| AS002281 | contig_64 | serine protease 42-like isoform X2 |
| AS002282 | contig_64 | dipeptidyl-peptidase 9 |
| AS002290 | contig_64 | hypothetical protein EVAR_35820_1 |
| AS002378 | contig_65 | aminoacylase |
| AS002349 | contig_65 | probable serine/threonine-protein kinase DDB_G0267686 |
| AS002438 | contig_66 | uncharacterized protein LOC113230887 |
| AS002555 | contig_66 | pikachurin-like |
| AS002803 | contig_78 | synaptogenesis protein syg-2-like |
| AS002931 | contig_82 | O-acyltransferase like protein-like |
| AS003137 | contig_82 | Ras-like protein family member 10B |
| AS003138 | contig_82 | hypothetical protein AB894_15215 |
| AS003143 | contig_82 | succinate dehydrogenase (ubiquinone) flavoprotein subunit |
| AS002953 | contig_82 | hypothetical protein B5V51_13448 |
| AS002961 | contig_82 | Retrovirus-related Pol polyprotein from transposon TNT 1-94 |
| AS003253 | contig_82 | NA |
| AS003370 | contig_86 | prickle |
| AS003594 | contig_96 | alkylglycerol monooxygenase |
| AS003557 | contig_96 | unnamed protein product |
| AS003665 | contig_98 | uncharacterized protein LOC115447671 |
| AS003674 | contig_98 | hypothetical protein B5V51_3202 |
| AS003675 | contig_98 | uncharacterized protein LOC116770084 |
| AS003683 | contig_98 | NA |
| AS003684 | contig_98 | hypothetical protein B5X24_HaOG204395 |
| AS003638 | contig_98 | rho GTPase-activating protein conundrum isoform X1 |
| AS003730 | contig_101 | RNA binding protein fox-1 |
| AS003736 | contig_102 | uncharacterized protein LOC110373325 |
| AS003862 | contig_107 | zonadhesin-like isoform X1 |
| AS003863 | contig_107 | unnamed protein product |
| AS003897 | contig_109 | hypothetical protein B5X24_HaOG207692 |
| AS004123 | contig_120 | E3 ubiquitin-protein ligase RNF130 |
| AS004124 | contig_120 | histone acetyltransferase |
| AS004317 | contig_129 | acyl-CoA delta 11 desaturase |
| AS004343 | contig_129 | uncharacterized protein LOC110377500 |
| AS004344 | contig_129 | hypothetical protein evm_014177 |
| AS004407 | contig_134 | unnamed protein product |
| AS004427 | contig_134 | succinate dehydrogenase (ubiquinone) iron-sulfur subunit |
| AS004517 | contig_135 | nose resistant to fluoxetine protein 6-like |
| AS004590 | contig_138 | NA |
| AS004591 | contig_138 | uncharacterized protein LOC114351244 |
| AS004665 | contig_138 | uncharacterized protein LOC114362701 |
| AS004774 | contig_139 | hypothetical protein B5V51_6138 |
| AS004970 | contig_140 | gustatory receptor |
| AS004807 | contig_140 | protein purity of essence |
| AS005247 | contig_151 | NA |
| AS005248 | contig_151 | gamma-glutamyl transpeptidase / glutathione hydrolase / leukotriene-C4 hydrolase |
| AS005306 | contig_152 | uncharacterized protein LOC110371177 |
| AS005420 | contig_153 | succinate dehydrogenase (ubiquinone) flavoprotein subunit |
| AS005421 | contig_153 | uncharacterized protein LOC110373980 |
| AS005435 | contig_153 | uncharacterized protein LOC113225924 |
| AS005481 | contig_153 | atrial natriuretic peptide receptor A |
| AS005499 | contig_153 | uncharacterized protein LOC110371277 |
| AS005783 | contig_156 | endonuclease-reverse transcriptase |
| AS005784 | contig_156 | membrane dipeptidase |
| AS005785 | contig_156 | sorting nexin-29 |
| AS005786 | contig_156 | ATP-binding cassette |
| AS005842 | contig_159 | acidic fibroblast growth factor intracellular-binding protein |
| AS005843 | contig_159 | NA |
| AS005854 | contig_159 | 26S proteasome regulatory subunit N9 |
| AS005863 | contig_159 | NA |
| AS005864 | contig_159 | putative tick transposon |
| AS005865 | contig_159 | NA |
| AS005906 | contig_160 | calcium/calmodulin-dependent 3',5'-cyclic nucleotide phosphodiesterase |
| AS005933 | contig_162 | NA |
| AS005978 | contig_162 | leucine-rich repeat kinase 1 |
| AS005939 | contig_162 | aldehyde reductase |
| AS006010 | contig_162 | endocuticle structural glycoprotein ABD-4-like |
| AS006011 | contig_162 | MFS transporter |
| AS006185 | contig_168 | MFS transporter |
| AS006272 | contig_169 | uncharacterized protein LOC105380103 |
| AS006200 | contig_169 | uncharacterized protein LOC113498988 |
| AS006201 | contig_169 | optic atrophy protein 1 |
| AS006214 | contig_169 | nuclear pore complex protein Nup88 |
| AS006517 | contig_171 | uncharacterized protein LOC114355921 |
| AS006951 | contig_174 | uncharacterized protein LOC113503947 |
| AS006952 | contig_174 | uncharacterized protein LOC110379268 |
| AS006953 | contig_174 | uncharacterized protein LOC113503947 |
| AS006878 | contig_174 | tyrosine-protein kinase Abl |
| AS006982 | contig_174 | glutamate receptor 3 |
| AS007197 | contig_176 | NA |
| AS007220 | contig_179 | uncharacterized protein LOC111356679 |
| AS007293 | contig_185 | unnamed protein product |
| AS007395 | contig_193 | uncharacterized protein LOC113507973 |
| AS007396 | contig_193 | hypothetical protein evm_003375 |
| AS007467 | contig_194 | liprin-alpha-1 isoform X9 |
| AS007558 | contig_197 | THUMP domain-containing protein 1 homolog |
| AS007621 | contig_198 | hypothetical protein B5V51_13509 |
| AS007622 | contig_198 | GPI transamidase component PIG-T |
| AS007623 | contig_198 | ubiquitin carboxyl-terminal hydrolase L3 |
| AS007624 | contig_198 | major facilitator superfamily domain-containing protein 12-like isoform X1 |
| AS007634 | contig_198 | very low-density lipoprotein receptor |
| AS007901 | contig_204 | synaptic vesicle glycoprotein 2B-like |
| AS007912 | contig_204 | hypothetical protein B5X24_HaOG213278 |
| AS008236 | contig_216 | NA |
| AS008240 | contig_216 | uncharacterized protein LOC111357782 |
| AS008255 | contig_216 | Uncharacterized protein OBRU01_10652 |
| AS008266 | contig_216 | NA |
| AS008364 | contig_218 | NA |
| AS008527 | contig_219 | Retrovirus-related Pol polyprotein from transposon 297 |
| AS008694 | contig_227 | carboxypeptidase Q |
| AS008695 | contig_227 | sarcosine oxidase / L-pipecolate oxidase |
| AS008692 | contig_227 | cytochrome P450 |
| AS008693 | contig_227 | DNA-directed RNA polymerase II subunit RPB1 |
| AS008727 | contig_228 | hypothetical protein B5V51_9330 |
| AS008742 | contig_230 | NA |
| AS008833 | contig_235 | 30S ribosomal protein S10 |
| AS008792 | contig_235 | pancreatic lipase-related protein 2 |
| AS008900 | contig_236 | cAMP-specific phosphodiesterase 4 |
| AS008962 | contig_236 | vesicular glutamate transporter 1 isoform X1 |
| AS009032 | contig_237 | neuropeptide FF receptor 2 |
| AS009044 | contig_237 | RNA-directed DNA polymerase |
| AS009104 | contig_238 | NA |
| AS009168 | contig_239 | Uncharacterized protein OBRU01_06070 |
| AS009183 | contig_239 | uncharacterized protein LOC115452494 |
| AS009184 | contig_239 | Retrovirus-related Pol polyprotein from transposon TNT 1-94 |
| AS009187 | contig_239 | dedicator of cytokinesis protein 1 isoform X3 |
| AS009127 | contig_239 | hypothetical protein B5V51_1238 |
| AS009128 | contig_239 | uncharacterized protein LOC111357260 |
| AS009237 | contig_240 | RecName: Full=Neuropeptide CCHamide-2; Short=CCHa; Flags: Precursor |
| AS009441 | contig_244 | calmodulin-binding transcription activator 2 |
| AS009673 | contig_250 | growth arrest-specific 1 |
| AS009575 | contig_250 | NA |
| AS009576 | contig_250 | NA |
| AS009577 | contig_250 | chemosensory protein 16 |
| AS009818 | contig_251 | uncharacterized protein LOC111361297 |
| AS009819 | contig_251 | hypothetical protein B5V51_679 |
| AS009885 | contig_253 | hypothetical protein B5V51_10833 |
| AS009896 | contig_254 | cytochrome P450 |
| AS009902 | contig_254 | NA |
| AS009903 | contig_254 | phosphoinositide-3-kinase |
| AS009904 | contig_254 | NA |
| AS009937 | contig_256 | succinate dehydrogenase (ubiquinone) flavoprotein subunit |
| AS010055 | contig_260 | NA |
| AS010060 | contig_260 | uncharacterized protein K02A2.6-like |
| AS010062 | contig_260 | uncharacterized protein LOC113495927 |
| AS010109 | contig_262 | phosphoinositide-3-kinase |
| AS010110 | contig_262 | gonadotropin-releasing hormone receptor |
| AS010252 | contig_268 | hypothetical protein B5X24_HaOG206492 |
| AS010376 | contig_270 | monocarboxylate transporter 12-like |
| AS010396 | contig_274 | Retrovirus-related Pol polyprotein from transposon TNT 1-94 |
| AS010406 | contig_274 | NA |
| AS010407 | contig_274 | uncharacterized protein LOC113238652 |
| AS010408 | contig_274 | hypothetical protein B5V51_12759 |
| AS010756 | contig_297 | glutathione S-transferase |
| AS010757 | contig_297 | glutathione S-transferase |
| AS010949 | contig_308 | importin-11 isoform X1 |
| AS011053 | contig_319 | hypothetical protein evm_015016 |
| AS011037 | contig_319 | uncharacterized protein LOC106116894 |
| AS011038 | contig_319 | Pol protein |
| AS011039 | contig_319 | hypothetical protein B5V51_10841 |
| AS011056 | contig_319 | hypothetical protein B5V51_2999 |
| AS011057 | contig_319 | NA |
| AS011043 | contig_319 | hypothetical protein B5V51_1590 |
| AS011103 | contig_319 | hypothetical protein B5X24_HaOG203910 |
| AS011340 | contig_328 | hypothetical protein RF55_22593 |
| AS011367 | contig_330 | major facilitator superfamily domain-containing protein 9-like |
| AS011415 | contig_334 | uncharacterized protein LOC111356792 |
| AS011416 | contig_334 | SWI/SNF-related matrix-associated actin-dependent regulator of chromatin subfamily A-like protein 1 |
| AS011466 | contig_336 | hypothetical protein B5V51_9932 |
| AS011625 | contig_338 | uncharacterized protein LOC110370010 |
| AS011626 | contig_338 | caspase 7 |
| AS011627 | contig_338 | uncharacterized protein LOC110370008 |
| AS011628 | contig_338 | probable chitinase |
| AS011743 | contig_338 | hypothetical protein B5V51_2643 |
| AS011579 | contig_338 | hypothetical protein B5V51_7125 |
| AS011862 | contig_340 | acylphosphatase |
| AS011863 | contig_340 | RNA-directed DNA polymerase |
| AS011864 | contig_340 | phosphoglucomutase-2 |
| AS011812 | contig_340 | succinate dehydrogenase (ubiquinone) flavoprotein subunit |
| AS011915 | contig_343 | scavenger receptor class B |
| AS011921 | contig_343 | hypothetical protein B5V51_11304 |
| AS011922 | contig_343 | hypothetical protein B5V51_11304 |
| AS011923 | contig_343 | hypothetical protein B5V51_11304 |
| AS011924 | contig_343 | neurofibromin isoform |
| AS011967 | contig_345 | waprin-Phi1-like |
| AS011950 | contig_345 | Ca-activated chloride channel homolog |
| AS011951 | contig_345 | Rho GTPase-activating protein 1 |
| AS011954 | contig_345 | pre-mRNA-processing factor 17 |
| AS012056 | contig_352 | gustatory receptor |
| AS012057 | contig_352 | uncharacterized protein LOC111357782 |
| AS012046 | contig_352 | NA |
| AS012047 | contig_352 | NA |
| AS012048 | contig_352 | phosphate carrier protein, mitochondrial-like |
| AS012262 | contig_364 | aquaporin rerated protein |
| AS012268 | contig_364 | ubiquitin-conjugating enzyme E2 S |
| AS012269 | contig_364 | uncharacterized protein LOC110383465 |
| AS012270 | contig_364 | hypothetical protein B5V51_10673 |
| AS012288 | contig_365 | NA |
| AS012447 | contig_377 | tyrosine-protein phosphatase non-receptor type 23 |
| AS012448 | contig_377 | hypothetical protein B5X24_HaOG214372 |
| AS012432 | contig_377 | uncharacterized protein LOC114362701 |
| AS012483 | contig_378 | G protein-coupled receptor 84 |
| AS012579 | contig_414 | uncharacterized protein LOC110380914 |
| AS012823 | contig_448 | paired box protein 2 |
| AS012976 | contig_481 | CD63 antigen |
| AS012978 | contig_481 | translation initiation factor 4G |
| AS013005 | contig_481 | NA |
| AS013016 | contig_481 | hypothetical protein B5X24_HaOG206415 |
| AS013250 | contig_489 | uncharacterized protein LOC110383558 |
| AS013251 | contig_489 | phosphopantothenate-cysteine ligase |
| AS013284 | contig_493 | division abnormally delayed protein |
| AS013301 | contig_496 | zwei Ig domain protein zig-8-like isoform X5 |
| AS013293 | contig_496 | gustatory receptor |
| AS013294 | contig_496 | hypothetical protein B5V51_10841 |
| AS013343 | contig_499 | NA |
| AS013391 | contig_505 | NA |
| AS013347 | contig_505 | NA |
| AS013588 | contig_515 | protein toll |
| AS013592 | contig_515 | uncharacterized protein LOC112053638 |
| AS013593 | contig_515 | hypothetical protein B5V51_3863 |
| AS013776 | contig_549 | fatty acid synthase |
| AS014129 | contig_569 | hypothetical protein B5X24_HaOG205504 |
| AS014114 | contig_569 | inositol-1,4,5-trisphosphate 5-phosphatase |
| AS014241 | contig_577 | pyruvate |
| AS014313 | contig_601 | pyrroline-5-carboxylate reductase |
| AS014336 | contig_601 | endothelin-converting enzyme |
| AS014390 | contig_603 | NA |
| AS014391 | contig_603 | NA |
| AS014431 | contig_604 | hypothetical protein B5V51_13862 |
| AS014526 | contig_608 | putative Copia protein |
| AS014545 | contig_609 | hypothetical protein evm_013355 |
| AS014544 | contig_609 | uncharacterized protein LOC114240369 |
| AS014723 | contig_633 | uncharacterized protein K02A2.6-like |
| AS014724 | contig_633 | endoribonuclease Dicer |
| AS014784 | contig_633 | WD and tetratricopeptide repeats protein 1 |
| AS014733 | contig_633 | hypothetical protein B5V51_11567 |
| AS014734 | contig_633 | uncharacterized protein LOC113399197 |
| AS014967 | contig_652 | uncharacterized protein LOC111359891 |
| AS015020 | contig_654 | uncharacterized protein LOC110373470 isoform X2 |
| AS014992 | contig_654 | optic atrophy protein 1 |
| AS014994 | contig_654 | ribosome production factor 2 |
| AS014995 | contig_654 | endonuclease-reverse transcriptase |
| AS014996 | contig_654 | optic atrophy protein 1 |
| AS015146 | contig_677 | hypothetical protein B5X24_HaOG209551 |
| AS015299 | contig_711 | NA |
| AS015523 | contig_772 | NA |
| AS015540 | contig_773 | optic atrophy protein 1 |
| AS015584 | contig_783 | NA |
| AS015679 | contig_801 | solute carrier family 26 (sodium-independent sulfate anion transporter) |
| AS015685 | contig_804 | mannosyl-glycoprotein endo-beta-N-acetylglucosaminidase |
| AS015690 | contig_804 | NA |
| AS015691 | contig_804 | NA |
| AS015827 | contig_843 | cytochrome P450 |
| AS015838 | contig_843 | hypothetical protein B5V51_12644 |
| AS015839 | contig_843 | protein MAK16 |
| AS015847 | contig_843 | tenascin-like isoform X3 |
| AS016007 | contig_901 | voltage-gated potassium channel |
| AS016111 | contig_932 | intermediate filament protein if |
| AS016112 | contig_932 | AP-3 complex subunit mu |
| AS016187 | contig_944 | NA |
| AS016242 | contig_957 | SWI/SNF-related matrix-associated actin-dependent regulator of chromatin subfamily A-like protein 1 |
| AS016395 | contig_1036 | hypothetical protein B5X24_HaOG212633 |
| AS016396 | contig_1036 | lysosomal-associated transmembrane protein |
| AS016484 | contig_1063 | allatostatin neuropeptide precursor |
| AS016485 | contig_1063 | hypothetical protein B5X24_HaOG211194 |
| AS016522 | contig_1074 | protein 60A |
| AS016722 | contig_1108 | NA |
| AS016706 | contig_1108 | polyprotein |
| AS016707 | contig_1108 | xanthine dehydrogenase/oxidase |
| AS016848 | contig_1203 | heat shock 70kDa protein 1/8 |
| AS016849 | contig_1203 | heat shock 70kDa protein 1/8 |
| AS016850 | contig_1203 | gustatory receptor |
| AS016851 | contig_1203 | heat shock 70kDa protein 1/8 |
| AS016994 | contig_1285 | NA |
| AS017325 | contig_1517 | heparan sulfate 2-O-sulfotransferase HS2ST1 |
| AS017388 | contig_1541 | NA |
| AS017405 | contig_1549 | hemicentin |
| AS017406 | contig_1549 | poly(U)-binding-splicing factor PUF60 |
| AS017613 | contig_1664 | thyrostimulin beta-5 subunit-like |
| AS017711 | contig_1676 | hypothetical protein B5V51_2366 |
| AS017752 | contig_1738 | 26S proteasome regulatory subunit N8 |
| AS017811 | contig_1856 | hypothetical protein B5V51_13811 |
| AS017824 | contig_1873 | uncharacterized protein LOC116770131 |
| AS017825 | contig_1873 | hypothetical protein B5X24_HaOG204786 |
| AS017826 | contig_1873 | NA |
| AS017822 | contig_1873 | NIMA (never in mitosis gene a)-related kinase |
| AS017863 | contig_1880 | uncharacterized protein LOC116774539 |
| AS018077 | contig_1895 | fatty acid synthase |
| AS018106 | contig_1896 | WD and tetratricopeptide repeats protein 1 |
| AS018208 | contig_1907 | NA |
| AS018416 | contig_1957 | kinesin family member 18/19 |
| AS018500 | contig_2036 | NA |
| AS018501 | contig_2036 | NA |
| AS018511 | contig_2043 | potassium large conductance calcium-activated channel subfamily M alpha member 1 |

Notes: NA (not applicable).

**Table S25. Regional and environmental data for environmental correlation analysis**

| **Population ID** | **Latitude** | **Annual Mean Temperature** | **Min Temperature of Coldest Month** |
| --- | --- | --- | --- |
| XJ | 41.7 | 11.5 | -12.4 |
| YN | 25.6 | 15.7 | 1.6 |
| HN | 34.7 | 14.3 | -4.8 |
| HB | 39.5 | 12.1 | -9.2 |
| SD | 37.5 | 12.5 | -4.5 |
| HLJ | 45.5 | 3.3 | -26 |

**Table S26. Strong associated genes in SNPs-environment association analysis using GEMMA**

| **Term** | **Gene ID** | **Contig** | ***p*-value** | **Annotation** |
| --- | --- | --- | --- | --- |
| Annual Mean Temperature | AS011608 | contig_338 | 5.23E-16 | nucleosome-remodeling factor subunit NURF |
|  | AS015015 | contig_654 | 2.18E-14 | ATP synthase subunit beta |
|  | AS011726 | contig_338 | 5.09E-14 | RNA-binding protein Musashi homolog Rbp6 |
|  | AS016098 | contig_932 | 7.62E-14 | chromodomain-helicase-DNA-binding protein 7 |
|  | AS012027 | contig_345 | 8.14E-13 | cytochrome P450 |
|  | AS016105 | contig_932 | 1.43E-12 | WD repeat-containing protein 55 |
| Min Temperature of Coldest Month | AS011608 | contig_338 | 1.78E-14 | nucleosome-remodeling factor subunit NURF |
|  | AS015015 | contig_654 | 5.69E-12 | ATP synthase subunit beta |
|  | AS000386 | contig_9 | 5.75E-12 | NA |
|  | AS016098 | contig_932 | 1.01E-11 | chromodomain-helicase-DNA-binding protein 7 |
|  | AS015014 | contig_654 | 1.13E-11 | 60S ribosomal export protein NMD3 |
|  | AS011726 | contig_338 | 2.41E-11 | RNA-binding protein Musashi homolog Rbp6 |

Notes: NA (not applicable).

**Table S27. Strong associated genes in SVs-environment association analysis using GEMMA**

| **Term** | **Gene ID** | **Contig** | ***p*-value** | **Annotation** | **Type** | **SNP** |
| --- | --- | --- | --- | --- | --- | --- |
| Latitude | AS006396 | contig_171 | 9.63E-11 | coiled-coil domain-containing protein AGAP005037 | DEL | √ |
|  | AS002712 | contig_76 | 1.02E-08 | oxysterol-binding protein-related protein 3/6/7 | DEL | √ |
|  | AS011775 | contig_339 | 7.54E-08 | exportin-1 | DEL | √ |
|  | AS013371 | contig_505 | 2.82E-07 | odorant binding protein | DEL | √ |
|  | AS013370 | contig_505 | 3.11E-07 | odorant binding protein | DEL | √ |
|  | AS006305 | contig_169 | 8.65E-07 | fatty acid synthase | DEL | √ |
|  | AS007242 | contig_183 | 1.09E-06 | uncharacterized protein LOC111357700 | DEL | √ |
|  | AS004632 | contig_138 | 1.35E-06 | peroxidase | DEL | NA |
|  | AS018442 | contig_1972 | 1.36E-06 | ecdysone-responsive G-protein coupled protein-2 | DEL | NA |
| Annual Mean Temperature | AS007234 | contig_183 | 1.10E-07 | hypothetical protein B5V51_5409 | DEL | NA |
|  | AS016105 | contig_932 | 4.29E-08 | WD repeat-containing protein 55 | DEL | √ |
| Min Temperature of Coldest Month | AS007234 | contig_183 | 7.28E-08 | hypothetical protein B5V51_5409 | DEL | NA |
|  | AS016105 | contig_932 | 2.64E-07 | WD repeat-containing protein 55 | DEL | √ |
